# Alkenyl-Functionalized Open-Cage Silsesquioxanes (RSiMe_2_O)_3_R′_7_Si_7_O_9_: A Novel Class of Building Nanoblocks

**DOI:** 10.1021/acs.inorgchem.1c00689

**Published:** 2021-06-16

**Authors:** Kinga Stefanowska, Jakub Szyling, Jędrzej Walkowiak, Adrian Franczyk

**Affiliations:** † Center for Advanced Technology, Adam Mickiewicz University in Poznań, Uniwersytetu Poznańskiego 10, 61-614 Poznań, Poland; ‡ Faculty of Chemistry, Adam Mickiewicz University in Poznań, Uniwersytetu Poznańskiego 8, 61-614 Poznań, Poland

## Abstract

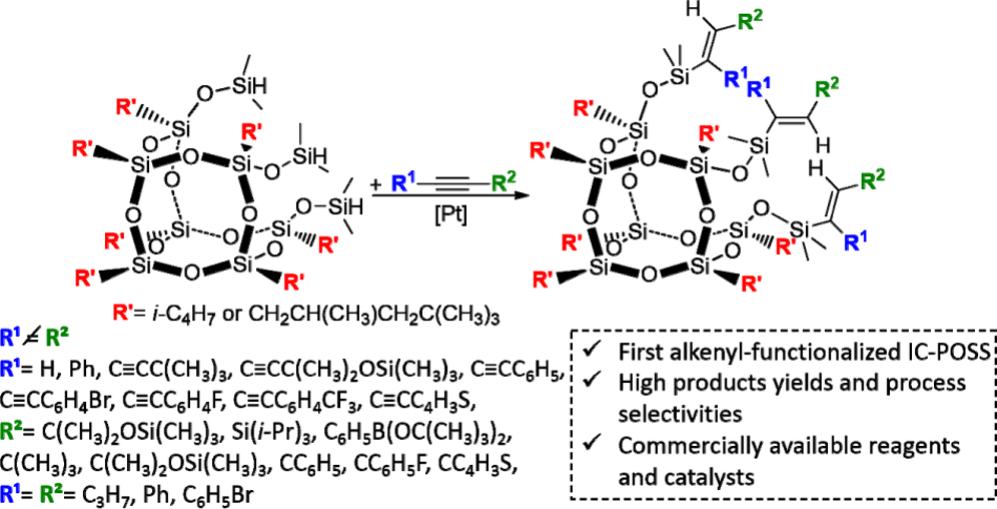

Trifunctional incompletely condensed polyhedral oligomeric silsesquioxanes (RSiMe_2_O)_3_R′_7_Si_7_O_9_ (**IC-POSS**s) are considered as intriguing building nanoblocks dedicated to constructing highly advanced organic–inorganic molecules and polymers. Up to now, they have been mainly obtained *via* hydrosilylation of olefins, while the hydrosilylation of the C≡C bonds has not been studied at all, despite the enormous potential of this approach resulting from the possibility of introducing 3, 6, or even more functional groups into the **IC-POSS** structure. Therefore, in this work, we present a highly selective and efficient synthesis of the first example of tripodal alkenyl-functionalized **IC-POSS**s, obtained *via* platinum-catalyzed hydrosilylation of the terminal and internal alkynes, as well as symmetrically and nonsymmetrically 1,4-disubstituted buta-1,3-diynes with silsesquioxanes (HSiMe_2_O)_3_R′_7_Si_7_O_9_ (R′ = *i*-C_4_H_9_ (**1a**), (H_3_C)_3_CH_2_C(H_3_C)HCH_2_C (**1b**)). The resulting products are synthetic intermediates that contain C=C bonds and functional groups (e.g., OSiMe_3_, SiR_3_, Br, F, B(O(C(CH_3_)_2_)_2_ (Bpin)), thienyl), which make them suitable for application in the synthesis of novel, complex, hybrid materials with unique properties.

## Abstract 2

The first example of the synthesis of alkenyl-functionalized open-cage silsesquioxanes (**IC-POSS**) via platinum-catalyzed hydrosilylation of C−C triple bonds in alkynes and buta-1,3-diynes is presented. The optimized synthetic procedure allowed for the selective and efficient synthesis of 20 new functional molecules capable of further modification by hydrosilylation, hydroboration, or other chemical processes.

## Introduction

Trifunctional incompletely condensed silsesquioxanes (RSiMe_2_O)_3_R′_7_Si_7_O_9_ (**IC-POSS**s) have attracted much attention since they were first recognized as building nanoblocks for the synthesis of advanced hybrid materials.^1−8^ These compounds, based on the silicon–oxygen cubic core in which one corner is open, inherit many features of completely condensed polyhedral oligomeric silsesquioxanes (R_8_Si_8_O_12_, POSS) and at the same time possess unique properties that can give them an advantage over the POSS in some areas of application. For instance, it was found that open-cage structures **IC-POSS**s are characterized by excellent thermal stability, similar to their POSS analogues. However, because of low symmetry, their melting points are remarkably lowered.^9^ This effectively restricts crystallinity,^9−11^ and they are much better dispersed in polymer matrixes,^11^ compared to completely condensed POSS, which are more prone to aggregation.^12,13^

The leading representatives of trifunctional **IC-POSS**s are commercially available trisilanols (HO)_3_R′_7_Si_7_O_9_ (R′ = Et, *i*-C_4_H_7_, CH_2_CH(CH_3_)CH_2_C(CH_3_)_3_ or Ph, trisilanol-POSS).^14^ They have been prepared by hydrolytic condensation of RSiX_3_ (X = Cl, OR, etc.)^15−17^ or by the controlled cleavage of R_8_Si_8_O_12_.^18−20^ The (HO)_3_R′_7_Si_7_O_9_ have been used as models for the silica surfaces,^7,8,21^ dispersants in polymer matrixes,^22−24^ reactive additives (which improve the moduli and thermal stability of composites),^25−27^ components for the preparation of noncrystalline poly(silsesquioxane)s,^28^ as well as in biomedical studies focused on the tissue healing.^29^ However, most of the published reports still have concern for their use as the main intermediates for the synthesis of completely condensed monofunctionalized silsesquioxanes RR′_7_Si_8_O_12_ (R = reactive group, R′ = inert group)^30,31^ or **IC-POSS**s with a wide variety of functionalities situated at the opening moieties.^9,11,32−41^

The most common starting reagents for the synthesis of trifunctional **IC-POSS** compounds are (RSiMe_2_O)_3_R′_7_Si_7_O with R = H or HC=CH_2_ groups. Their modification *via* hydrosilylation processes led to a very rich group of new derivatives.^9,11,34−36^ They have been used as effective emulsifiers for the synthesis of stable oil-in-water emulsions,^9^ nanofillers for tuning properties of optically transparent polymer materials, stabilizers of a quantum dot (binding ligand in nanocrystalline electroluminescent materials),^42^ cross-linking agents in binders, hot-melt adhesives,^43^ insoluble Langmuir films,^44^ and monomers in the synthesis of high-temperature resistance polymers.^34,35^ They were also employed in the manufacture of liquid-crystal displays,^45^ photosensitive materials,^46−48^ optical fibers, and materials.^49,50^

All of the above-mentioned studies have concern for the use of trifunctional **IC-POSS**s obtained only by the hydrosilylation of carbon–carbon double bond (C=C), in which the research was focused on the uses of the desired products, and in most cases, no optimization of the reaction conditions was made. Therefore, there is still a great need for developing the synthetic approaches leading to new compounds, which will open areas of research not available so far. One of them is the hydrosilylation of the carbon–carbon triple bonds (C≡C) in alkynes and 1,3-diynes. This method together with hydrosilylation of functional olefins seems to be one of the most powerful tools, which, when used appropriately, can easily provide a multiplicity of functional **IC-POSS**s.^51,52^ The obtained compounds possess C=C bond(s) and other functional groups that can be easily modified by addition and condensation reactions, Sonogashira, Suzuki, or Heck couplings, as well as they can be used as monomers or initiators in atom transfer radical polymerization (ATRP) or reagents in click chemistry.^53−56^ Such alkenyl-functionalized **IC-POSS**s constitute excellent precursors for the construction of advanced hybrid materials, for instance, dedicated to optoelectronics.^57−60^

Therefore, in this work, we decided to describe the synthesis and characterization of new tripodal alkenyl-functionalized **IC-POSS**s afforded by hydrosilylation of alkynes and more challenging symmetrical and nonsymmetrical 1,4-disubstituted buta-1,3-diynes with silsesquioxanes (HSiMe_2_O)_3_R′_7_Si_7_O_9_ (R′ = *i*-C_4_H_9_ (**1a**), (H_3_C)_3_CH_2_C(H_3_C)HCH_2_C (**1b**)). The application of two different silsesquioxane substrates allowed obtaining compounds characterized by different physical properties and checking if the type of inert groups in the **IC**-**POSS** structure has an impact on the time and selectivity of the processes. It should be mentioned that substrates **1a** and **1b** can be easily synthesized with high yields via the previously reported methods, which is an additional advantage of the synthetic protocols proposed in this manuscript.^33,61^

## Results and Discussion

Firstly, we investigated the hydrosilylation of terminal alkynes ([(1,1-dimethyl-2-propynyl)oxy]trimethylsilane (**2a**) and tri(*iso*-propyl)silylacetylene (**2b**)) with silsesquioxanes (HSiMe_2_O)_3_R′_7_Si_7_O_9_ (R′ = *i*-C_4_H_9_ (**1a**) or (H_3_C)_3_CH_2_C(H_3_C)HCH_2_C (**1b**)). In our experiments, we used commercially available platinum catalysts: Karstedt’s catalyst (Pt_2_(dvs)_3_ (**I**), PtO_2_/XPhos (XPhos = 2-dicyclohexylphosphino-2′,4′,6′-tri(*iso*-propyl)biphenyl) (**II**), and Pt(PPh_3_)_4_ (**III**)) (Table 1, entries 1–6). The reactions were carried out with reagents in a ratio [**1**]:[**2**] = 1:3, in toluene or tetrahydrofuran (THF), at 100 °C, without any purification of the acquired chemicals. The progress of the reactions was monitored by ^1^H NMR after 24 h, while the process selectivity was calculated using ^1^H and ^29^Si NMR.

**Table 1:**
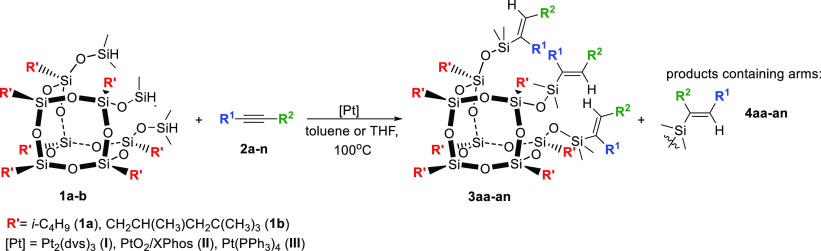

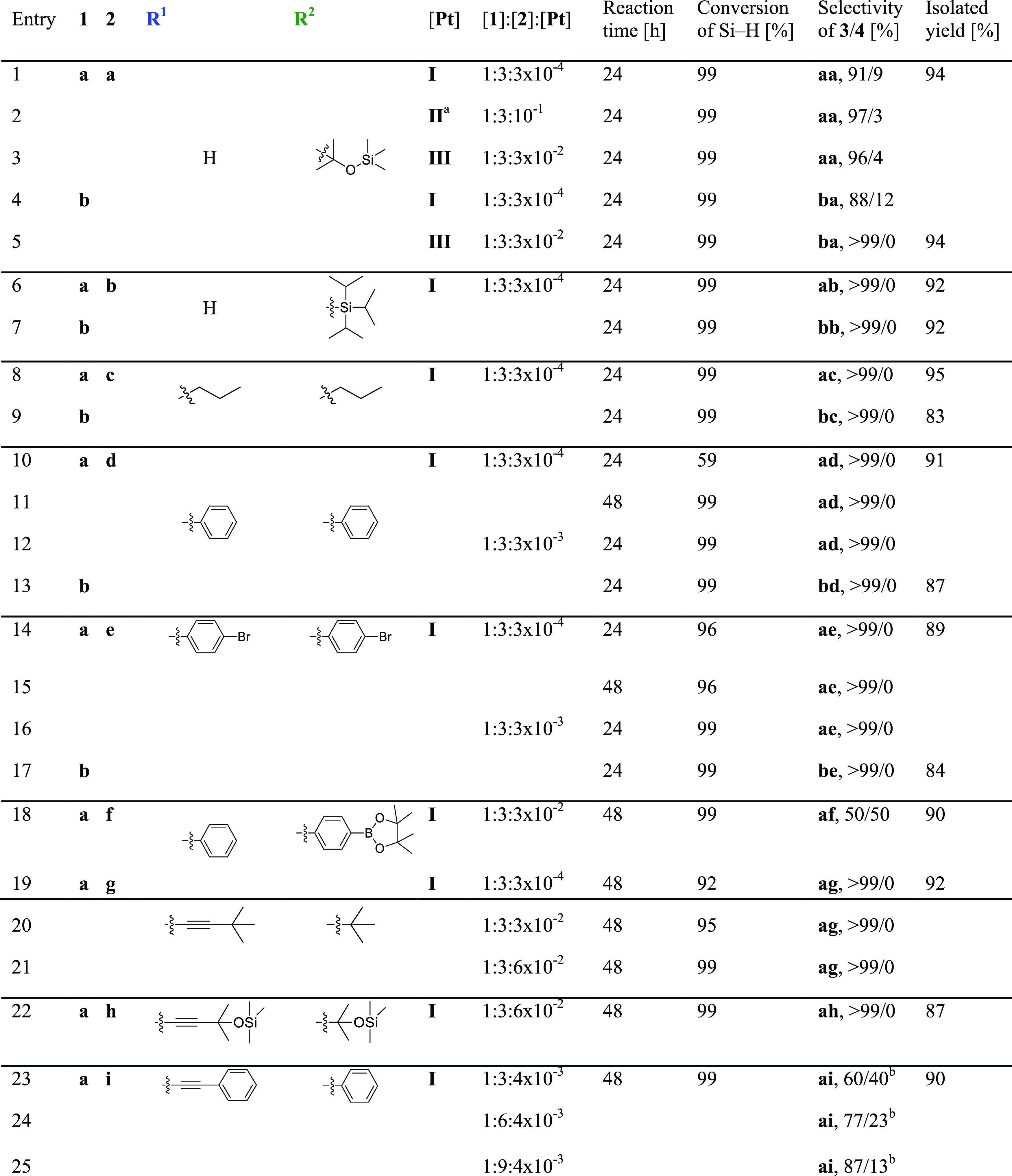

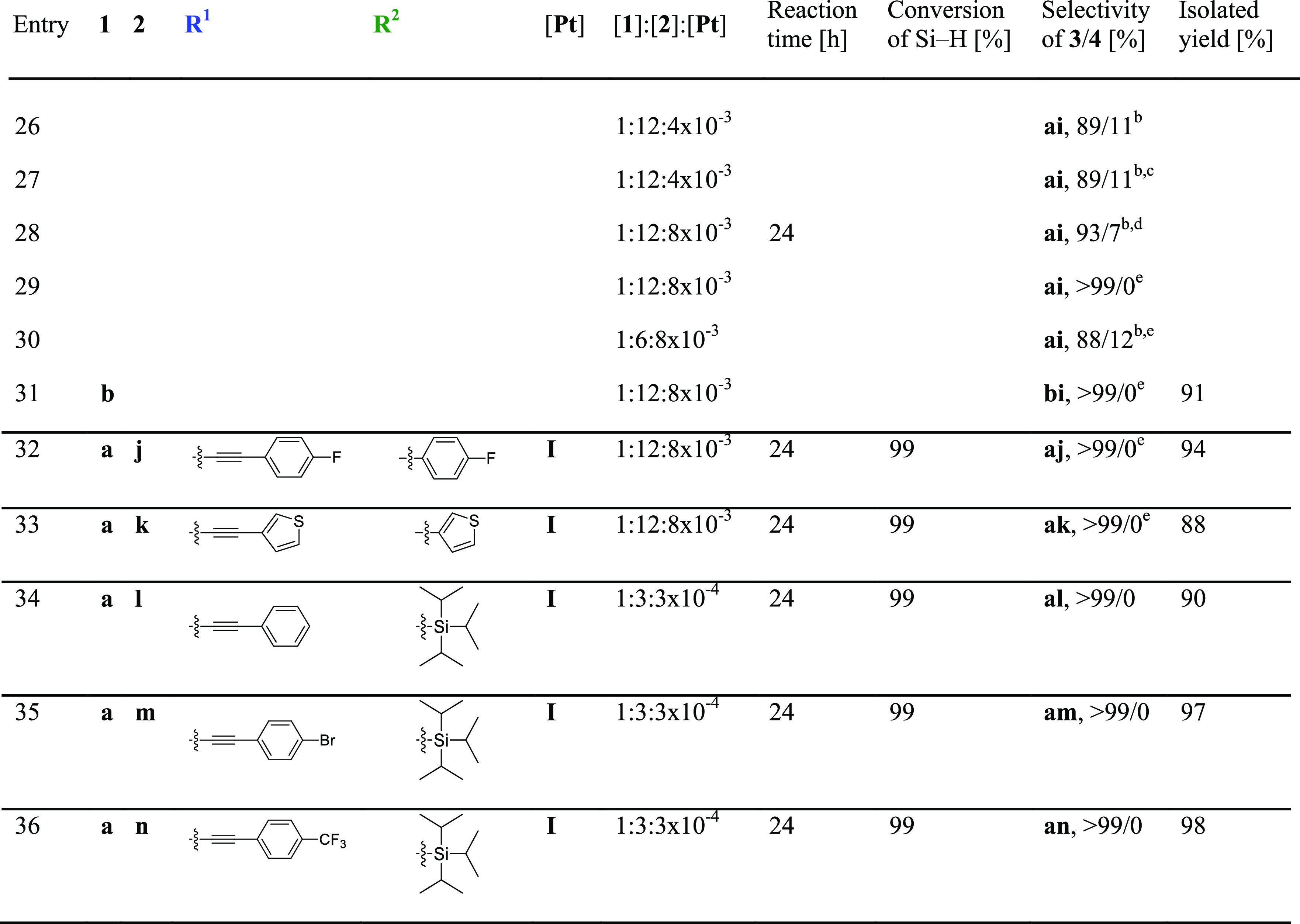
Hydrosilylation of Alkynes **2a**–**f** and 1,3-Diynes **2g**–**n** with **IC-POSS**s **1a,b**^f^

a *m*_s(1)_/*V*_THF_ = 50 mg mL^–1^, argon; 2 × 10^–1^ mol of XPhos was added.

b Instead of Z-isomers, bishydrosilylated products were formed.

c 60 °C.

d 40 °C.

e *m*_s(1)_/*V*_tol._ = 100 mg mL^–1^, 40 °C. Conversions of reagents were determined by ^1^H NMR; the selectivity for all experiments was confirmed by ^1^H, ^13^C, ^29^Si NMR, Fourier transform infrared (FT-IR), and MALDI time-of-flight (TOF). The isolated yield of products = 83–95% (see the Supporting Information (SI)).

f Reaction conditions: 100 °C, *m*_s(1)_/*V*_tol._ = 50 mg mL^–1^ (where *m*_S(1)_ is the mass of the substance **1a** or **1b**).

The hydrosilylation of [(1,1-dimethyl-2-propynyl)oxy]trimethylsilane (**2a**) with silsesquioxanes **1a**,**b** carried out in the presence of Karstedt’s catalyst (**I**) resulted in the formation of products **3aa** and **3ba** with selectivities of 91 and 88%, respectively. Traces of α-isomers (**4aa, 4ba**) were noticed. The selectivity of the synthesis of **3aa** was improved up to 97% when the PtO_2_/XPhos (**II**) system^62−65^ was used (Table 1, entry 2). A similar result was obtained when the process was carried out in the presence of Pt(PPh_3_)_4_ (**III**), 96% (Table 1, entry 3). Moreover, the application of Pt(PPh_3_)_4_ (**III**) allowed reducing the catalyst loading to 3 × 10^–2^ mol of Pt per mol of SiH. The same catalyst was used in the hydrosilylation of **2a** with **1b** and led exclusively to product **3ba** (>99%) (Table 1, entry 5). The processes with sterically more hindered tri(*iso-*propyl)silylacetylene (**2b**) resulted in the formation of products **3ab** and **3bb** already using Karstedt’s (**I**) catalyst.

Based on the obtained results, we can perceive a relationship between the type of alkyne and the type of catalyst that needs to be used to obtain the products with high regioselectivity. In the hydrosilylation of alkyne **2a**, it was necessary to use the catalysts that possess bulky triarylphosphine (PPh_3_) and dialkylarylphosphine (XPhos) ligands in their structures to impart a high level of process selectivity. The improvement of the selectivity of the hydrosilylation of terminal alkynes by use of the Pt catalyst associated with bulky ligands has been already widely reported in the literature.^62,63,65−68^ On the other hand, when more sterically congested alkyne **2b** was hydrosilylated, the application of the commonly used Karstedt’s catalyst in this process was sufficient to selectively obtain products **3ab** and **3bb**.

In the next step, we decided to study hydrosilylation of internal symmetrical and nonsymmetrical alkynes (4-octyne (**2c**), 1,2-diphenylacetylene (**2d**), bis(4-bromophenyl)acetylene (**2e**), 4-(phenylethynyl)phenylboronic acid pinacol ester (**2f**), Table 1, entries 8–18).

The hydrosilylation of symmetrically disubstituted internal alkynes **2c**–**e** with **1a** and **1b** in the presence of Pt_2_(dvs)_3_ (**I**) (3 × 10^–4^–3 × 10^–2^ Pt/ mol of SiH) demonstrated the selective formation of products **3ac**–**ae** (Table 1, entries 8–17). Along with the increase of the steric hindrance and the presence of functional groups in the structure of alkyne, the time needed to achieve full reagent conversion increased, and a higher catalyst concentration was needed.

In the hydrosilylation of unsymmetrically disubstituted 4-(phenylethynyl)phenylboronic acid pinacol ester (**2f**) with silsesquioxane **1a**, the mixtures of products **3af**/**4af** were obtained in an equal ratio of 50/50 (Table 1, entry 18). The reason for this is the presence of almost the same aryl substituents in the structure, which cannot be recognized by catalysts.

The synthetic methods described are the unique and direct ways for the synthesis of 1,2-(*E*)-disubstituted and 1,1,2-(*E*)-trisubstituted alkenyl-functionalized **IC-POSS**s, allowing for the introduction of three, six, or even more the same (hydrosilylation of symmetrically disubstituted C≡C) or different (hydrosilylation of unsymmetrically disubstituted C≡C) organic functional substituents into the tripodal **IC-POSS** structures. To date, this group of compounds cannot be directly synthesized via any other synthetic methods. Moreover, the obtained novel products (**3aa**–**af**) can be considered as useful and versatile building blocks, in which further transformation of unsaturated C=C bonds and/or other functionalities such as boron pinacol ester or blocked OH might occur.

Encouraged by the results from the hydrosilylation of alkynes, we decided to use this approach to perform the hydrosilylation of much more complex and challenging reagents, namely, symmetrically and nonsymmetrically 1,4-disubstituted buta-1,3-diynes.

First, the hydrosilylation of 2,2,7,7-tetramethyl-3,5-octadiyne (**2g**) and 1,4-(1,1-dimethyloxy-trimethysilyl)buta-1,3-diyne (**2h**) with silsesquioxane (HSiMe_2_O)_3_(*i*-C_4_H_7_)_7_Si_7_O_9_ (**1a**) was performed in the presence of Karstedt′s catalyst with the equimolar stoichiometry [**1a**]/[**2g** or **2h**]/**[Pt]** = 1:3:6 × 10^–2^. It was found that in both cases the reaction exclusively led to the products of the 1,2-addition of SiH group to one of the two C≡C bonds in diyne molecule (**3ag** and **3ah**; Table 1, entries 20 and 21). Analogue influence of the *t*-Bu and (CH_3_)_2_OSi(CH_3_)_3_ groups on forming the product of monohydrosilylation of 1,3-diynes was previously observed.^69,70^

Subsequently, the hydrosilylation of 1,4-diphenylbuta-1,3-diyne (**2i**), 1,4-di(4-fluorophenyl)buta-1,3-diyne (**2j**), and 1,4-bis(thiophen-3-yl)buta-1,3-diyne (**2k**) was performed (Table 1, entries 23–33). It turned out that reactions of diaryl-1,3-diynes with aryl substituents resulted in the mixture of mono- and bissilylated products. However, the addition of the 12-fold excess of diyne and the increase of solution concentration allowed obtaining monohydrosilylated products (**3ai–ak, 3bi**) with quantitative yields (Table 1, entries 29, 31–33). The excess of diynes was easily removed by flash chromatography.

Our preliminary tests of hydrosilylation of 1,4-diphenylbuta-1,3-diyne with **IC-POSS** (under conditions conducive to polymerization) confirmed the formation of oligomers (degree of polymerization of ca. 10). Synthesis of longer-chain polymers and cross-linked systems probably will be the real challenge due to the high steric hindrance of both diynes and **IC-POSS**s **1a** and **1b**. Based on our experience with the scope of Pt-catalysts and reagents, which we have tested so far, we believe that for the linear dialkylbuta-1,3-diynes, higher-molecular-weight oligomeres can be obtained than that for diphenylbuta-1,3-diyne, while for the diynes with bulky/more steric groups, e.g., *t*-Bu, even dimerization should not be observed. However, the use of different methods and reagents can lead to different results and conclusions. In the approach presented in this manuscript, the excess of buta-1,3-diyne favors the formation of monoadducts, and no oligomerization is observed. It should be noticed that the 4-fold excess of diyne leads to the selective formation of product **3**.

The last group of tested compounds was nonsymmetrically substituted 1,3-diynes (tri(*iso*-propyl)(4-phenylbuta-1,3-diyn-1-yl)silane (**2l**), tri(*iso*-propyl)((4-bromophenyl)buta-1,3-diyn-1-yl)silane (**2m**), and tri(*iso*-propyl)((4-(trifluoromethyl)phenyl)buta-1,3-diyn-1-yl)silane (**2n**)). It was found that the presence of silyl groups directed the SiH addition to the C≡C bond without the presence of a silicon atom, which highly improved the selectivity of the process. Therefore, an equimolar amount of diynes was applied to obtained products (**3al–an**) with very high yields. A similar influence of the silyl group on the addition of the SiH group to the C≡C in terminal and internal alkynes was previously reported.^64,71,72^

The above-described straightforward and efficient synthetic protocols allowed for the preparation of tripodal **IC-POSS**s with three alkenyl substituents bearing at the same time functional groups such as 4-bromophenyl, 4-fluorophenyl, thienyl, silyl, or blocked OH. These systems are considered to be the perfect components for further modification by hydrosilylation, hydroboration, and other chemical processes. They represent a new family of trifunctional **IC-POSS**s, which cannot be obtained directly by other synthetic methods.

The thermal properties of the majority of obtained products were characterized by the differential scanning calorimetry (DSC) and thermogravimetric analysis (TGA) (performed under an inert atmosphere). The results of DSC analysis carried out in the range of −50–100 °C showed that for all tested **IC-POSS**s no transitions are observed under the tested conditions—all of them appear as viscous liquids.

On the other hand, TGA analysis showed that, in general, silsesquioxanes **3** are thermally stable up to 300 °C (Table 2). The highest thermal stability was observed for the products of hydrosilylation of tri(*iso*-propyl)silylacetylene (**2b**) with silsesquioxane **1b** (**3bb**, 339 °C) and bis(4-bromophenyl)acetylene (**2e**) with silsesquioxane **1a** (**3ae**, 355 °C). On the other hand, hydrosilylation of 1,4-diphenylbuta-1,3-diyne (**2i**) with **1b** gave the product stability up to 337 °C.

**Table 2:**
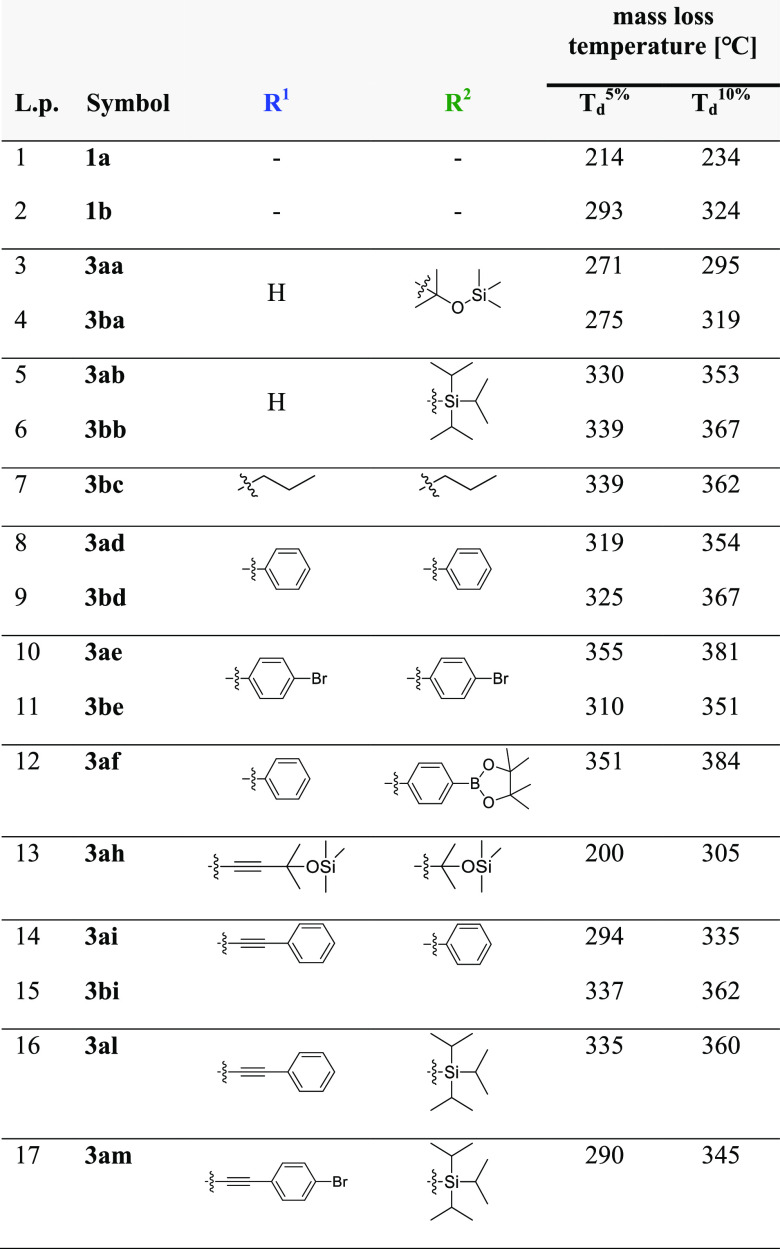
Thermal Properties of Selected IC-POSSs^a^

a Conditions: N_2_ atmosphere (20 mL/min); 29–995 °C at a heating rate of 10 °C/min.

**Figure 1:**
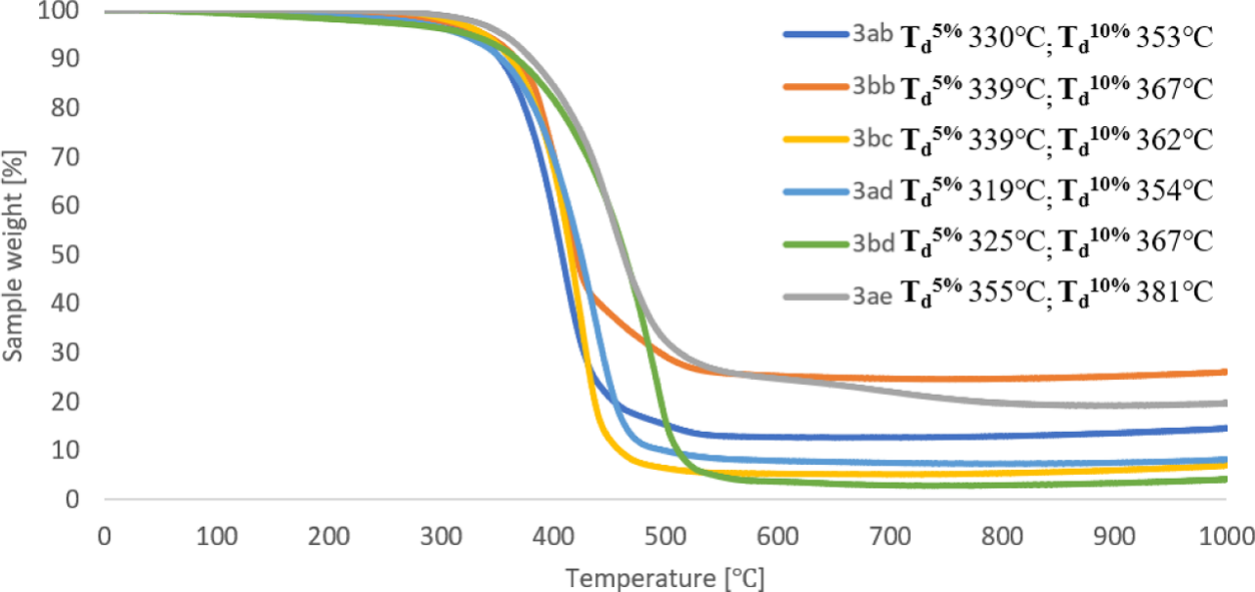
TGA curves for compounds **3ab**, **3bb**, **3bc**, **3ad**, **3bd**, and **3ae** obtained via hydrosilylation of alkynes with **IC-POSS**.

**Figure 2:**
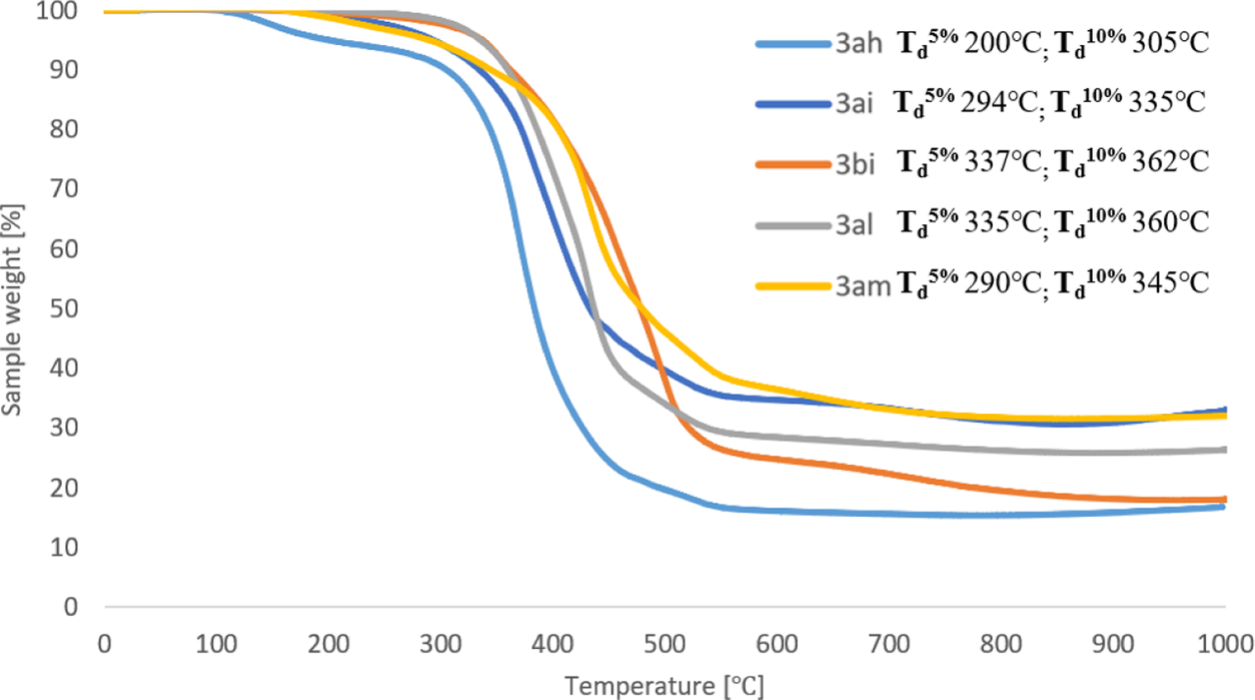
TGA curves for compounds **3ah**, **3ai**, **3bi**, **3al**, and **3am** obtained via hydrosilylation of 1,4-butadiynes with **IC-POSS**.

The lowest thermal stability was observed for the compounds containing blocked hydroxyl groups (OSiMe_3_). Data from TGA analysis is summarized in Table 2, while selected TGA curves are presented in Figures 1 and 2. The curves for the remaining tested compounds are presented in the Supporting Information.

## Conclusions

In this study, we presented for the first time the examination of hydrosilylation of the terminal and internal alkynes as well as symmetrically and nonsymmetrically 1,4-disubstituted buta-1,3-diynes with silsesquioxanes (HSiMe_2_O)_3_R′_7_Si_7_O_9_ (R = *i-*C_4_H_9_ (**1a**) and (H_3_C)_3_CH_2_C(H_3_C)HCH_2_C (**1b**)). The application of commercially available platinum catalysts, air-stable reagents, and the 100% atom economic efficiency of the hydrosilylation process proved that the developed methods are extremely efficient and lead to the alkenyl-functionalized tripodal **IC-POSS**s that cannot be obtained by other direct catalytic and noncatalytic reactions.

We successfully synthesized 20 novel products that possess both unsaturated double or/and triple bonds and other highly reactive organic substituents in their structures, e.g., OSiMe_3_, SiR_3_, Br, F, B(O(C(CH_3_)_2_)_2_ (Bpin)), and thienyl. The possibility of introducing 3, 6, or even more reactive functional groups into the POSS molecules in the presence of seven inert substituents makes the obtained compounds the novel class of sophisticated, nanometric building blocks, which have never been synthesized before. Herein, we have presented ideal examples of functional molecules that could be further modified and used in the preparation of advanced molecules with desired physicochemical properties. The products have been fully characterized by ^1^H, ^13^C, ^29^Si NMR, FT-IR, and high-resolution mass spectrometry (HRMS), as well as DSC and TGA analysis. The DSC results showed that no transitions are observed. On the other hand, the TGA proved the high thermal stability of alkenyl-functionalized **IC-POSS**s up to 300 °C.

## Experimental Section

Silsesquioxanes **1a,b** were synthesized according to previously reported methods.^33,61^ Buta-1,3-diynes **2g**–**h** and **2j–k** were synthesized by Glaser homocoupling of terminal alkynes—3,3-dimethyl-1-butyne, **2a**, and 1-ethynyl-4-fluorobenzene, 3-ethynylthiophene, respectively.^69^ Buta-1,3-diynes **2l–n** were synthesized by the Cadiot–Chodkiewicz cross-coupling reaction.^73^

### General Procedure for Hydrosilylation of Alkynes **2a**–**f** and 1,3-Diynes (**2g**–**n**) with IC-POSSs **1a,b** in the Presence of Karstedt’s Catalyst or Pt(PPh_3_)_4_

Karstedt’s catalyst (**I**) or Pt(PPh_3_)_4_ (**III**) was added to a solution of silsesquioxane **1a,b** (0.1 g, 0.103 mmol (**1a**), 0.073 mmol (**1b**)), and an appropriate alkyne or buta-1,3-diyne (0.219–1.236 mmol) in toluene in an amount that varied from 3 × 10^–4^ to 6 × 10^–2^ mol of Pt, depending on the experiment. Subsequently, the reaction mixture was heated to 100 °C. The conversion of the reagents was determined by ^1^H NMR spectroscopy after 24 and 48 h. Then, the solvent was evaporated under a vacuum. The crude product was dissolved in petroleum ether and filtered through silica gel or silica gel modified by HMDS for compounds **3aa**, **3af**/**4af**, **3ba**/**4ba**, **6ah**. After the evaporation of the solvents, the product was washed with methanol and dried for 6 h under a vacuum. The excess of **2i–k** was separated from products **3ai–3ak** and **3bi** using flash column chromatography in hexane/ethyl acetate. The isolated products were characterized by NMR, FT-IR spectroscopy, and MALDI TOF spectrometry.

For detailed data, see the Electronic Supporting Information.

### General Procedure for Hydrosilylation of Alkynes **2a** with **IC-POSSs1a** in the Presence of PtO_2_/XPhos System

The reaction was carried out in an argon atmosphere. PtO_2_ (**II**) (10 mol %) and 2-dicyclohexylphosphino-2′,4′,6′-tri(*iso*-propyl)biphenyl (20 mol %; XPhos) were added to a Schlenk flask with a Rotaflo stopcock and equipped with a magnetic stirrer. The catalyst and XPhos were dried under vacuum conditions for 1 h. Then, the flask was flushed quickly with argon, and anhydrous and degassed THF (1 mL) were added. The mixture was stirred at 60 °C for 30 min until a homogeneous system was obtained. After this, silsesquioxane **1a** (0.1 g, 0.103 mmol), an alkyne **2a** (60 μL, 0.310 mmol), and THF (1 mL) were added. The reaction was carried out at 100 °C. The conversion of the reagents was determined by ^1^H NMR spectroscopy after 24 and 48 h. The procedures of isolation and analysis of the obtained products were carried out as described above.

## Supporting Information Available

Materials, NMR spectra, IR spectra, and MALDI TOF MS spectra (PDF)
