# Supplementary material for: Alkenyl-Functionalized Open-Cage Silsesquioxanes (RSiMe2O)3R′7Si7O9: A Novel Class of Building Nanoblocks
Source: Inorg Chem. 2021 Jun 16;60(15):11006–13. doi: 10.1021/acs.inorgchem.1c00689 (PMC8335724; doi:10.1021/acs.inorgchem.1c00689)
Supplement: Supplementary file 1 — ic1c00689_si_001.pdf [file ic1c00689_si_001.pdf]

Alkenyl-functionalized open-cage silsesquioxanes $(RSiMe_2O)_3R'_7Si_7O_9$ – a novel class of building nanoblocks

Kinga Stefanowska[†,‡], *Jakub Szyling*[†,‡], *Jędrzej Walkowiak*[†], *Adrian Franczyk*[*,†]

[†]Center for Advanced Technology, Adam Mickiewicz University in Poznań, Uniwersytetu Poznańskiego 10, 61-614 Poznań, Poland.
[‡]Faculty of Chemistry, Adam Mickiewicz University in Poznań, Uniwersytetu Poznańskiego 8, 61-614 Poznań, Poland.

Table of Contents

1. Materials........ 2

2. Characterization of analytical methods........ 2

2.1 NMR analysis........ 2

2.2 Matrix-assisted ultraviolet laser desorption/ionization time-of-flight mass spectroscopy (MALDI-TOF-MS)........ 2

2.3 FT-IR analysis........ 2

2.4 Thermogravimetric analysis (TGA)........ 2

2.5 Differential scanning calorimetry (DSC)........ 3

2.6 Gel permeation chromatography (GPC)........ 3

4. Characterization of obtained products........ 4

1. Materials

1,3,5,7,9,11,14-heptaisobutyltricyclo[7.3.3.15,11]-heptasiloxane-endo-3,7,14-triol - TriSilanolisobutyl POSS® ((*i*-Bu)$_7$Si$_7$O$_9$(OH)$_3$, Hybrid Plastics), 1,3,5,7,9,11,14-heptaisooctyltricyclo[7.3.3.15,11]-heptasiloxane-endo-3,7,14-triol - TriSilanolisooctyl POSS® ((*i*-Oct)$_7$Si$_7$O$_9$(OH)$_3$, Hybrid Plastics), chlorodimethylsilane (98%, Sigma-Aldrich), [(1,1-dimethyl-2-propynyl)oxy]trimethylsilane (98%, Sigma-Aldrich), tri(isopropyl)silylacetylene (97%, Sigma-Aldrich), 3,3-dimethyl-1-butyne (98%, Sigma-Aldrich), 4-octyne (98%, Sigma-Aldrich), 1,2-diphenylacetylene (98%, Sigma-Aldrich), bis(4-bromophenyl)acetylene (98%, ABCR), 4-(phenylethynyl)phenylboronic acid pinacol ester (97%, Sigma-Aldrich), 1,4-diphenylbutadiyne (98%, Sigma-Aldrich), 3-ethynyltiophene (96%, Sigma-Aldrich), 1-ethynyl-4-fluorobenzene (99%, Sigma-Aldrich), phenylacetylene (98%, Sigma-Aldrich), 1-bromo-4-ethynylbenzene (98%, Sigma-Aldrich), magnesium sulfate (98%, Sigma-Aldrich), platinum(0)-1,3-divinyl-1,1,3,3-tetramethyldisiloxane (Karstedt's catalyst, solution in xylene, Pt 2%, Sigma-Aldrich), petroleum ether (pure, POCh), methanol (pure, POCh), chloroform-d$_1$ (99.96 atom% D, Sigma-Aldrich), *n*-butylamine (99.5%, Sigma-Aldrich), calcium hydride (95%, chunks, +4 mesh, reagent grade, Sigma-Aldrich), copper(I) chloride (≥90%, Sigma-Aldrich), piperidine (95.5%, Sigma-Aldrich), silica gel (high-purity grade, pore size ,60 Å, 60-100 mesh, Sigma-Aldrich), Celite (Sigma-Aldrich) were used as received. Toluene (CHROMSOLV PLUS, for HPLC, 99.9%, Aldrich) and THF (CHROMSOLV PLUS, for HPLC, ≥ 99.9%, inhibitor free, Aldrich) were used after purification by MBRAUN Solvent Purification Systems 500. Triethylamine (Et_3N, ≥ 99.5%, Sigma-Aldrich) was distilled from CaH_2 and stored in Schlenk's flask under argon atmosphere.

2. Characterization of analytical methods

2.1 NMR analysis

NMR spectra were recorded at 25°C on a Bruker Ultra Shield 300 MHz and Bruker Ascend 400 MHz NANOBAY spectrometers. $CDCl_3$ was used as a solvent and for internal deuterium lock. Chemical shifts are reported in ppm with reference to the residual solvent peaks for ^{1}H and ^{13}C NMR and to TMS for ^{29}Si NMR.

2.2 Matrix-assisted ultraviolet laser desorption/ionization time-of-flight mass spectroscopy (MALDI-TOF-MS)

MALDI-TOF mass spectra were recorded on a UltrafleXtreme mass spectrometer (Bruker Daltonics), equipped with a SmartBeam II laser (355 nm) in 500-4000 m/z range. 2,5-Dihydroxybenzoic acid (DHB, Bruker Daltonics, Bremen, Germany) served as matrix and was prepared in TA30 solvent (30:70 v/v acetonitrile: 0.1% TFA in water) at a concentration of 20 mg/mL. Studied samples were dissolved in dichloromethane (2 mg/mL) and then mixed in a ratio 1:1 v/v with matrix solution. Matrix/sample mixtures (1 μL) were spotted onto the MALDI target and dried in air. Mass spectra were measured in reflection mode. The data were analyzed using the software provided with the Ultraflex instrument - FlexAnalysis (version 3.4). Mass calibration (cubic calibration based on five to seven points) was performed using external standards (Peptide Calibration Standard).

2.3 FT-IR analysis

Fourier Transform-Infrared (FT-IR) spectra were recorded on a Bruker Tensor 27 Fourier transform spectrophotometer equipped with a SPECAC Golden Gate, diamond ATR unit, with ensuring a resolution of 2cm^{-1}.

2.4 Thermogravimetric analysis (TGA)

Thermogravimetric Analyses (TGA) were performed using a Netzsch TG 209 Libra thermal gravimetric analyzer. The measurements were conducted under nitrogen (flow of 20 mL/min), from 29°C to 995°C at the heating rate of 10°C/min. The temperature of initial degradation ($T_{5\%}$) was taken as the onset temperature at which 5 wt% of mass loss occurs.

2.5 Differential scanning calorimetry (DSC)

Differential Scanning Calorimetry (DSC) analysis was carried out with the use of a DSC 209 F1 Phoenix (Netzsch). The measurements were conducted in the range of -50 ℃ to 100℃ with a heating rate of 10℃/min. in nitrogen (25 mL $min.^{-1}$). The results after the second cycle were analyzed.

2.6 Gel permeation chromatography (GPC)

GPC analyses were performed using Agilent 1260 Infinity system equipped with RI detector and Phenogel 10um Linear(2) 300x7.8 mm column. THF was used as a mobile phase in a flow rate of 1 mL·min−1. Temperatures of RI detector and column were set at 35ºC. Time of analysis was 17 min. Molecular weights (number average, Mn; weight average, Mw) and polydispersity index (Mw/Mn, PDI) were calculated based on the calibration curve using polystyrene standards (Shodex) in a range of 1000 - 3 500 000, using Agilent Software GPC/SEC – 1260 GPC set.

4. Characterization of obtained products

1a

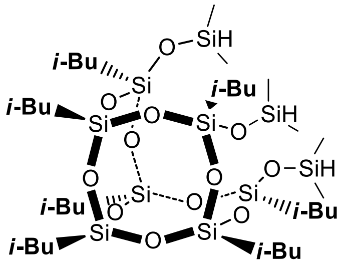

1a

Chemical Formula: $C_{34}H_{84}O_{12}Si_{10}$
Molecular Weight: 965.88

Isolated yield = 93%, colorless oil.

^{1}H NMR ($CDCl_3$, 300 MHz, δ, ppm): 0.22 (s, 9H, $SiCH_3$), 0.23 (s, 9H, $SiCH_3$), 0.54-0.58 (m, 14H, CH_2), 0.94-0.98 (m, 42H, CH_3), 1.79-1.89 (m, 7H, CH), 4.73-4.76 (m, 3H, SiH). **^{13}C NMR** ($CDCl_3$, 75 MHz, δ, ppm): 0.80 ($SiCH_3$), 22.60, 23.75, 24.04 ($CH_2CH(CH_3)_2$), 24.12, 24.22, 24.77 ($CH_2CH(CH_3)_2$), 25.78, 26.00, 26.03, 26.12 ($CH_2CH(CH_3)_2$). **^{29}Si NMR** ($CDCl_3$, 79 MHz, δ, ppm): -68.02, -67.68, -67.13 (SiO_3), -5.48 ($OSi(CH_3)_2$). **FT IR (cm^{-1}):** 2953.85, 2902.25, 2870.10, 2161.64, 2137.02, 1560.30, 1465.27, 1366.12, 1331.27, 1253.03, 1227.69, 1050.42, 901.29, 835.02, 739.77, 609.37, 440.21.

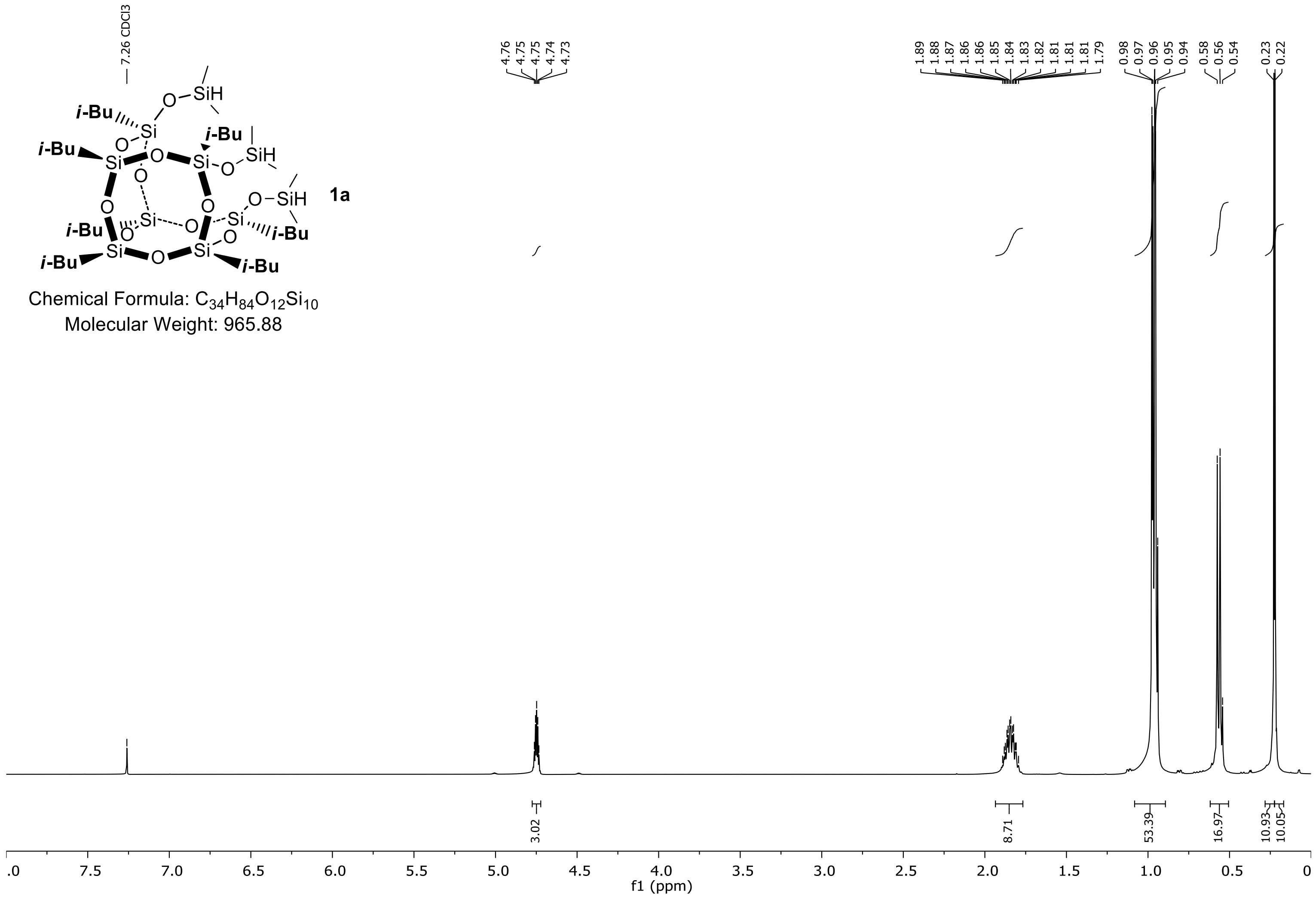

Figure S1. ^{1}H NMR of compound **1a**.

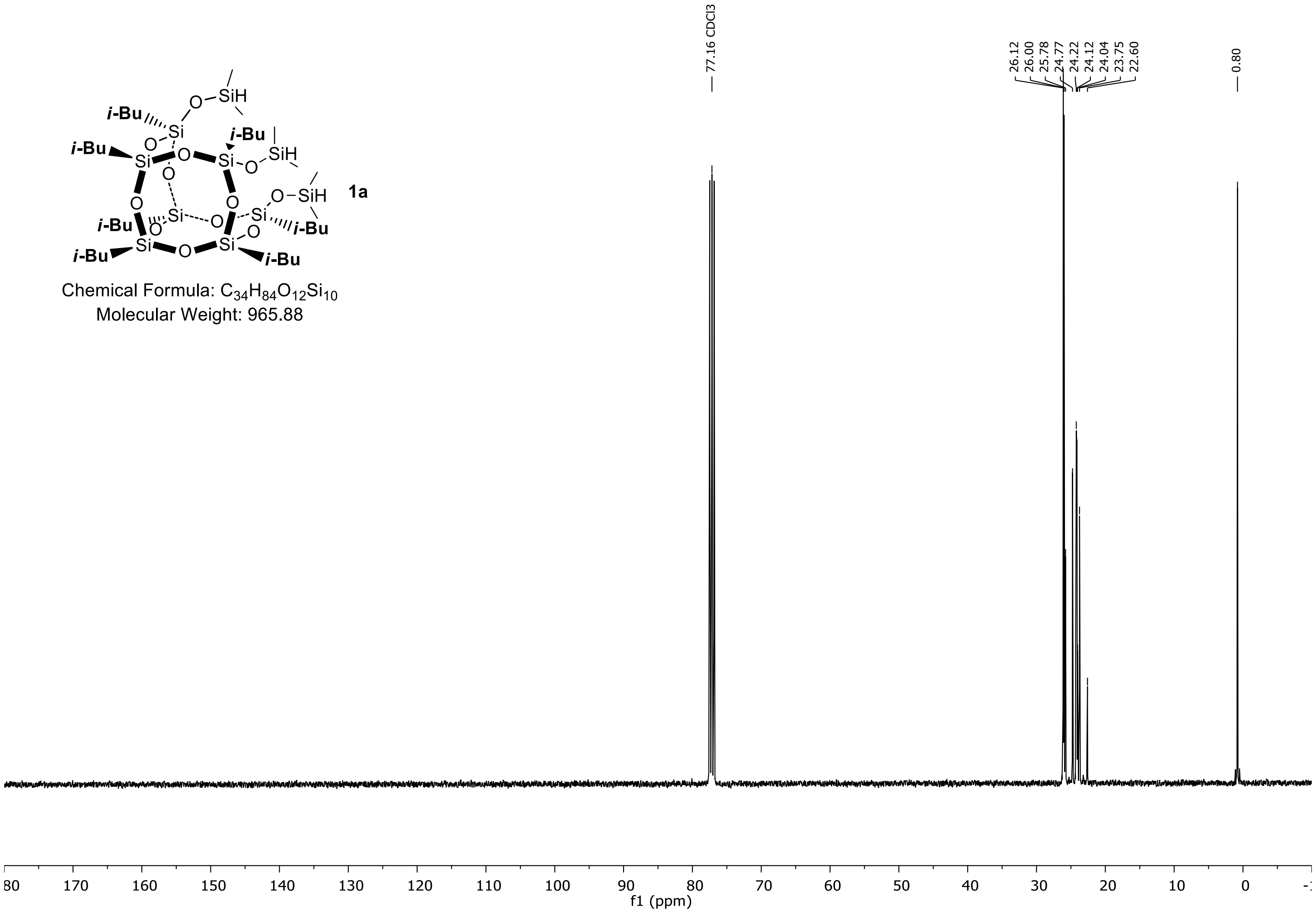

Figure S2. ^{13}C NMR of compound **1a**.

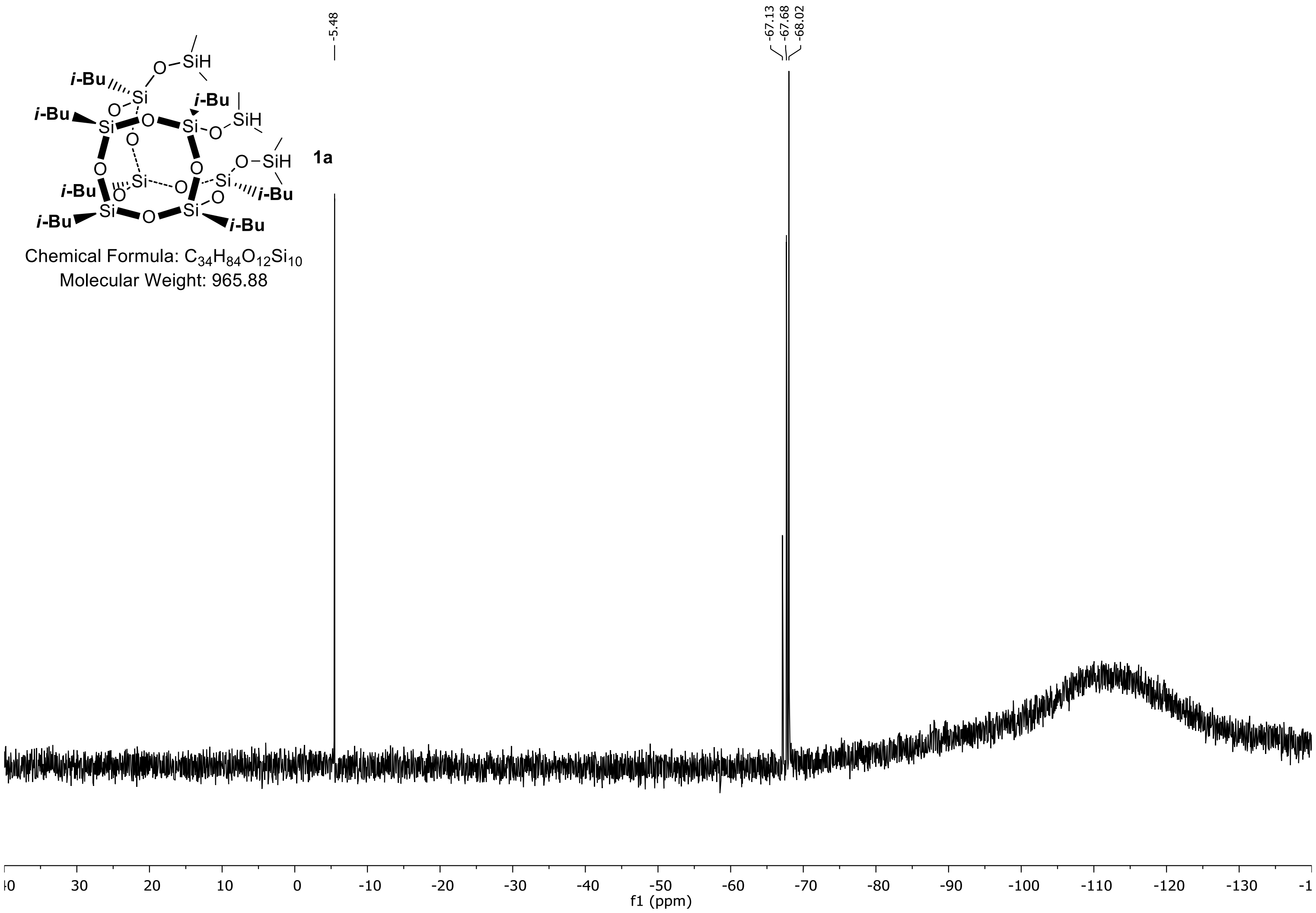

Figure S3. ^{29}Si NMR of compound **1a**.

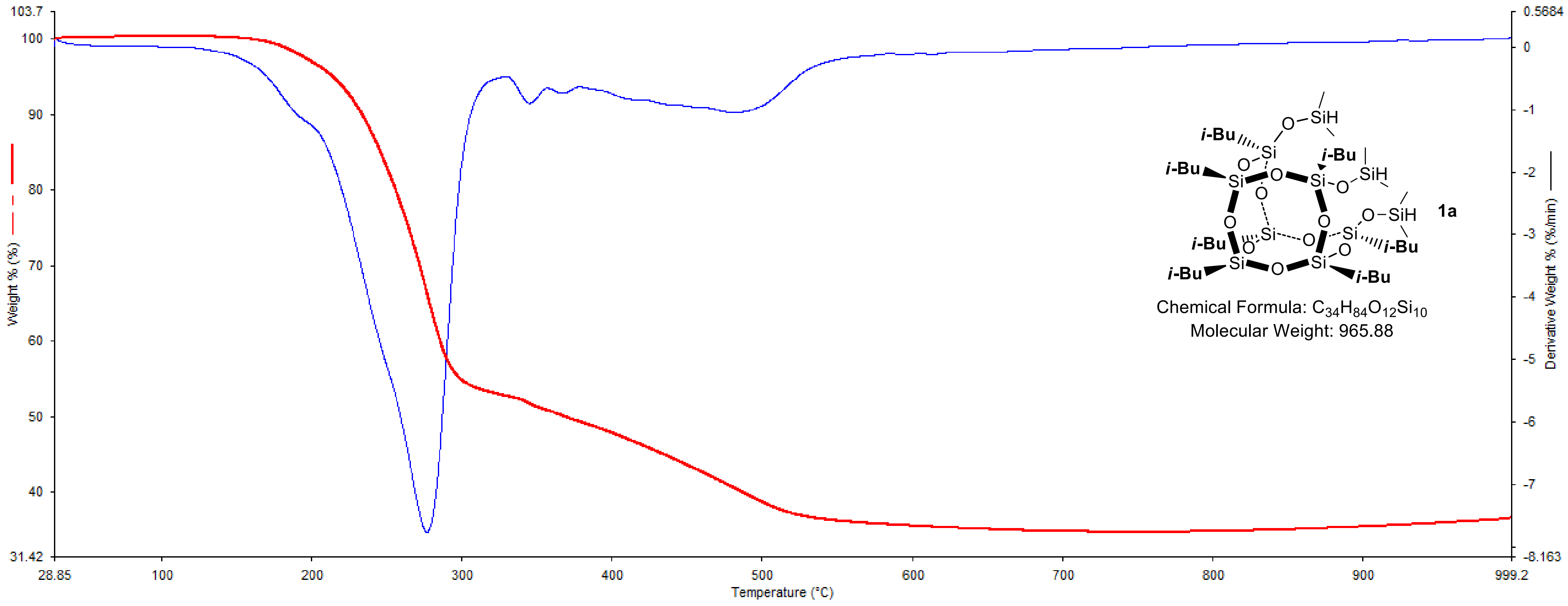

Figure S4. TGA/DTG curves of compound **1a**.

1b

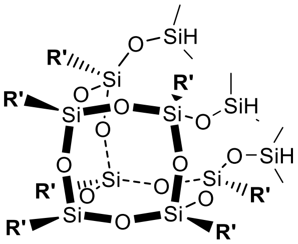

1b

R'= $CH_2CH(CH_3)CH_2C(CH_3)_3$

Chemical Formula:

$C_{36}H_{140}O_{12}Si_{10}$

Molecular Weight: 1358.65

Isolated yield = 90%, colorless oil.

^{1}H NMR ($CDCl_3$, 300 MHz, δ, ppm): 0.23 (s, 18H, $SiC\underline{H}_3$), 0.50-0.57 (m, 7H, $SiC\underline{H}_2$), 0.72-0.77 (m, 7H, $SiC\underline{H}_2$), 0.89-0.91 (m, 63H, $C(C\underline{H}_3)_3$), 0.98-1.02 (m, 21H, $CH(C\underline{H}_3)$), 1.14-1.18 (m, 7H, $C\underline{H}_2$), 1.25-1.30 (m, 7H, $C\underline{H}_2$), 1.81-1.84 (m, 7H, $C\underline{H}(CH_3)$), 4.75-4.80 (m, 3H, $Si\underline{H}$). **^{13}C NMR** ($CDCl_3$, 75 MHz, δ, ppm): 0.87 ($OSi\underline{C}H_3$), 23.56, 25.06, 25.10, 25.17, 25.39, 25.45, 25.47, 25.58, 25.73, 25.75, 26.09, 26.11 ($\underline{C}H$, $Si\underline{C}H_2$, $CH(\underline{C}H_3)$), 30.32, 30.41 ($(\underline{C}H_3)_3$), 31.33, 31.36 ($\underline{C}(CH_3)_3$), 54.48, 55.00 ($\underline{C}H_2C(CH_3)_3$). **^{29}Si NMR** ($CDCl_3$, 79 MHz, δ, ppm): -68.31, -68.23, -68,07, -67.94, -67.34 (SiO_3), -5.87, -5.79, -5.72 ($OSi(CH_3)_2$). **FT IR (cm^{-1}):** 2952.19, 2905.29, 2868.95, 2139.04, 1467.30, 1393.04, 1252.28, 1225.91, 1084.63, 1053.83, 902.06, 835.35, 768.65, 475.90, 429.05.

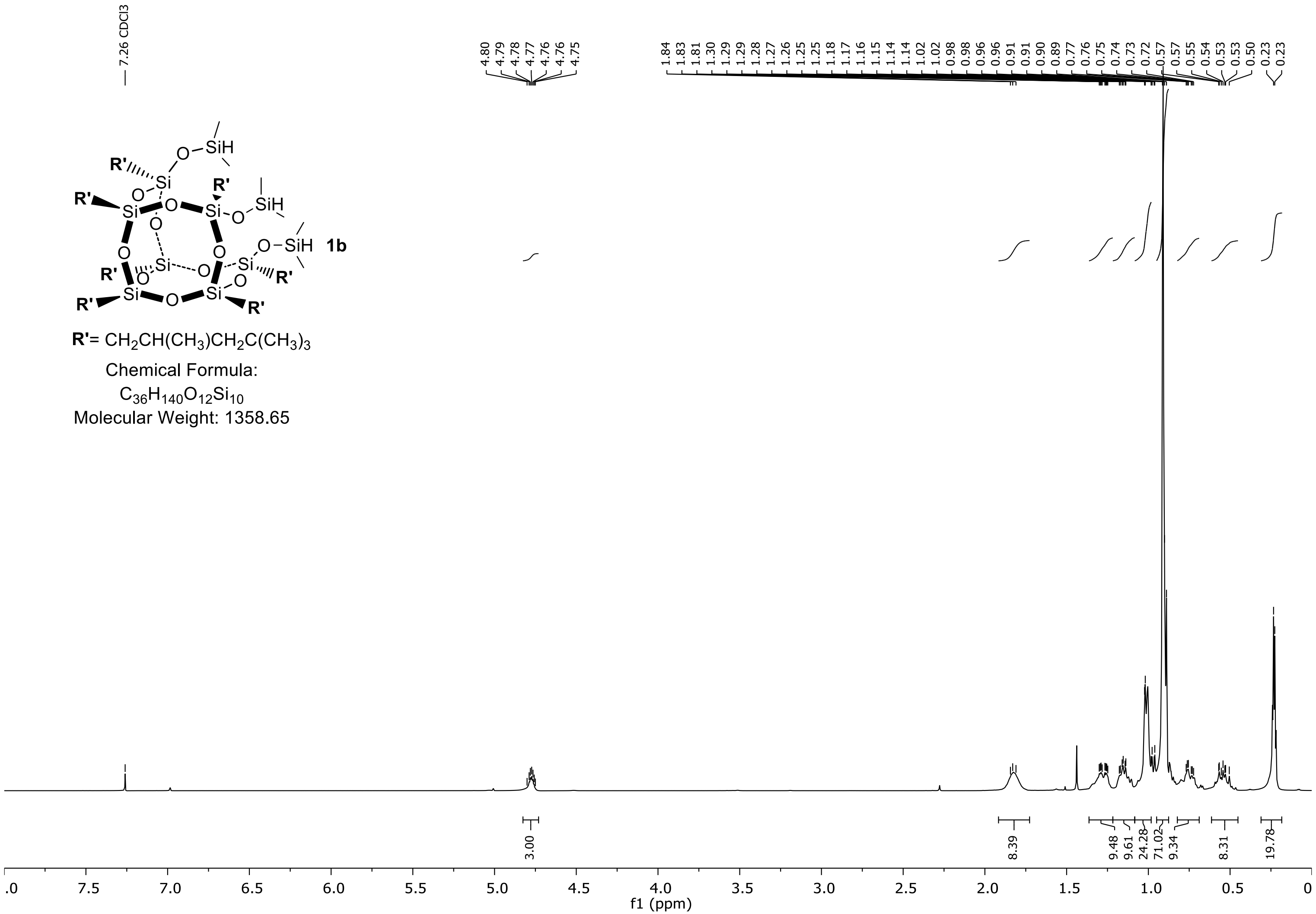

Figure S5. ^{1}H NMR of compound **1b**.

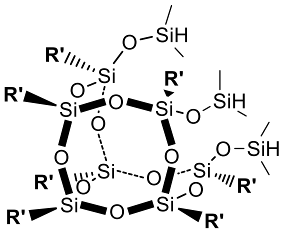

1b

R'= $CH_2CH(CH_3)CH_2C(CH_3)_3$

Chemical Formula:

$C_{36}H_{140}O_{12}Si_{10}$

Molecular Weight: 1358.65

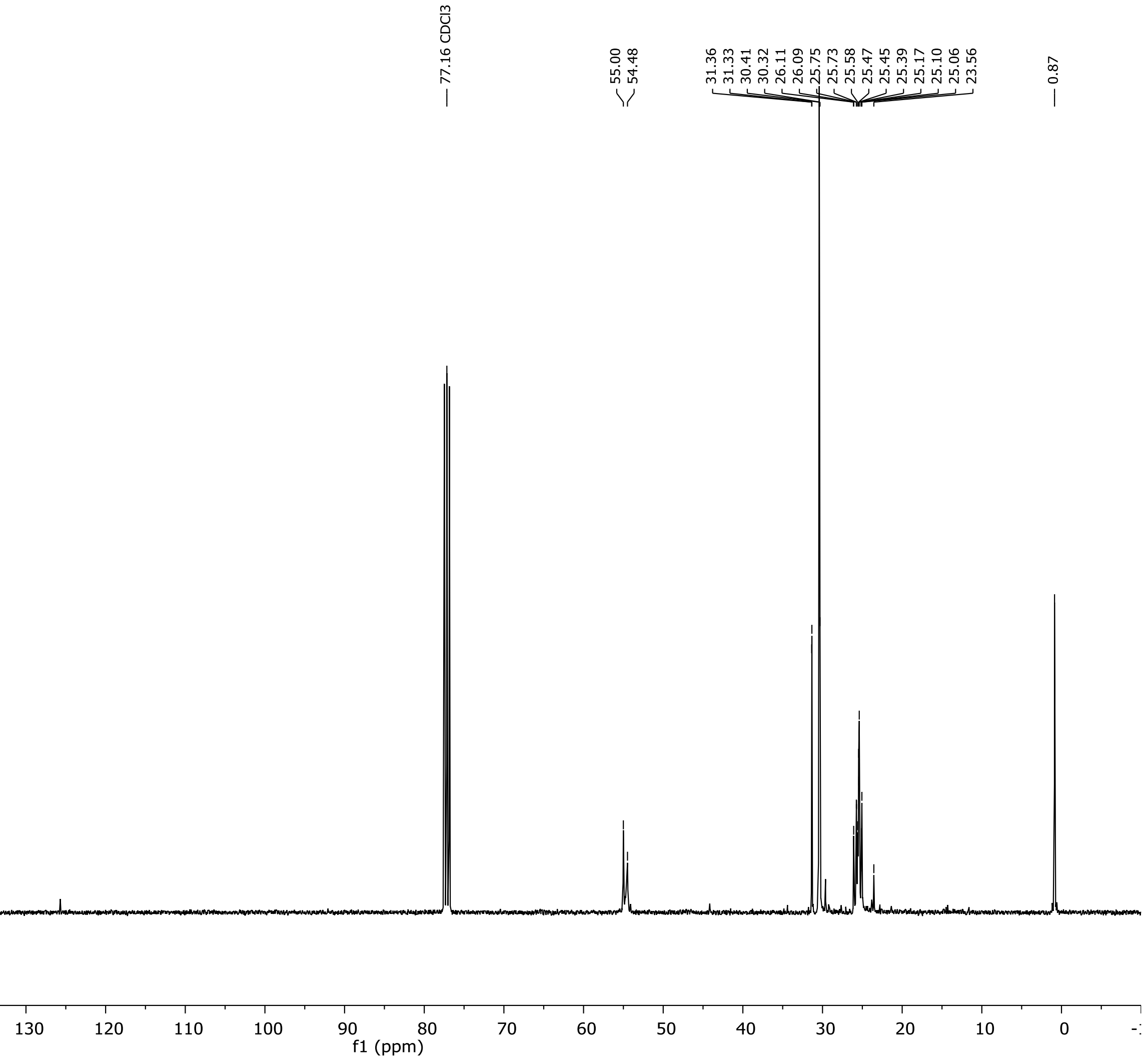

Figure S6. ^{13}C NMR of compound **1b**.

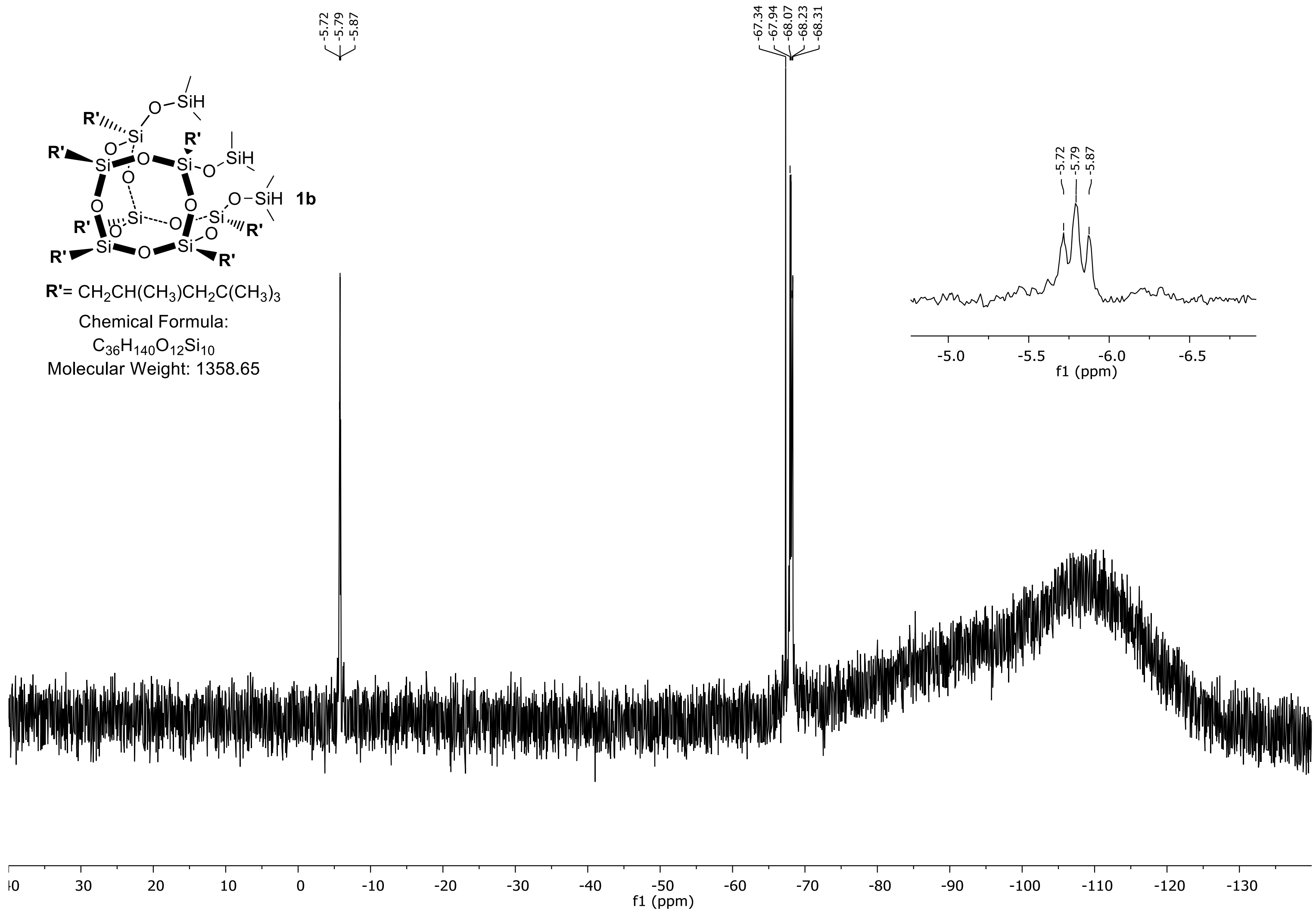

Figure S7. ^{29}Si NMR of compound **1b**.

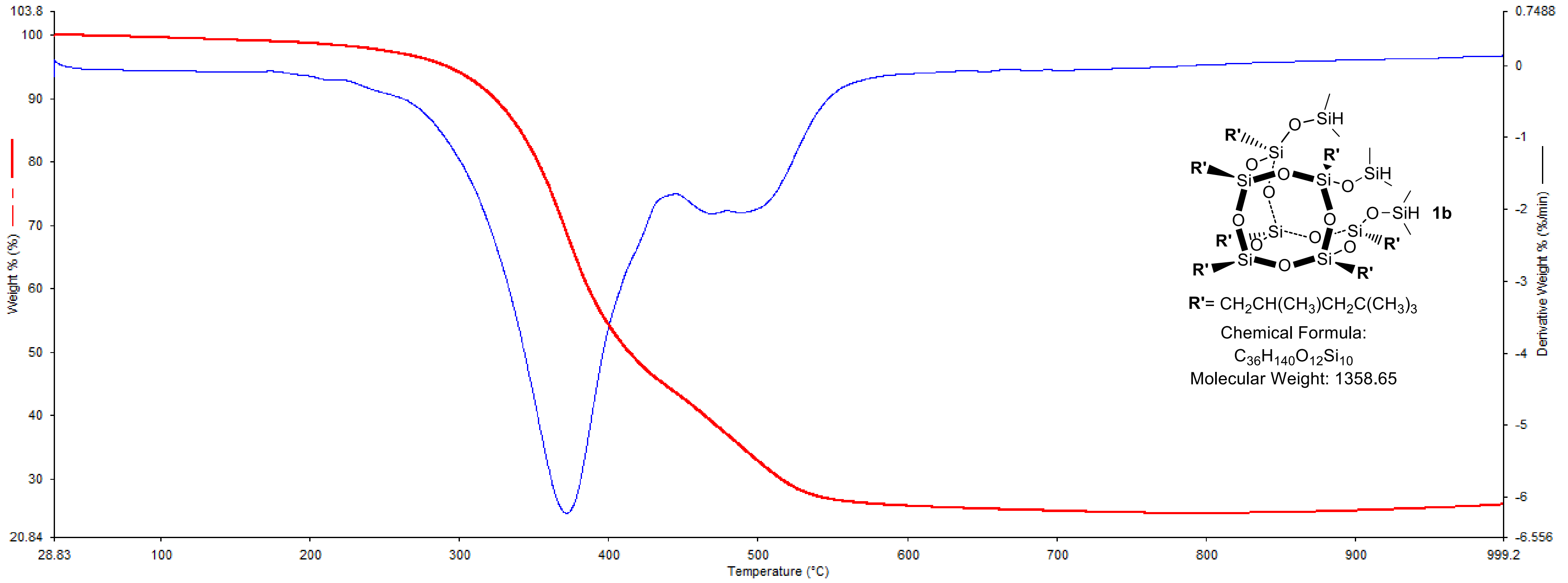

Figure S8. TGA/DTG curves of compound **1b**.

3aa/4aa = 97/3

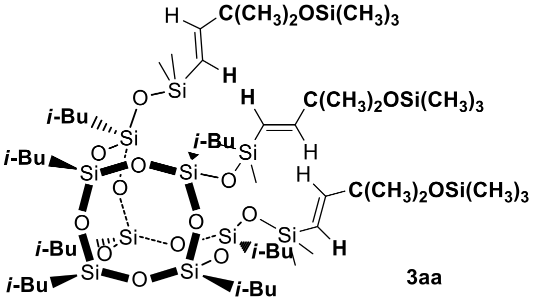

3aa

Chemical Formula: $C_{58}H_{132}O_{15}Si_{13}$
Molecular Weight: 1434.77

Isolated yield = 94%, colorless oil.

^{1}H NMR ($CDCl_3$, 300 MHz, δ, ppm): 0.10 (s, 27H, $C(CH_3)_2OSi(C\underline{H}_3)_3$), 0.21 (s, 18H, $SiC\underline{H}_3$), 0.55-0.58 (m, 14H, $C\underline{H}_2$), 0.95-0.97 (m, 42H, $C\underline{H}_3$), 1.28 (s, 18H, $C(C\underline{H}_3)_2OSi(CH_3)_3$), 1.82-1.87 (m, 7H, $C\underline{H}$), 5.54 (s, 2H, $=C\underline{H}_2$, product **4ab**), 5.73 (d, 3H, $=C(\underline{H})$, $J_{(H,H)}$= 18.97 Hz), 6.06 (s, 2H, $=C\underline{H}_2$, product **4ab**), 6.17 (d, 3H, $=C(\underline{H})$, $J_{(H,H)}$= 18.95 Hz), 6.33 (s, 2H, $=C\underline{H}_2$, product **4ab**). **^{13}C NMR** ($CDCl_3$, 75 MHz, δ, ppm): 0.88 ($Si\underline{C}H_3$), 2.75 ($OSi(\underline{C}H_3)_3$, 22.62, 23.91, 24.02 ($\underline{C}H_2CH(CH_3)_2$), 24.13, 24.19, 25.17 ($CH_2\underline{C}H(CH_3)_2$), 25.79, 26.05, 26.21 ($CH_2CH(\underline{C}H_3)_2$), 30.10 ($C(\underline{C}H_3)_2OSi(CH_3)_3$), 74.79 ($\underline{C}(CH_3)_2OSi(CH_3)_3$), 124.14 ($=\underline{C}Si(CH_3)_2O$), 154.81 ($=\underline{C}(H)C(CH_3)_2OSi(CH_3)_3$). **^{29}Si NMR** ($CDCl_3$, 79 MHz, δ, ppm): -68.03, -67.90, -67.24 (SiO_3), -1.69 ($OSi(CH_3)_2$), 9.68 ($OSi(CH_3)_3$). **FT IR (cm^{-1}):** 2953.5, 2902.2, 2869.4, 1465.2, 1401.6, 1365.7, 1250.5, 1227.3, 1074.4, 1033.1, 835.0,750.1, 444.6. **MALDI TOF MS -** (m/z) ([M+Na], (%)): 1457.64.

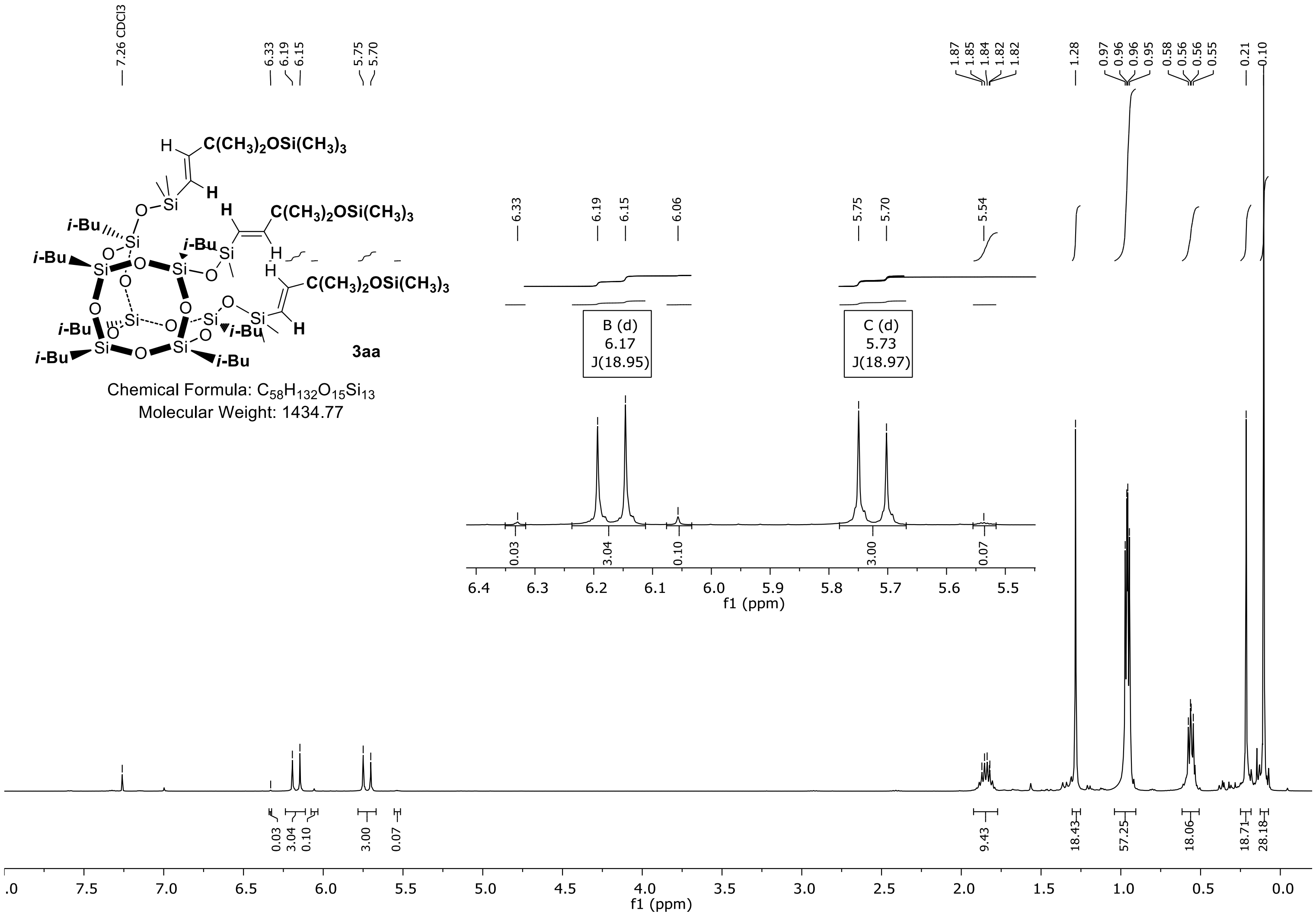

Figure S9. ^{1}H NMR of compound **3aa**.

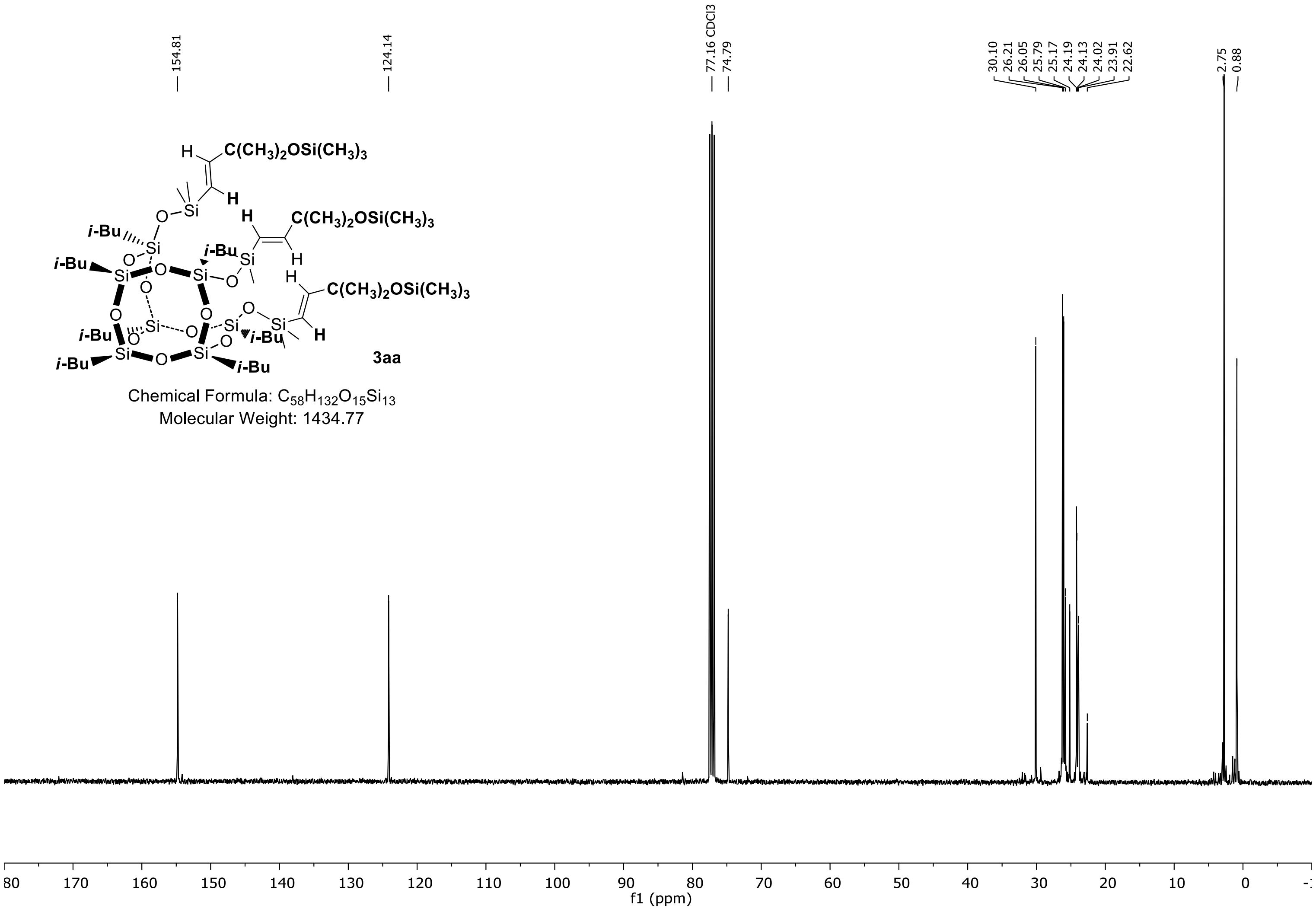

Figure S10. ^{13}C NMR of compound **3aa.**

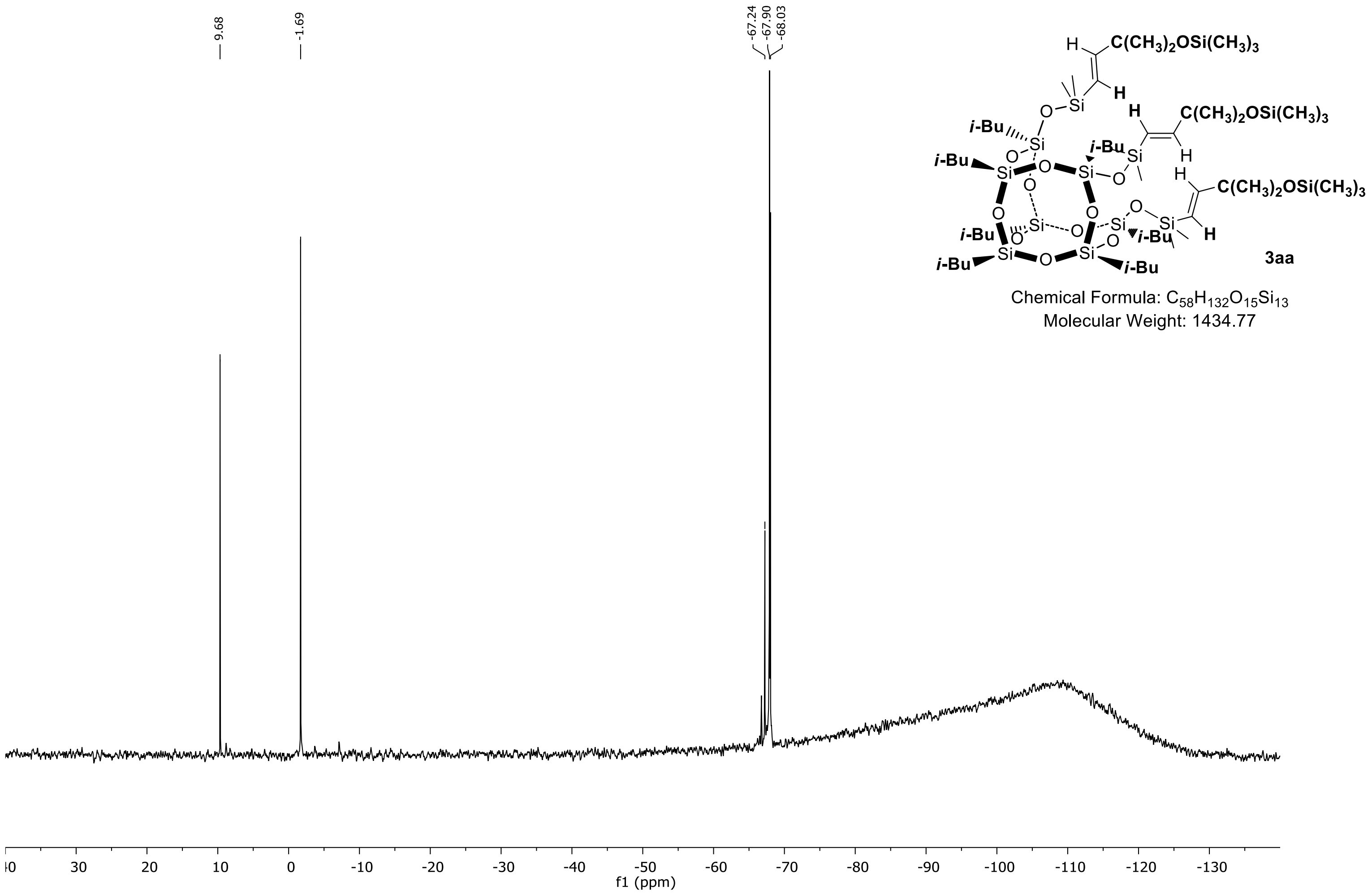

Figure S11. ^{29}Si NMR of compound **3aa**.

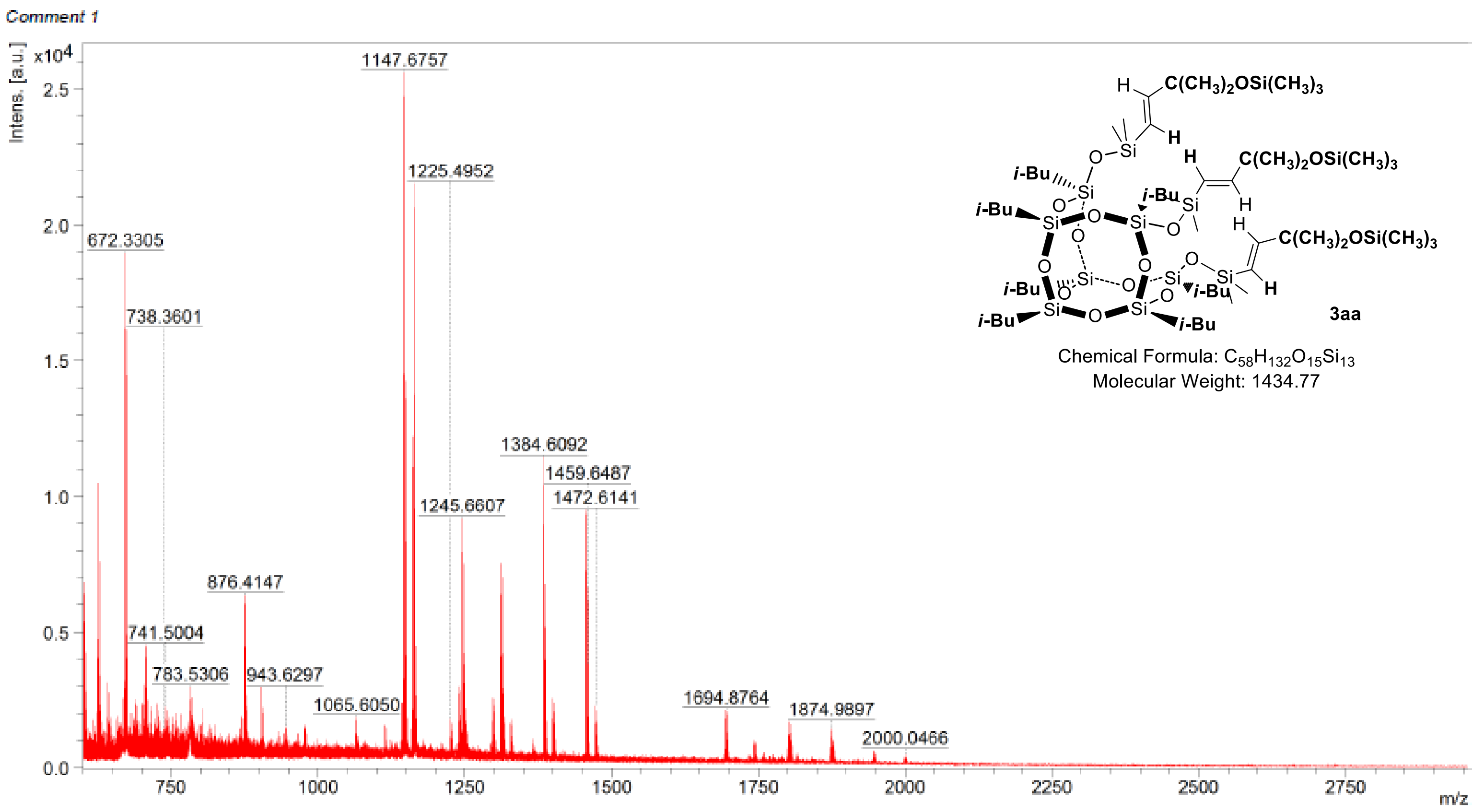

Figure S12. MALDI TOF MS spectra of compound **3aa**.

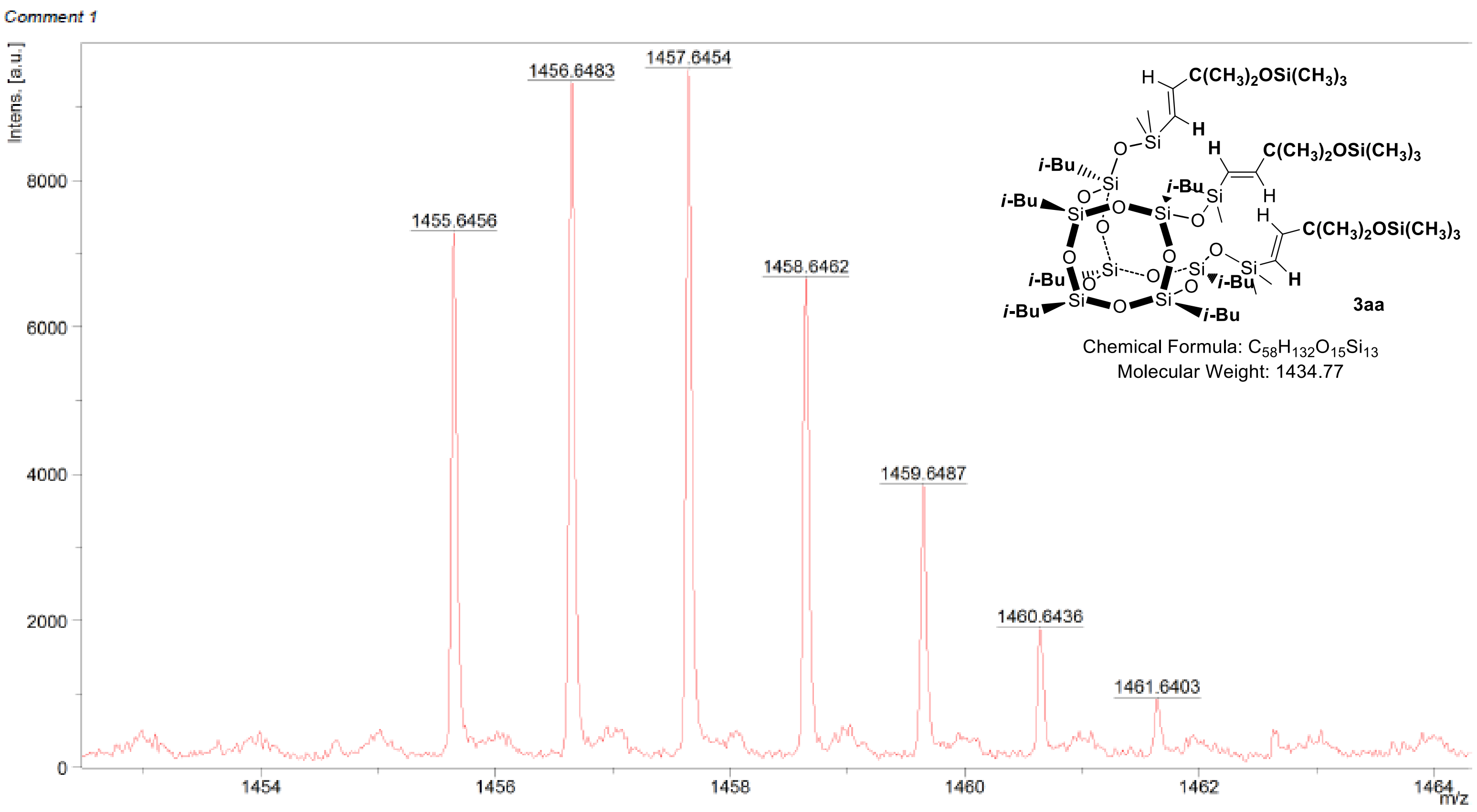

Figure S13. MALDI TOF MS spectra of compound **3aa**.

Molecular Weight Averages

Peak	Mp	Mn	Mw	Mz	Mz+1	Mv	PD
Peak 1	1570	1546	1725	1928	2161	1895	1.116

Peak information

	Start (mins)	End (mins)
Baseline region 1	3.03	9.38
Baseline region 2	15.71	15.88
Peak 1	10.72	11.98

Peak	Trace	Peak Max RT (mins)	Peak Area (mV.s)	Peak Height (mV)
Peak 1	RI	11.44	92212.898	4343.662
Peak 1	UV 250nm	11.20	846.707	-65.654

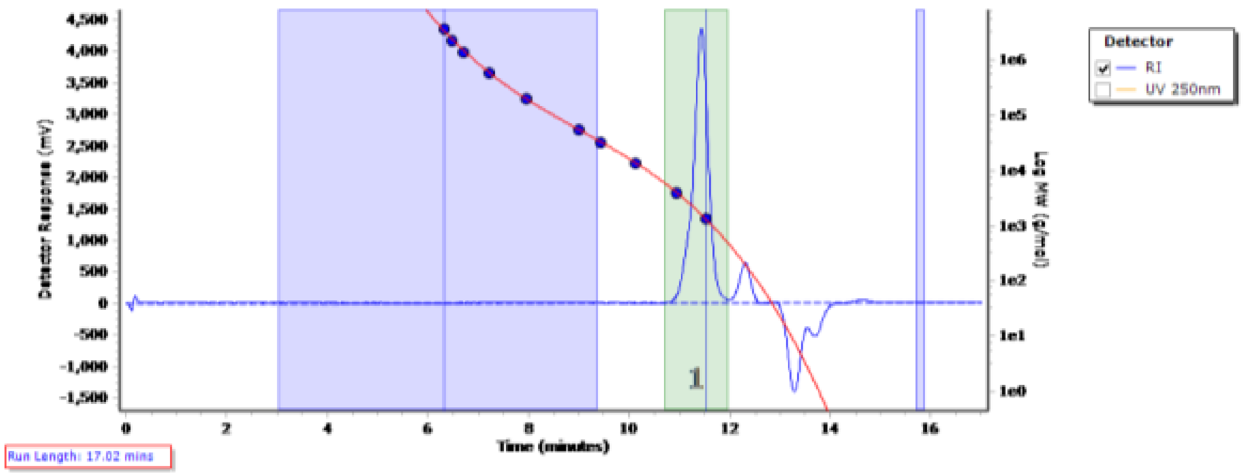

Figure S14. GPC chromatogram of compound **3aa**.

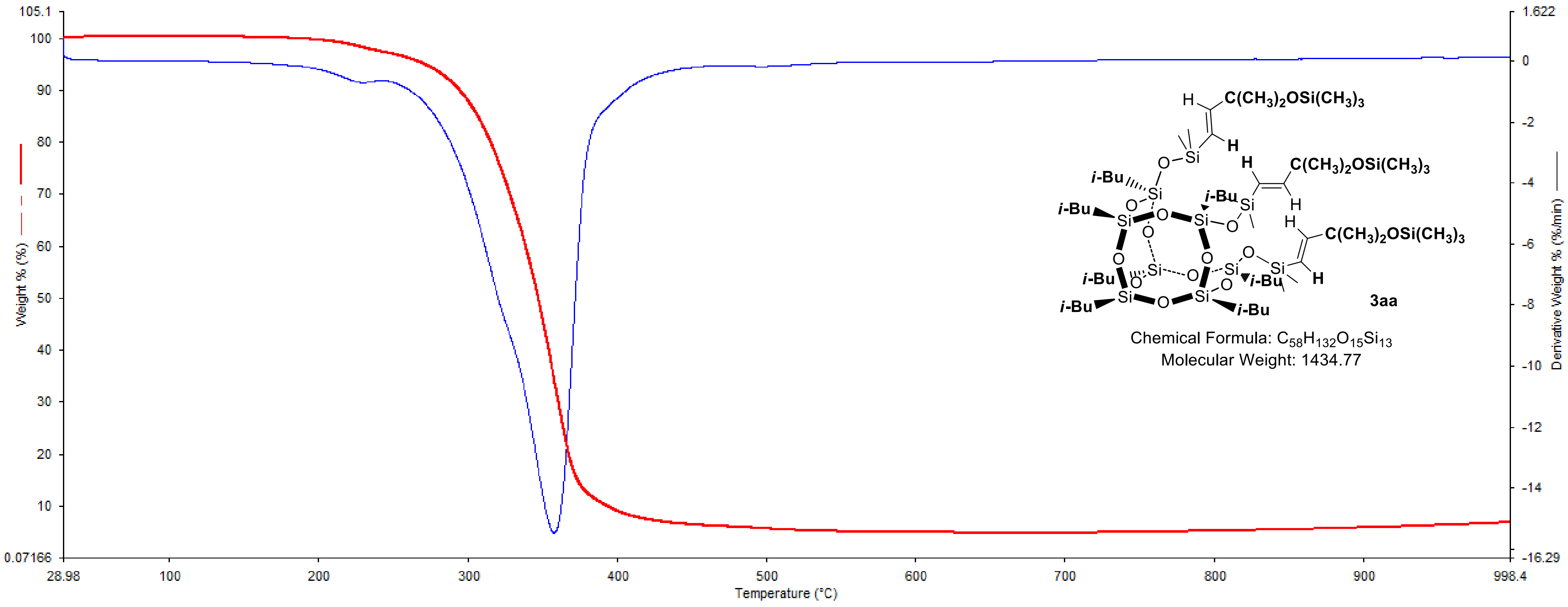

Figure S15. TGA/DTG curves of compound **3aa**.

3ba

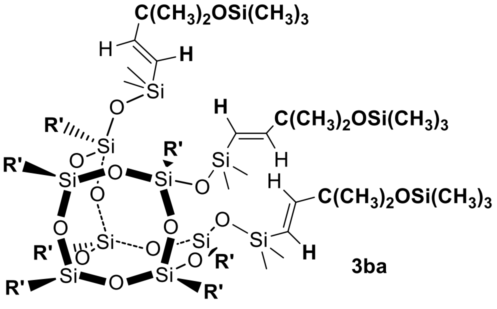

R'= $CH_2CH(CH_3)CH_2C(CH_3)_3$
Chemical Formula: $C_{60}H_{188}O_{15}Si_{13}$
Molecular Weight: 1827.52

Isolated yield = 94%, colorless oil.

^{1}H NMR ($CDCl_3$, 300 MHz, δ, ppm): 0.10 (s, 27H, $C(CH_3)_2OSi(C\underline{H}_3)_3$), 0.22 (s, 18H, $SiC\underline{H}_3$), 0.52-0.60 (m, 7H, $SiC\underline{H}_2$), 0.74-0.75 (m, 7H, $SiC\underline{H}_2$), 0.90-0.91 (m, 63H, $C(C\underline{H}_3)_3$), 1.00, 1.01, 1.14-1.18 (m, 75H, $Si(CH(C\underline{H}_3)_2)_3$, $CH(C\underline{H}_3)$), 1.23-1.26 (m, 23H, $C\underline{H}_2$, $Si(C\underline{H}(CH_3)_2)_3$), 1.28 (s, 18H, $C(C\underline{H}_3)_2OSi(CH_3)_3$, 1.82-1.84 (m, 7H, $C\underline{H}(CH_3)$), 5.73 (d, 3H, =C($\underline{H}$), $J_{(H,H)}$= 18.96 Hz), 6.16 (d, 3H, =C($\underline{H}$), $J_{(H,H)}$= 19.01 Hz). **^{13}C NMR** ($CDCl_3$, 75 MHz, δ, ppm): 1.03 ($OSi(\underline{C}H_3)_2$), 2.79 ($OSi(\underline{C}H_3)_3$), 25.45, 25.85 ($\underline{C}H$, $Si\underline{C}H_2$, $CH(\underline{C}H_3)$), 30.13 ($C(\underline{C}H_3)_2OSi(CH_3)_3$), 30.32, 30.46 ($(\underline{C}H_3)_3$), 31.34 ($\underline{C}(CH_3)_3$), 54.68 ($\underline{C}H_2C(CH_3)_3$), 74.80 ($\underline{C}(CH_3)_2OSi(CH_3)_3$), 124.22 (=$\underline{C}Si(CH_3)_2$), 154.78 (=$\underline{C}(H)C(CH_3)_2OSi(CH_3)_3$). **^{29}Si NMR** ($CDCl_3$, 79 MHz, δ, ppm): -68.23, -67.86, -67.47 (SiO_3), -1.77 ($OSi(CH_3)_2$), 9.60 ($OSi(CH_3)_3$). **FT IR (cm^{-1}):** 2952.91, 2904.85, 2869.17, 1467.07, 1393.20, 1376.63, 1363.82, 1250.08, 1223.25, 1080.38, 1035.84, 835.89, 786.50, 755.65, 475.89, 432.50.

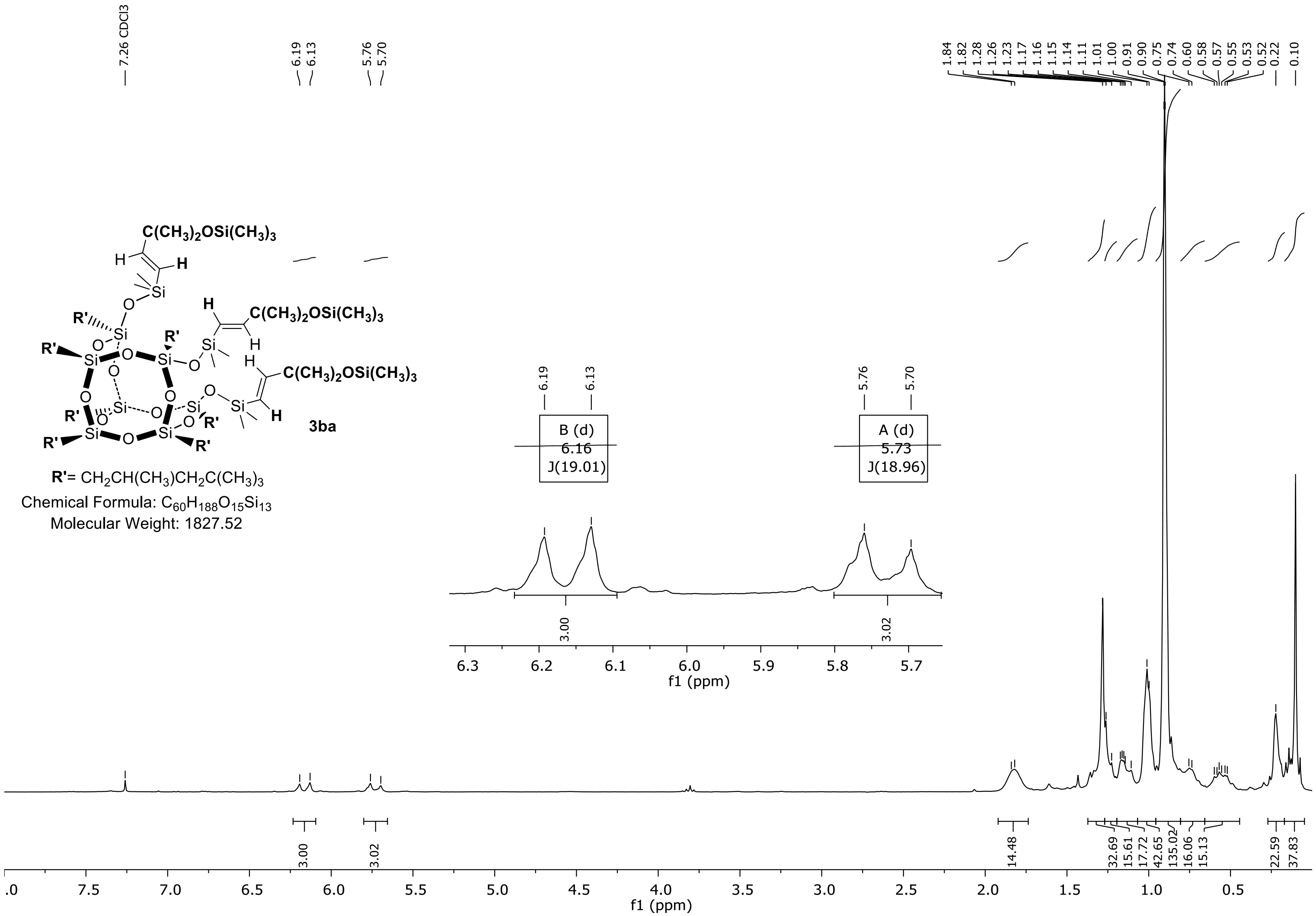

Figure S16. ^{1}H NMR of compound **3ba**.

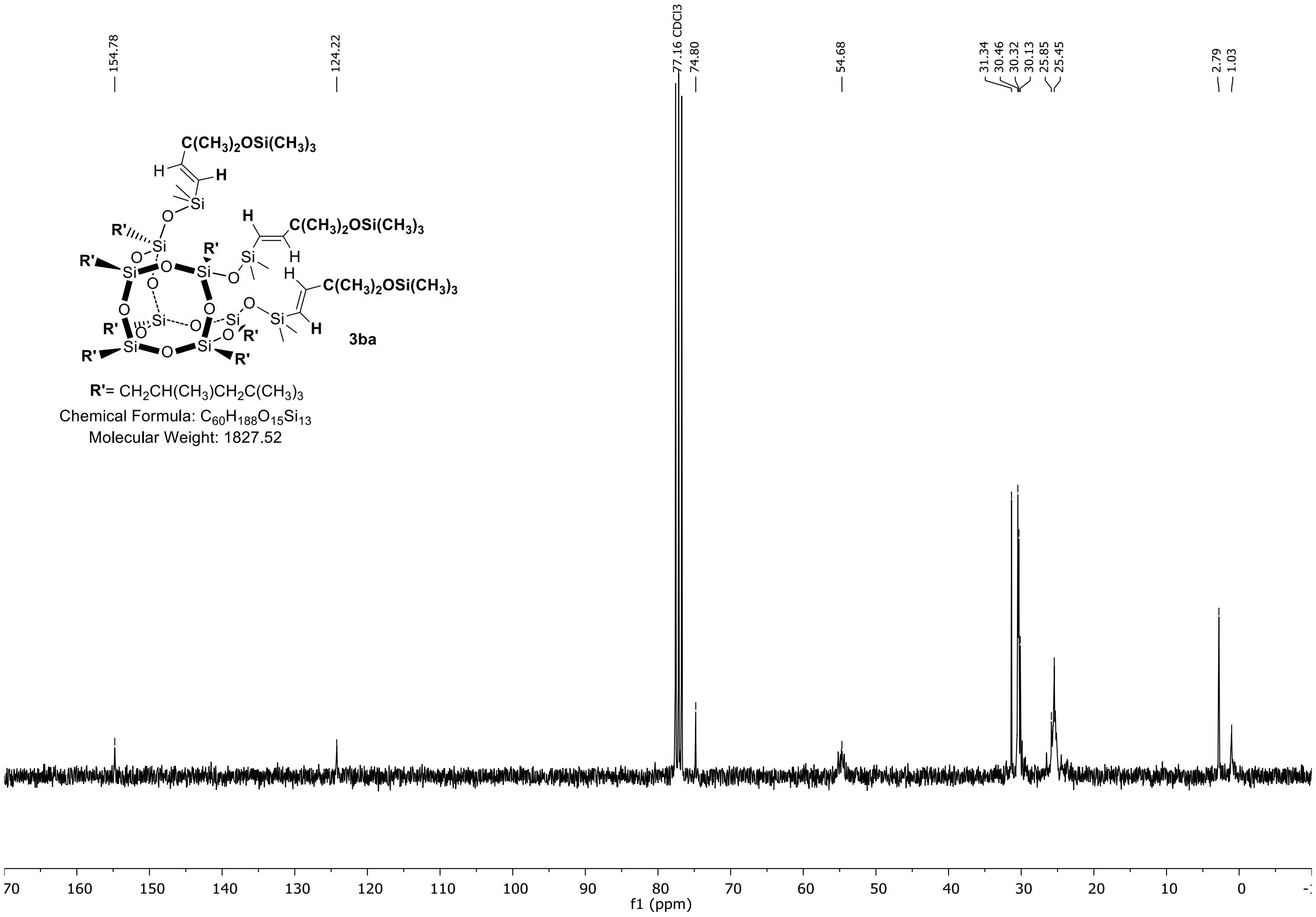

Figure S17. ^{13}C NMR of compound **3ba**.

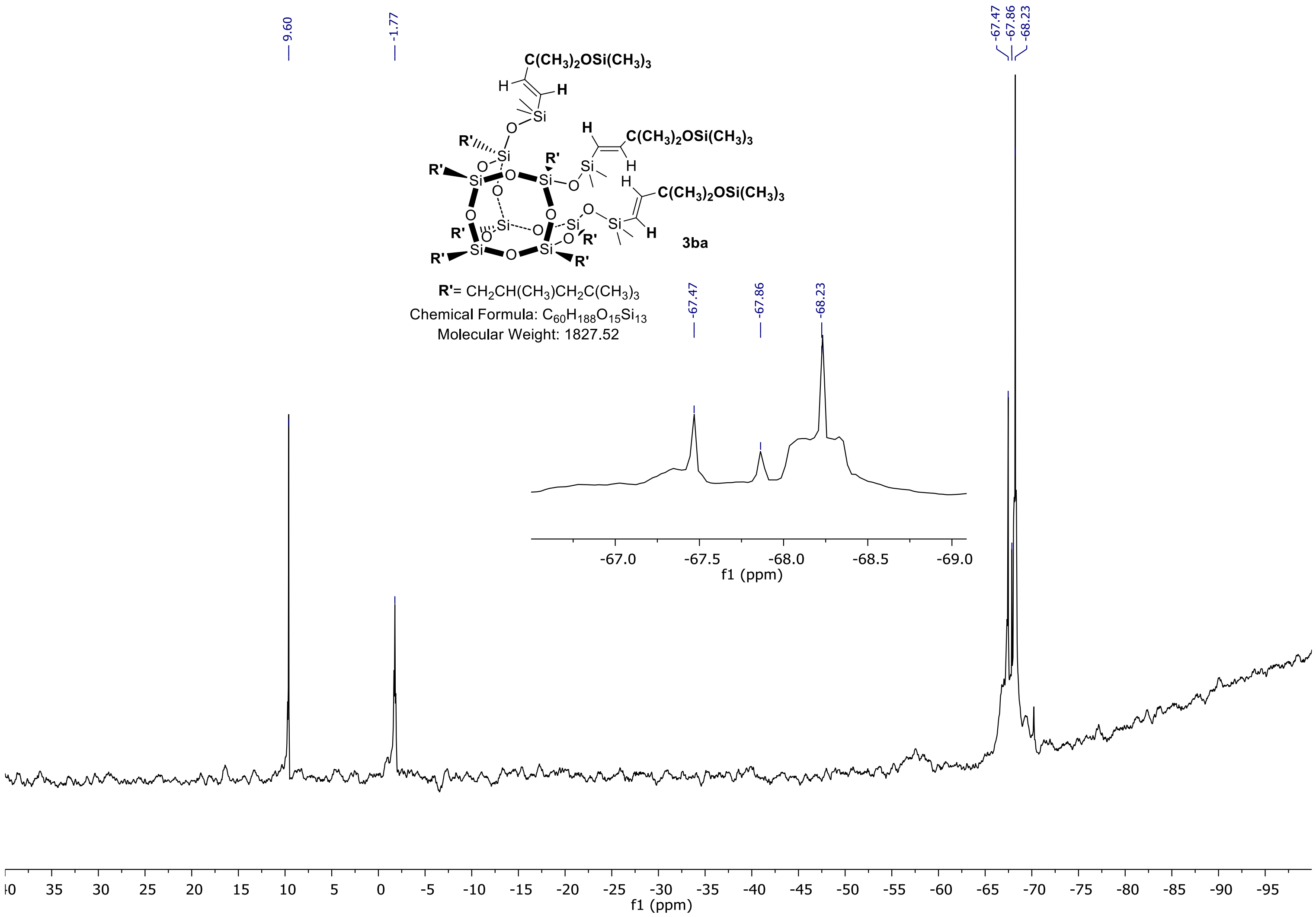

Figure S18. ^{29}Si NMR of compound **3ba**.

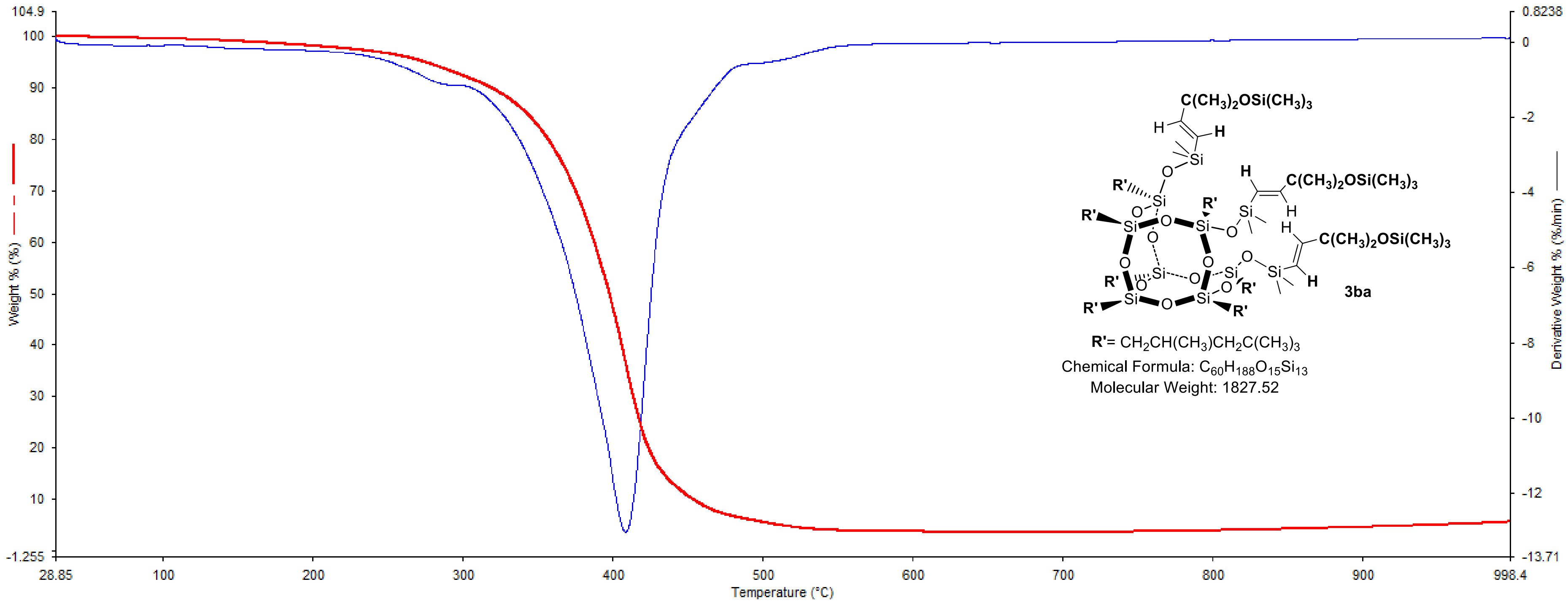

Figure S19. TGA/DTG curves of compound **3ba**.

3ab

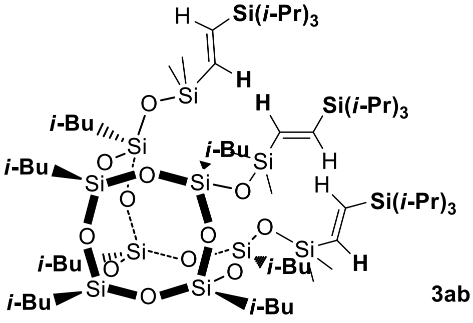

3ab

Chemical Formula: $C_{64}H_{150}O_{12}Si_{13}$
Molecular Weight: 1513.01

Isolated yield = 92%, colorless oil.

^{1}H NMR ($CDCl_3$, 300 MHz, δ, ppm): 0.21 (s, 18H, SiC$\underline{H}_3$), 0.55-0.58 (m, 14H, C$\underline{H}_2$), 0.94-0.97 (m, 42H, C$\underline{H}_3$), 1.03, 1.05 (d, 54H, Si(CH(C$\underline{H}_3$)$_2$)$_3$), 1.07-1.14 (m, 9H, Si(C$\underline{H}$(CH_3)$_2$)$_3$), 1.82-1.87 (m, 7H, C$\underline{H}$), 6.54 (d, 3H, =C($\underline{H}$), $J_{(H,H)}$= 23.07 Hz), 6.65 (d, 3H, =C($\underline{H}$), $J_{(H,H)}$= 23.11 Hz). **^{13}C NMR** ($CDCl_3$, 75 MHz, δ, ppm): 0.57 (Si$\underline{C}H_3$), 10.84 (Si(CH($\underline{C}H_3$)$_2$)$_3$), 18.78 (Si($\underline{C}$H(CH_3)$_2$)$_3$), 22.65, 23.90, 24.03 ($\underline{C}H_2$CH(CH_3)$_2$), 24.16, 24.25, 25.16 ($CH_2$$\underline{C}$H($CH_3$)$_2$), 25.80, 26.02, 26.18 ($CH_2$CH($\underline{C}H_3$)$_2$), 145.62 (=$\underline{C}$Si($CH_3$)$_2$O), 152.90 (=$\underline{C}$(H)Si((CH($CH_3$)$_2$)$_3$). **^{29}Si NMR** ($CDCl_3$, 79 MHz, δ, ppm): -68.10, -67.92, -67.24 (SiO_3), -4.53 (Si(CH(CH_3)$_2$)$_3$), -1.71 (OSi(CH_3)$_2$). **FT IR (cm^{-1}):** 2953.1, 2865.8, 1463.9, 1382.5, 1331.3, 1251.3, 1076.9, 1047.0, 1014.7, 881.8, 836.8, 794.1, 737.7, 444.2. **MALDI TOF MS** - (m/z) ([M+Na], (%)): 1535.80.

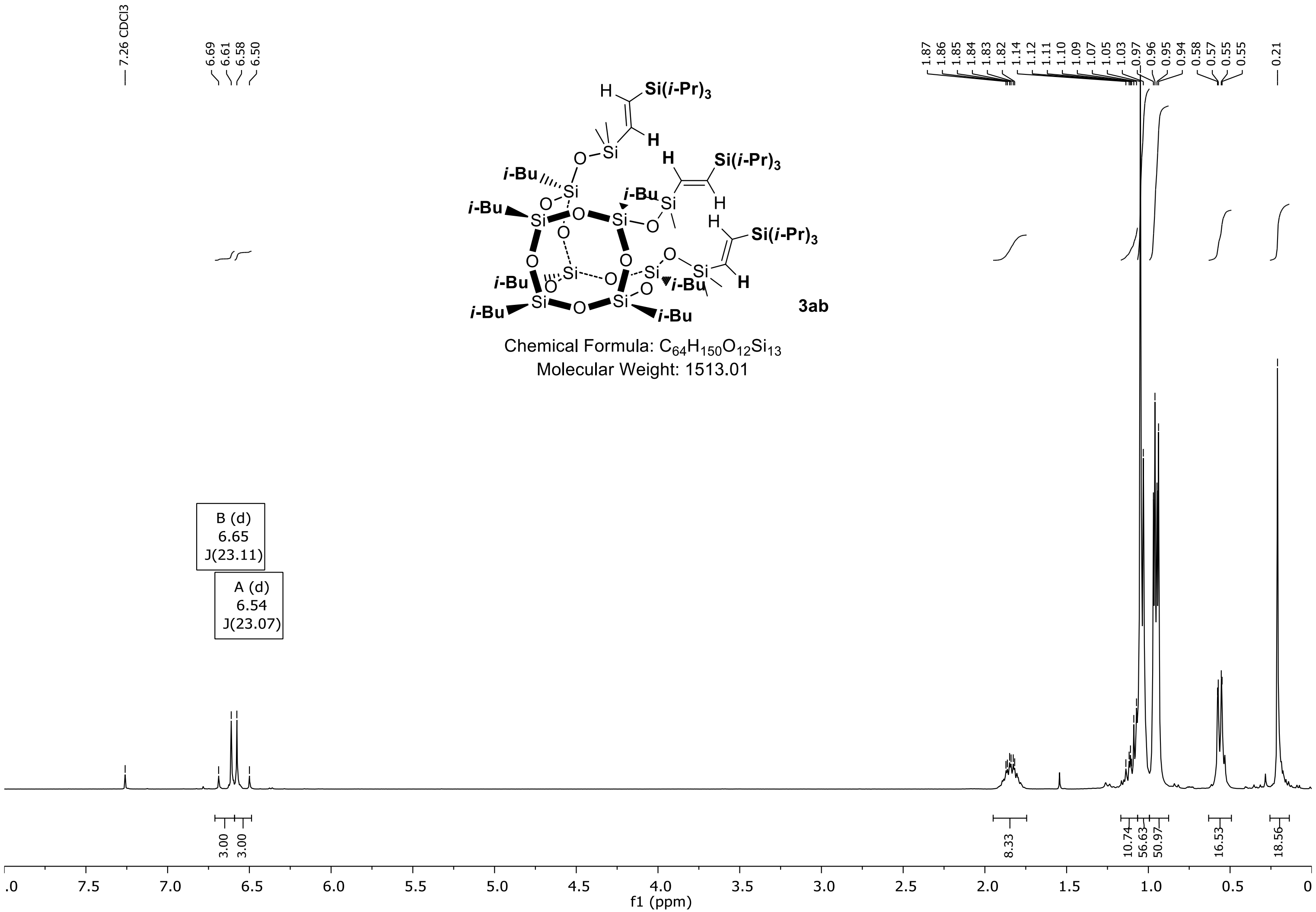

Figure S20. ^{1}H NMR of compound **3ab**.

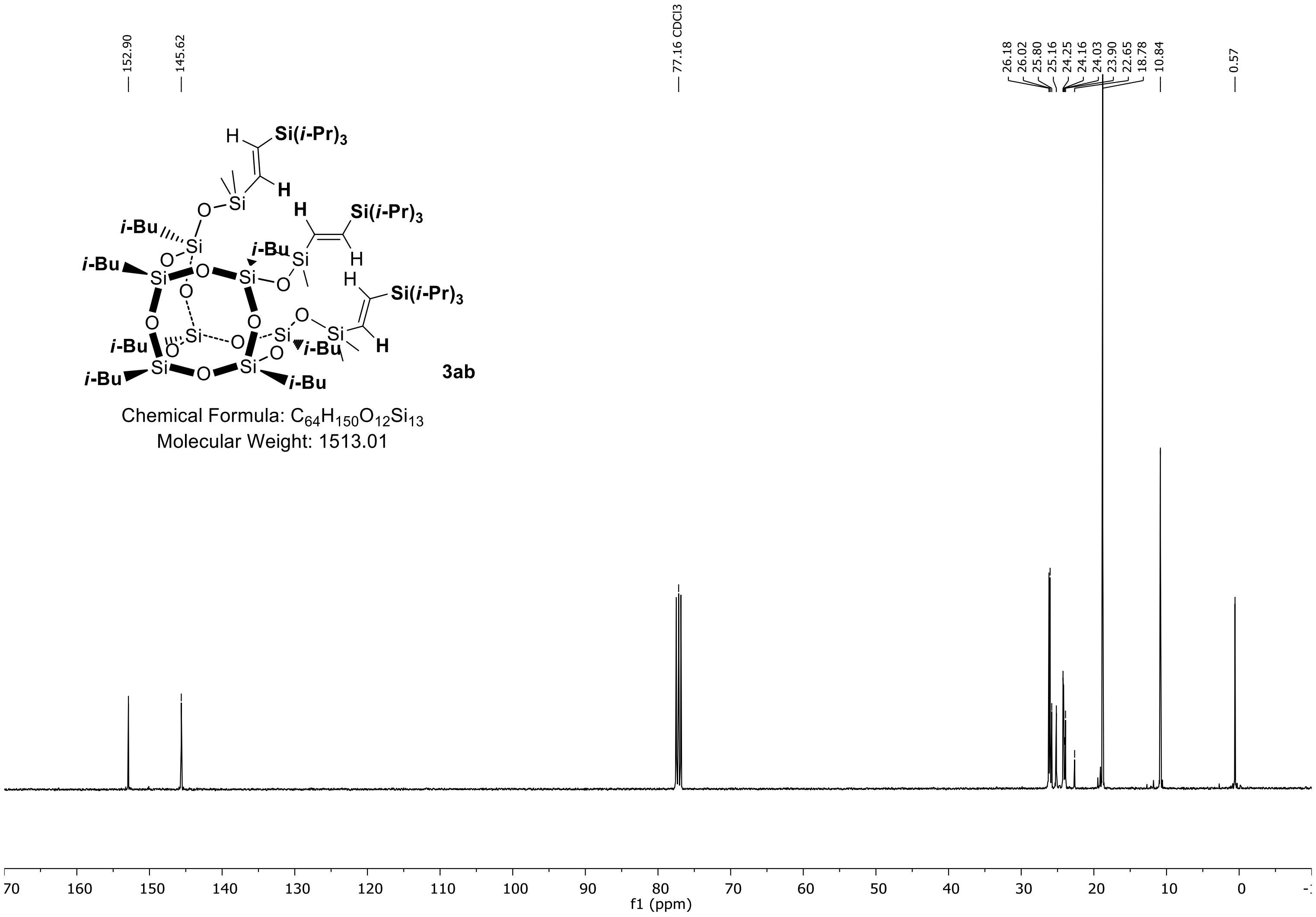

Figure S21. ^{13}C NMR of compound **3ab**.

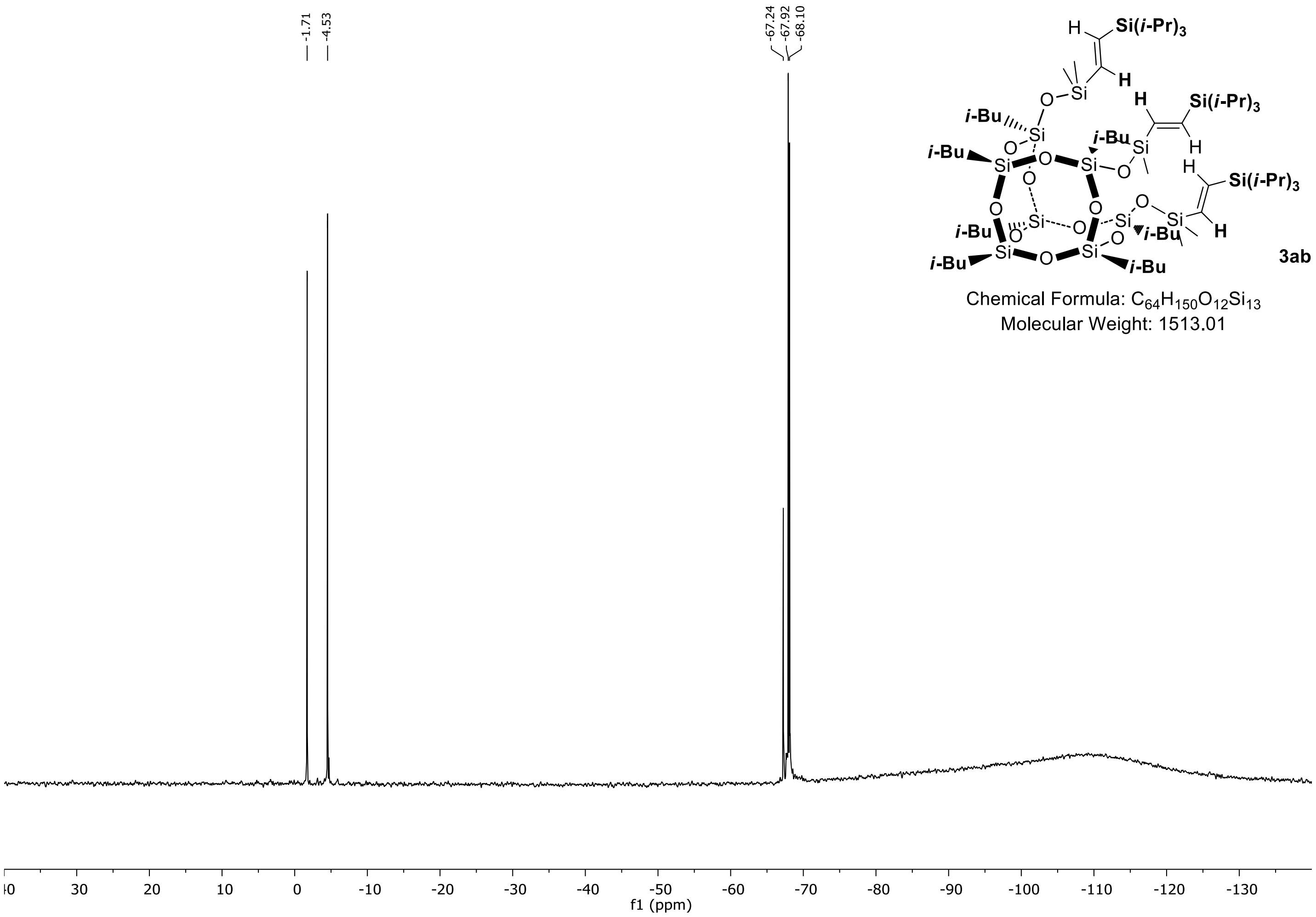

Figure S22. ^{29}Si NMR of compound **3ab**.

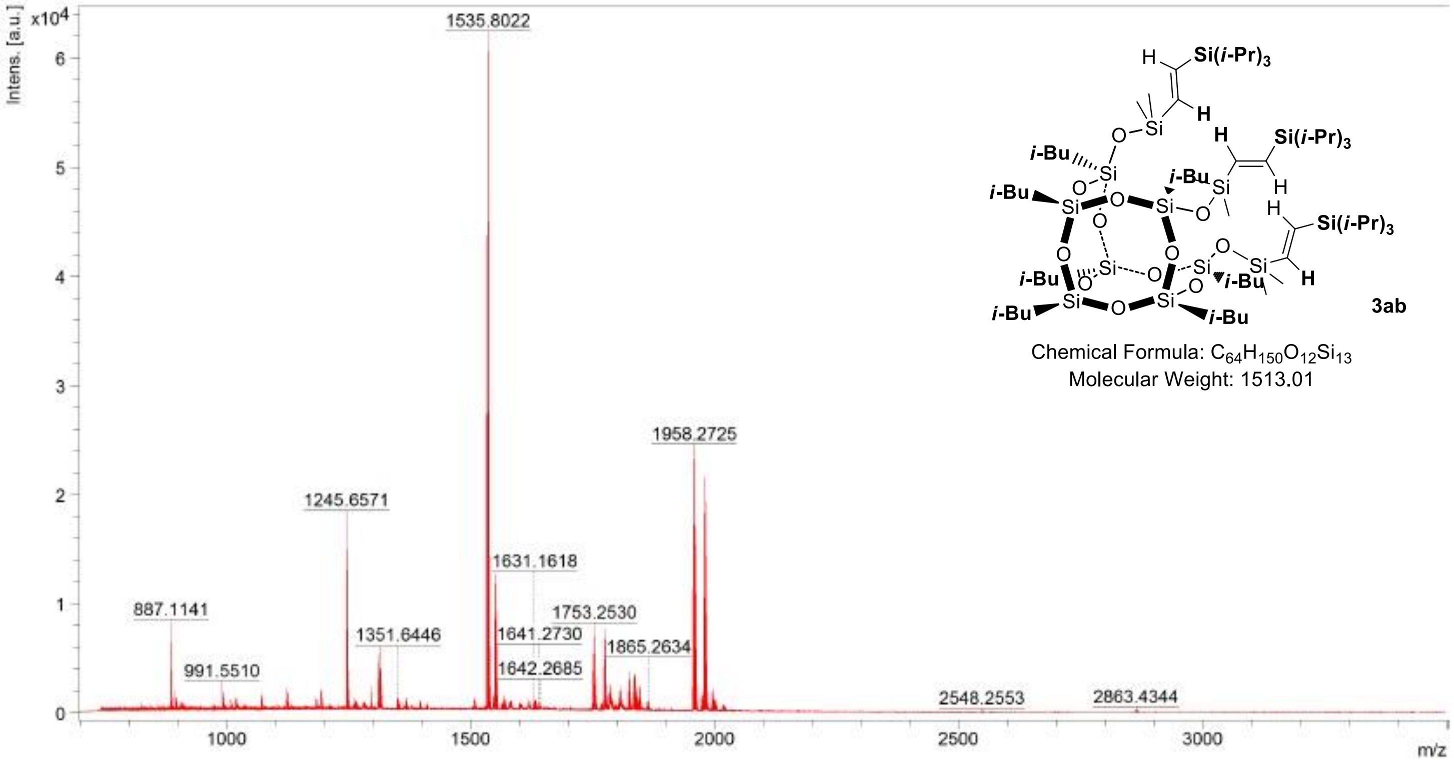

Figure S23. MALDI TOF MS spectra of compound **3ab**.

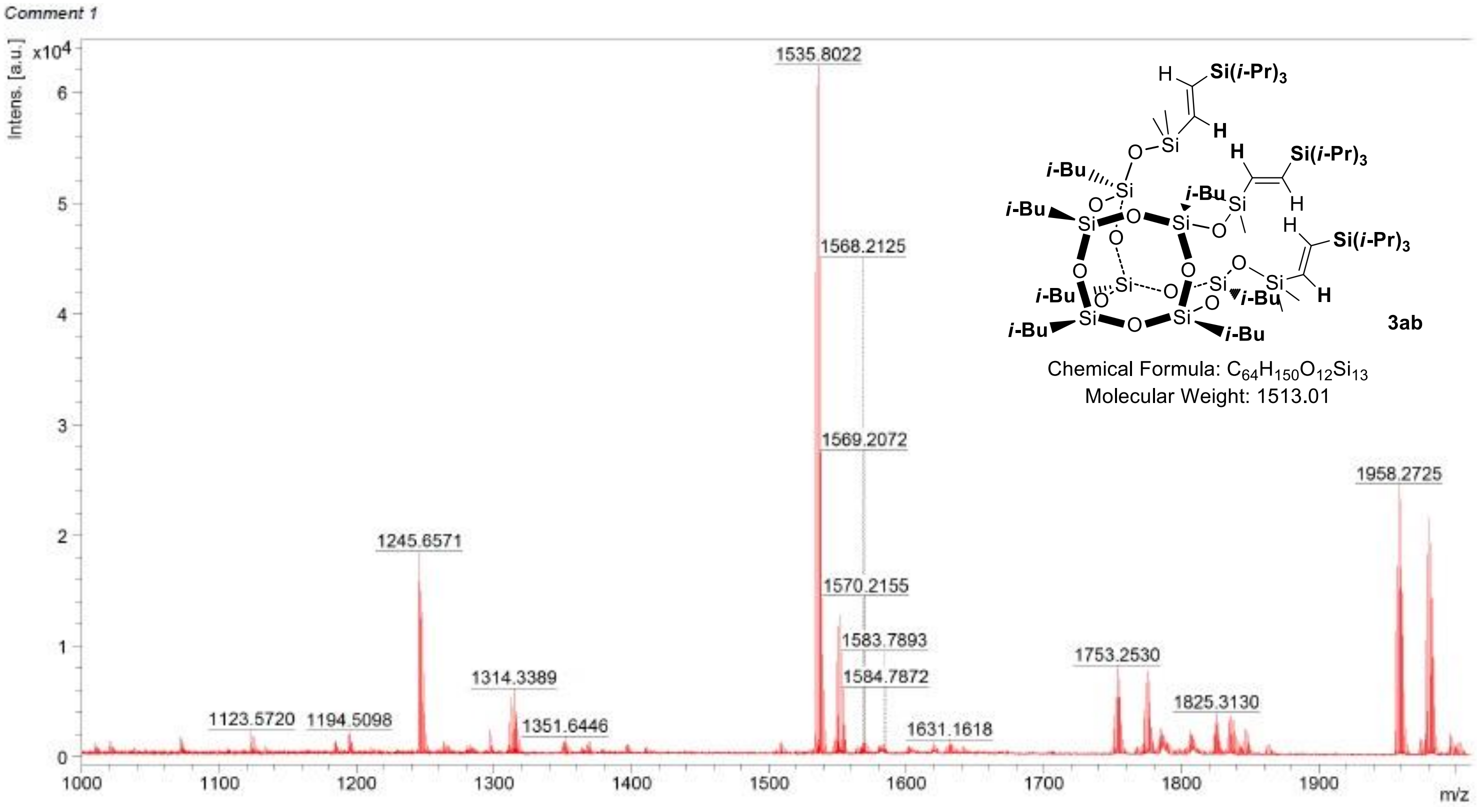

Figure S24. MALDI TOF MS spectra of compound **3ab**.

Molecular Weight Averages

Peak	Mp	Mn	Mw	Mz	Mz+1	Mv	PD
Peak 1	1708	1630	1734	1842	1958	1825	1.064

Peak information

	Start (mins)	End (mins)
Baseline region 1	2.70	9.85
Peak 1	10.78	11.78

Peak	Trace	Peak Max RT (mins)	Peak Area (mV.s)	Peak Height (mV)
Peak 1	RI	11.40	81093.834	4743.953
Peak 1	UV 250nm	11.08	3595.968	-174.941

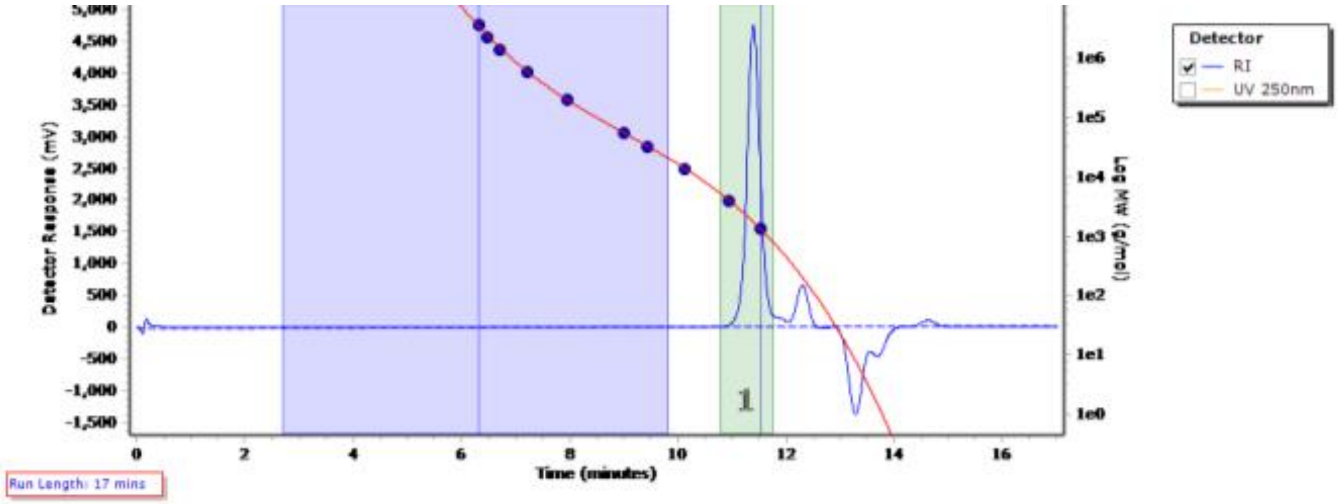

Figure S25. GPC chromatogram of compound **3ab**.

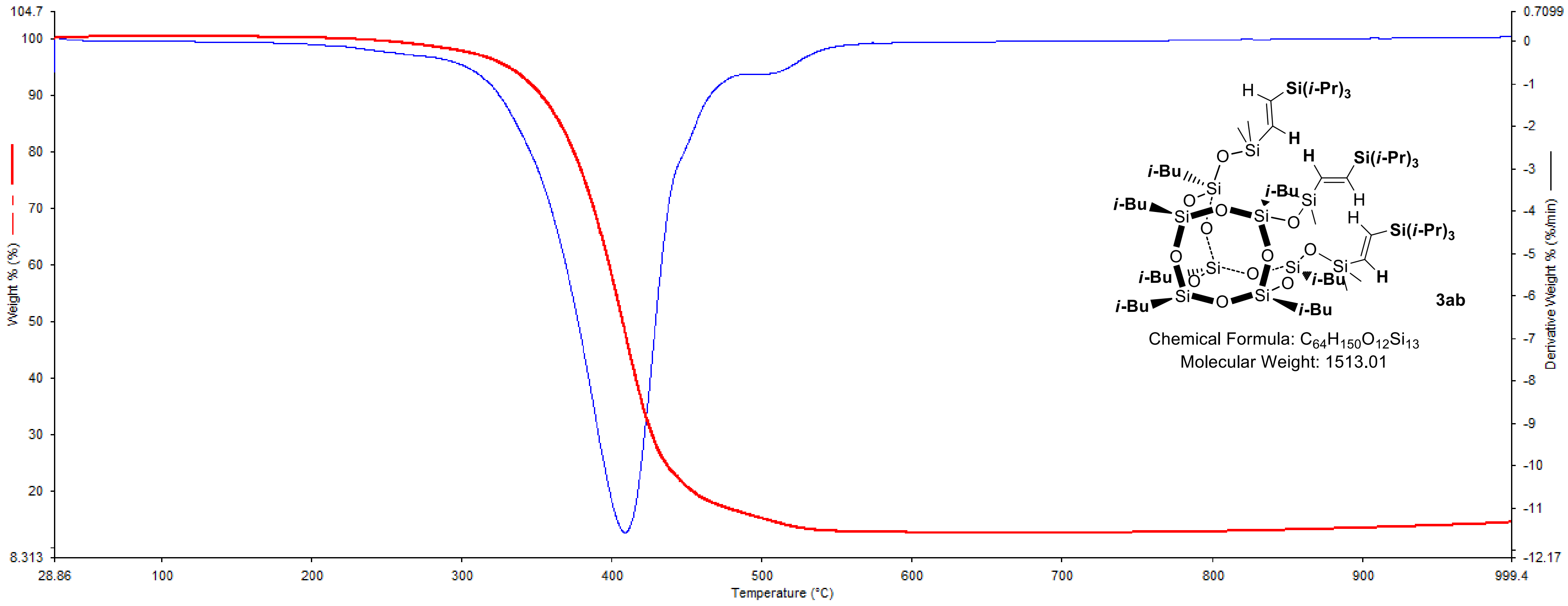

Figure S26. TGA/DTG curves of compound **3ab**.

3bb

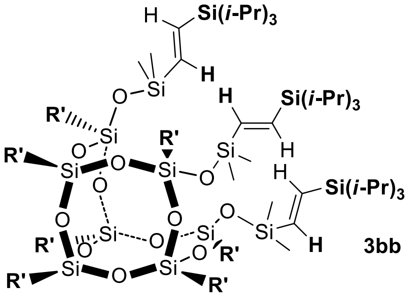

3bb

R'= $CH_2CH(CH_3)CH_2C(CH_3)_3$

Chemical Formula: $C_{69}H_{206}O_{12}Si_{13}$

Molecular Weight: 1905.76

Isolated yield = 92%, colorless oil.

^{1}H NMR ($CDCl_3$, 300 MHz, δ, ppm): 0.22 (s, 18H, $SiC\underline{H}_3$), 0.49-0.58 (m, 7H, $SiC\underline{H}_2$), 0.72-0.78 (m, 7H, $SiC\underline{H}_2$), 0.89-0.90 (m, 63H, $C(C\underline{H}_3)_3$), 1.03, 1.05 (m, 75H, $Si(CH(C\underline{H}_3)_2)_3$, $CH(C\underline{H}_3)$), 1.09-1.26 (m, 23H, $C\underline{H}_2$, $Si(C\underline{H}(CH_3)_2)_3$), 1.81-1.84 (m, 7H, $C\underline{H}(CH_3)$), 6.53 (d, 3H, =C($\underline{H}$), $J_{(H,H)}$= 23.05 Hz), 6.66 (d, 3H, =C($\underline{H}$), $J_{(H,H)}$= 23.09 Hz). **^{13}C NMR** ($CDCl_3$, 75 MHz, δ, ppm): 0.73 ($OSi\underline{C}H_3$), 10.85 ($Si(CH(\underline{C}H_3)_2)_3$), 18.83 ($Si(\underline{C}H(CH_3)_2)_3$), 22.83, 23.55, 23.82, 25.07, 25.17, 25.25, 25.38, 25.50, 25.53, 25.89, 26.53 ($\underline{C}H$, $Si\underline{C}H_2$, $CH(\underline{C}H_3)$), 30.32, 30.46, 30.49 ($(\underline{C}H_3)_3$), 31.34 ($\underline{C}(CH_3)_3$), 54.80, 55.21 ($\underline{C}H_2C(CH_3)_3$), 145.53 ($=\underline{C}Si(CH_3)_2O$), 153.01 ($=\underline{C}(H)Si((CH(CH_3)_2)_3)$. **^{29}Si NMR** ($CDCl_3$, 79 MHz, δ, ppm): -68.31, -68.41, -68.23, -67.49 (SiO_3), -4.68, -4.56, -4.44 ($Si(CH(CH_3)_2)_3$), -1.73 ($OSi(CH_3)_2$). **FT IR (cm^{-1}):** 2951.8, 2865.4, 1464.6, 1364.1, 1250.7, 1226.1, 1077.1, 1046.6, 908.3, 881.3, 836.5, 793.8, 726.0, 432.9.

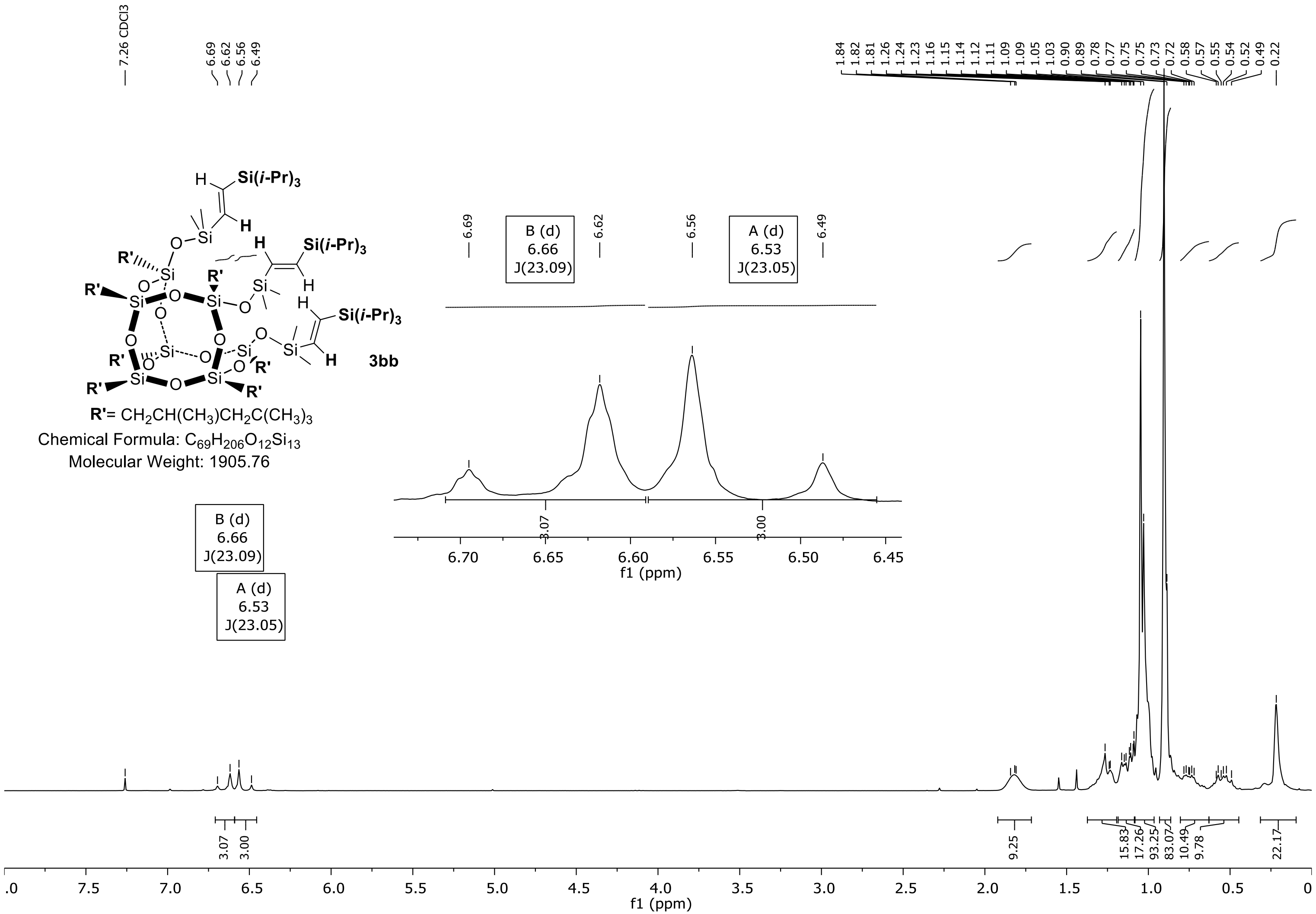

Figure S27. ^{1}H NMR of compound **3bb**.

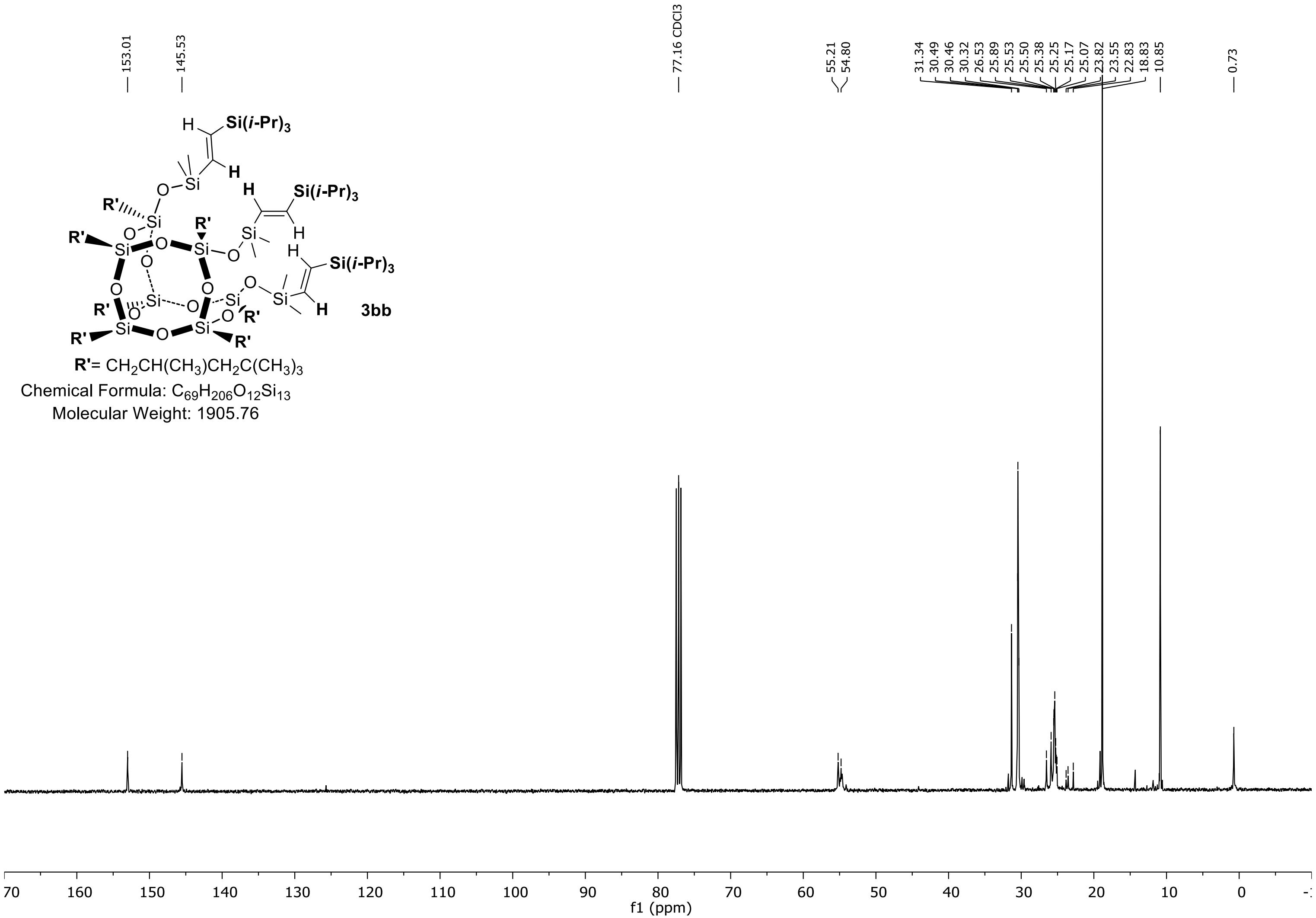

Figure S28. ^{13}C NMR of compound **3bb**.

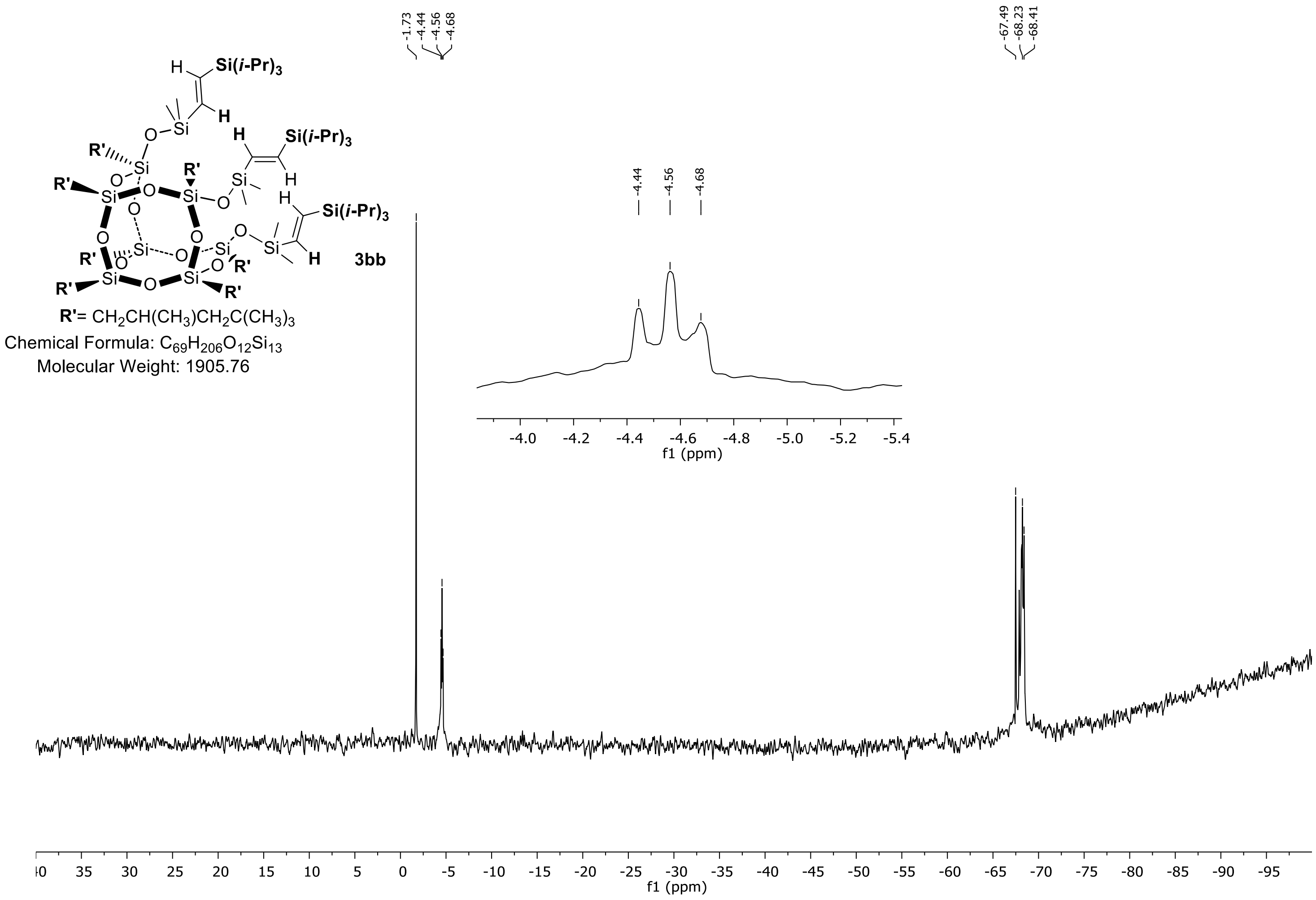

Figure S29.^{29}Si NMR of compound **3bb**.

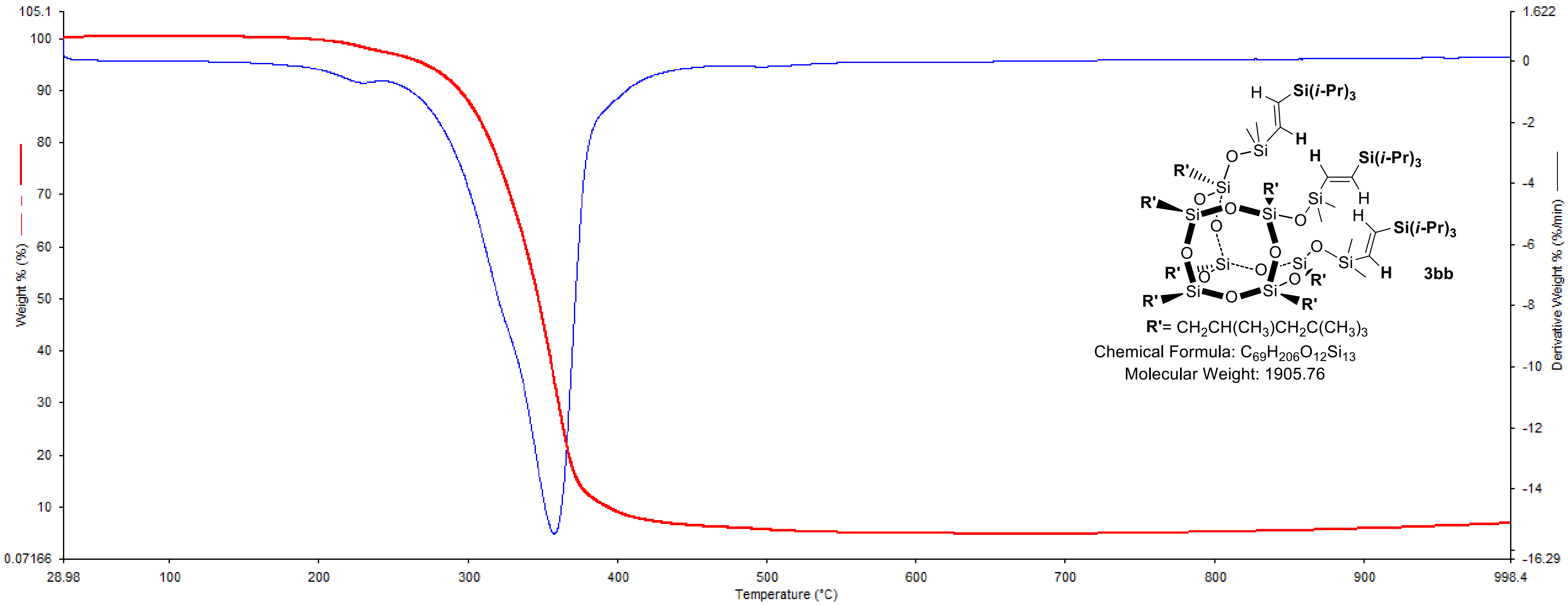

Figure S30. TGA/DTG curves of compound **3bb**.

3ac

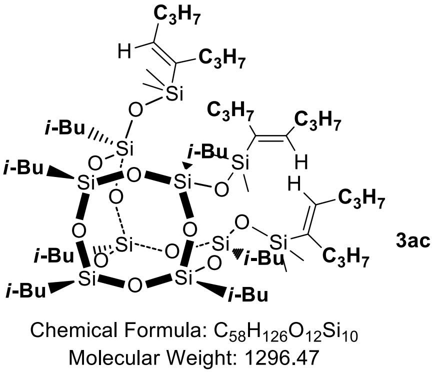

Isolated yield = 95%, colorless oil.

^{1}H NMR ($CDCl_3$, 300 MHz, δ, ppm): 0.19 (s, 18H, SiC<u>H</u>$_3$), 0.55-0.58 (m, 14H, C<u>H</u>$_2$), 0.88-0.93 (m, 18H, CH_2C<u>H</u>$_3$), 0.95-0.98 (m, 42H, C<u>H</u>$_3$), 1.32-1.42 (m, 12H, CH_2C<u>H</u>$_2CH_3$), 1.83-1.88 (m, 7H, C<u>H</u>), 2.04-2.12 (m, 12H, C<u>H</u>$_2CH_2CH_3$), 5.80-5.84 (t, 3H, $J_{(H,H)}$= 6.85Hz, =C<u>H</u>). **^{13}C NMR** ($CDCl_3$, 75 MHz, δ, ppm): 0.95 (Si<u>C</u>H_3), 14.14, 14.66 (CH_2<u>C</u>H_3), 22.67, 22.85, 23.49 (<u>C</u>H_2CH_3), 23.97, 24.00, 24.05, 24.17, 24.21 (<u>C</u>$H_2CH(CH_3)_2$), 25.31, 25.81 (CH_2<u>C</u>$H(CH_3)_2$), 26.07, 26.25 (CH_2CH(<u>C</u>$H_3)_2$), 30.57, 31.45 (=C(H)<u>C</u>H_2, =C(Si)<u>C</u>H_2), 140.85 (=<u>C</u>$Si(CH_3)_2O$), 140.99 (=<u>C</u>H). **^{29}Si NMR** ($CDCl_3$, 79 MHz, δ, ppm): -68.18, -67.78, -67.40 (SiO_3), -1.14 ($OSi(CH_3)_2$). **FT IR (cm^{-1}):** 2953.6, 2929.9, 2870.2, 1613.3, 1465.2, 1401.5, 1365.8, 1331.5, 1250.7, 1227.4, 1074.5, 1045.1, 833.7, 777.8, 738.1, 444.7. **MALDI TOF MS** - (m/z) ([M+Na], (%)): 1318.66.

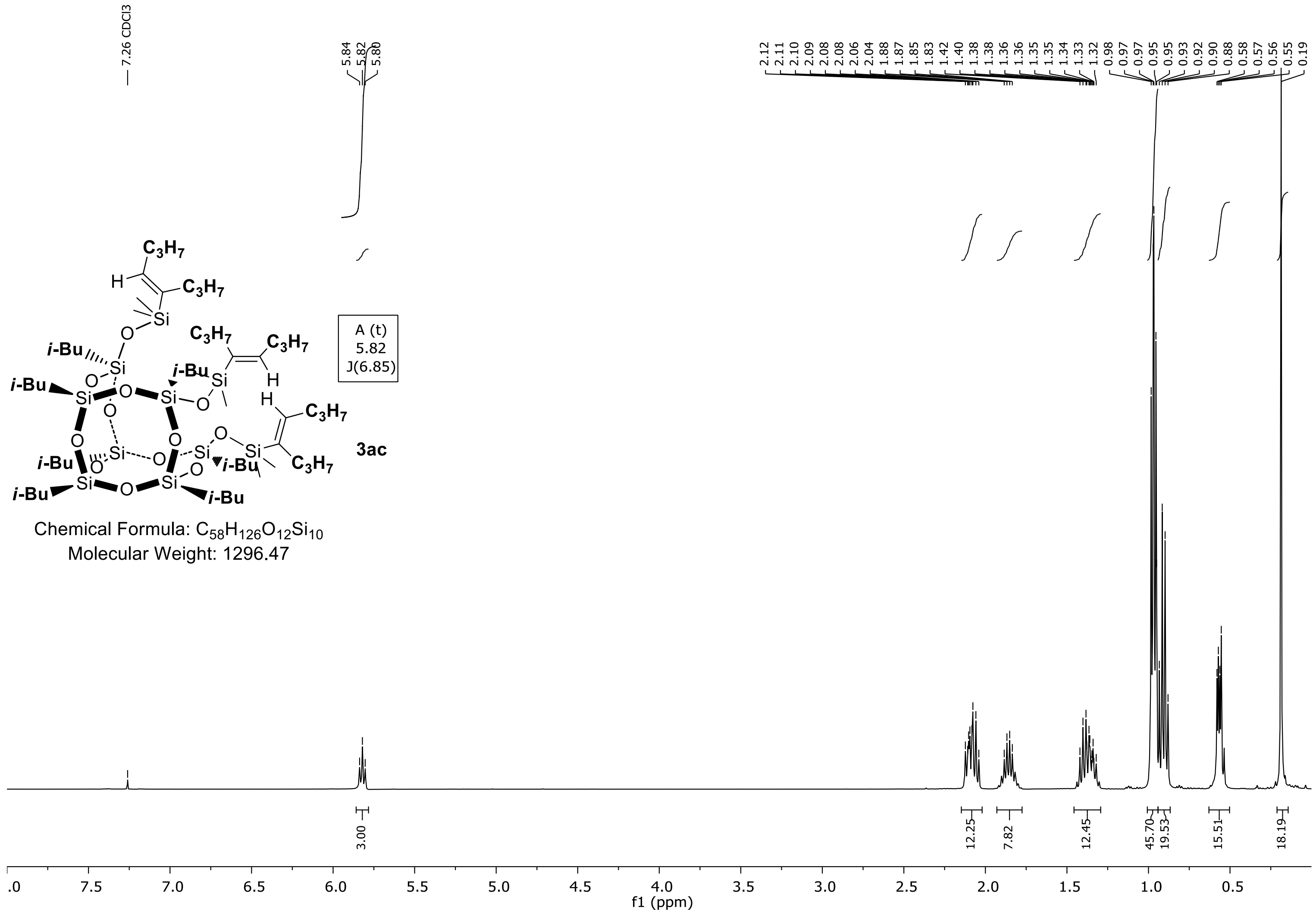

Figure S31. ^{1}H NMR of compound **3ac**.

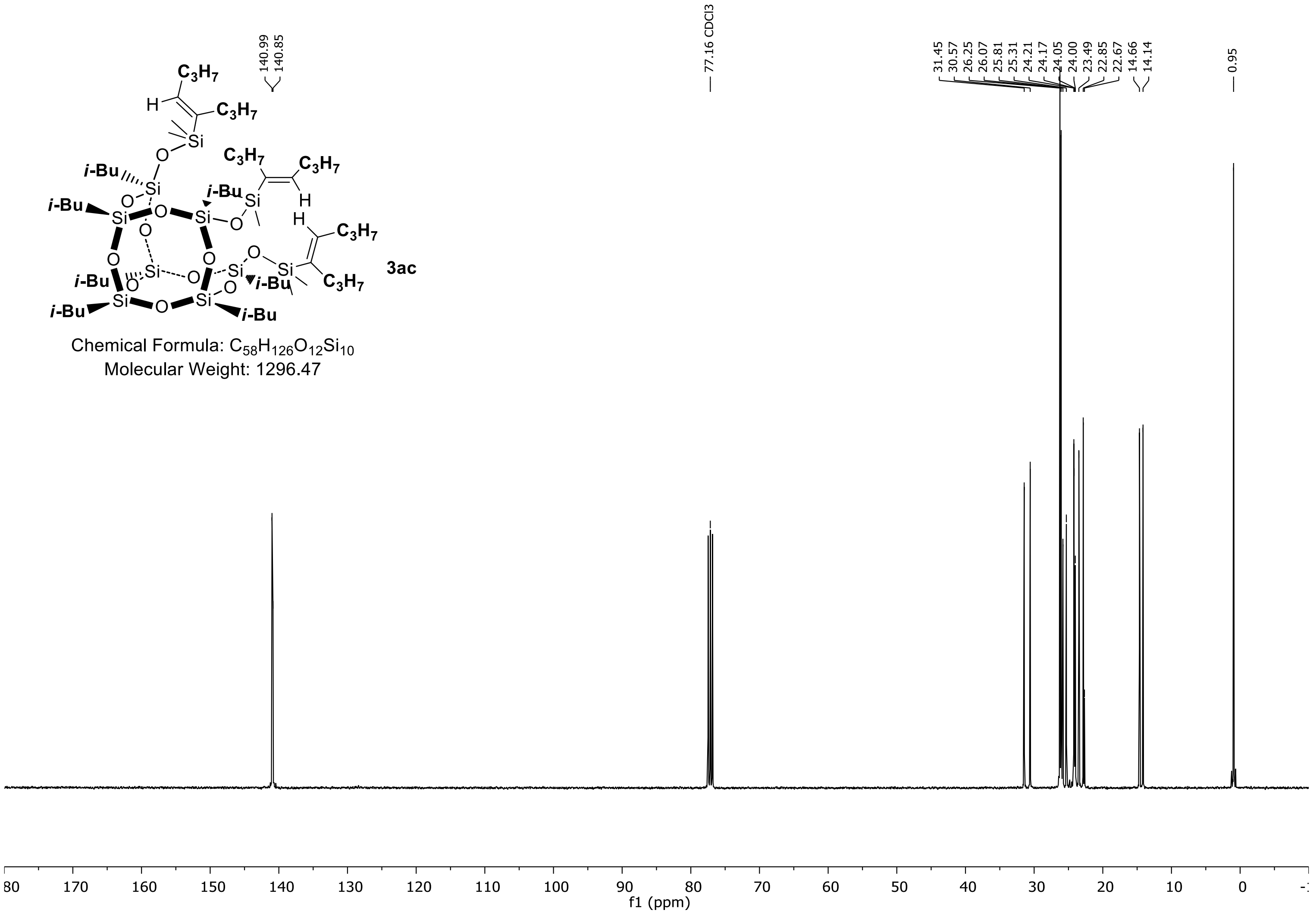

Figure S32. ^{13}C NMR of compound **3ac**.

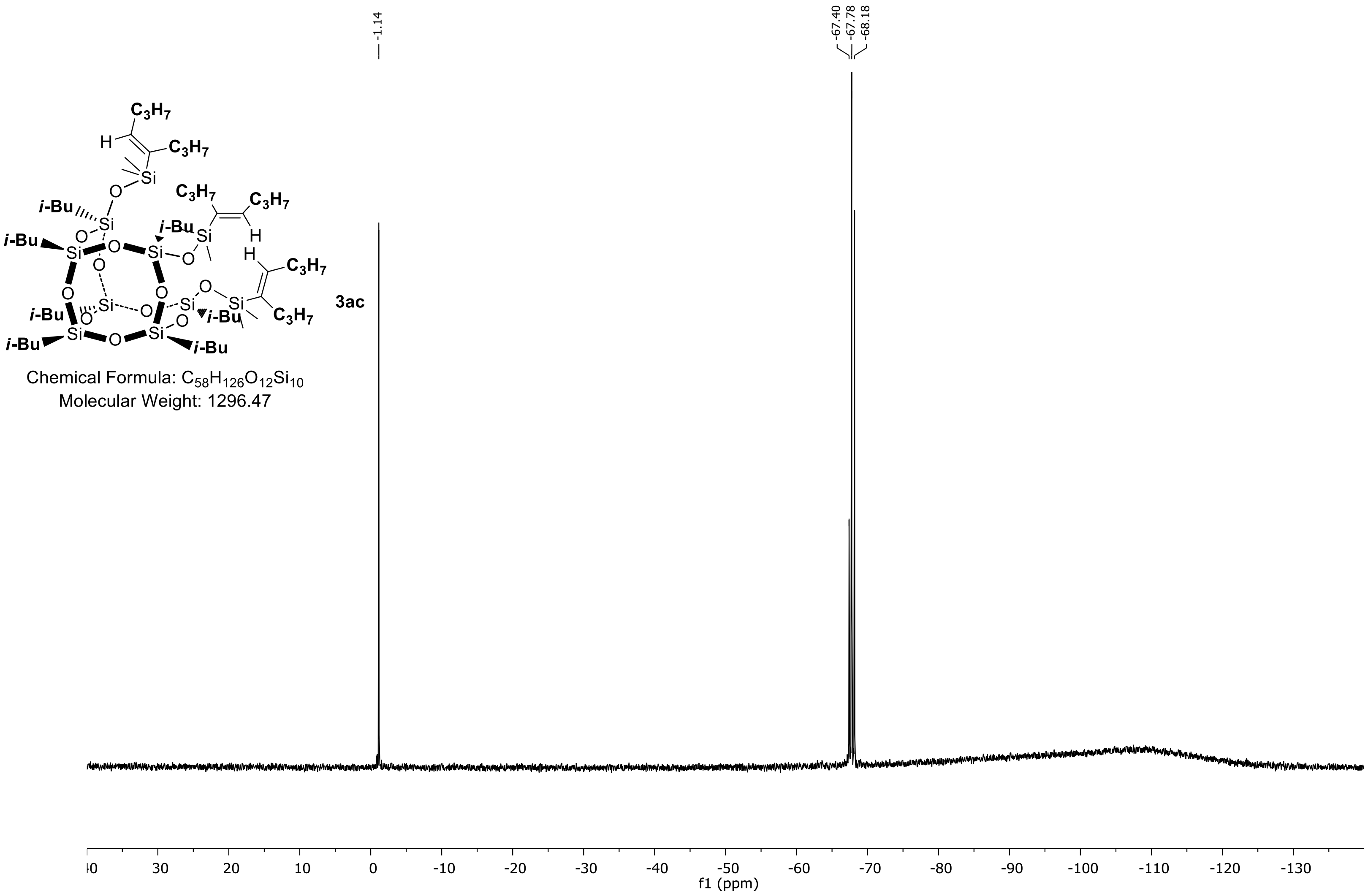

Figure S33. ^{29}Si NMR of compound **3ac**.

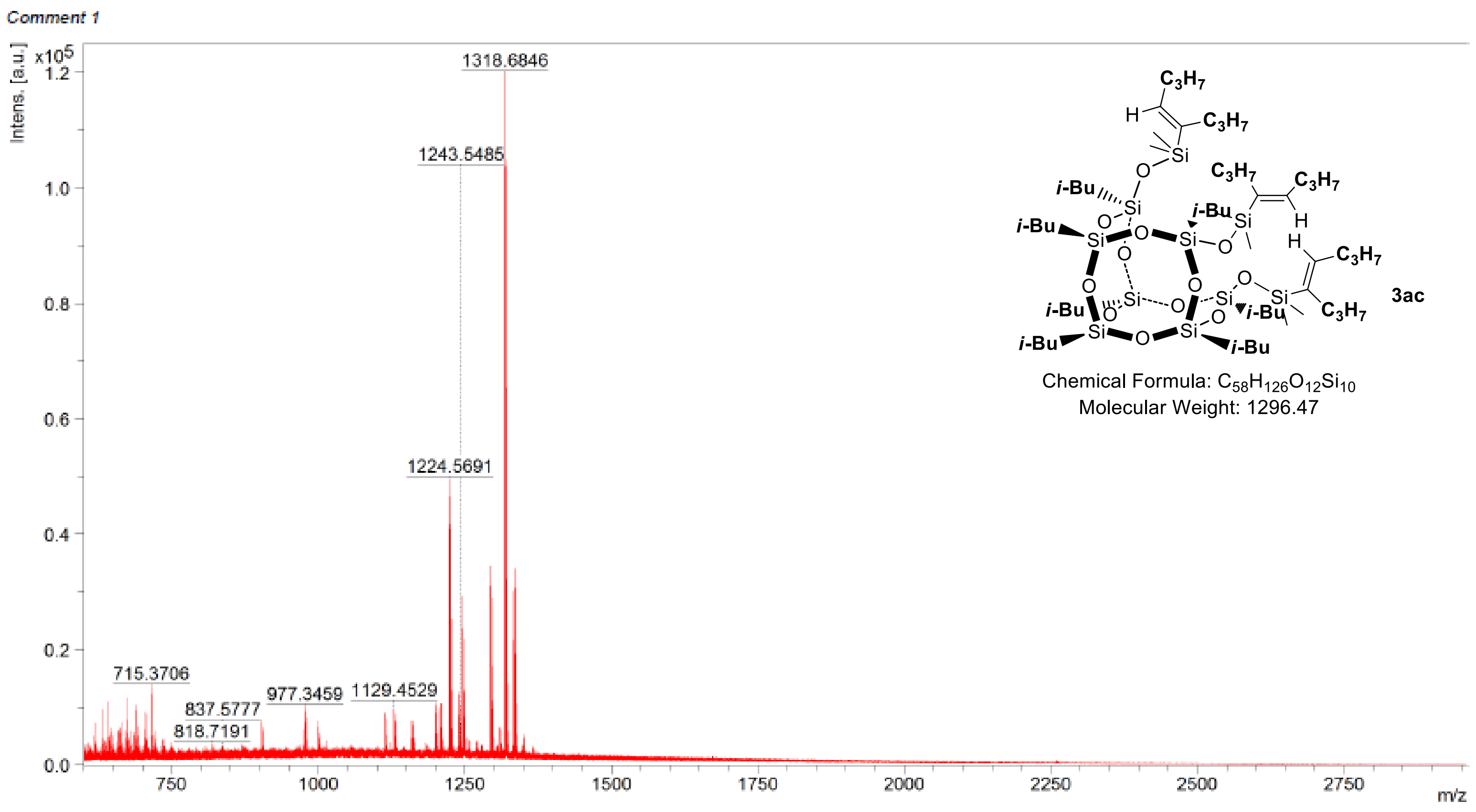

Figure S34. MALDI TOF MS spectra of compound **3ac**.

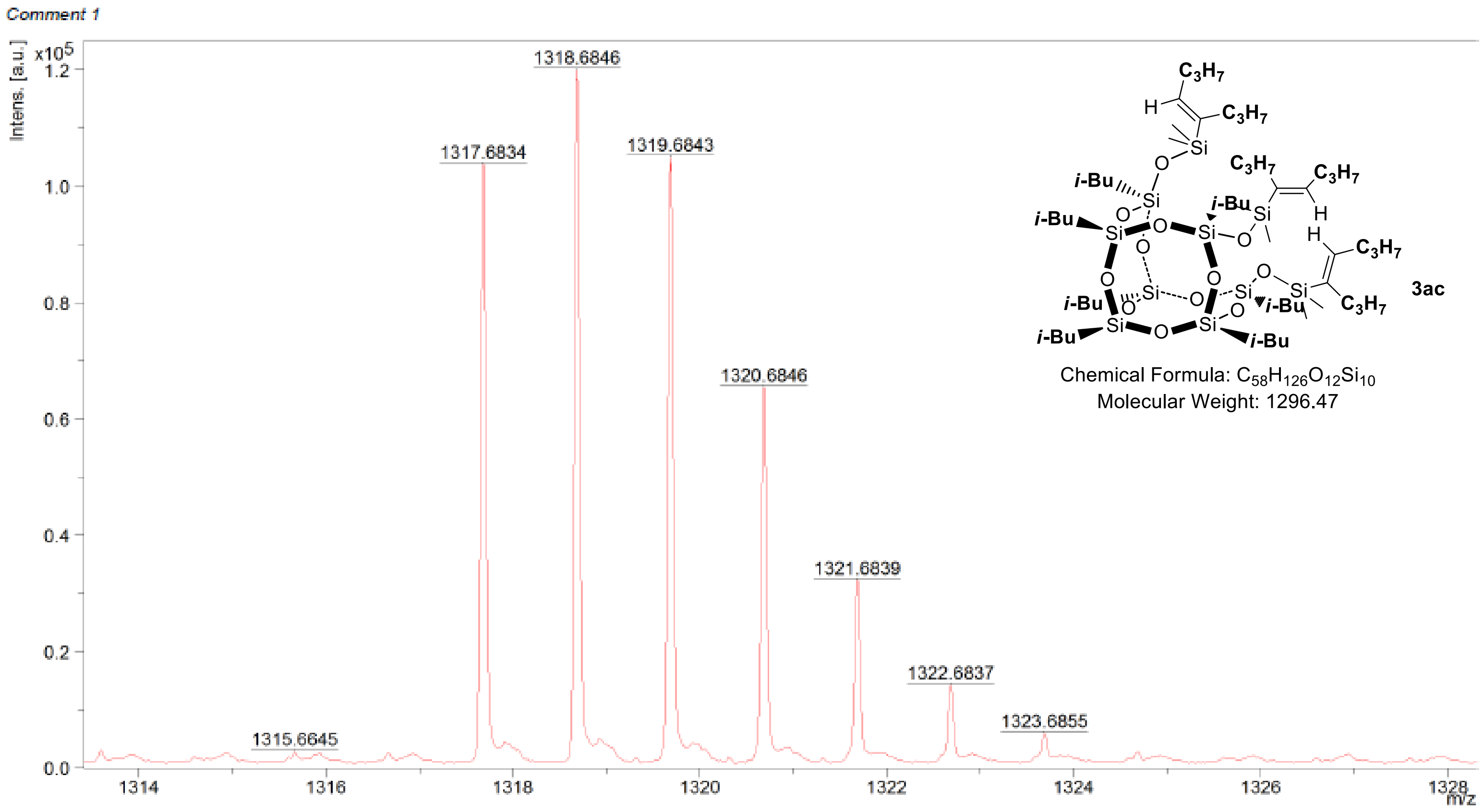

Figure S35. MALDI TOF MS spectra of compound **3ac**.

3bc

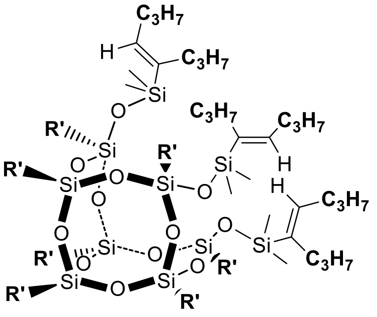

3bc

R'= $CH_2CH(CH_3)CH_2C(CH_3)_3$
Chemical Formula: $C_{60}H_{182}O_{12}Si_{10}$
Molecular Weight: 1689.24

Isolated yield = 83%, colorless oil.

^{1}H NMR ($CDCl_3$, 300 MHz, δ, ppm): 0.19 (s, 9H, $SiC\underline{H}_3$), 0.20 (s, 9H, $SiC\underline{H}_3$), 0.54-0.57 (m, 7H, $SiC\underline{H}_2$), 0.77-0.79 (m, 7H, $SiC\underline{H}_2$), 0.89-0.93 (m, 81H, $C(C\underline{H}_3)_3$, CH_3), 1.00-1.06 (m, 21H, $CH(C\underline{H}_3)$), 1.13-1.18 (m, 7H, $C\underline{H}_2$), 1.23-1.27 (m, 7H, $C\underline{H}_2$), 1.34-1.42 (m, 12H, $CH_2C\underline{H}_2CH_3$), 1.82-1.84 (m, 7H, $C\underline{H}(CH_3)$), 2.05-2.09 (m, 12H, $C\underline{H}_2CH_2CH_3$), 5.81-5.85 (m, 3H, $=C\underline{H}$). **^{13}C NMR** ($CDCl_3$, 75 MHz, δ, ppm): 1.13 ($OSi\underline{C}H_3$), 14.20, 14.71 ($CH_2CH_2\underline{C}H_3$), 22.87, 23.50, 25.08, 25.30, 25.36, 25.55, 25.90, 26.68 ($\underline{C}H_2CH_3$, $\underline{C}H_2CH_2CH_3$),$\underline{C}H$, $Si\underline{C}H_2$, $CH(\underline{C}H_3)$), 30.33, 30.48, 30.60 (($\underline{C}H_3)_3$), 31.36, 31.44 ($\underline{C}(CH_3)_3$), 55.23, 55.42 ($\underline{C}H_2C(CH_3)_3$), 140.74 ($=\underline{C}Si(CH_3)_2O$), 141.02 ($=\underline{C}H$). **^{29}Si NMR** ($CDCl_3$, 79 MHz, δ, ppm): -68.28, -68.23, -68,12, -68.08, -67.84, -67.61 (SiO_3), -1.31, -1.21, -1.15 ($OSi(CH_3)_2$). **FT IR (cm^{-1}):** 2953.5, 2905.3, 2870.2, 1613.3, 1466.4, 1392.9, 1377.0, 1250.1, 1225.3, 1075.8, 1043.3, 907.3, 832.2, 779.2, 730.6, 429.6.

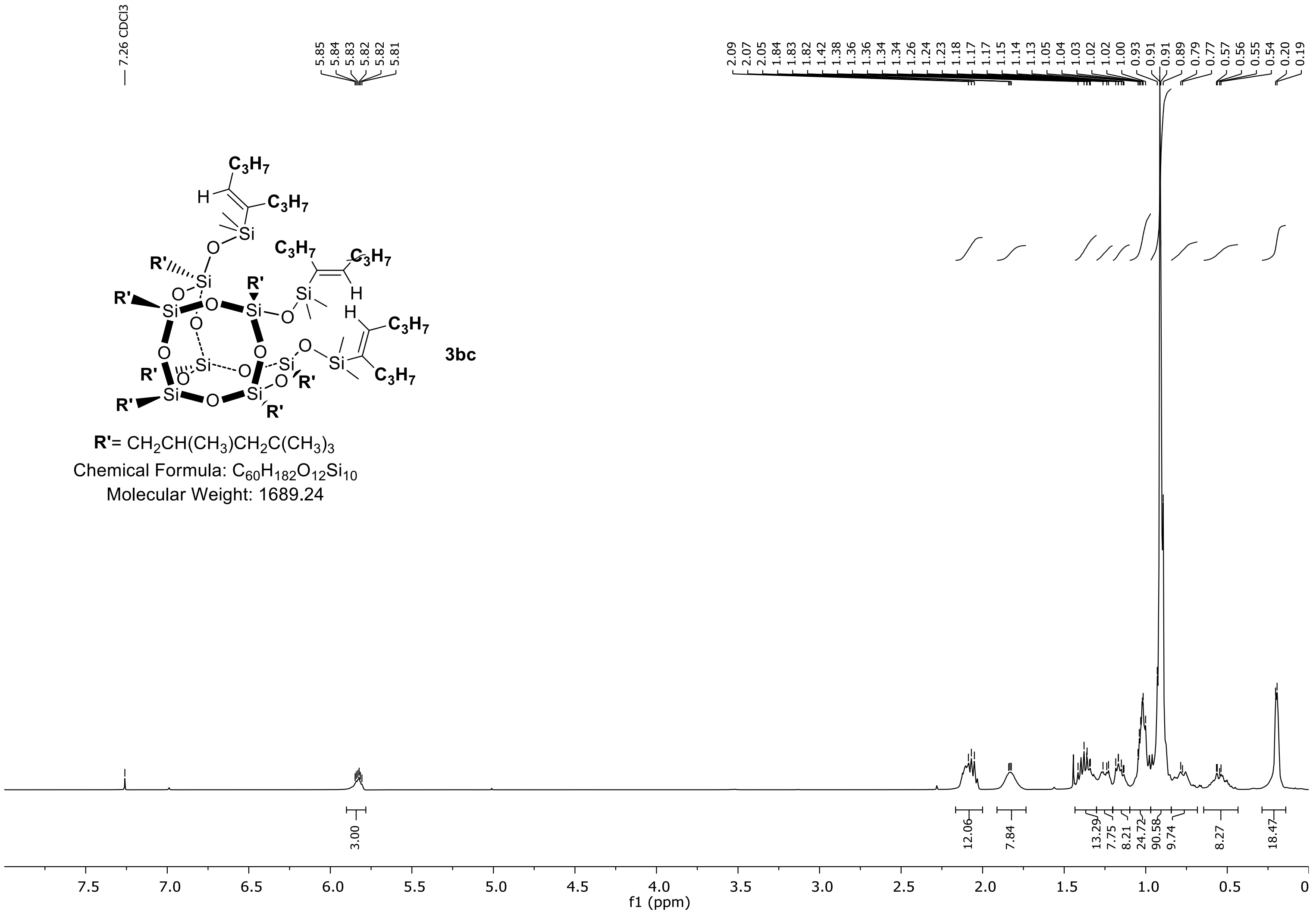

Figure S36. ^{1}H NMR of compound **3bc**.

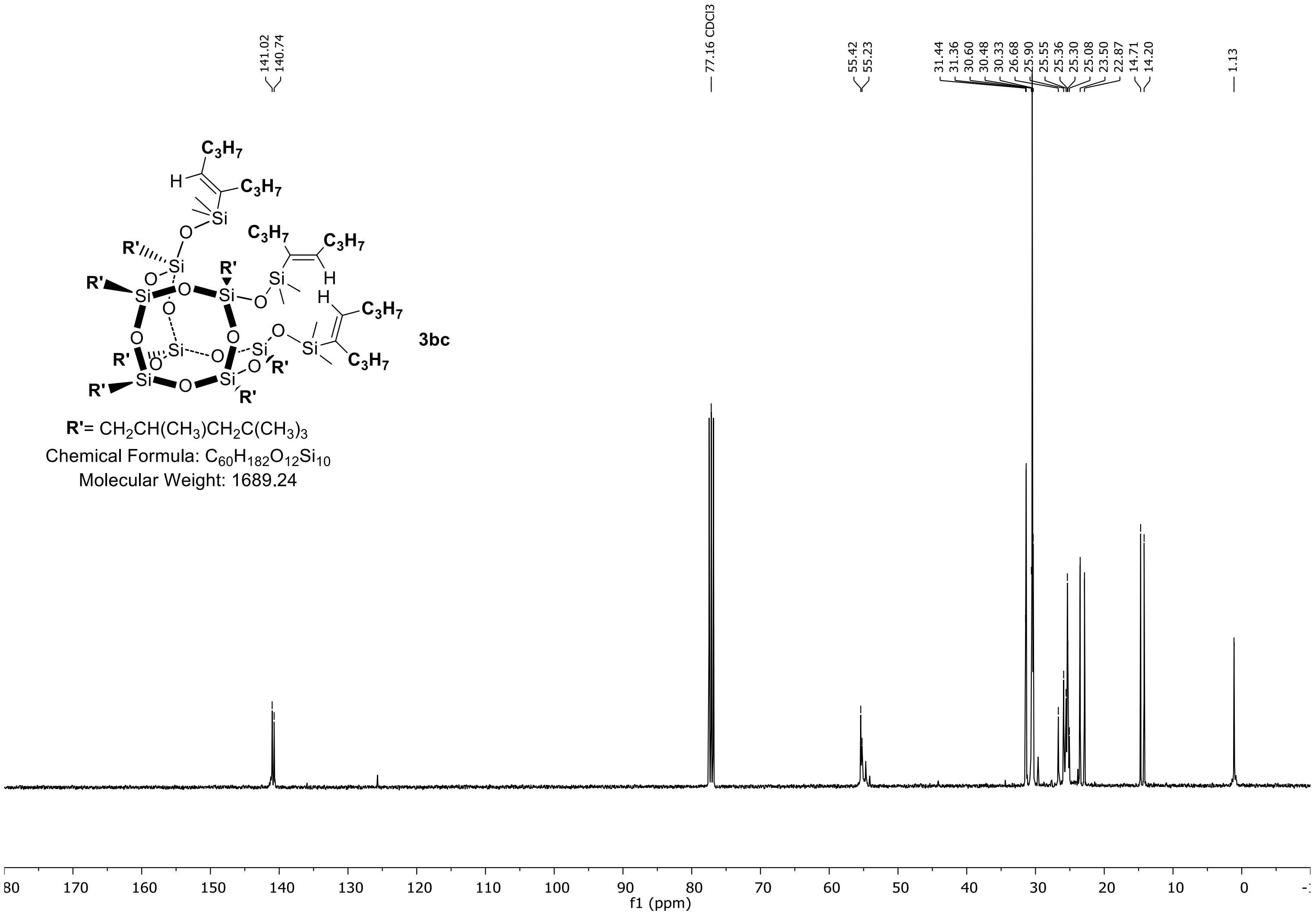

Figure S37. ^{13}C NMR of compound **3bc**.

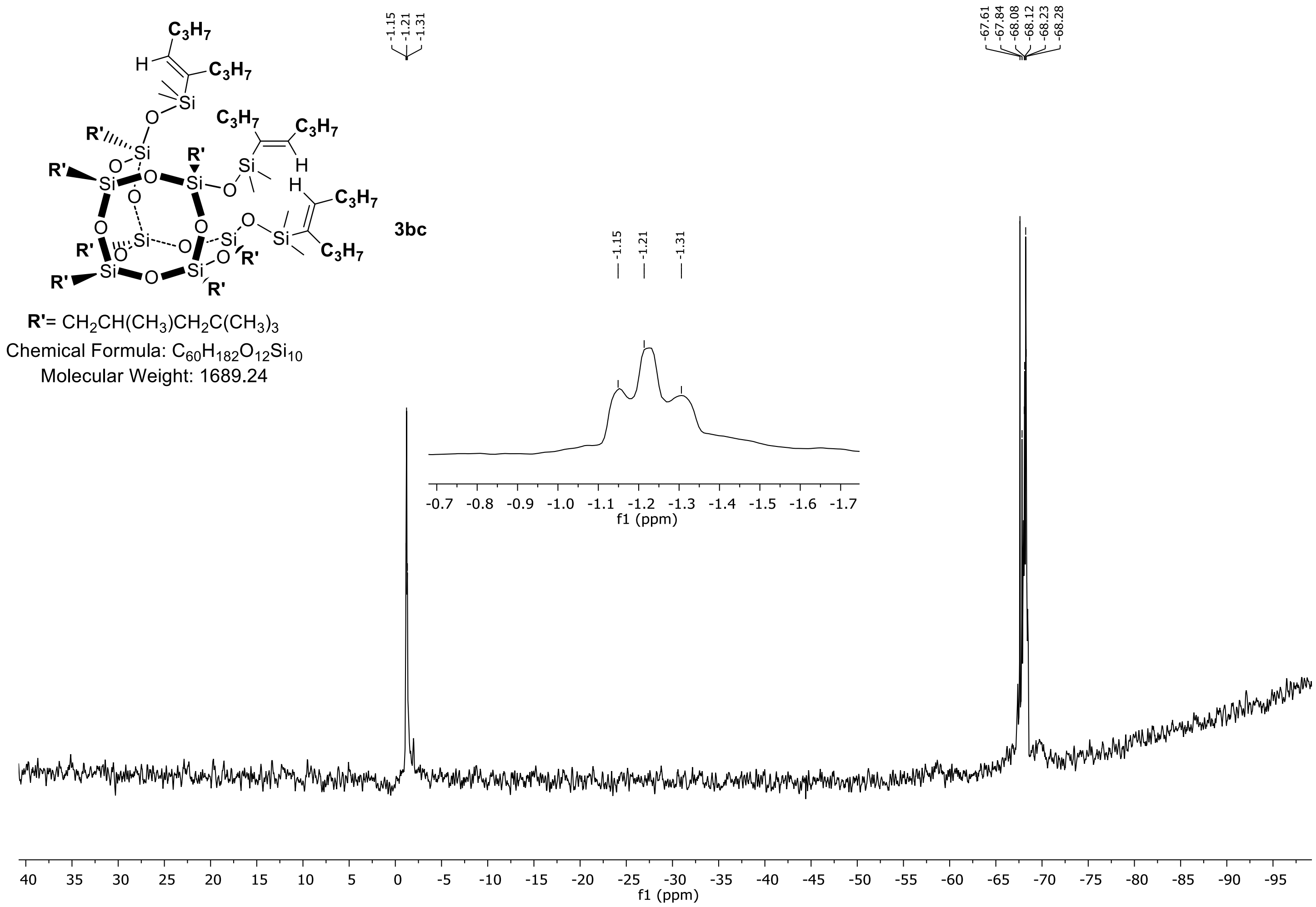

Figure S38. ^{29}Si NMR of compound **3bc**.

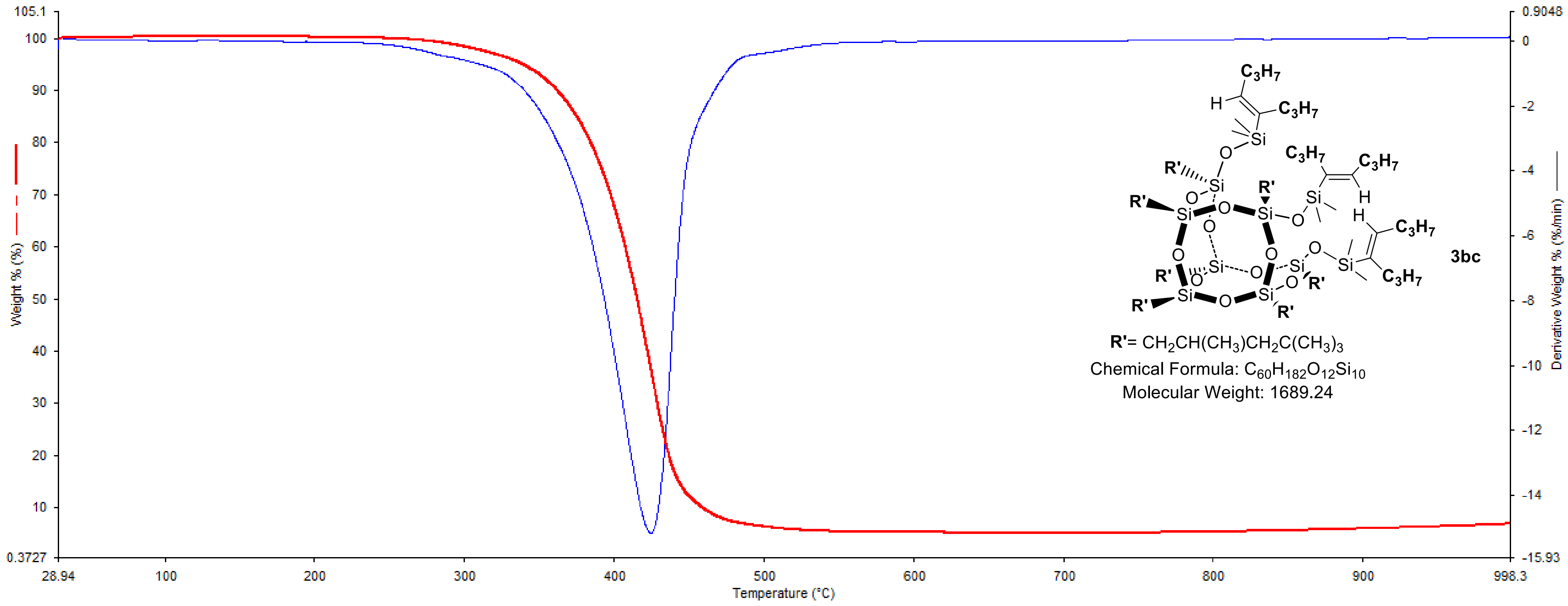

Figure S39. TGA/DTG curves of compound **3bc**.

3ad

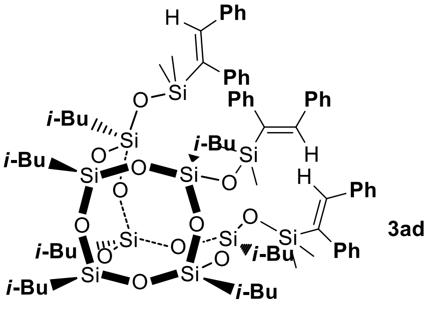

Chemical Formula: $C_{76}H_{114}O_{12}Si_{10}$
Molecular Weight: 1500.57

Isolated yield = 91%, colorless oil.

^{1}H NMR ($CDCl_3$, 300 MHz, δ, ppm): 0.26 (s, 18H, SiC<u>H</u>$_3$), 0.63-0.67 (m, 14H, C<u>H</u>$_2$), 1.00-1.05 (m, 42H, C<u>H</u>$_3$), 1.88-1.94 (m, 7H, C<u>H</u>), 6.95-7.30 (m, 33H, =C<u>H</u>, C_6H_5). **^{13}C NMR** ($CDCl_3$, 75 MHz, δ, ppm): 0.37 (Si<u>C</u>H_3), 22.60, 23.94, 24.02 (<u>C</u>$H_2CH(CH_3)_2$), 24.18, 25.16, 25.79 (CH_2<u>C</u>$H(CH_3)_2$), 26.07, 26.22 ($CH_2CH($<u>C</u>$H_3)_2$), 125.75–138.19 (<u>C</u>$_6H_5$), 141.97 (=<u>C</u>$Si(CH_3)_2O$), 145.75 (=<u>C</u>H). **^{29}Si NMR** ($CDCl_3$, 79 MHz, δ, ppm): -67.43, -67.35 (SiO_3), -2.55 ($OSi(CH_3)_2$). **FT IR (cm^{-1}):** 2952.8, 2901.9, 2868.7, 1600.0, 1493.4, 1464.9, 1401.2, 1365.8, 1331.2, 1251.9, 1227.4, 1070.8, 1045.3, 957.7, 901.8, 831.8, 782.0, 753.4, 691.4, 593.6, 539.3, 444.1. **MALDI TOF MS -** (m/z) ([M+Na], (%)): 1522.60.

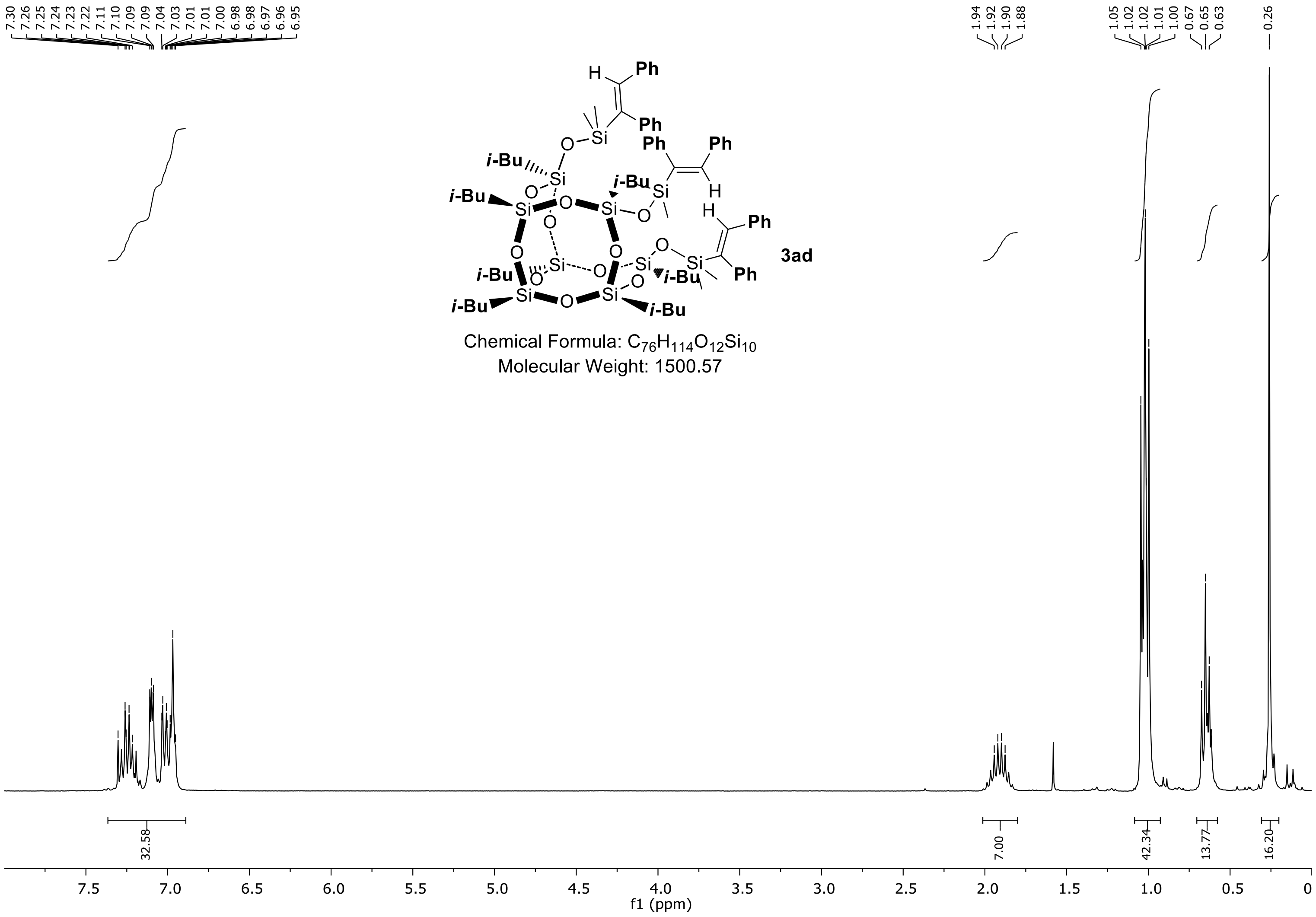

Figure S40. ^{1}H NMR of compound **3ad**.

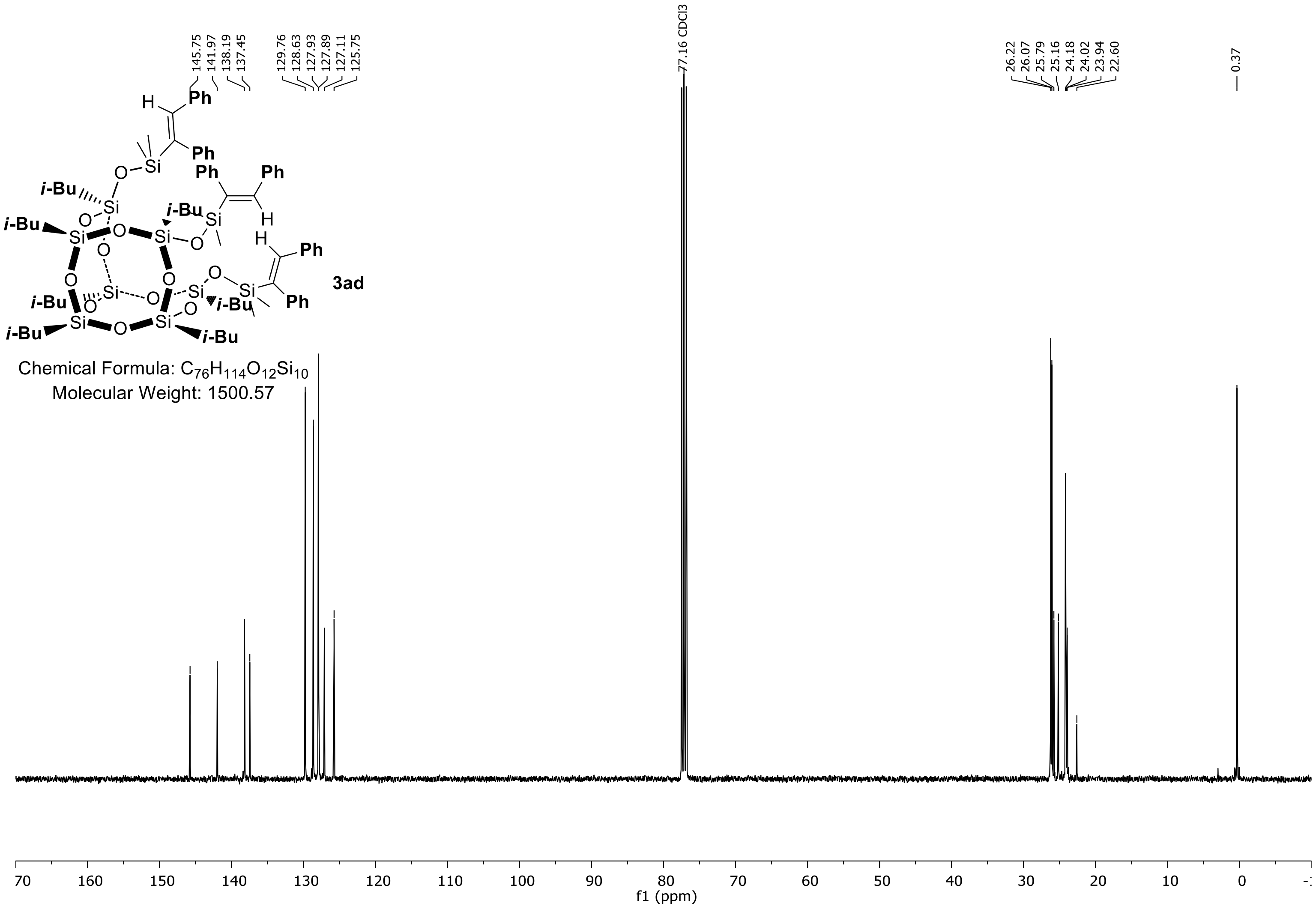

Figure S41. ^{13}C NMR of compound **3ad**.

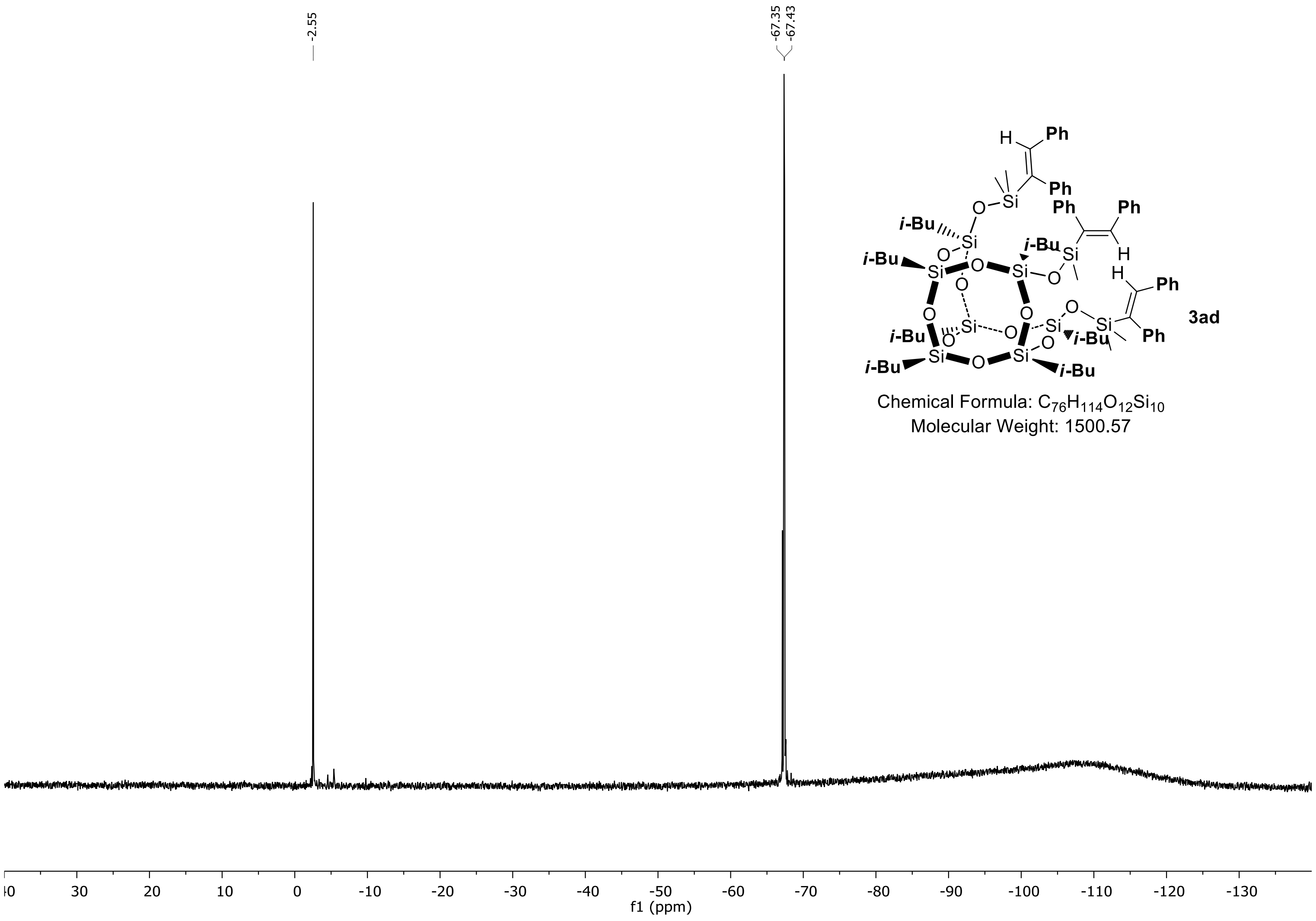

Figure S42. ^{29}Si NMR of compound **3ad**.

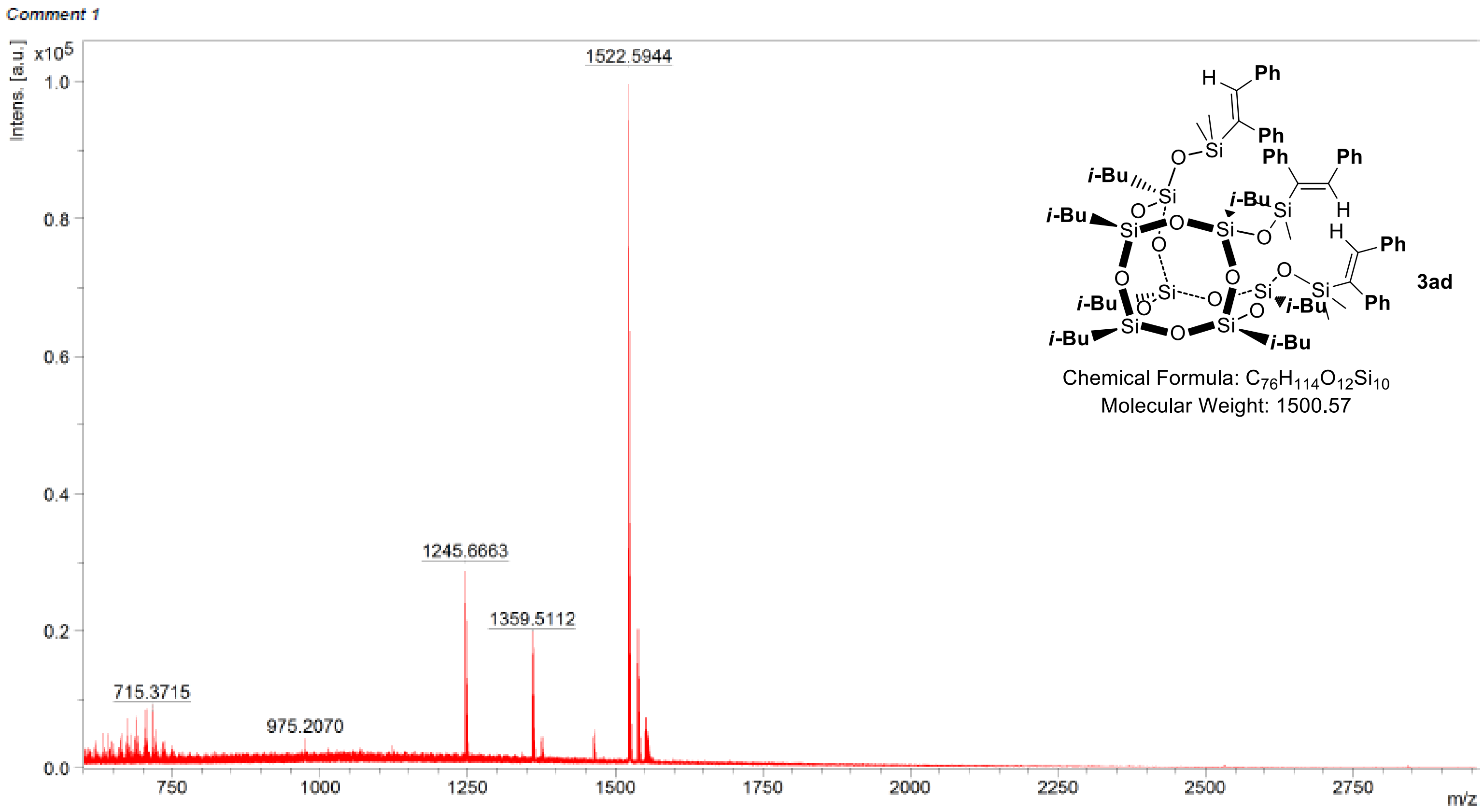

Figure S43. MALDI TOF MS spectra of compound **3ad**.

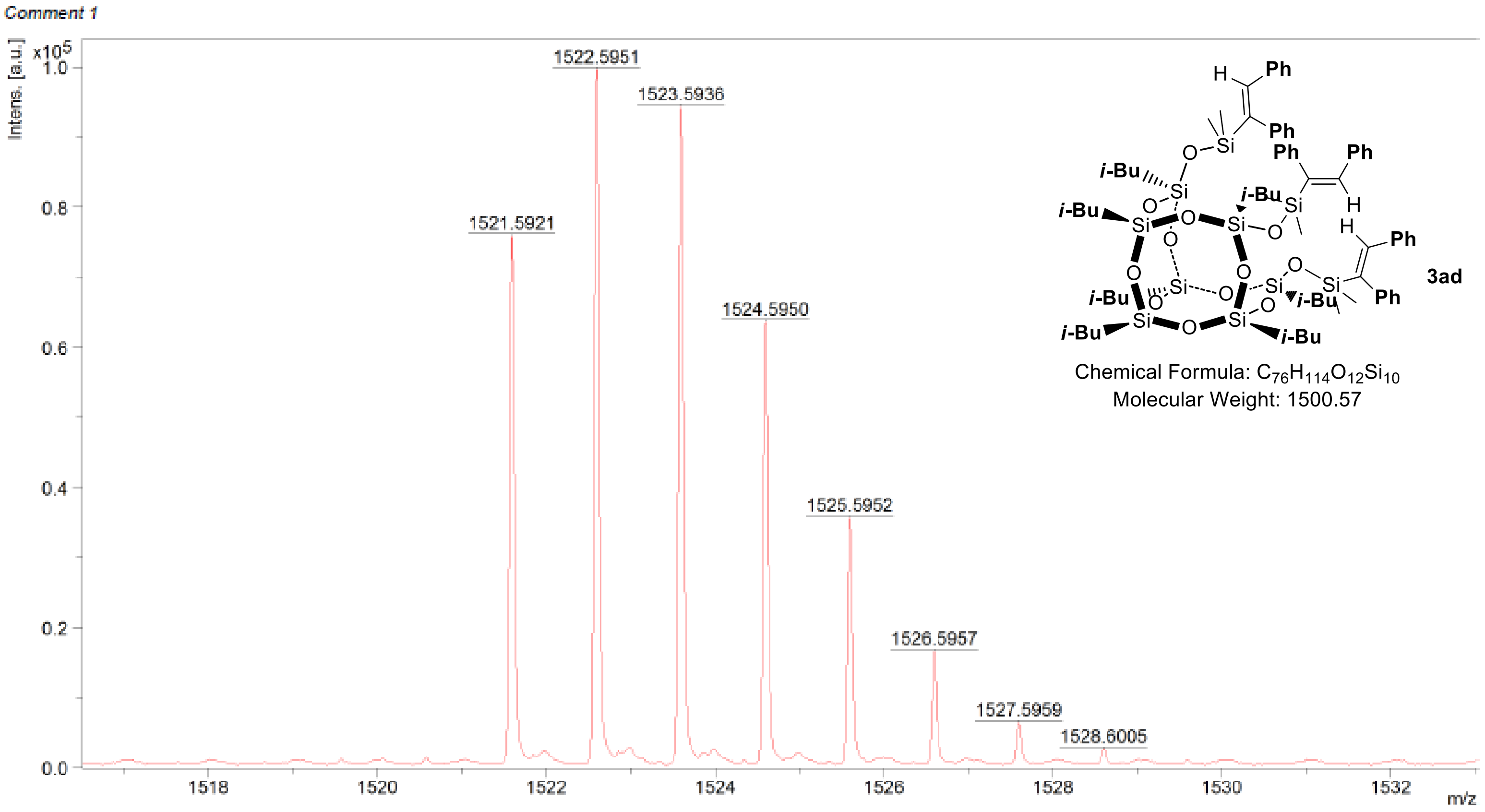

Figure S44. MALDI TOF MS spectra of compound **3ad**.

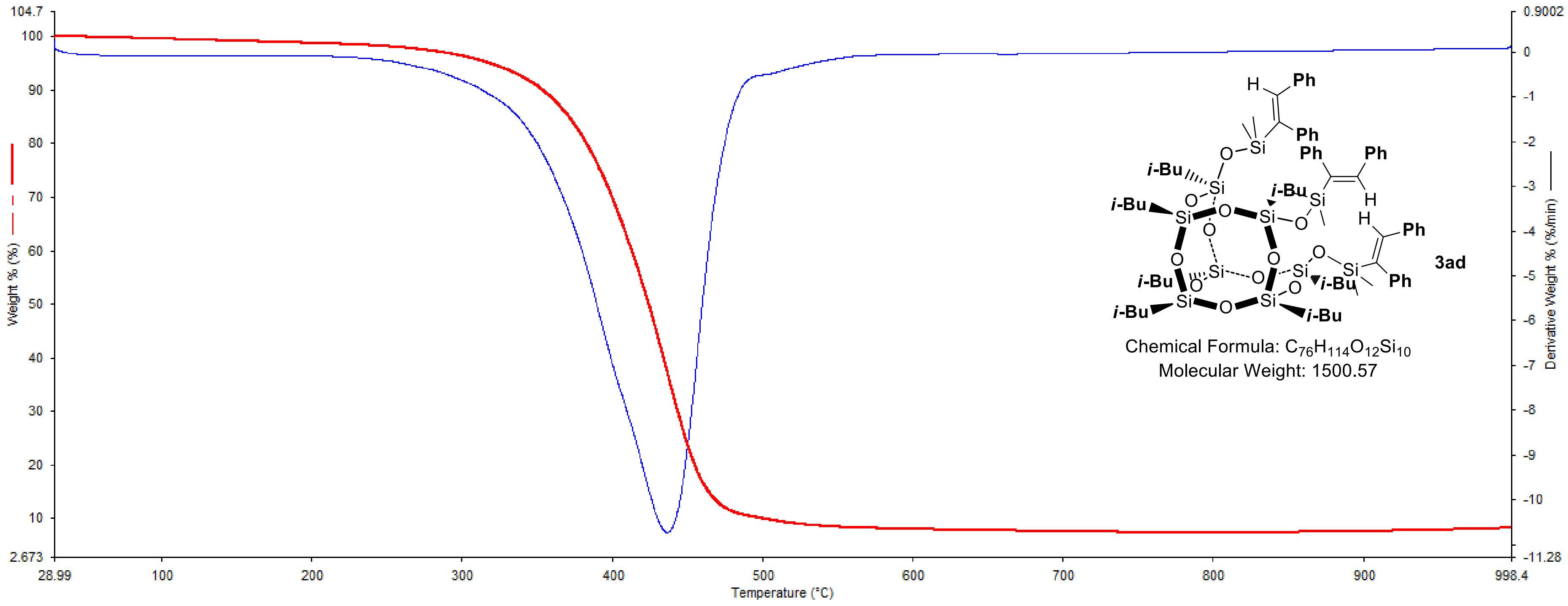

Figure S45. TGA/DTG curves of compound **3ad**.

3bd

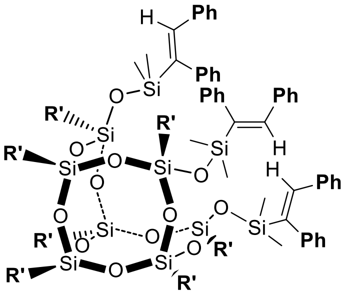

3bd

R'= $CH_2CH(CH_3)CH_2C(CH_3)_3$
Chemical Formula: $C_{78}H_{170}O_{12}Si_{10}$
Molecular Weight: 1893.34

Isolated yield = 87%, colorless oil.

^{1}H NMR ($CDCl_3$, 300 MHz, δ, ppm): 0.25 (s, 18H, $SiC\underline{H}_3$), 0.58-0.66 (m, 14H, $SiC\underline{H}_2$), 0.93-0.94 (m, 63H, $C(C\underline{H}_3)_3$), 1.04-1.07 (m, 21H, $CH(C\underline{H}_3)$), 1.17-1.25 (m, 7H, $C\underline{H}_2$), 1.27-1.38 (m, 7H, $C\underline{H}_2$), 1.88-1.91 (m, 7H, $C\underline{H}(CH_3)$), 6.92–7.29 (m, 33H, =$C\underline{H}$, $C_6\underline{H}_5$). **^{13}C NMR** ($CDCl_3$, 75 MHz, δ, ppm): 0.51, 1.18 ($OSi\underline{C}H_3$), 23.50, 25.06, 25.31, 25.35, 25.56, 25.89, 26.58 ($\underline{C}H$, $Si\underline{C}H_2$, $CH(\underline{C}H_3)$), 30.30, 30.44 ($(\underline{C}H_3)_3$), 31.33 ($\underline{C}(CH_3)_3$), 55.21, 55.35 ($\underline{C}H_2C(CH_3)_3$), 125.75-138.28 ($\underline{C}_6H_5$), 141.97 (=$\underline{C}Si(CH_3)_2O$), 145.61 (=$\underline{C}H$). **^{29}Si NMR** ($CDCl_3$, 79 MHz, δ, ppm): -68.19, -67.88, -67.71, -67.60, -67.44, -67.36 (SiO_3), -2.55, -2.62, -2.70 ($OSi(CH_3)_2$). **FT IR (cm^{-1}):** 2952.0, 2904.9, 2867.9, 1601.8, 1475.8, 1392.9, 1363.8, 1250.7, 1225.2, 1070.0, 1043.2, 958.2, 903.2, 831.8, 782.6, 754.2, 691.1, 593.6, 479.4, 429.1.

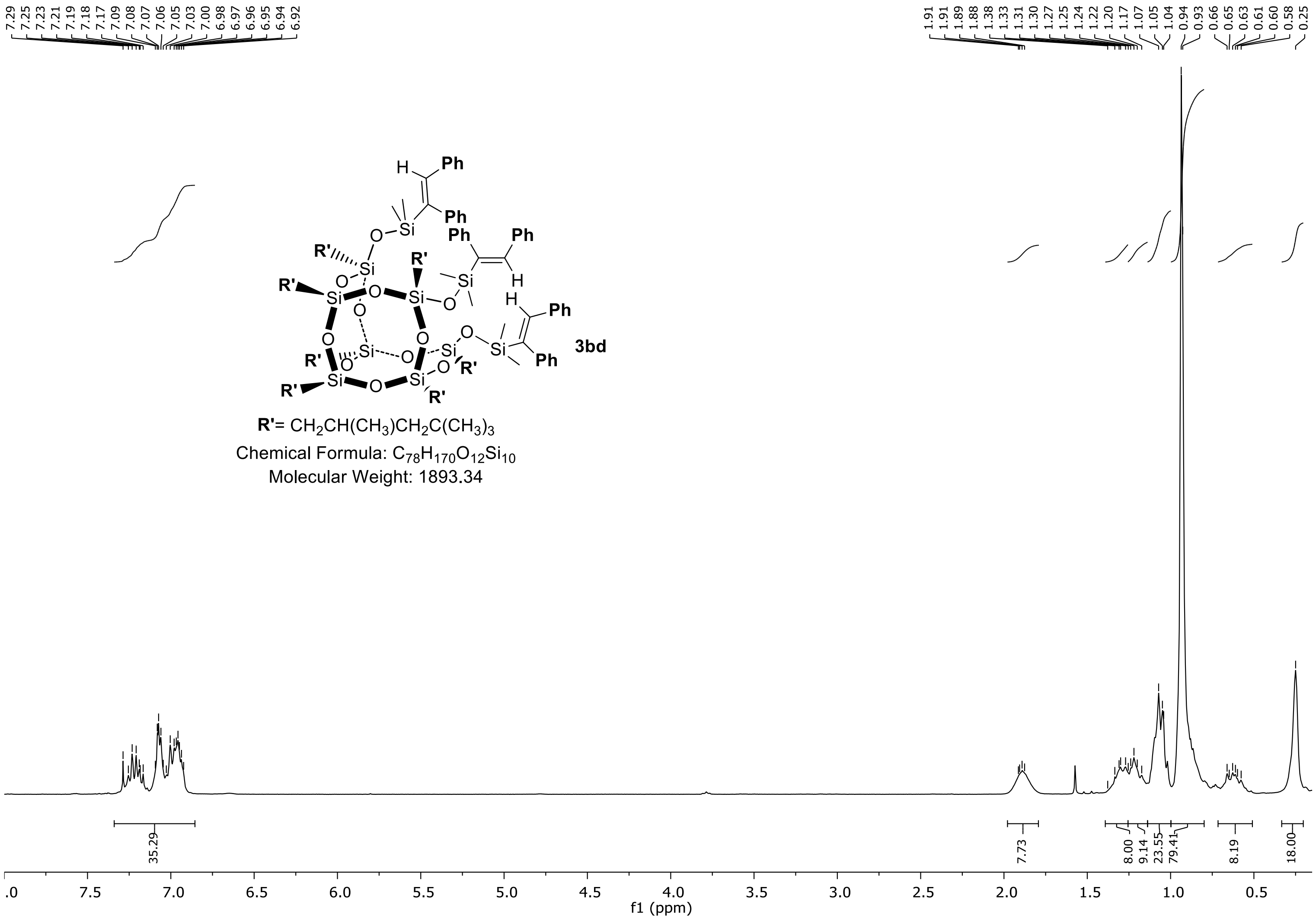

Figure S46. ^{1}H NMR of compound **3bd**.

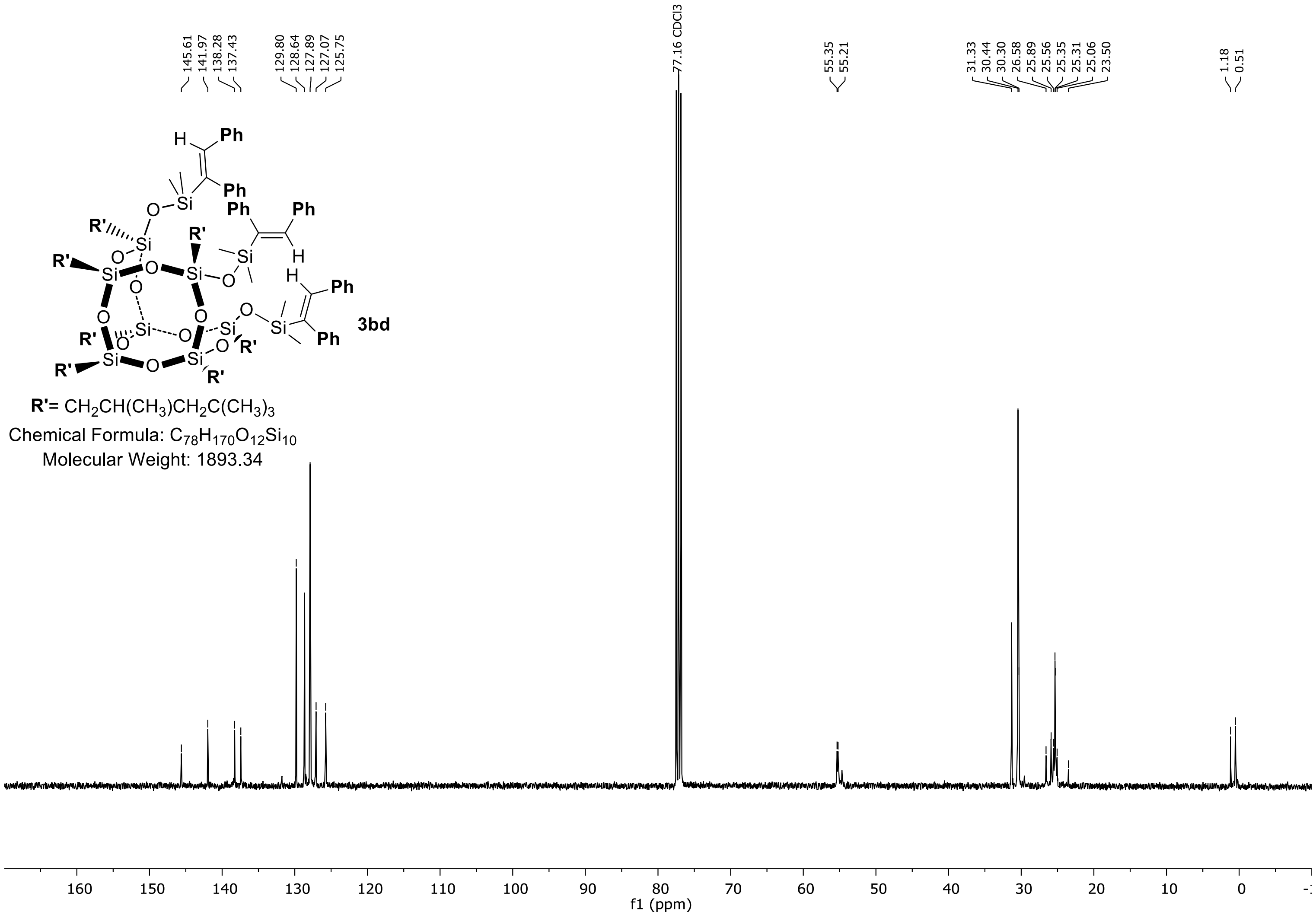

Figure S47. ^{13}C NMR of compound **3bd**.

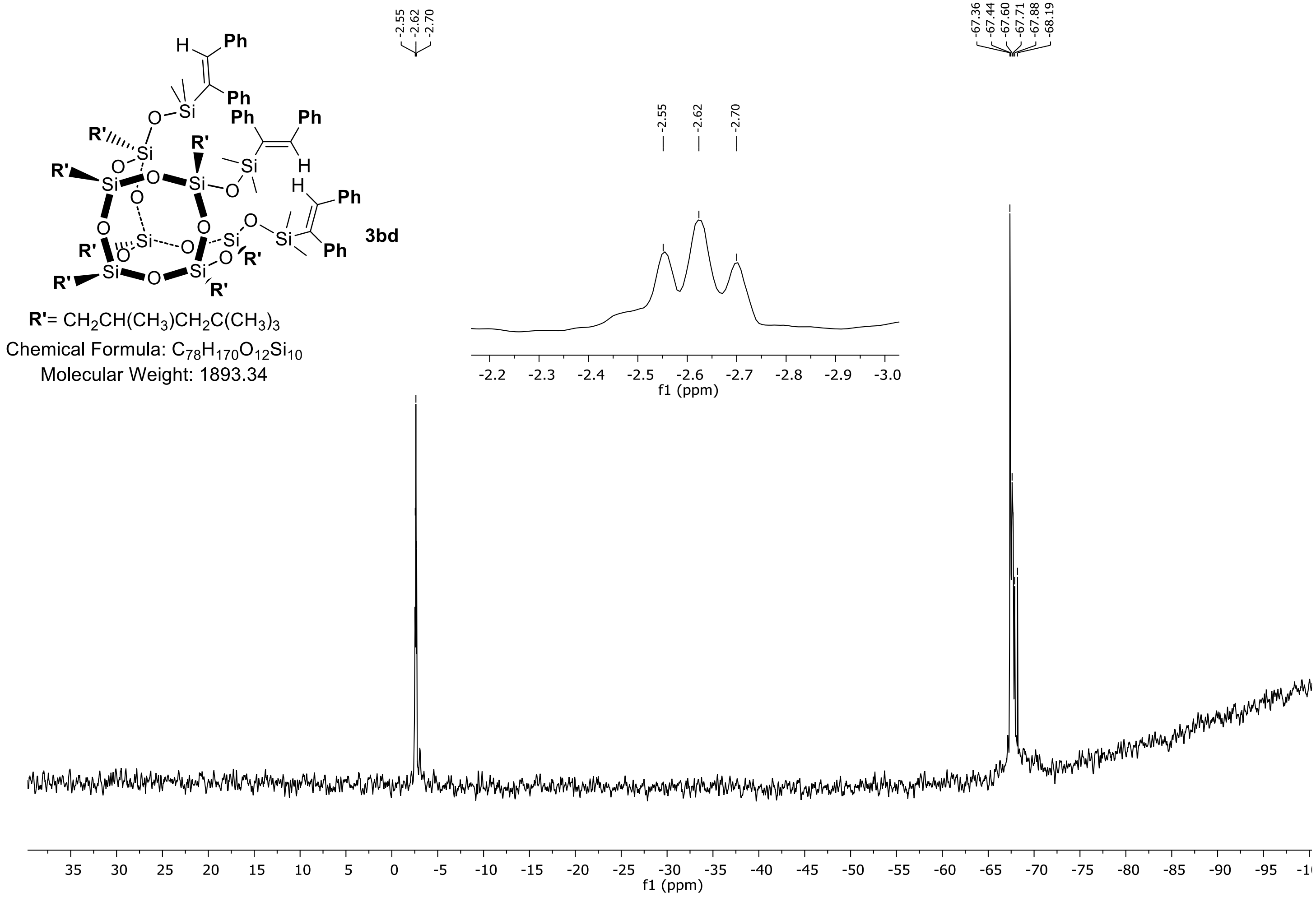

Figure S48. ^{29}Si NMR of compound **3bd**.

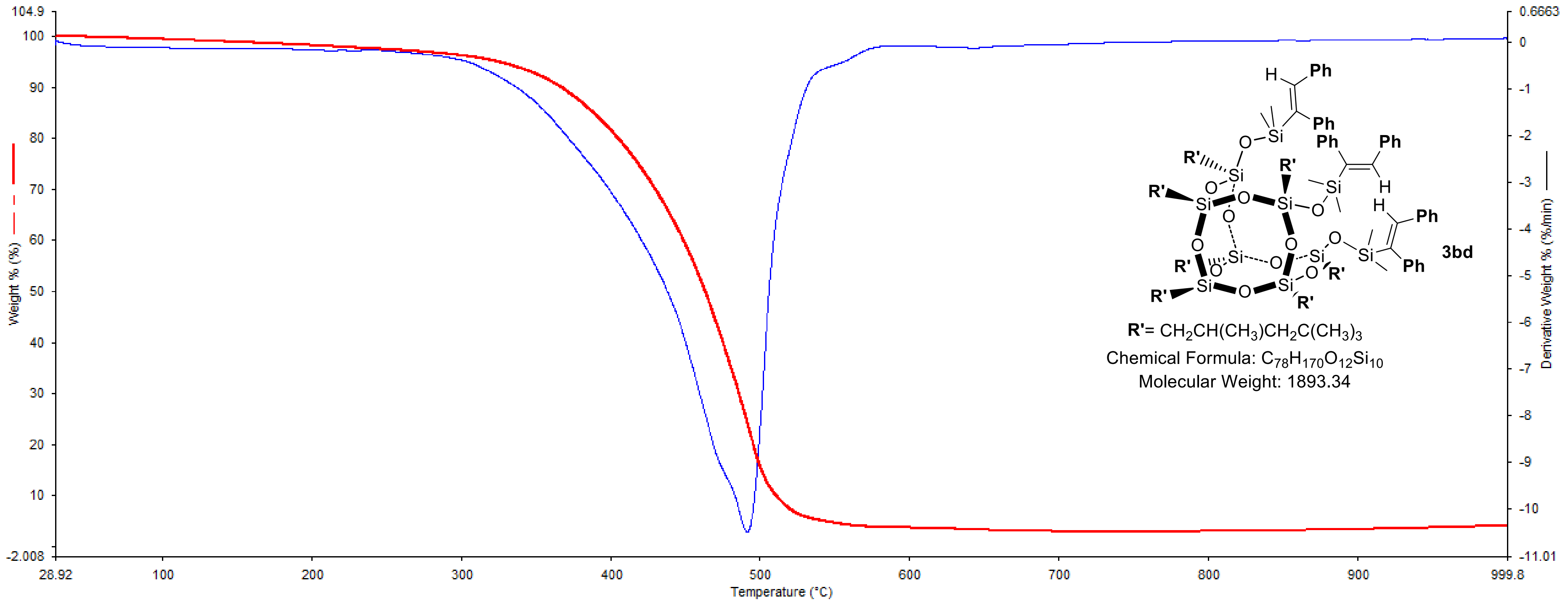

Figure S49. TGA/DTG curves of compound **3bd**.

3ae

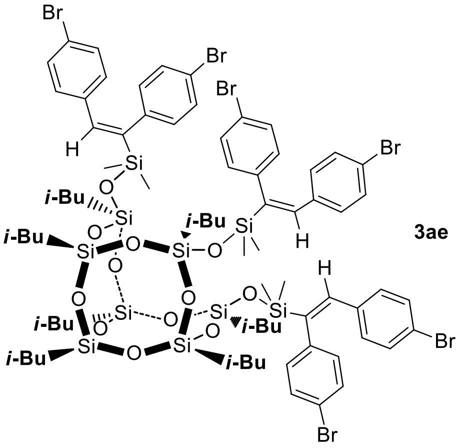

3ae

Chemical Formula: $C_{76}H_{108}Br_6O_{12}Si_{10}$
Molecular Weight: 1973.94

Isolated yield = 89%, colorless oil..

^{1}H NMR ($CDCl_3$, 300 MHz, δ, ppm): 0.19 (s, 18H, $SiC\underline{H}_3$), 0.56-0.63 (m, 14H, $C\underline{H}_2$), 0.94-1.00 (m, 42H, $C\underline{H}_3$), 1.79-1.89 (m, 7H, $C\underline{H}$), 6.75 (d, 6H, $J_{(H,H)}$= 8.55 Hz, $C_6\underline{H}_4Br$), 6.79 (s, 3H, =$C\underline{H}$), 6.80 (d, 6H, $J_{(H,H)}$= 8.35 Hz, $C_6\underline{H}_4Br$), 7.21 (d, 6H, $J_{(H,H)}$= 8.50 Hz, $C_6\underline{H}_4Br$), 7.35 (d, 6H, $J_{(H,H)}$= 8.39 Hz, $C_6\underline{H}_4Br$). **^{13}C NMR** ($CDCl_3$, 75 MHz, δ, ppm): 0.30 ($Si\underline{C}H_3$), 22.50, 23.87, 23.99 ($\underline{C}H_2CH(CH_3)_2$), 24.18, 25.09, 25.76 ($CH_2\underline{C}H(CH_3)_2$), 25.80, 26.04, 26.06, 26.18 ($CH_2CH(\underline{C}H_3)_2$), 120.13 – 137.50 ($\underline{C}_6H_4Br$), 140.35 (=$\underline{C}Si(CH_3)_2O$), 145.36 (=$\underline{C}H$). **^{29}Si NMR** ($CDCl_3$, 79 MHz, δ, ppm): -67.90, -67.23, -67.19, -67.08 (SiO_3), -2.75 ($OSi(CH_3)_2$). **FT IR (cm^{-1}):** 2952.7, 2901.8, 2868.6, 1583.9, 1483.8, 1464.8, 1400.7, 1365.5, 1331.2, 1252.5, 1227.8, 1068.9, 1032.1, 961.1, 933.8, 833.5, 814.3, 781.2, 746.2, 640.6, 589.8, 537.3, 444.0. **MALDI TOF MS -** (m/z) ([M+Na], (%)): 1997.07.

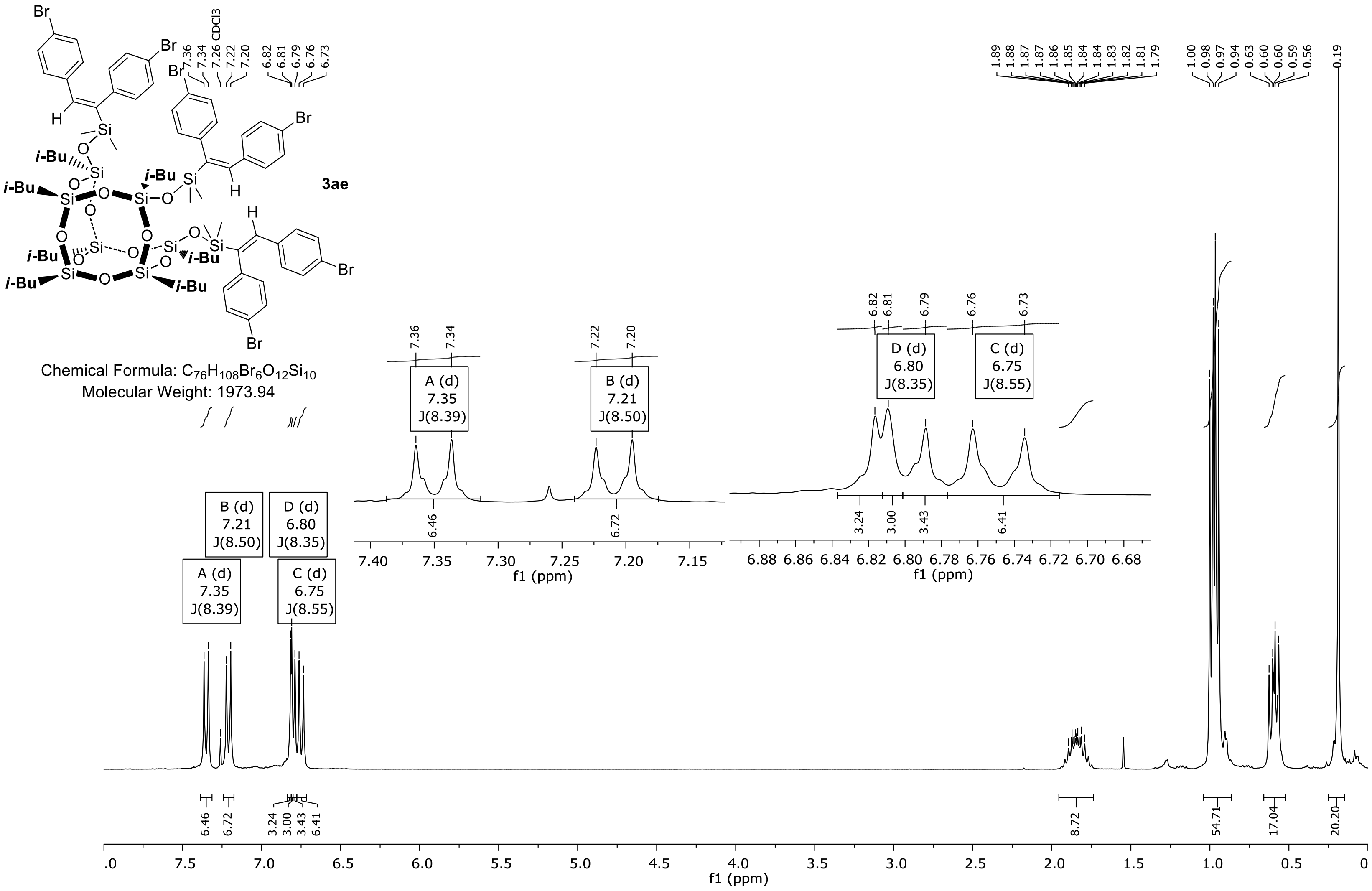

Figure S50. ^{1}H NMR of compound **3ae**.

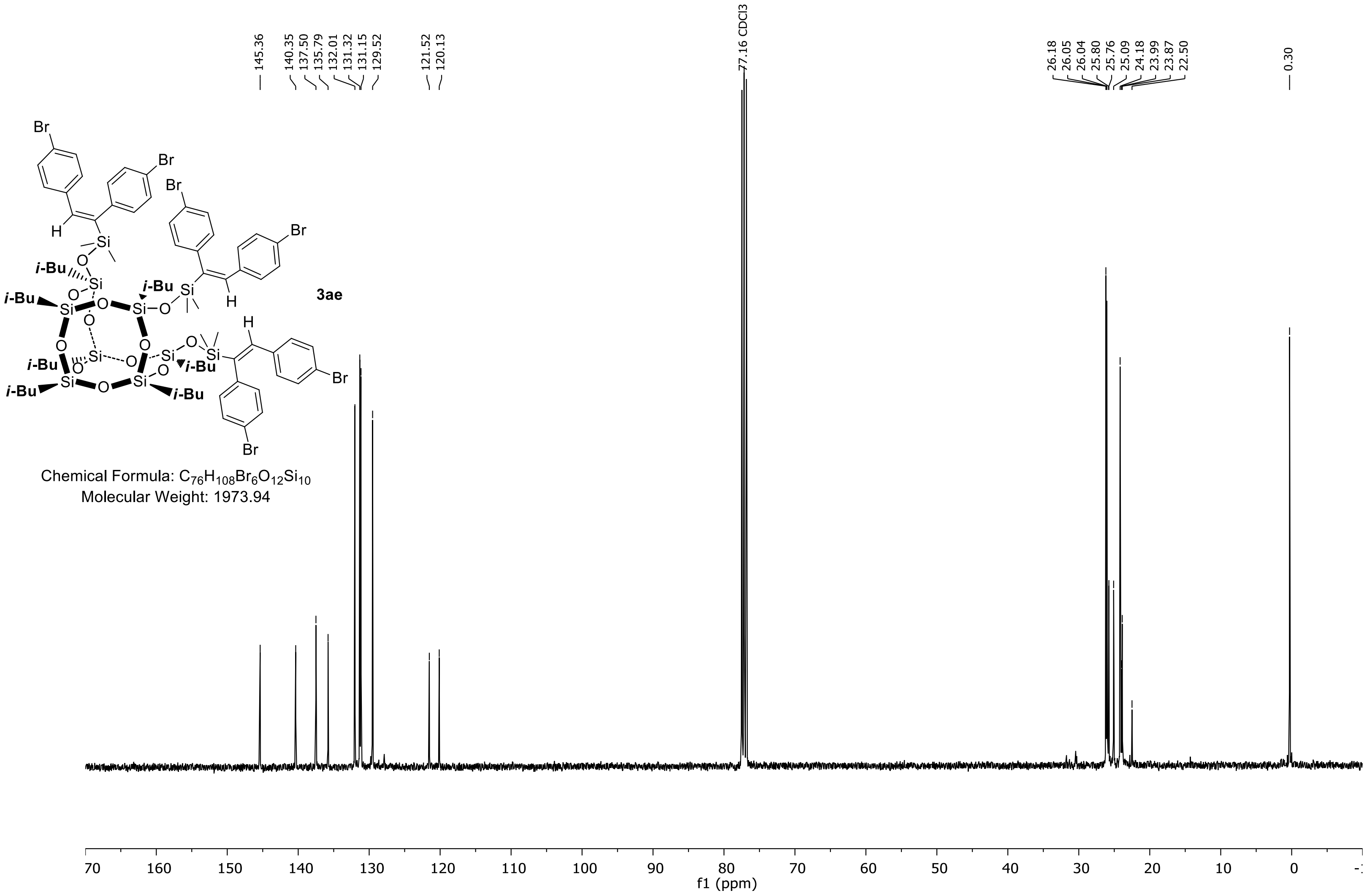

Figure S51. ^{13}C NMR of compound **3ae**.

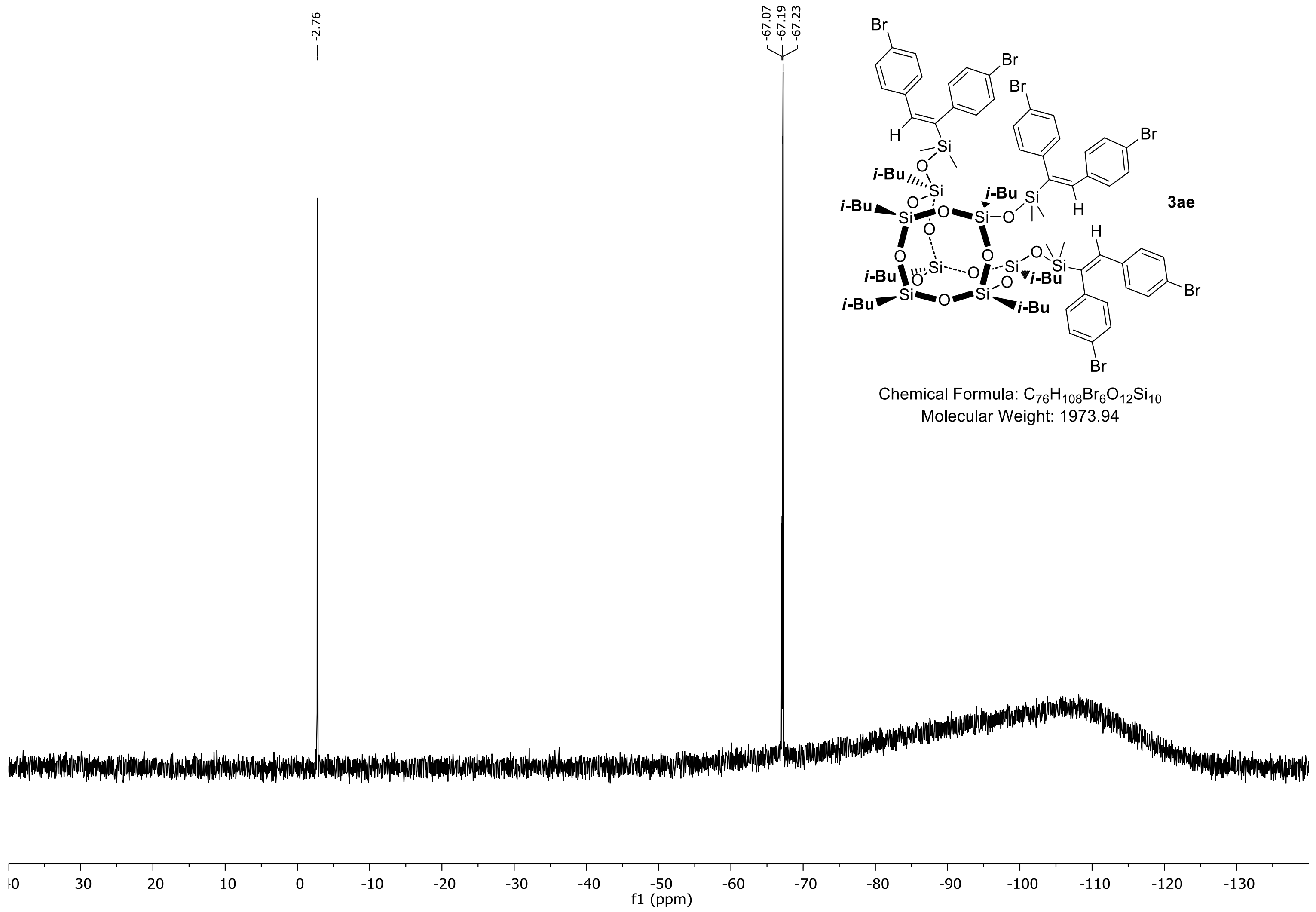

Figure S52. ^{29}Si NMR of compound **3ae**.

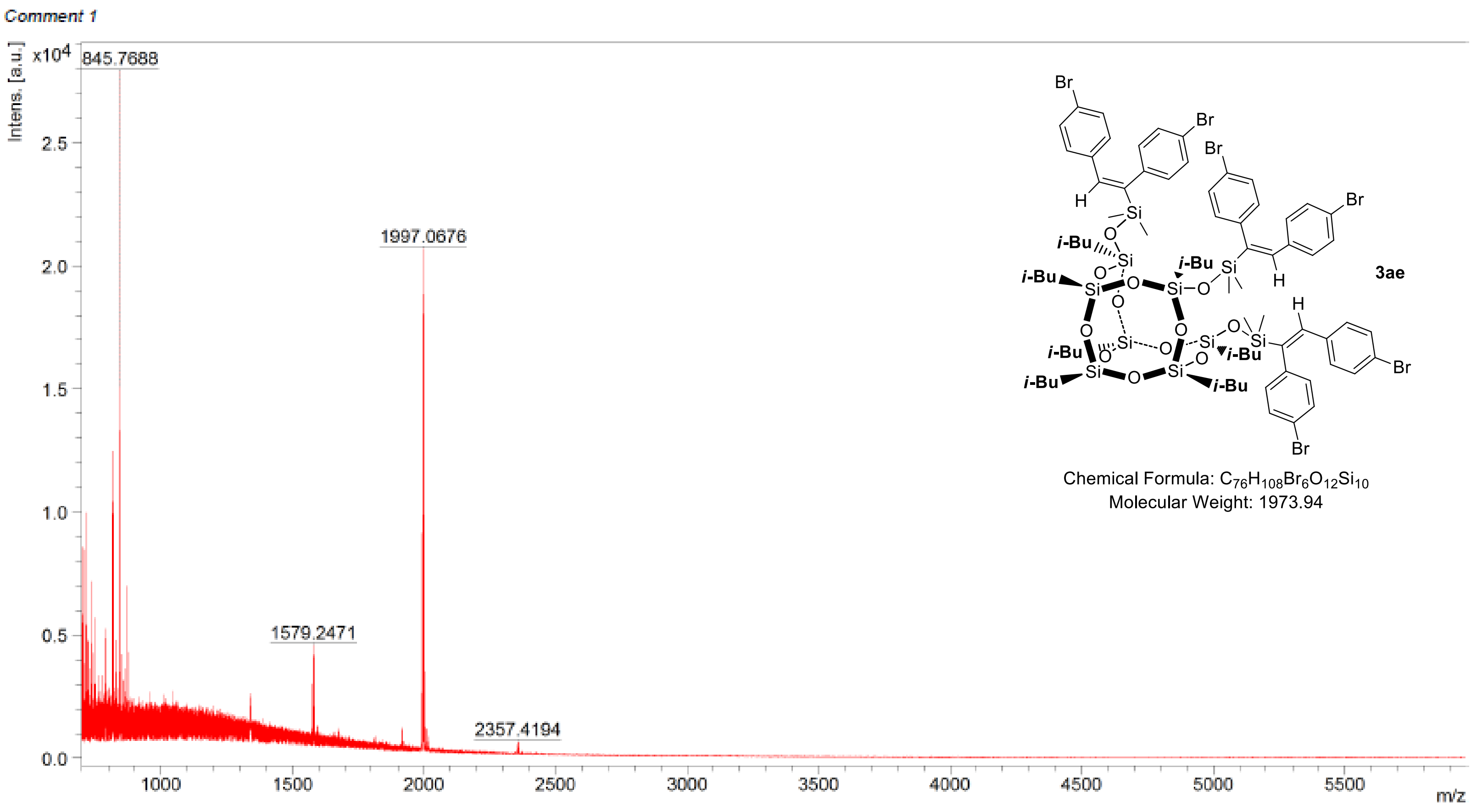

Figure S53. MALDI TOF MS spectra of compound **3ae**.

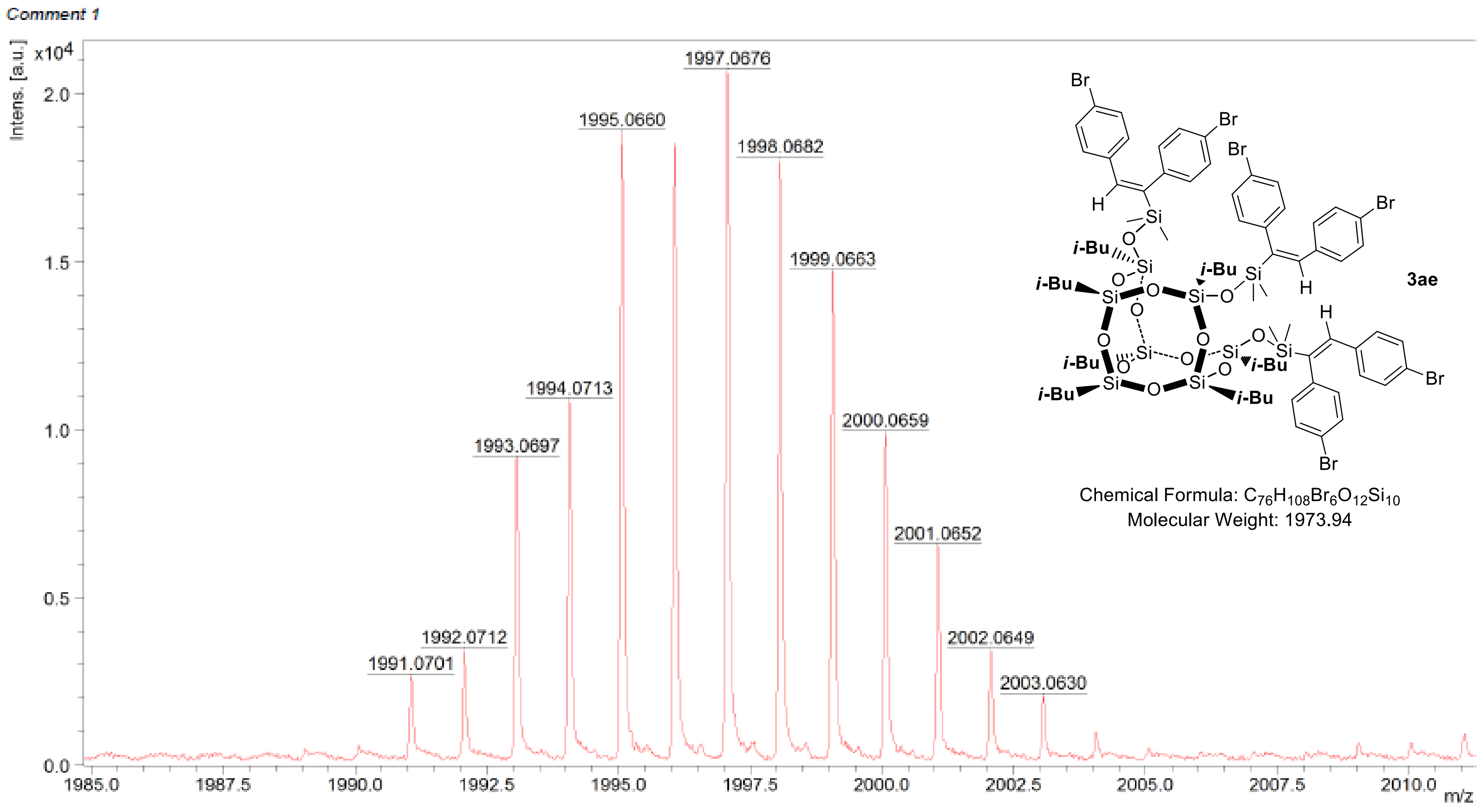

Figure S54. MALDI TOF MS spectra of compound **3ae**.

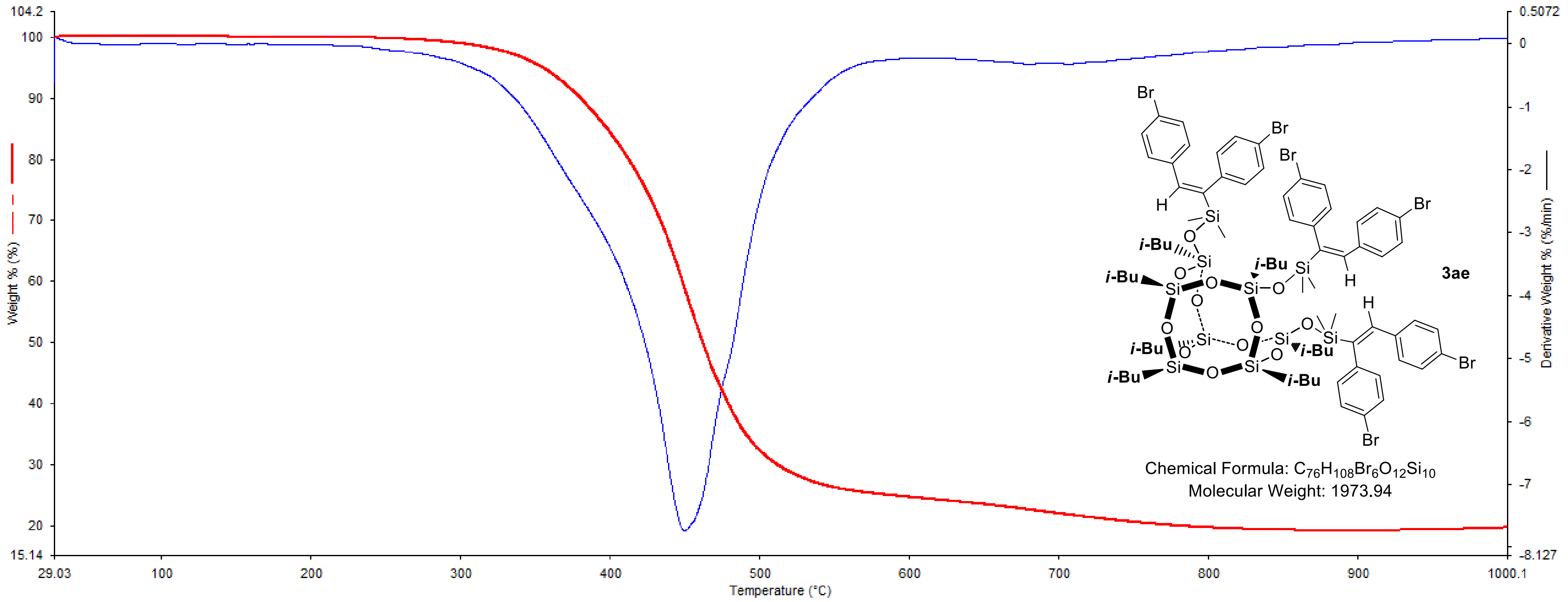

Figure S55. TGA/DTG curves of compound **3ae**.

3be

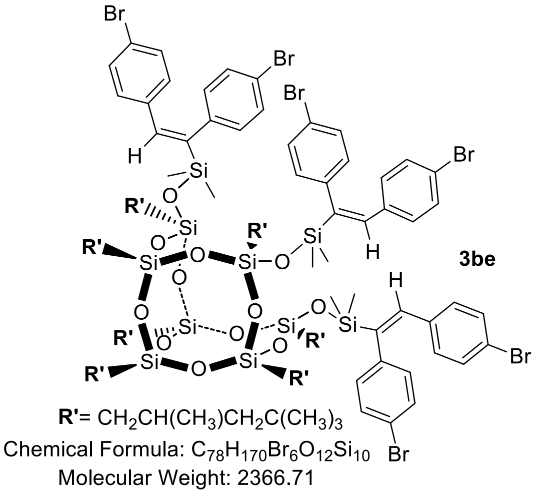

Isolated yield = 84%, colorless oil.

^{1}H NMR ($CDCl_3$, 300 MHz, δ, ppm): 0.18 (s, 18H, SiC<u>H</u>$_3$), 0.51-0.62 (m, 14H, SiC<u>H</u>$_2$), 0.88-0.90 (m, 63H, C(C<u>H</u>$_3$)$_3$), 0.97-1.05 (m, 21H, CH(C<u>H</u>$_3$)), 1.16-1.26 (m, 7H, C<u>H</u>$_2$), 1.27-1.38 (m, 7H, C<u>H</u>$_2$), 1.80-1.83 (m, 7H, C<u>H</u>(CH$_3$)), 6.72 (d, 6H, $J_{(H,H)}$= 8.27 Hz, C$_6$<u>H</u>$_4$Br), 6.77 (s, 3H, =C<u>H</u>), 6.80 (d, 6H, C$_6$<u>H</u>$_4$Br), 7.19 (d, 6H, $J_{(H,H)}$= 8.46 Hz, C$_6$<u>H</u>$_4$Br), 7.33 (d, 6H, $J_{(H,H)}$= 8.15 Hz, C$_6$<u>H</u>$_4$Br). **^{13}C NMR** ($CDCl_3$, 75 MHz, δ, ppm): 0.43 (OSi<u>C</u>H$_3$), 25.04, 25.33 (<u>C</u>H$_2$(CH$_2$)$_4$CH(CH$_3$)$_2$, 25.52, 25.85, 26.50 (<u>C</u>H, Si<u>C</u>H$_2$, CH(<u>C</u>H$_3$)), 30.29, 30.42 ((<u>C</u>H$_3$)$_3$), 31.33 (<u>C</u>(CH$_3$)$_3$), 55.18, 55.29 (<u>C</u>H$_2$C(CH$_3$)$_3$), 120.15–137.59 (<u>C</u>$_6$H$_4$Br), 140.34 (=<u>C</u>Si(CH$_3$)$_2$O), 145.26 (=<u>C</u>H). **^{29}Si NMR** ($CDCl_3$, 79 MHz, δ, ppm): -68.23, -67.43, -67.31, -67.27, -67.06 (SiO_3), -2.87, -2.79, -2.73 (OSi(CH$_3$)$_2$).

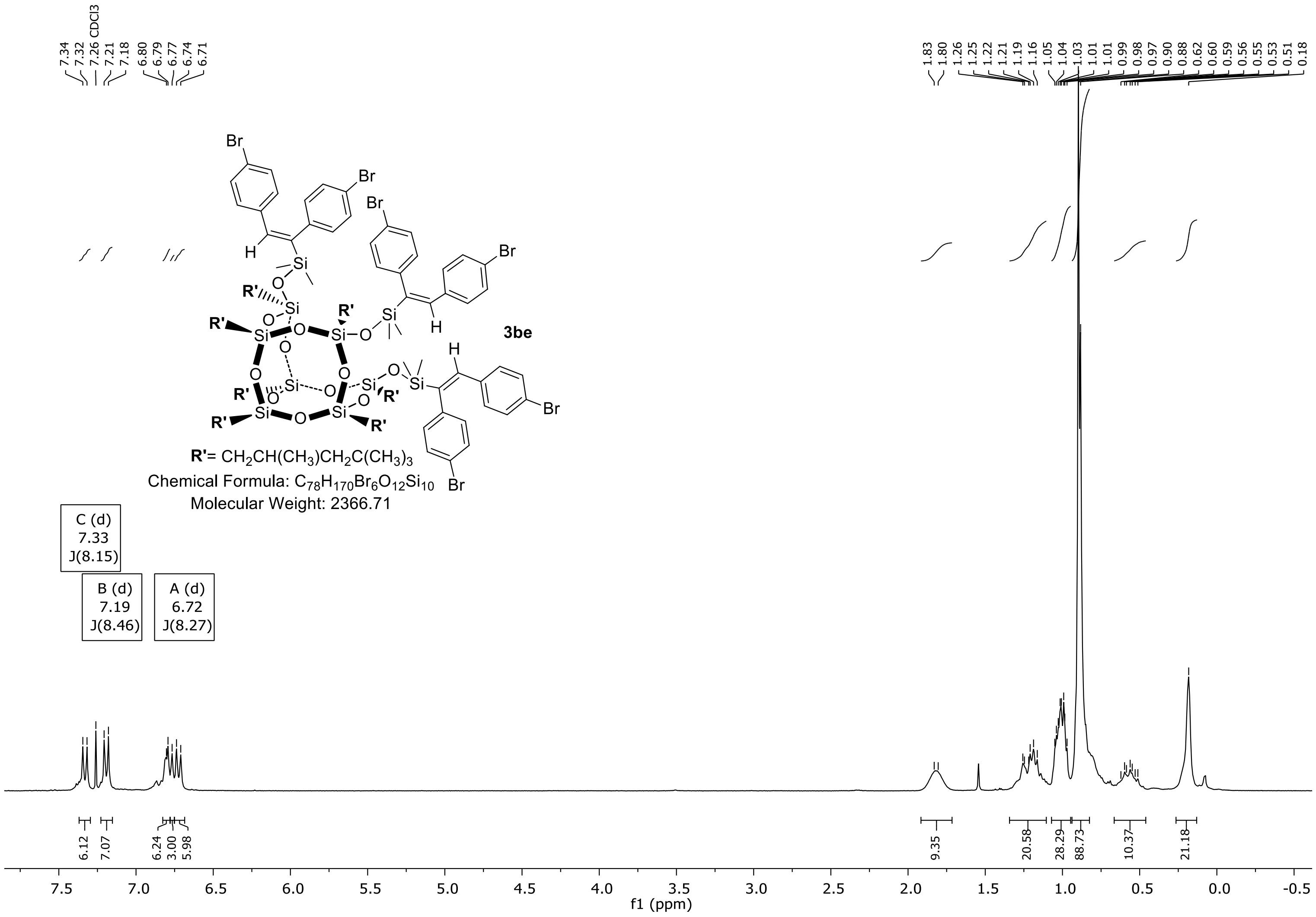

Figure S56. ^{1}H NMR of compound **3be**.

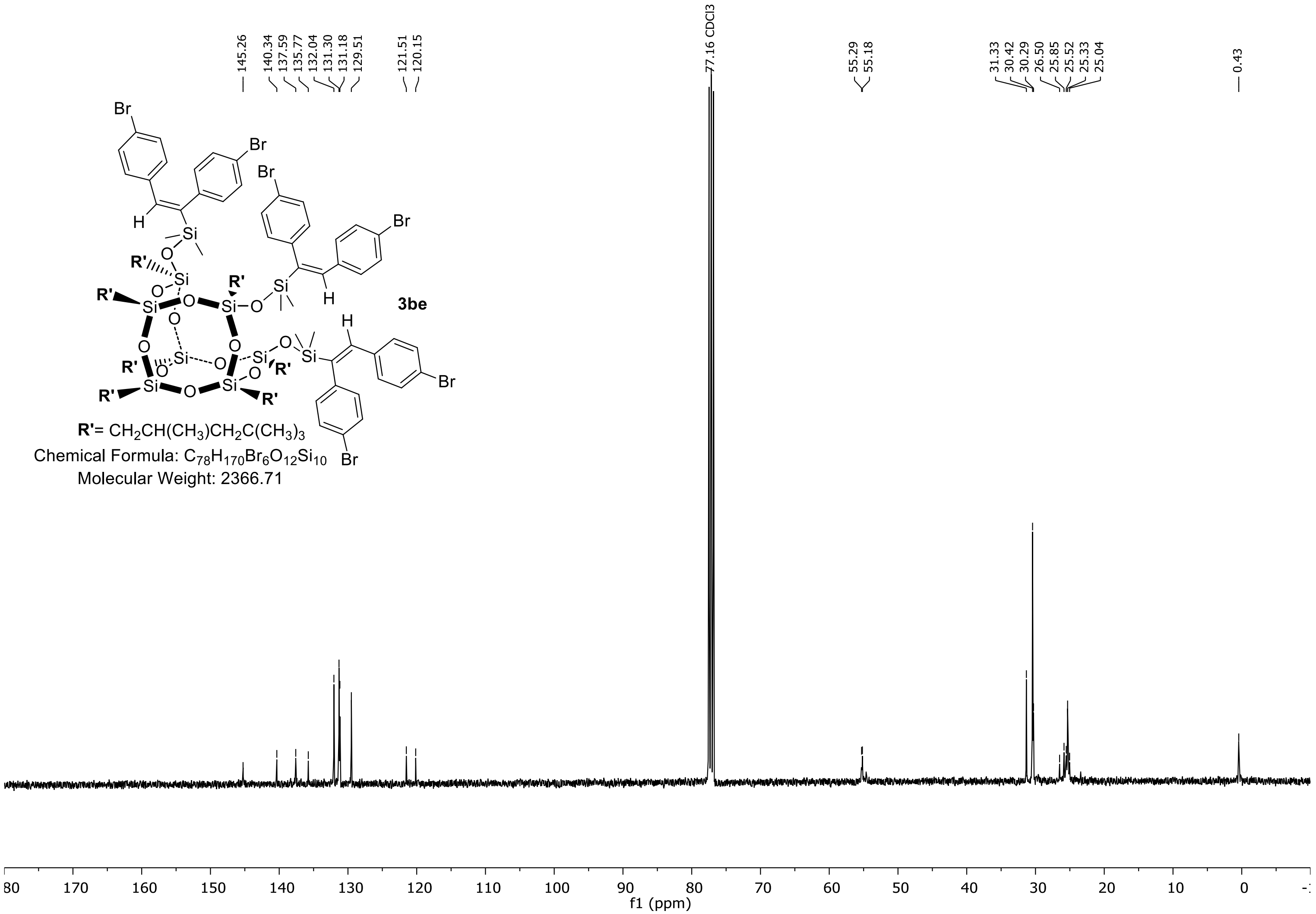

Figure S57. ^{13}C NMR of compound **3be**.

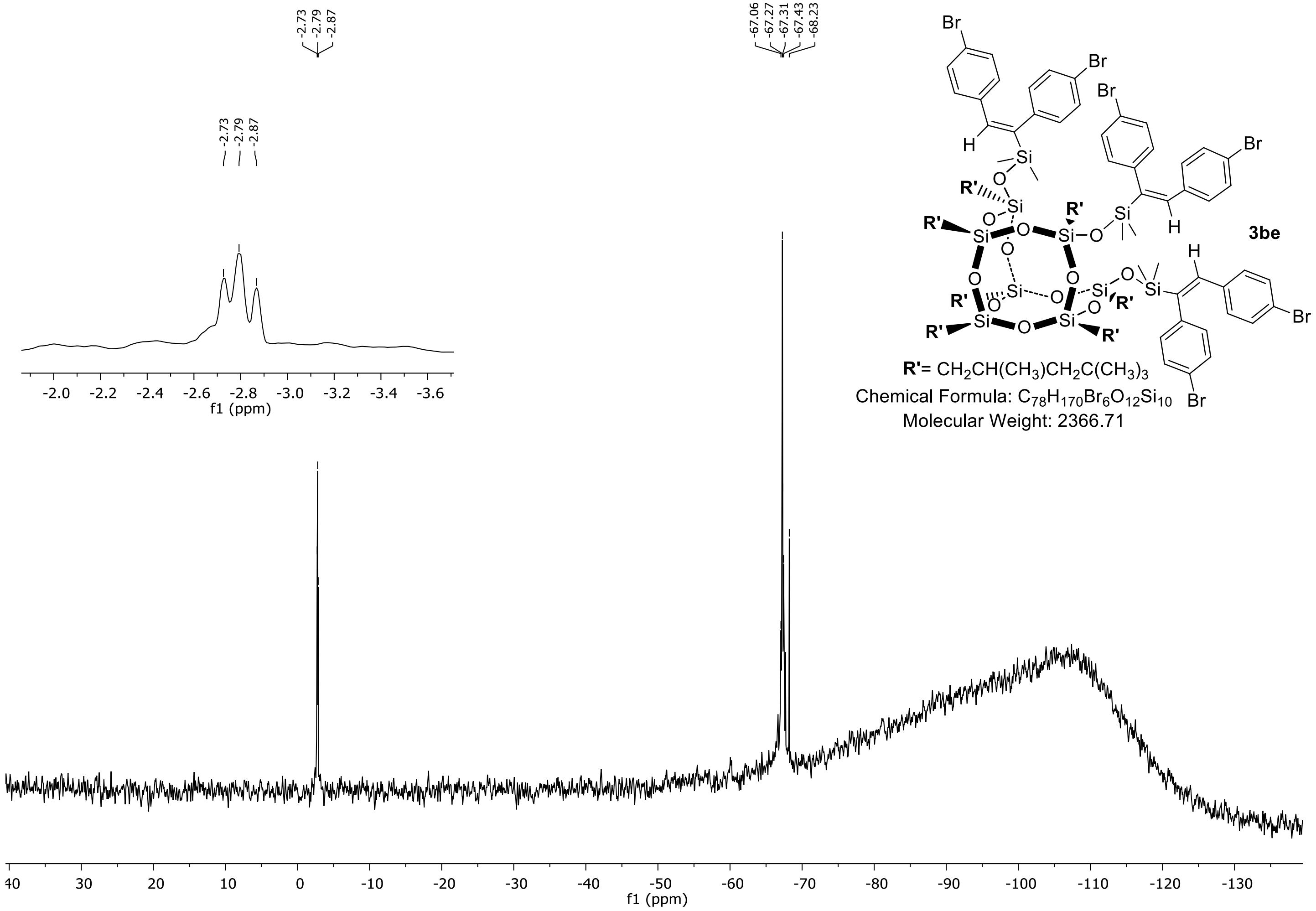

Figure S58. ^{29}Si NMR of compound **3be**.

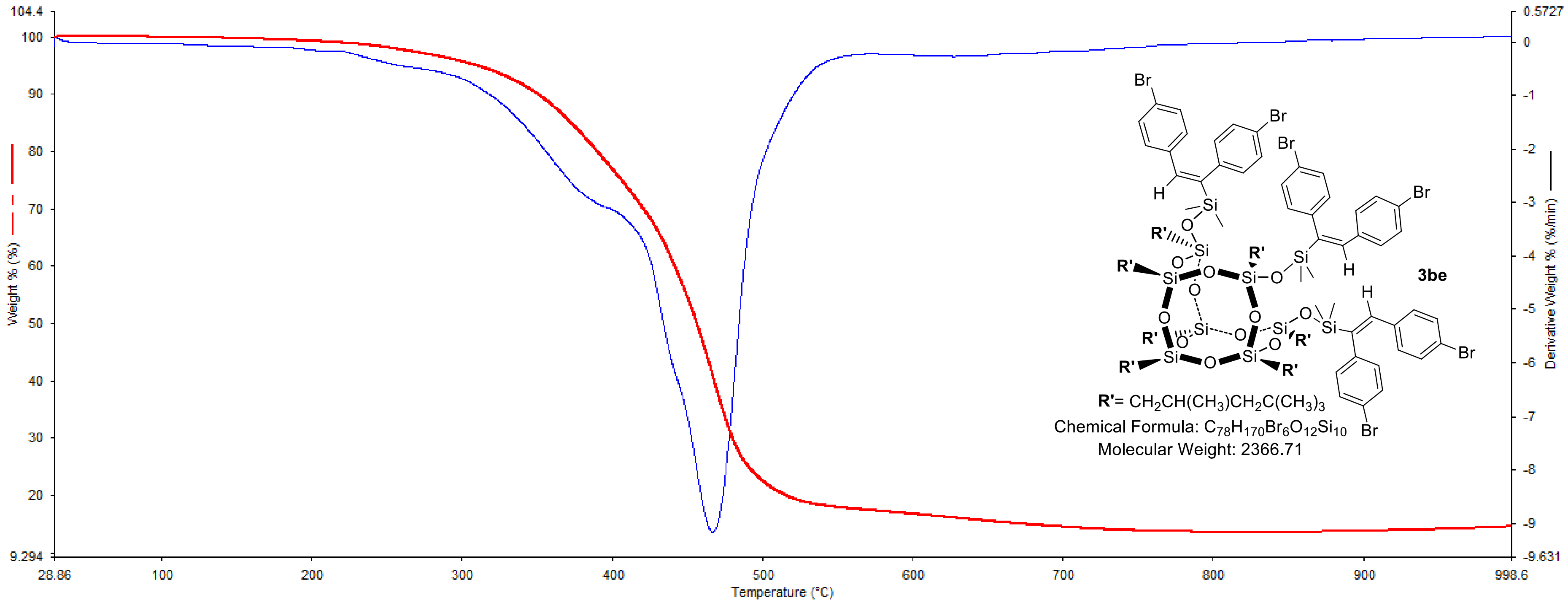

Figure S59. TGA/DTG curves of compound **3be**.

3af

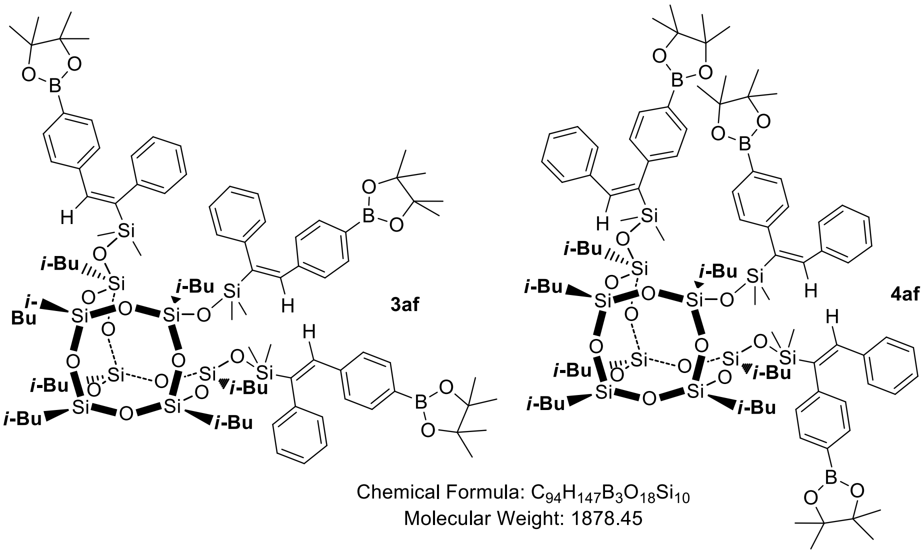

3af 4af

Chemical Formula: $C_{94}H_{147}B_3O_{18}Si_{10}$
Molecular Weight: 1878.45

Isolated yield = 90%, colorless oil.

^{1}**H NMR** ($CDCl_3$, 300 MHz, δ, ppm): 0.19-0.21 (m, 18H, $SiC\underline{H}_3$), 0.54-0.61 (m, 14H, $C\underline{H}_2$), 0.93-0.98 (m, 42H, $C\underline{H}_3$), 1.29 (s, 16H, $C(C\underline{H}_3)_2$), 1.35 (s, 20H, $C(C\underline{H}_3)_2$), 1.79-1.90 (m, 7H, $C\underline{H}$), 6.89-7.80 (m, 30H, $C_6\underline{H}_5$, $=C\underline{H}$, $C_6\underline{H}_4$). ^{13}**C NMR** ($CDCl_3$, 75 MHz, δ, ppm): 0.39 ($Si\underline{C}H_3$), 22.59, 23.92, 24.00 ($\underline{C}H_2CH(CH_3)_2$), 24.16 ($CH_2\underline{C}H(CH_3)_2$), 25.00, 25.08 ($C(\underline{C}H_3)_2$), 25.78, 26.05, 26.07, 26.21 ($CH_2CH(\underline{C}H_3)_2$), 83.74 ($\underline{C}(CH_3)_2$), 125.79 – 145.49 ($\underline{C}_6H_5$,$\underline{C}_6H_4BO_2(C(CH_3)_2)_2$), 145.73 ($=\underline{C}Si(CH_3)_2O$), 147.05 ($=\underline{C}H$). ^{29}**Si NMR** ($CDCl_3$, 79 MHz, δ, ppm): -67.47, -67.38, -67.35 (SiO_3), -2.53 ($OSi(CH_3)_2$).

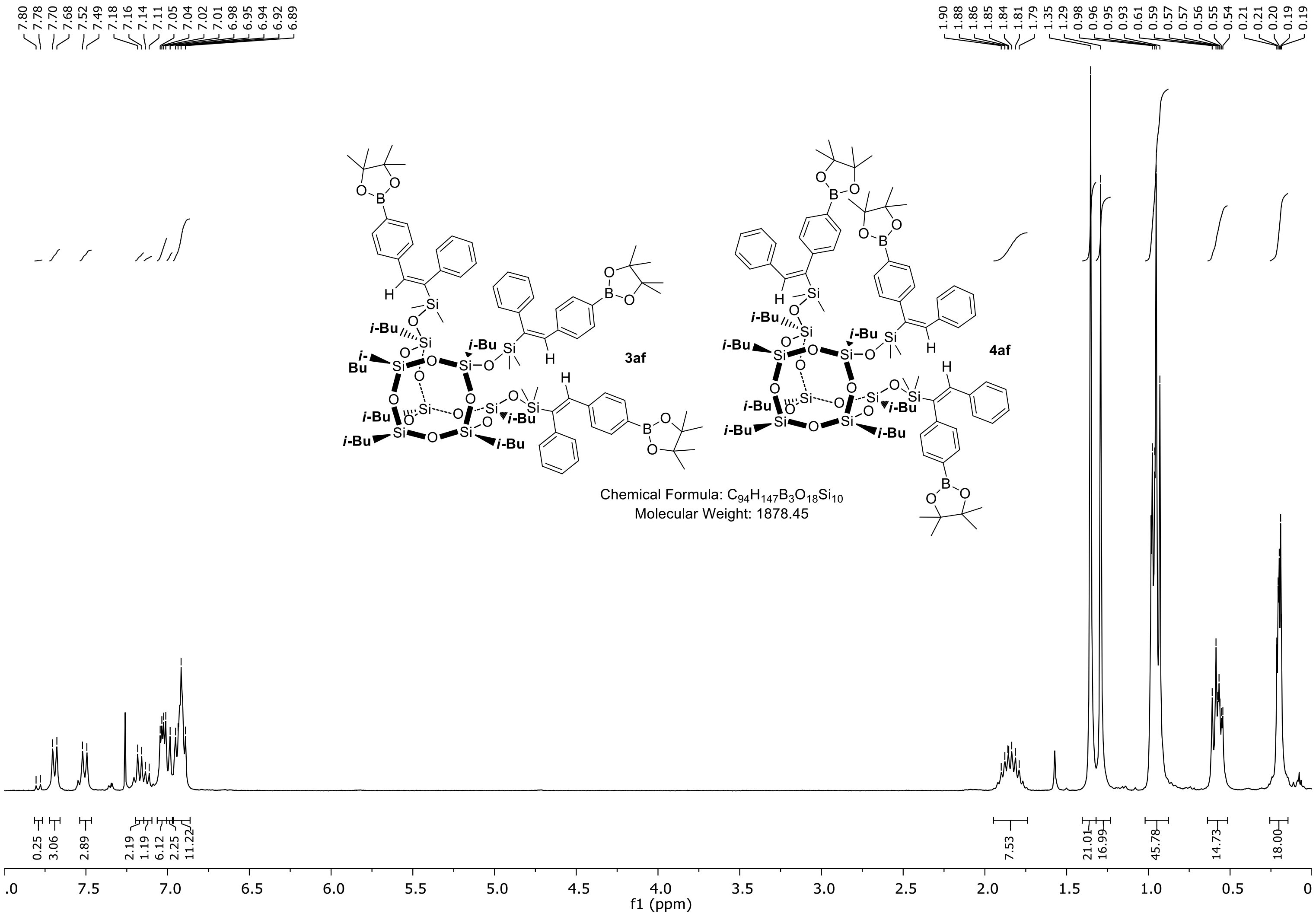

Figure S60. ^{1}H NMR of **3af/4af** mixture.

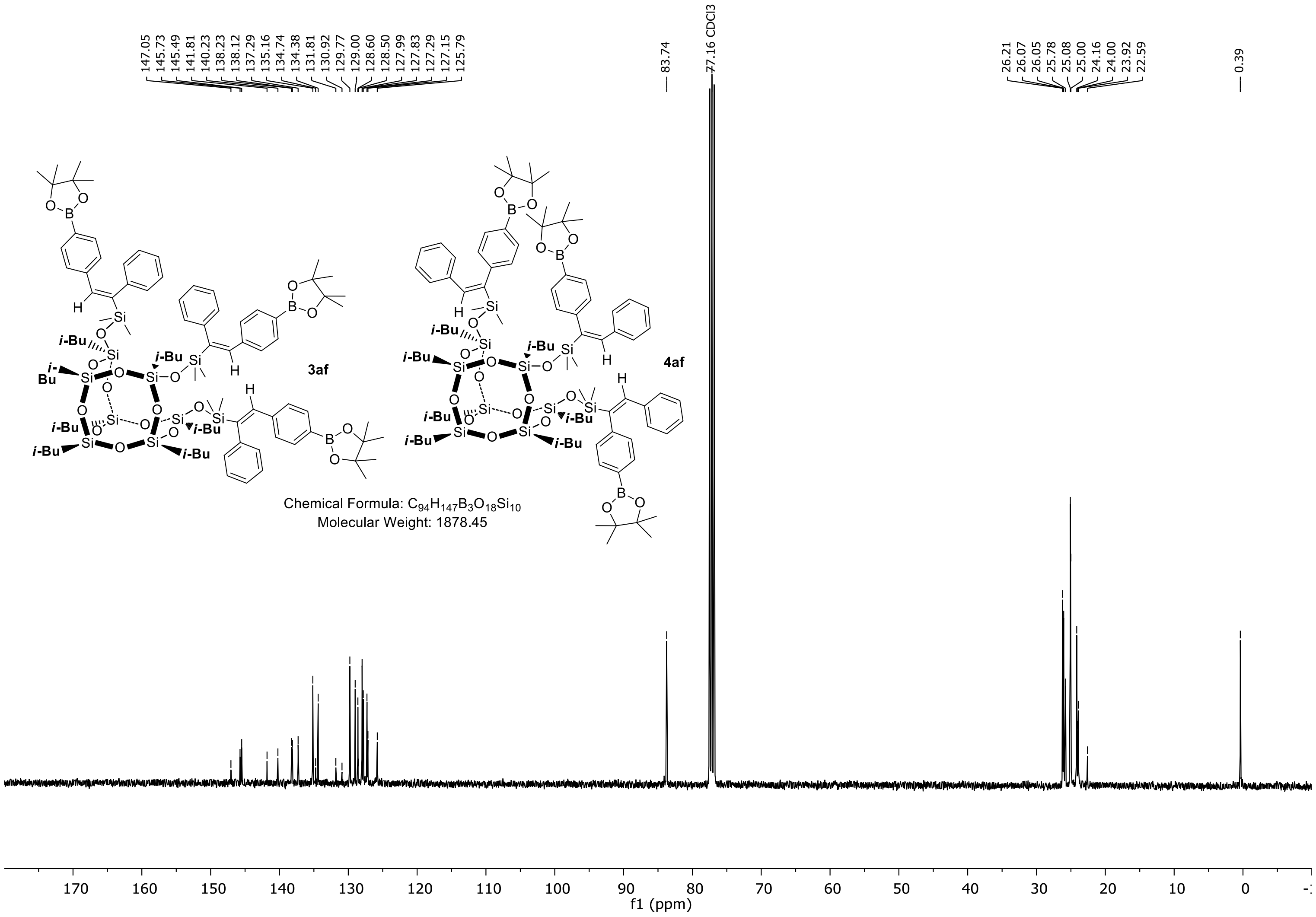

Figure S61. ^{13}C NMR of **3af/4af** mixture.

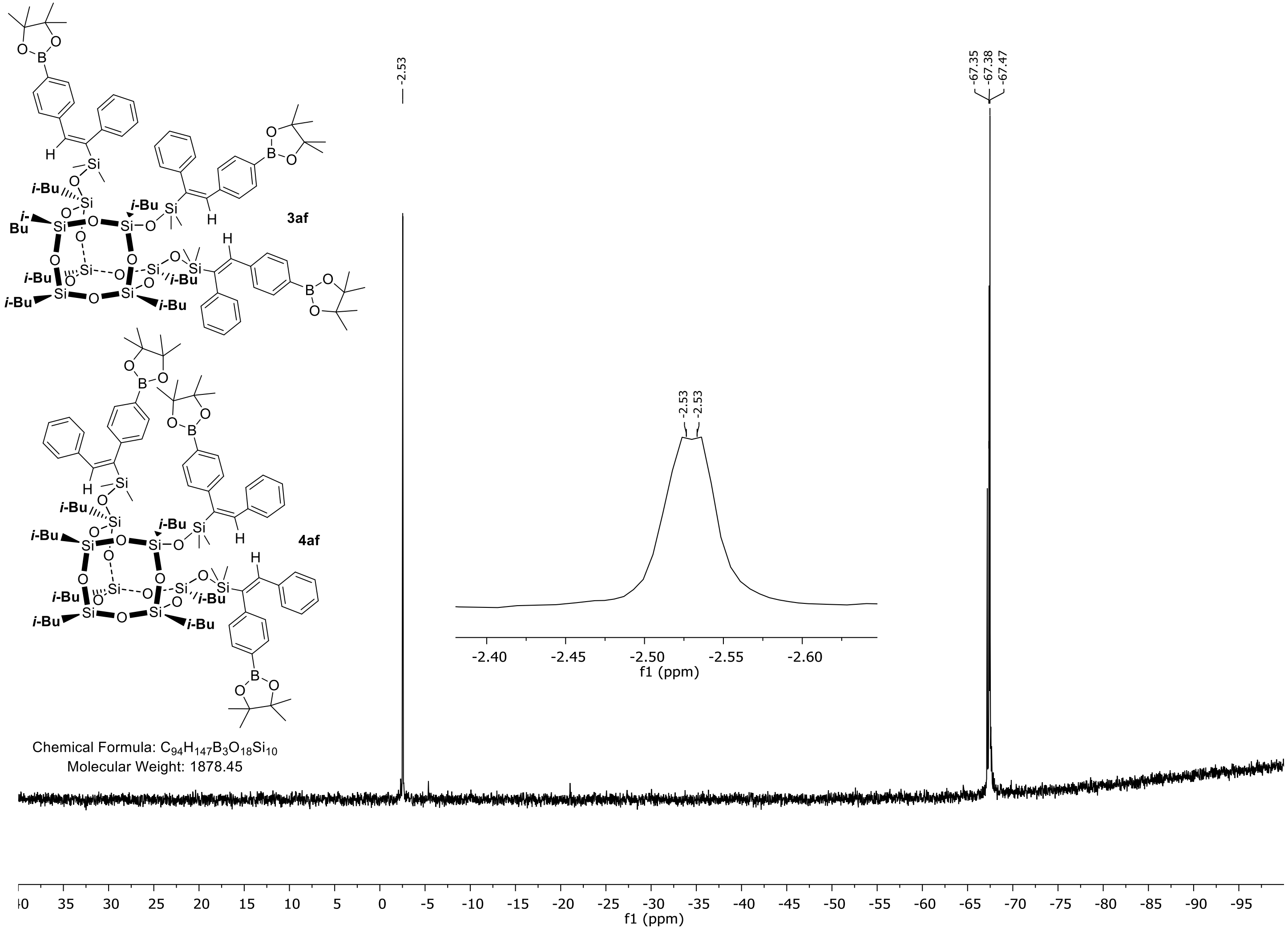

Figure S62. ^{29}Si NMR of **3af/4af** mixture.

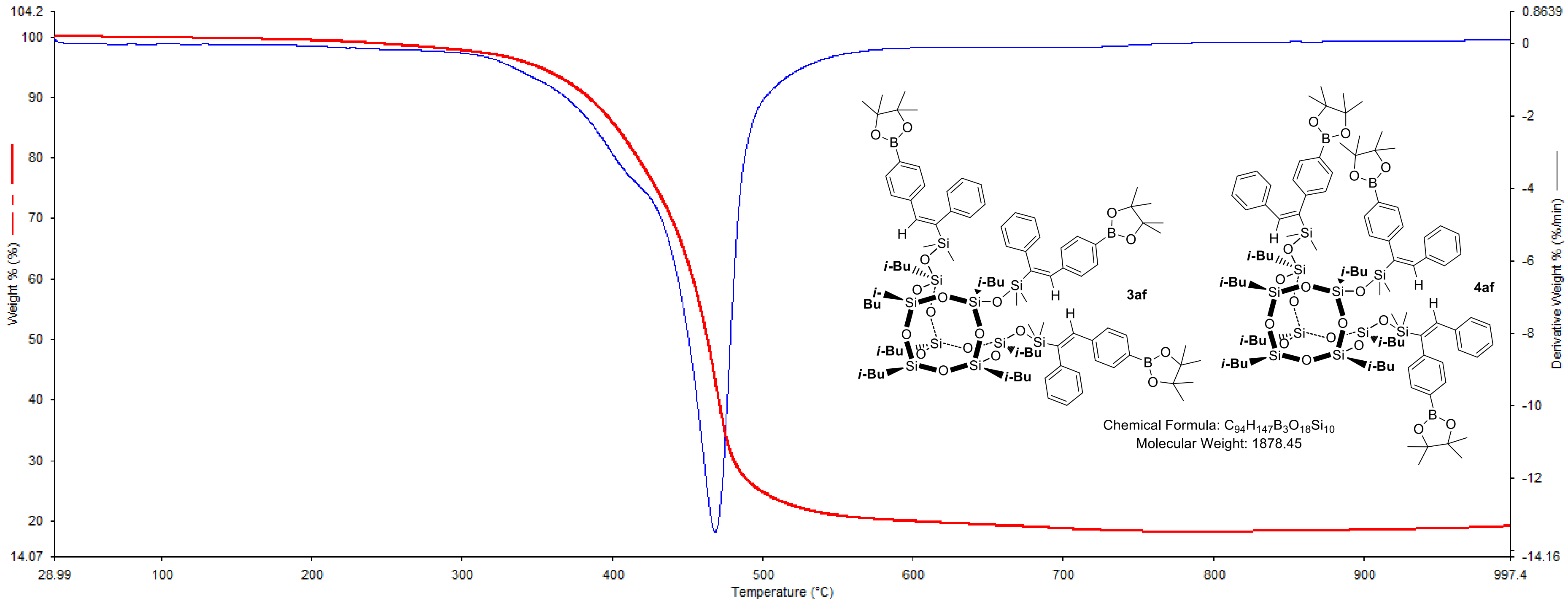

Figure S63. TGA/DTG curves of **3af/4af** mixture.

3ag

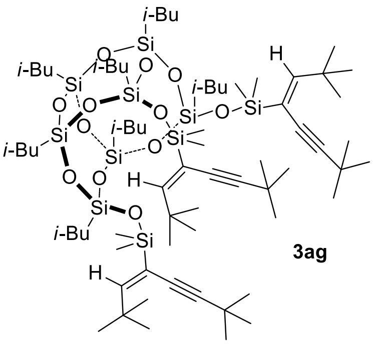

Chemical Formula: $C_{70}H_{138}O_{12}Si_{10}$
Molecular Weight: 1452,69

Isolated yield = 92%, colorless oil.

The traces of product **4ag** were observed.

^{1}H NMR ($CDCl_3$, 300 MHz, δ, ppm): 0.07-0.17 (m, 18H, $SiC\underline{H}_3$, product **4ag**), 0.26 (s, 18H, $SiC\underline{H}_3$, product **3ag**), 0.54-0.60 (m, 14H, $C\underline{H}_2$), 0.96-0.98 (m, 42H, $C\underline{H}_3$), 1.19 (s, 27H, $(C\underline{H}_3)_3$), 1.24 (s, 27H, $(C\underline{H}_3)_3$), 1.44 (s, 27H, $(=(OSi(CH_3)_3)CC\underline{H}_3$, product **4ag**), 1.83-1.91 (m, 21H, $C\underline{H}$), 6.08 (s, 3H, $=C\underline{H}$). **^{13}C NMR** ($CDCl_3$, 75 MHz, δ, ppm): -0.07 ($OSi\underline{C}H_3$), 22.66 ($\underline{C}H_2CH(CH_3)_2$), 23.96, 24.04, 24.18 ($CH_2\underline{C}H(CH_3)_2$), 25.13, 25.81, 26.10, 26.28 ($CH_2CH(\underline{C}H_3)_2$), 28.60 ($\equiv C\underline{C}(CH_3)_3$), 29.77 ($=C(H)C(\underline{C}H_3)_3$), 30.41, 30.51, 30.58 ($=(OSi(CH_3)_3)C(\underline{C}H_3)_3$, product **4ag**), 31.07 ($\equiv CC(\underline{C}H_3)_3$), 35.51 ($=C(H)\underline{C}(CH_3)_3$), 78.42 ($\equiv\underline{C}C(CH_3)_3$), 108.88 ($\underline{C}\equiv CC(CH_3)_3$), 121.51 ($=\underline{C}C\equiv CC(CH_3)_3$), 158.25 ($=\underline{C}(H)C(CH_3)_3$). **^{29}Si NMR** ($CDCl_3$, 79 MHz, δ, ppm): -66.76 – (- 70.30) (SiO_3), -4.02 ($OSi(CH_3)_2$, product **4ag**), -3.18, -3.12, -3.03 ($OSi(CH_3)_2$, product **3ag**). **FT IR (cm^{-1}):** 2952.7, 2901.6, 2868.0, 1464.2, 1401.7, 1364.7, 1331.5, 1252.0, 1227.6, 1074.5, 1045.6, 899.0, 834.0, 784.0, 738.3, 448.3. **MALDI TOF MS -** (m/z) ([M+Na], (%)): 1474.79.

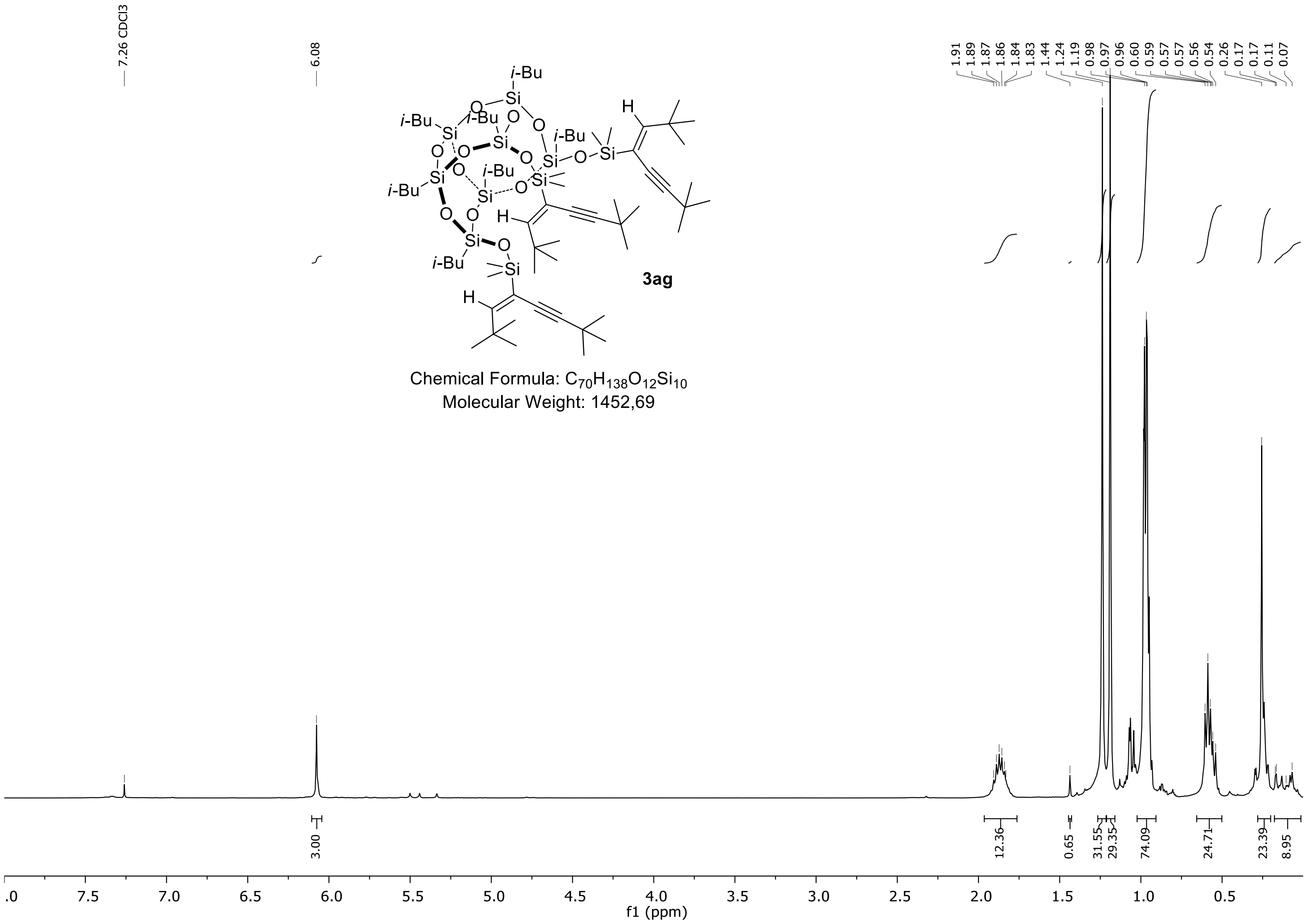

Figure S64. ^{1}H NMR of **3ag/4ag** mixture.

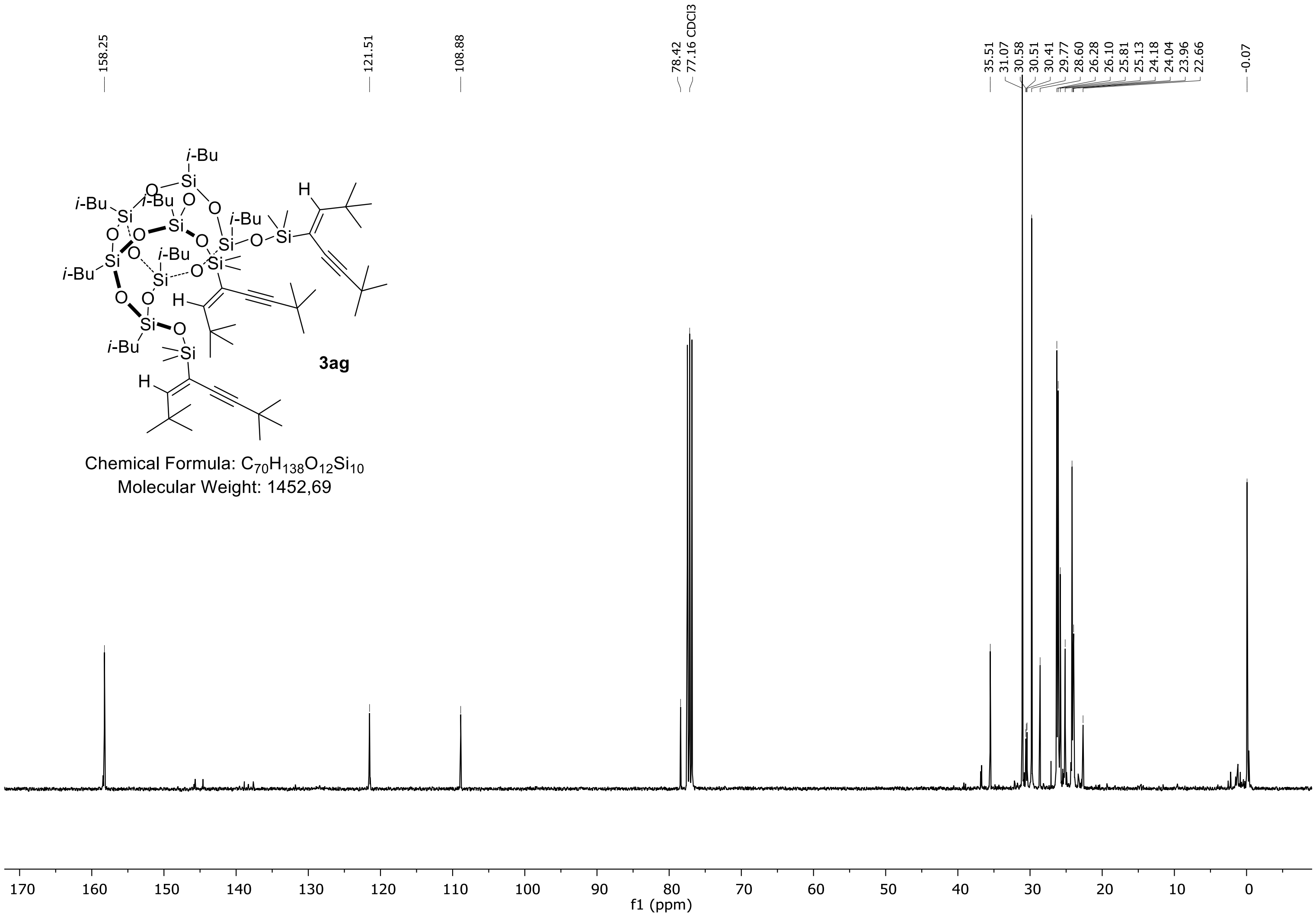

Figure S65. ^{13}C NMR of **3ag/4ag** mixture.

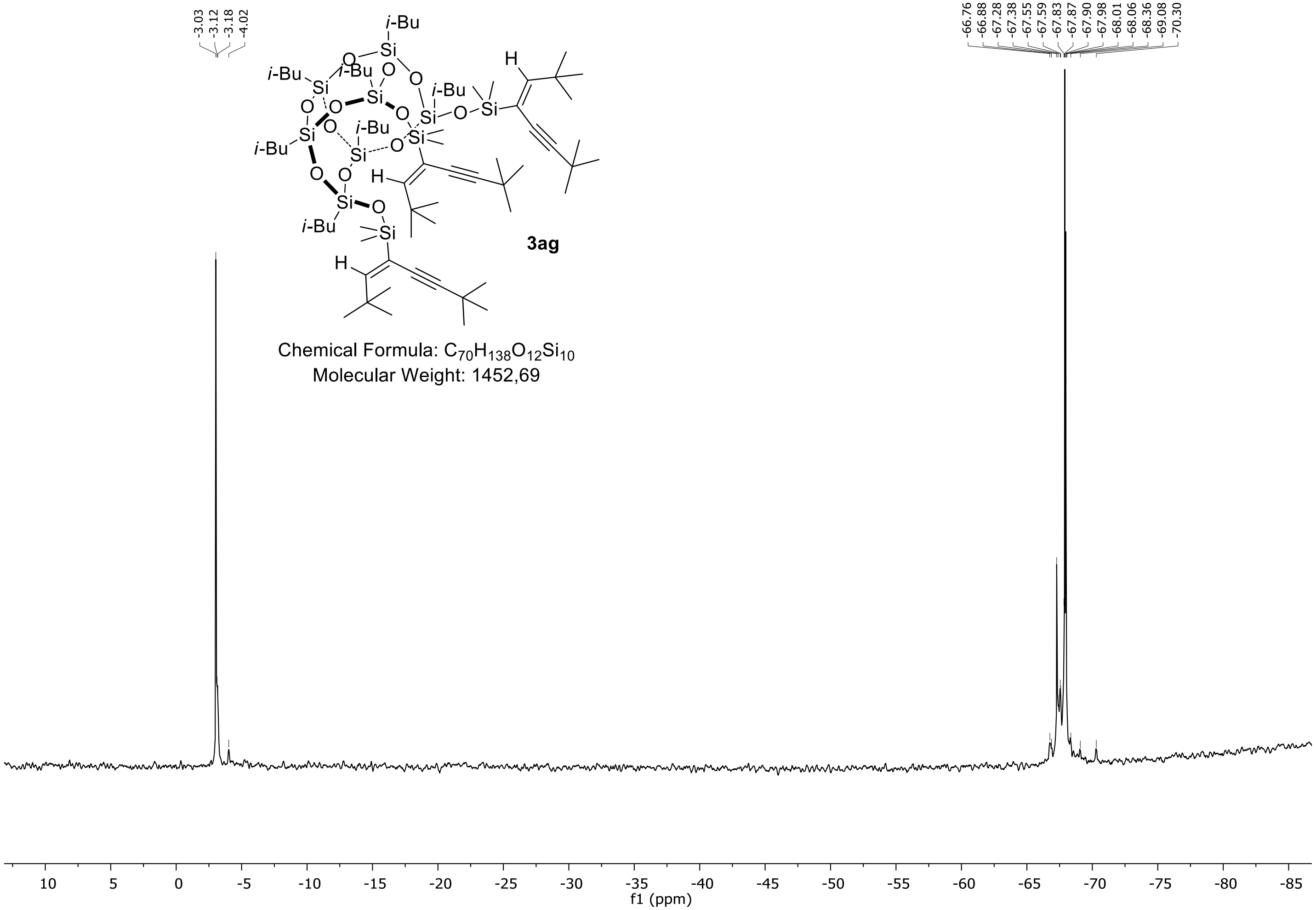

Figure S66. ^{29}Si NMR of **3ag/4ag** mixture.

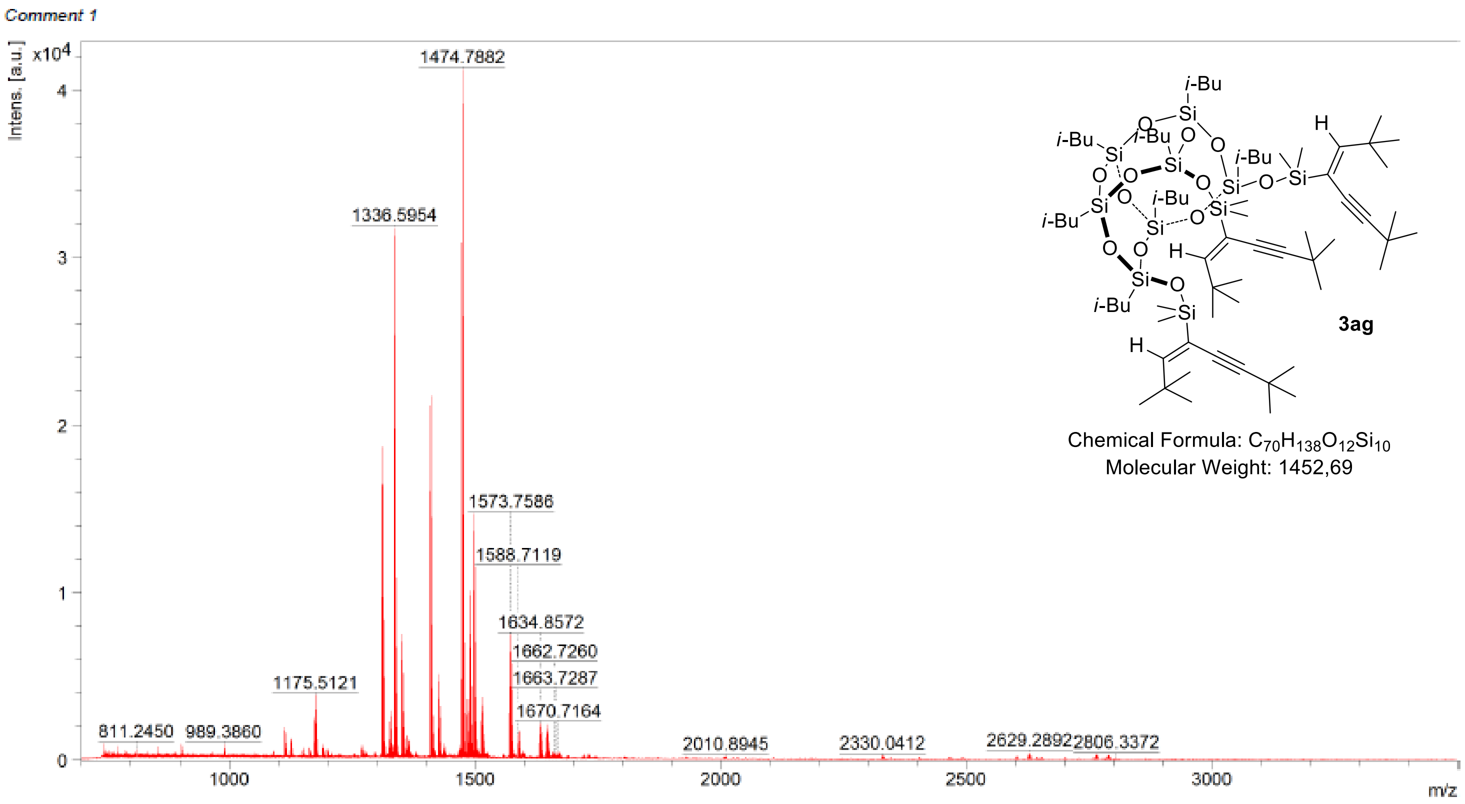

Figure S67. MALDI TOF MS spectra of **3ag/4ag** mixture.

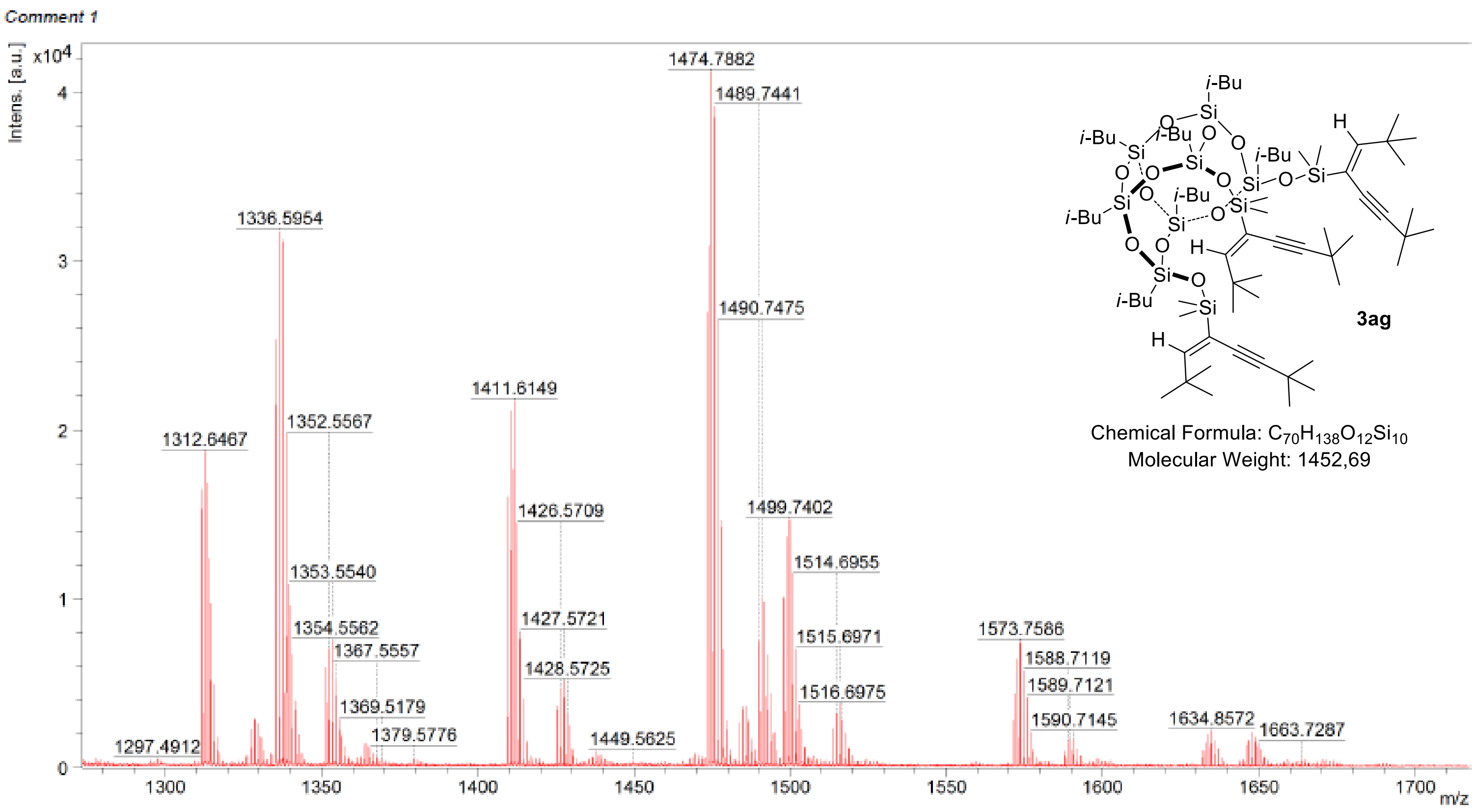

Figure S68. MALDI TOF MS spectra of **3ag/4ag** mixture.

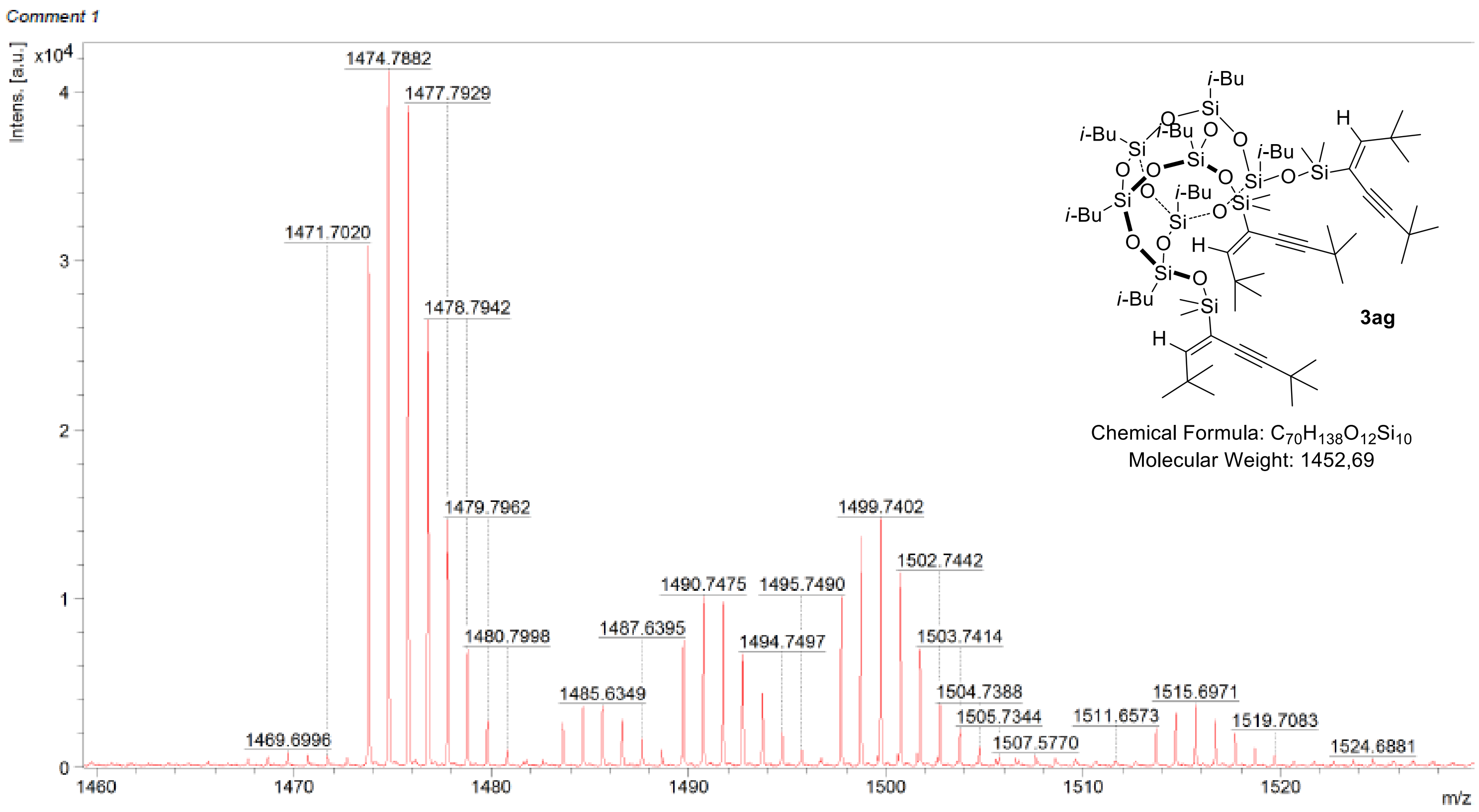

Figure S69. MALDI TOF MS spectra of **3ag/4ag** mixture.

3ah

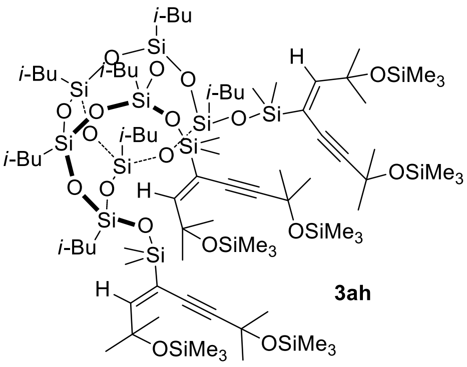

Chemical Formula: $C_{82}H_{174}O_{18}Si_{16}$
Molecular Weight: 1897,62

Isolated yield = 87%, colorless oil.

^{1}**H NMR** ($CDCl_3$, 300 MHz, δ, ppm): 0.12 (s, 9H, $SiC\underline{H}_3$), 0.17 (s, 9H, $SiC\underline{H}_3$), 0.29 (s, 6H, $OSiC\underline{H}_3$), 0.56-0.60 (m, 14H, $C\underline{H}_2$), 0.96-0.98 (m, 42H, $C\underline{H}_3$), 1.50 (s, 18H, $\equiv CC(C\underline{H}_3)_2OSi(CH_3)_3$), 1.51 (s, 18H, $=C(H)C(C\underline{H}_3)OSi(CH_3)_3$), 1.83-1.89 (m, 21H, $C\underline{H}$), 6.42 (s, 3H, $=C\underline{H}$). ^{13}**C NMR** ($CDCl_3$, 75 MHz, δ, ppm): 0.19 ($OSi\underline{C}H_3$), 2.13 ($\equiv CC(CH_3)_2OSi(\underline{C}H_3)_3$), 2.72 ($=C(H)C(CH_3)_2OSi(\underline{C}H_3)_3$), 22.61 ($\underline{C}H_2CH(CH_3)_2$), 23.95, 24.01, 24.13, 24.17, 24.27 ($CH_2\underline{C}H(CH_3)_2$), 25.06, 25.10, 25.80, 26.01, 26.11, 26.23, 26.29 ($CH_2CH(\underline{C}H_3)_2$), 29.94 ($=C(H)C(\underline{C}H_3)_2OSi(CH_3)_3$), 33.20 ($\equiv CC(\underline{C}H_3)_2OSi(CH_3)_3$), 67.49 ($\equiv C\underline{C}(CH_3)_2OSi(CH_3)_3$), 76.34 ($=C(H)\underline{C}(CH_3)_2OSi(CH_3)_3$), 81.37 ($\underline{C}\equiv CC(CH_3)_2OSi(CH_3)_3$), 106.17, 106.22 ($\equiv \underline{C}C(CH_3)_2OSi(CH_3)_3$), 119.72, 119.75 ($=\underline{C}C\equiv CC(CH_3)_2OSi(CH_3)_3$), 159.73, 159.79 ($=\underline{C}(H)C(CH_3)_2OSi(CH_3)_3$). ^{29}**Si NMR** ($CDCl_3$, 79 MHz, δ, ppm): -67.54, -67.34 (SiO_3), -2.47 ($OSi(CH_3)_2$), 9.22 ($=C(H)C(CH_3)_2OSi(CH_3)_3$), 12.47 ($\equiv CC(CH_3)_2OSi(CH_3)_3$). **FT IR (cm^{-1}):** 2954.0, 2902.0, 2869.8, 1464.9, 1402.0, 1365.9, 1331.6, 1249.6, 1227.3, 1159.6, 1076.9, 1030.3, 919.0, 835.0, 784.8, 750.9. **MALDI TOF MS -** (m/z) ([M+Na], (%)): 1919.87.

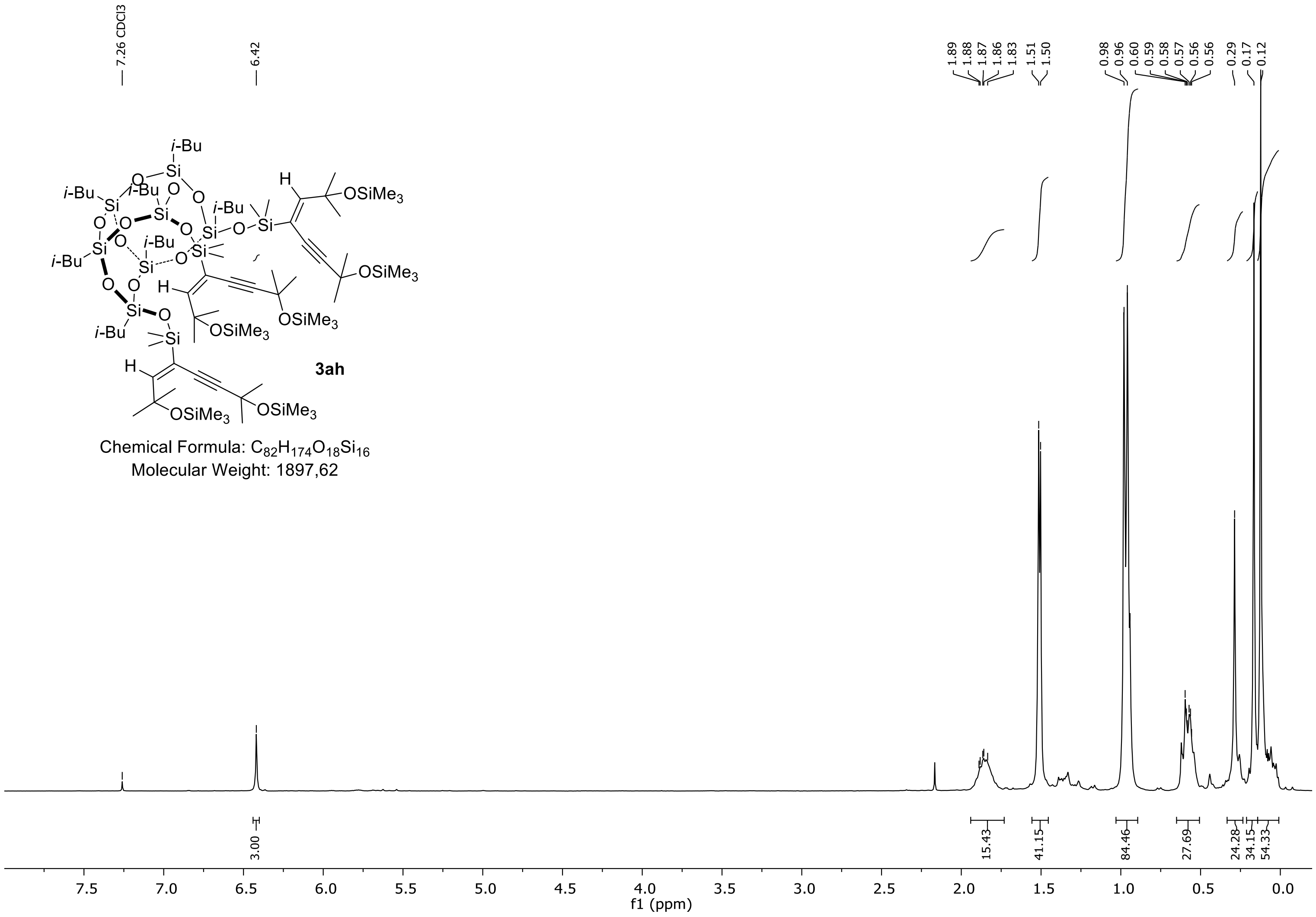

Figure S70. ^{1}H NMR of compound **3ah**.

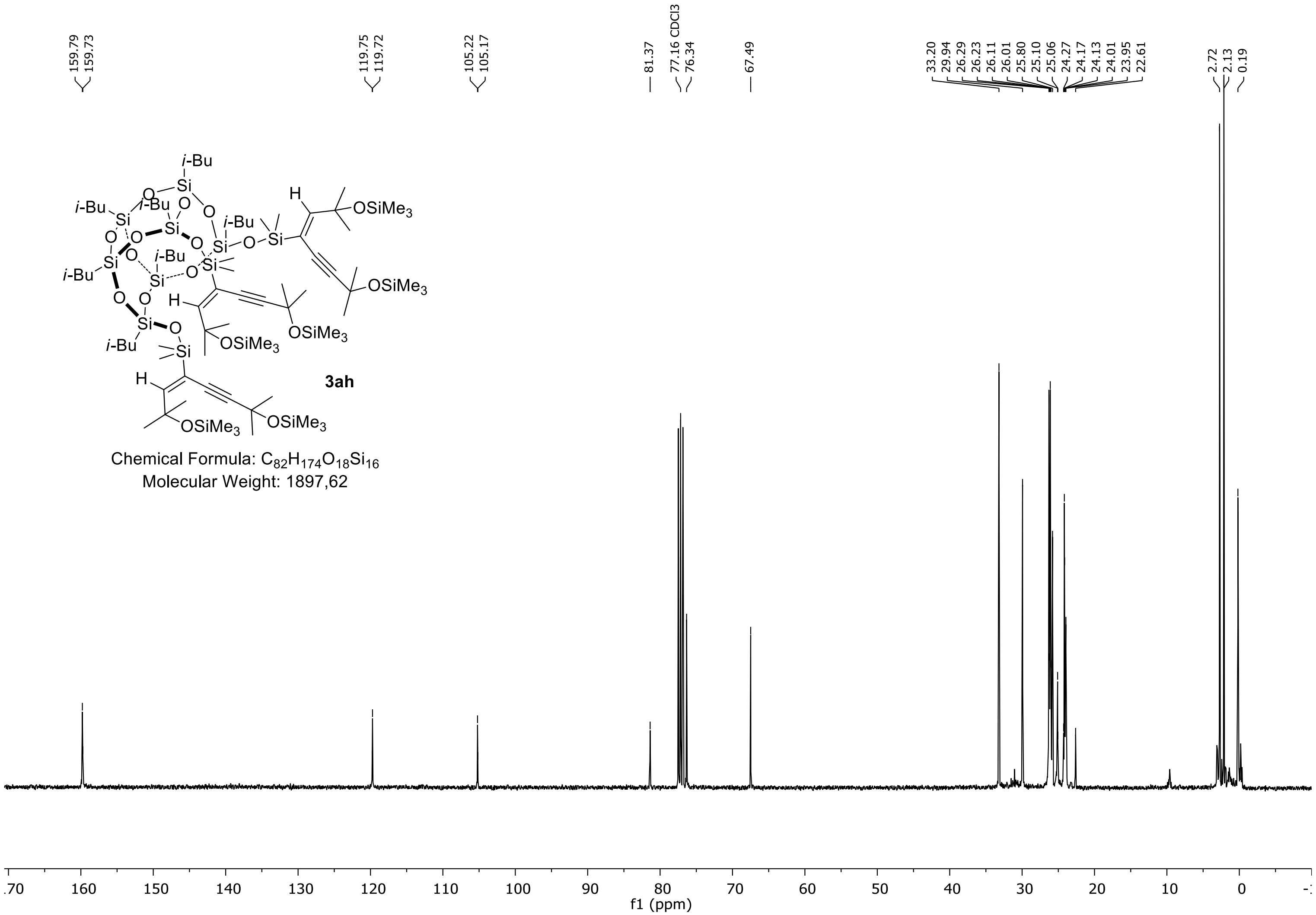

Figure S71. ^{13}C NMR of compound **3ah**.

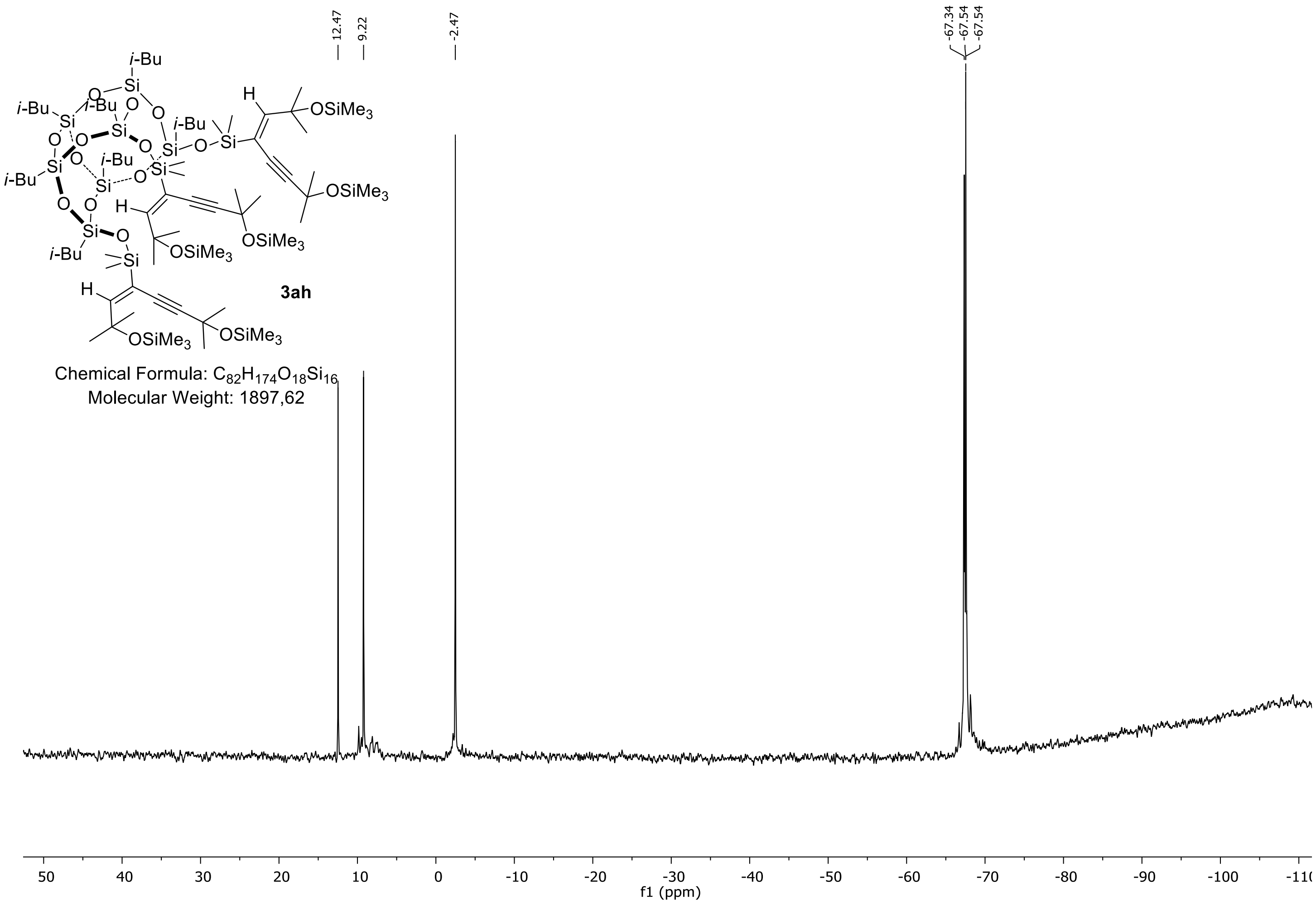

Figure S72. ^{29}Si NMR of compound **3ah**.

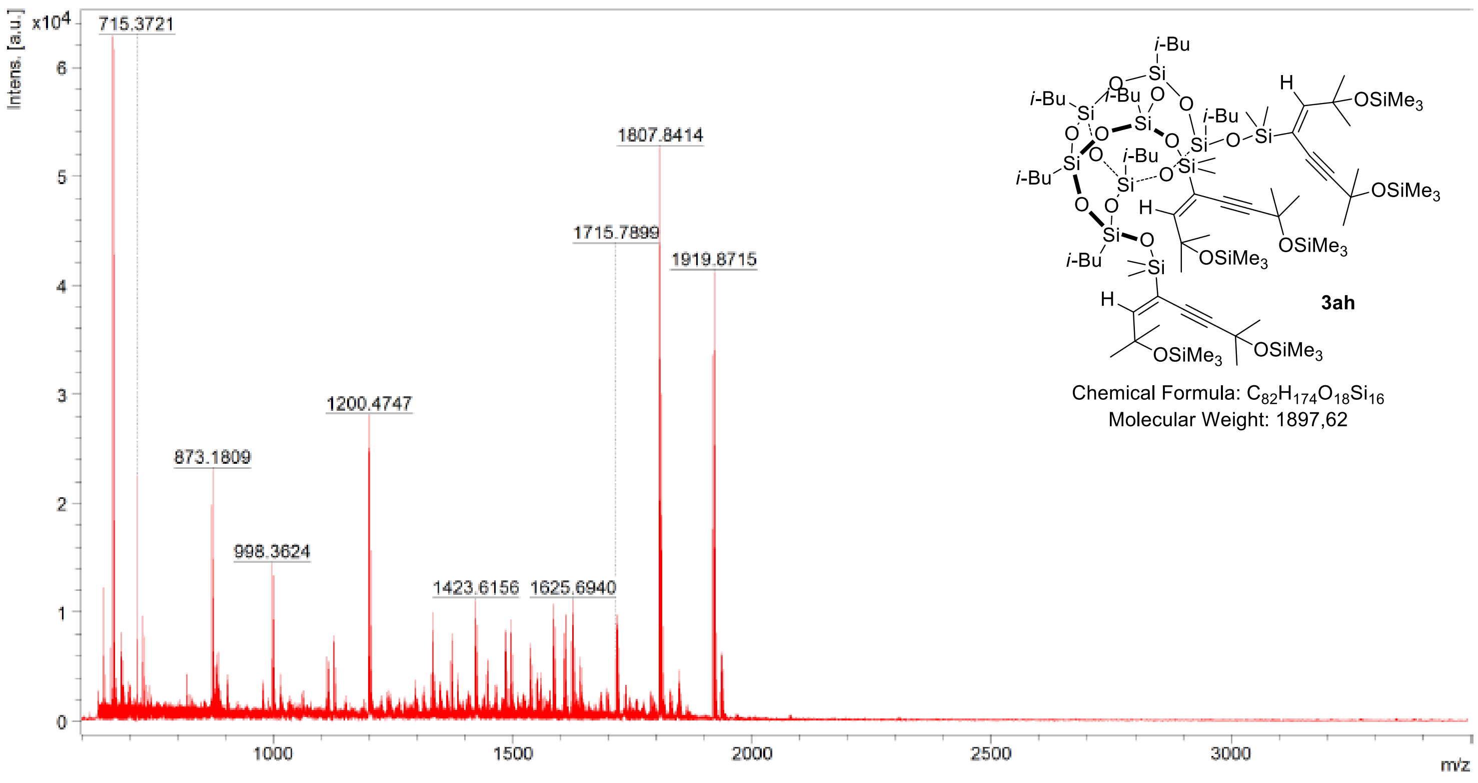

Figure S73. MALDI TOF MS spectra of compound **3ah**.

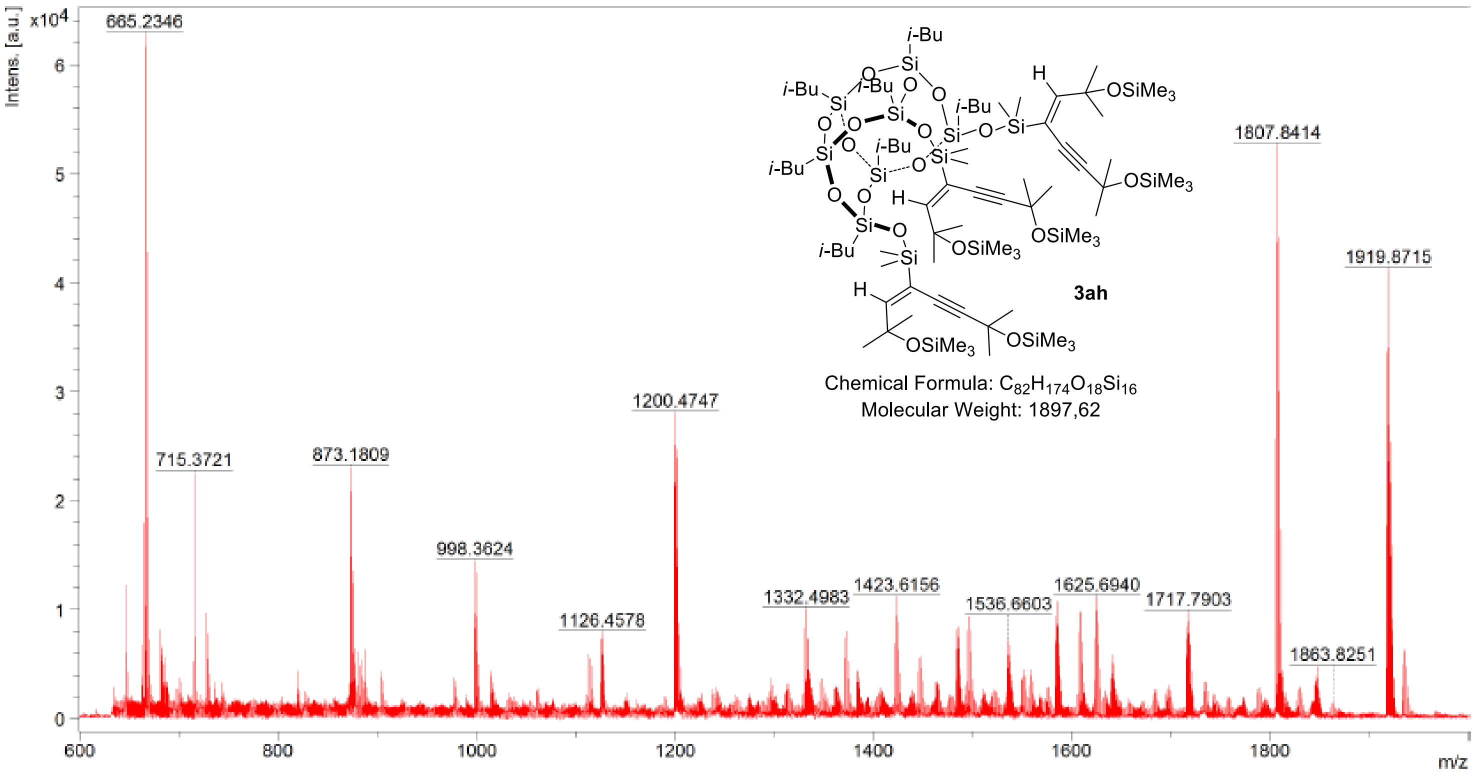

Figure S74. MALDI TOF MS spectra of compound **3ah**.

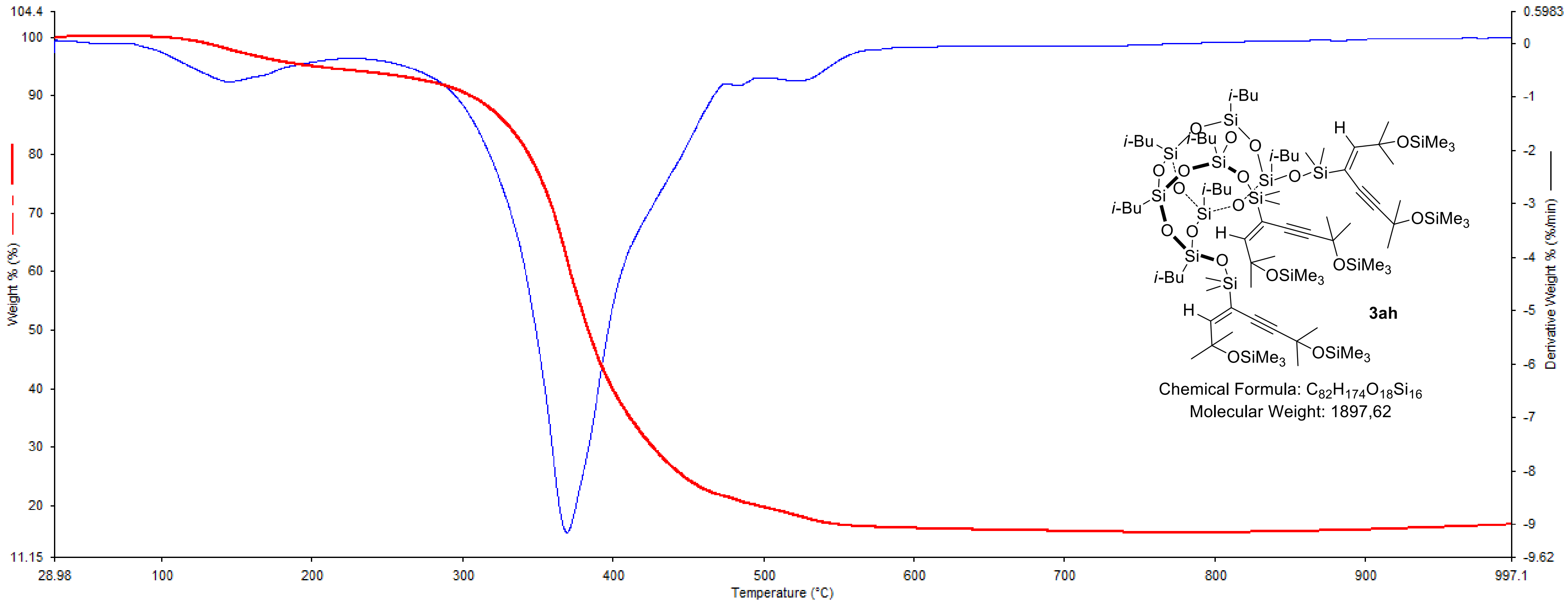

Figure S75. TGA/DTG curves of compound **3ah**.

3ai

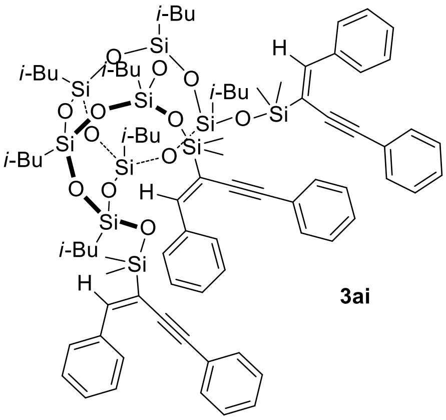

3ai

Chemical Formula: $C_{82}H_{114}O_{12}Si_{10}$
Molecular Weight: 1572,63

Isolated yield = 90%, colorless oil.

^{1}H NMR ($CDCl_3$, 300 MHz, δ, ppm): 0.39 (s, 18H, SiC$\underline{H}_3$), 0.54 – 0.65 (m, 14H, C$\underline{H}_2$), 0.89-0.94 (m, 42H, C$\underline{H}_3$), 1.79-1.90 (m, 21H, C$\underline{H}$), 6.99 (s, 3H, =C$\underline{H}$), 7.19-7.94 (m, 30H, $C_6\underline{H}_5$). **^{13}C NMR** ($CDCl_3$, 75 MHz, δ, ppm): 0.12 (OSi$\underline{C}H_3$), 22.59 ($\underline{C}H_2CH(CH_3)_2$), 23.87, 24.01, 24.13, 24.23 ($CH_2\underline{C}H(CH_3)_2$), 25.09, 25.79, 26.02, 26.22 ($CH_2CH(\underline{C}H_3)_2$), 90.33 ($\underline{C}$≡$CC_6H_5$), 100.79 (≡$\underline{C}CC_6H_5$), 122.48- 131.45 ($\underline{C}_6H_5$), 137.86 (=$\underline{C}$C≡$CC_6H_5$), 145.10 (=$\underline{C}(H)CC_6H_5$). **^{29}Si NMR** ($CDCl_3$, 79 MHz, δ, ppm): -67.27, -67.00 (SiO_3), -1.79 ($OSi(CH_3)_2$). **FT IR (cm^{-1}):** 2952.9, 2902.1, 2868.5, 1489.1, 1464.7, 1401.2, 1365.6, 1331.2, 1253.1, 1227.6, 1073.4, 1065.3, 854.5, 830.6, 784.7, 752.1, 734.5, 688.4, 595.0, 524.1, 444.1. **MALDI TOF MS -** (m/z) ([M+Na], (%)): 1594.59.

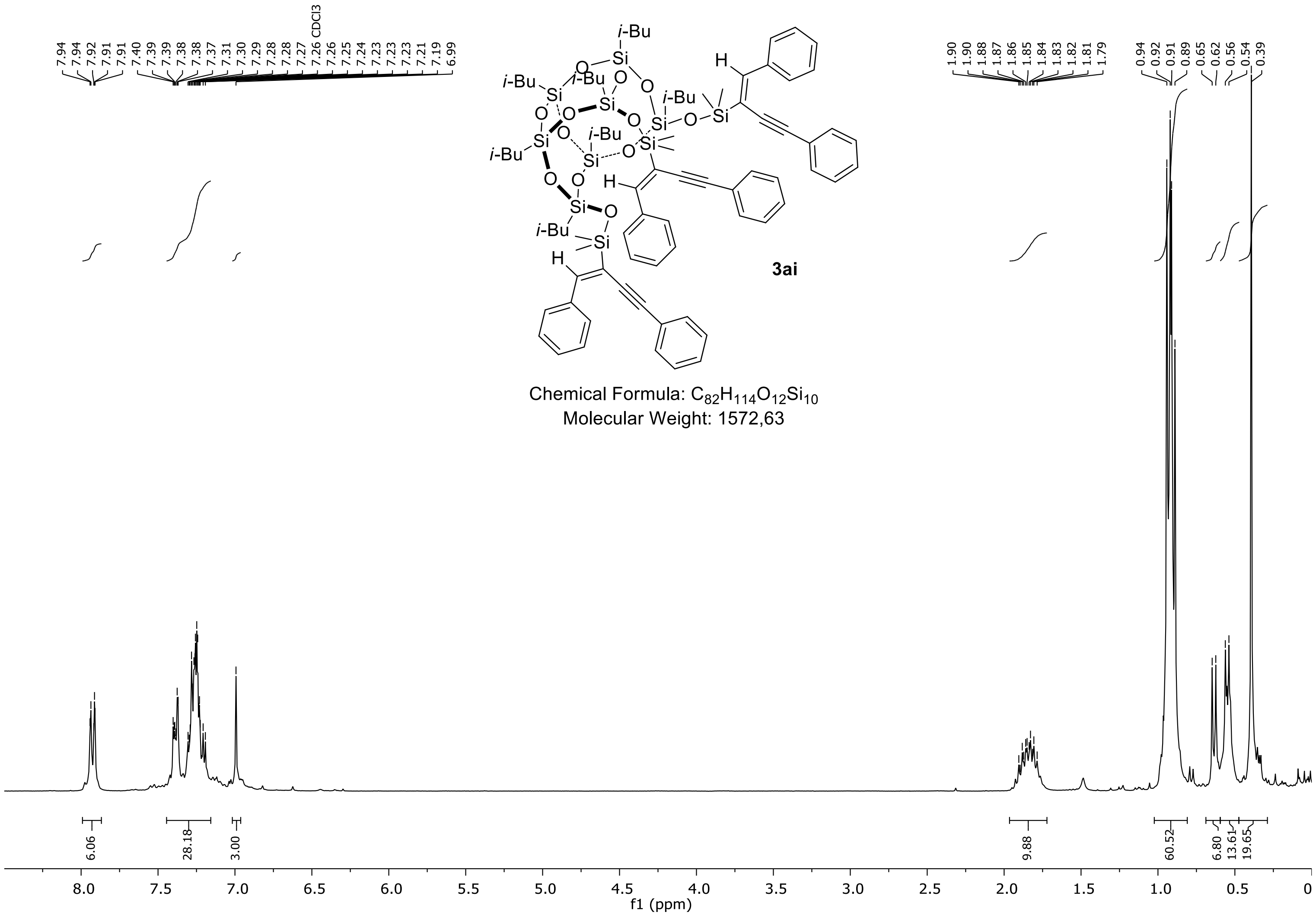

Figure S76. ^{1}H NMR of compound **3ai**.

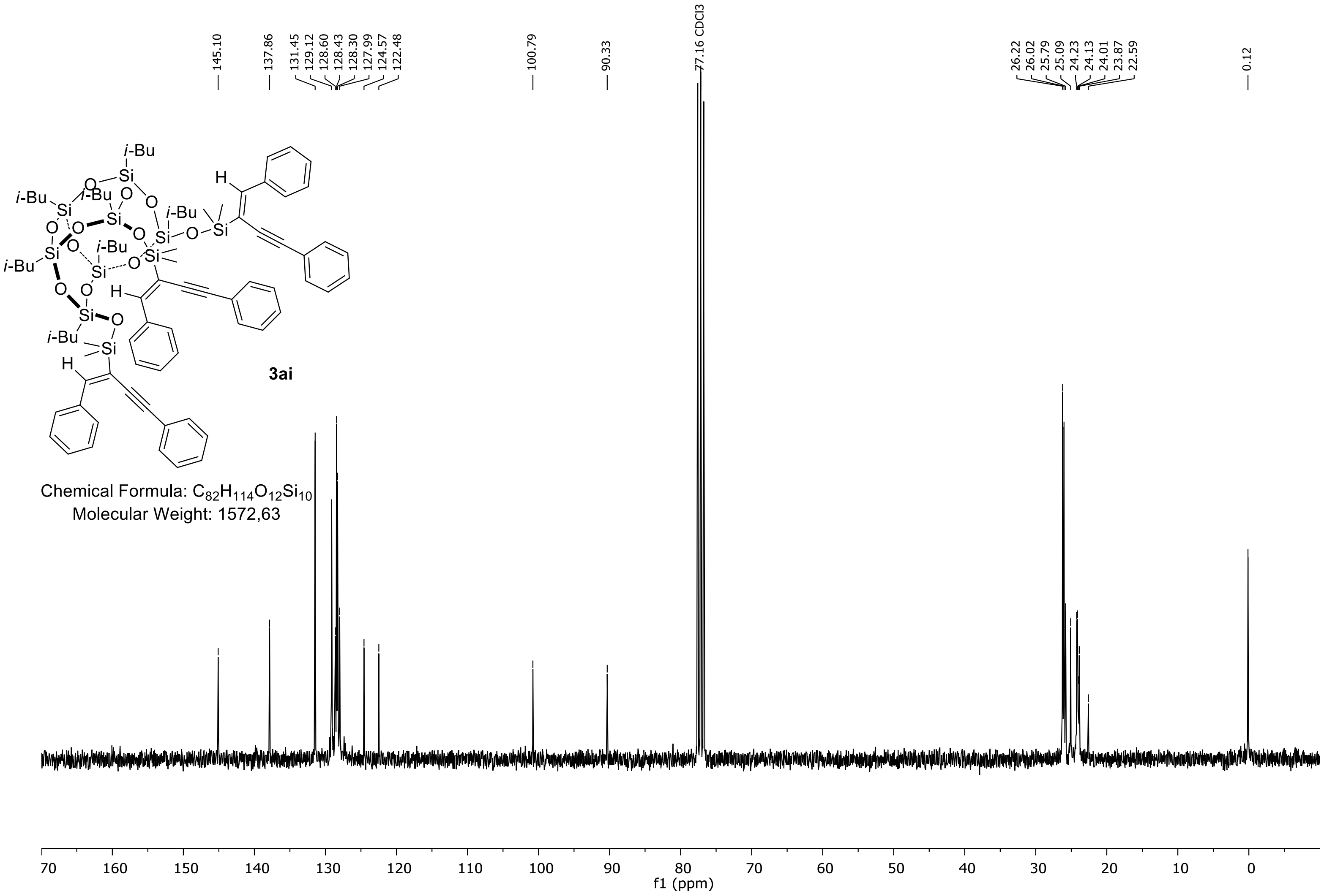

Figure S77. ^{13}C NMR of compound **3ai**.

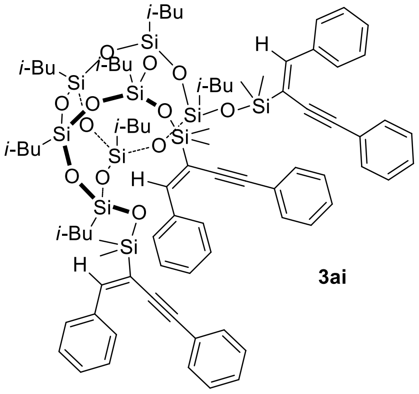

3ai

Chemical Formula: $C_{82}H_{114}O_{12}Si_{10}$
Molecular Weight: 1572,63

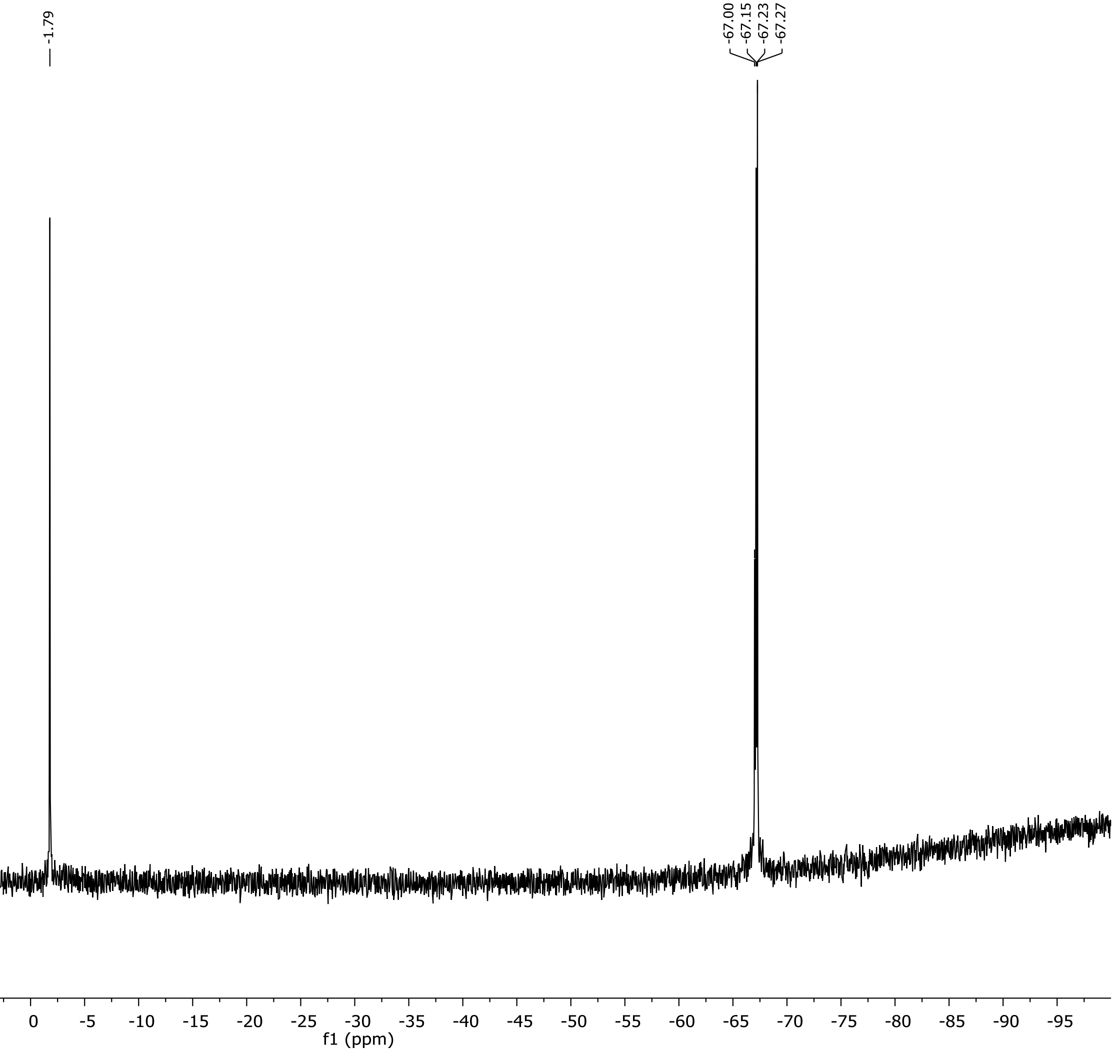

Figure S78. ^{29}Si NMR of compound **3ai**.

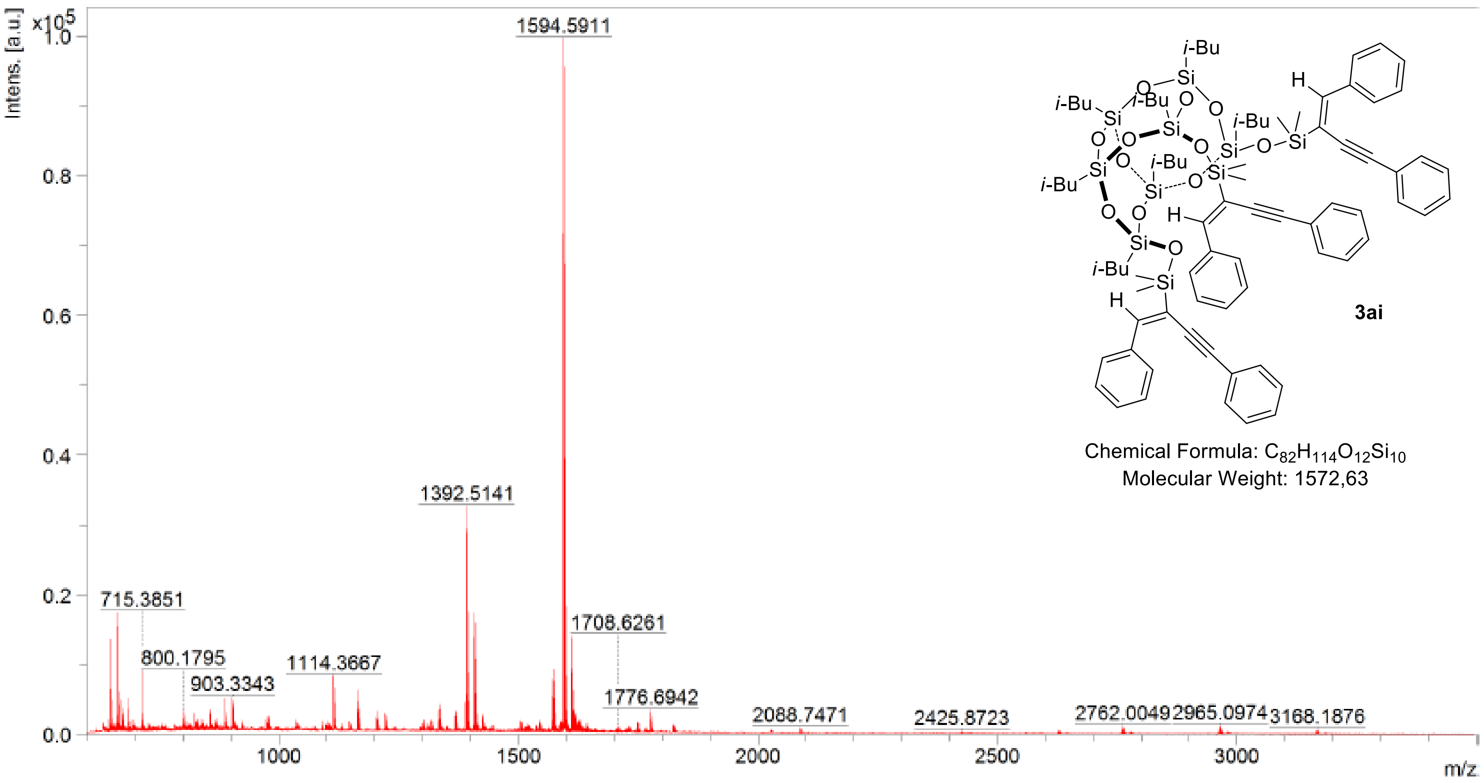

Figure S79. MALDI TOF MS spectra of compound **3ai**.

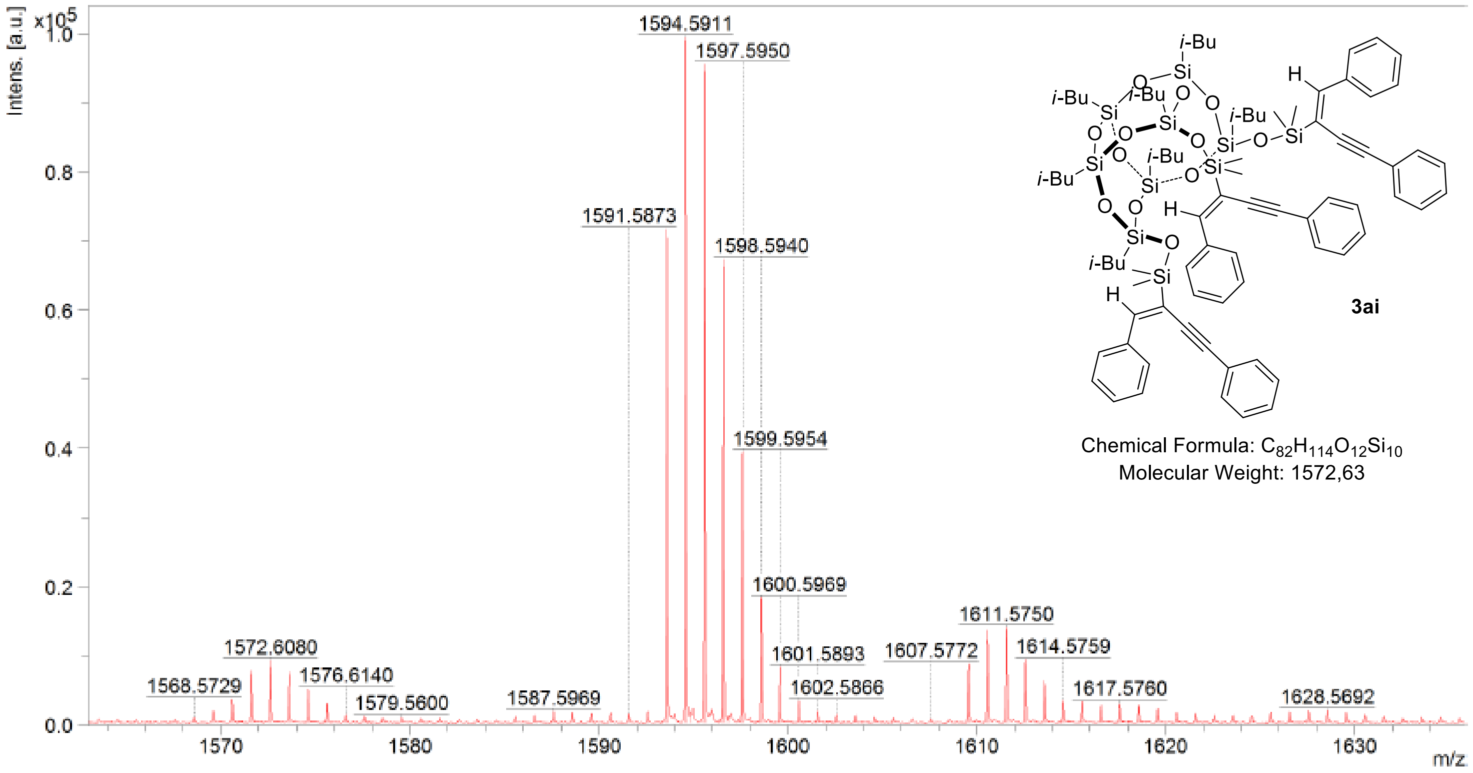

Figure S80. MALDI TOF MS spectra of compound **3ai**.

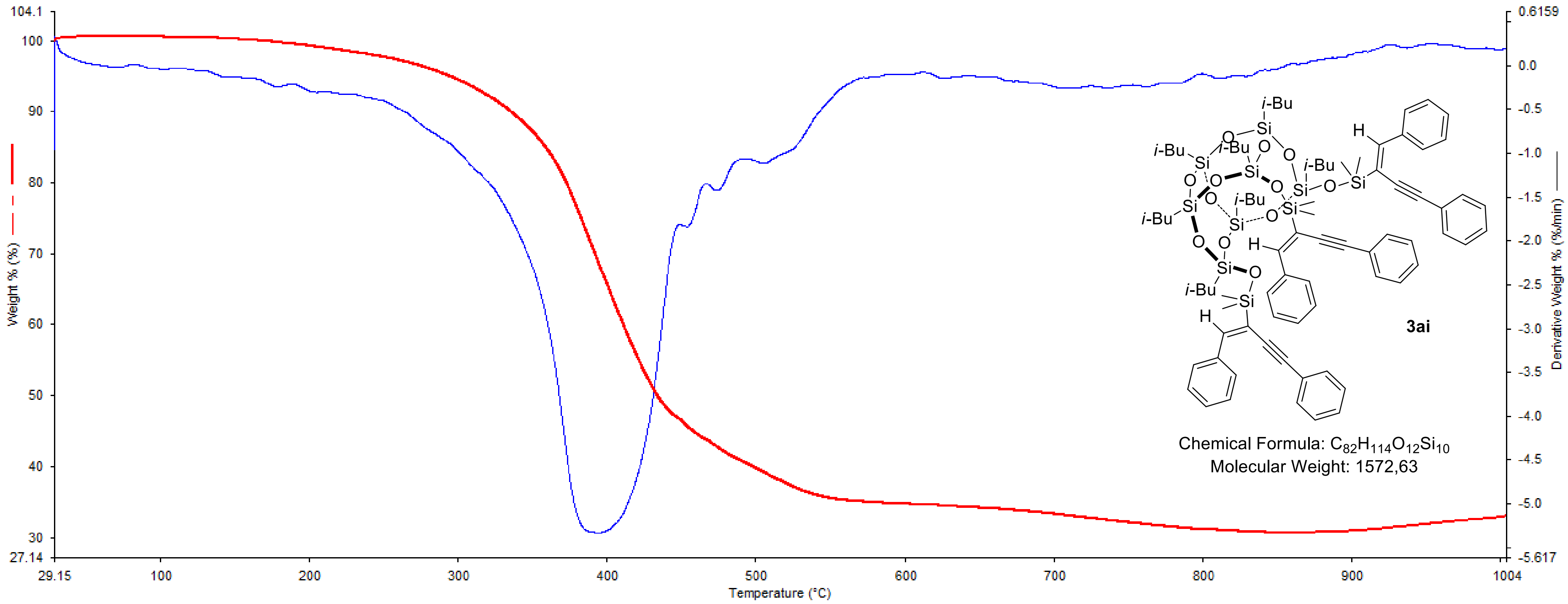

Figure S81. TGA/DTG curves of compound **3ai**.

3bi

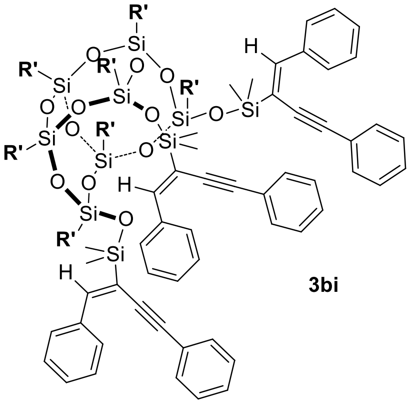

R'= $CH_2CH(CH_3)CH_2C(CH_3)_3$

Chemical Formula: $C_{110}H_{170}O_{12}Si_{10}$

Molecular Weight: 1965,4

Isolated yield = 91%, colorless oil.

^{1}H NMR ($CDCl_3$, 300 MHz, δ, ppm): 0.09 (s, 18H, SiC$\underline{H}_3$), 0.56 – 0.64 (m, 14H, SiC$\underline{H}_2$), 0.86 – 0.91 (m, 63H, C(C$\underline{H}_3$)$_3$, 1.01 – 1.03 (m, 21H, CH(C$\underline{H}_3$)), 1.12 – 1.16 (m, 7H, C$\underline{H}_2$), 1.21 – 1.28 (m, 7H, C$\underline{H}_2$), 1.84 – 1.89 (m, 7H, C$\underline{H}$(CH_3)), 7.04 (s, 3H, =C$\underline{H}$), 7.30 – 7.97 (m, 30H, $C_6\underline{H}_5$). **^{13}C NMR** ($CDCl_3$, 75 MHz, δ, ppm): 0.28 (OSi$\underline{C}H_3$), 25.25, 25.39 ($\underline{C}H_2(CH_2)_4CH(CH_3)_2$, 25.87, 26.46 ($\underline{C}H, Si\underline{C}H_2$, CH($\underline{C}H_3$)), 30.32, 30.41 (($\underline{C}H_3$)$_3$), 31.29, 31.32, 31.34 ($\underline{C}(CH_3)_3$), 54.38, 55.00, 55.26 ($\underline{C}H_2C(CH_3)_3$), 90.38 ($\underline{C}$≡$CC_6H_5$), 100.78 (≡$\underline{C}CC_6H_5$), 122.45 – 132.66 ($\underline{C}_6H_5$), 137.85 (=$\underline{C}$C≡$CC_6H_5$), 145.15 (=$\underline{C}(H)CC_6H_5$). **^{29}Si NMR** ($CDCl_3$, 79 MHz, δ, ppm): -67.35 (SiO_3), -2.10, -2.01, -1.92 (OSi$(CH_3)_2$). **FT IR (cm^{-1}):** 2904.9, 2868.0, 1489.1, 1466.9, 1392.9, 1363.8, 1252.2, 1225.7, 1076.8, 908.7, 854.5, 785.3, 752.9, 688.7.

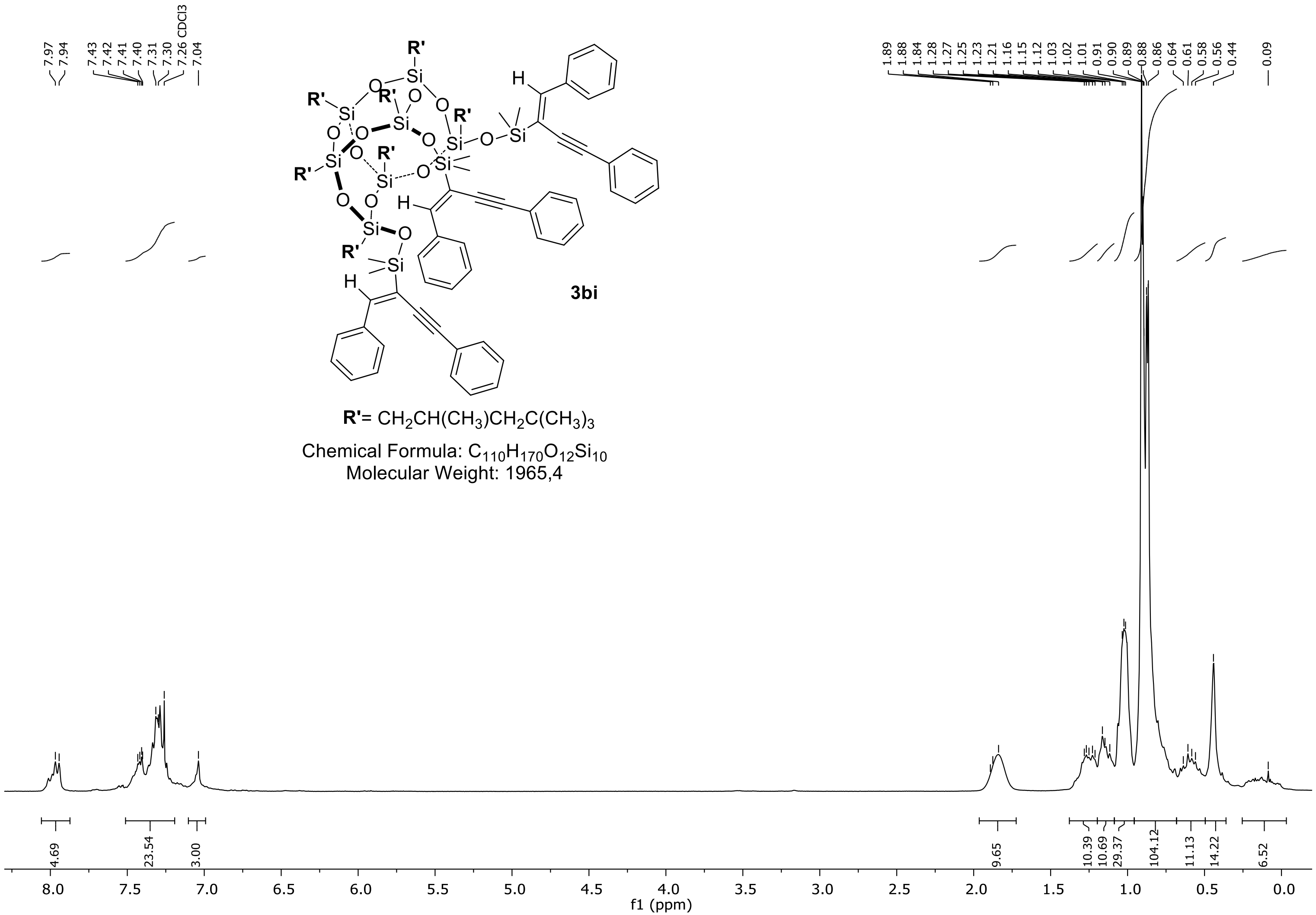

Figure S82. ^{1}H NMR of compound **3bi**.

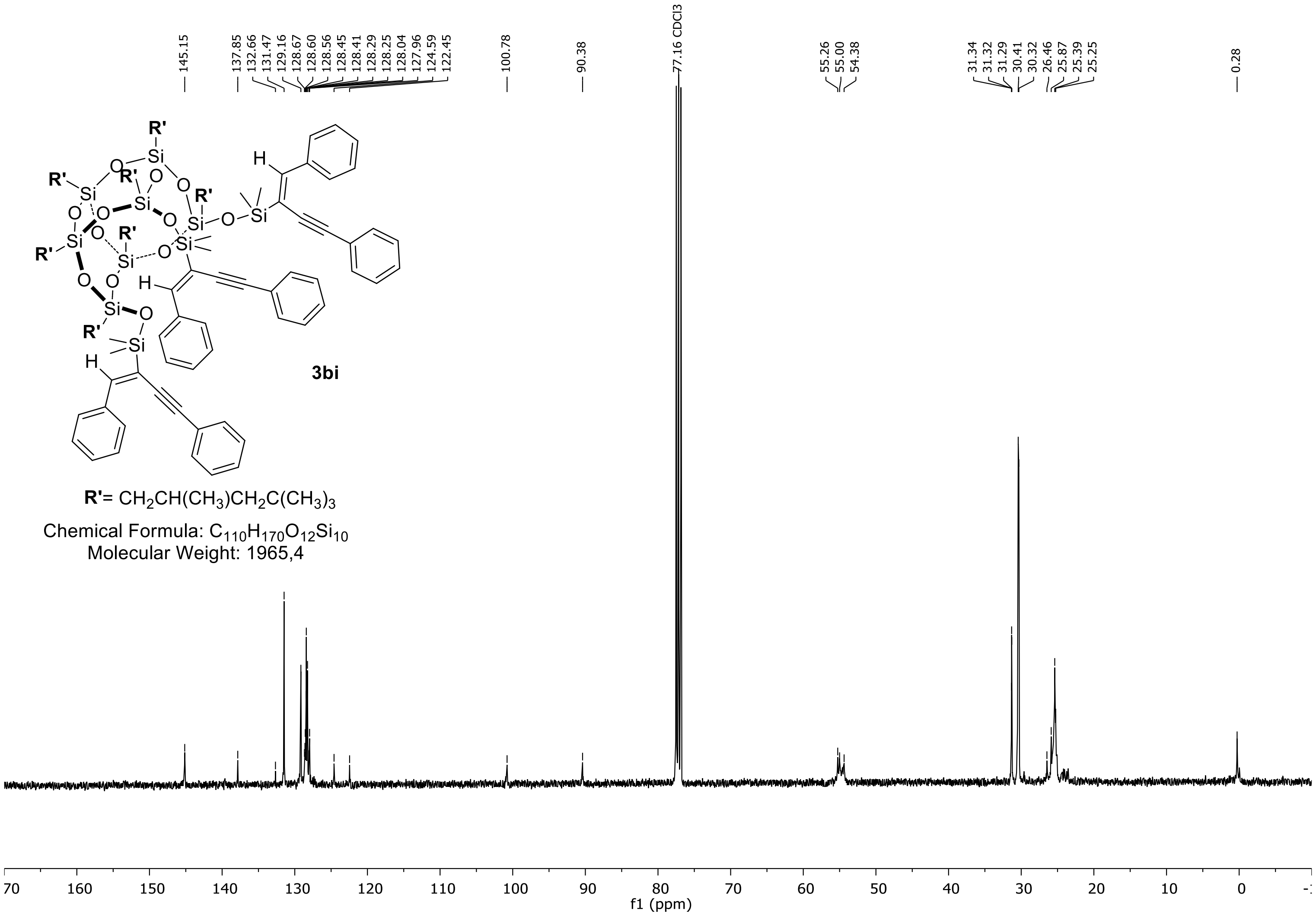

Figure S83. ^{13}C NMR of compound **3bi**.

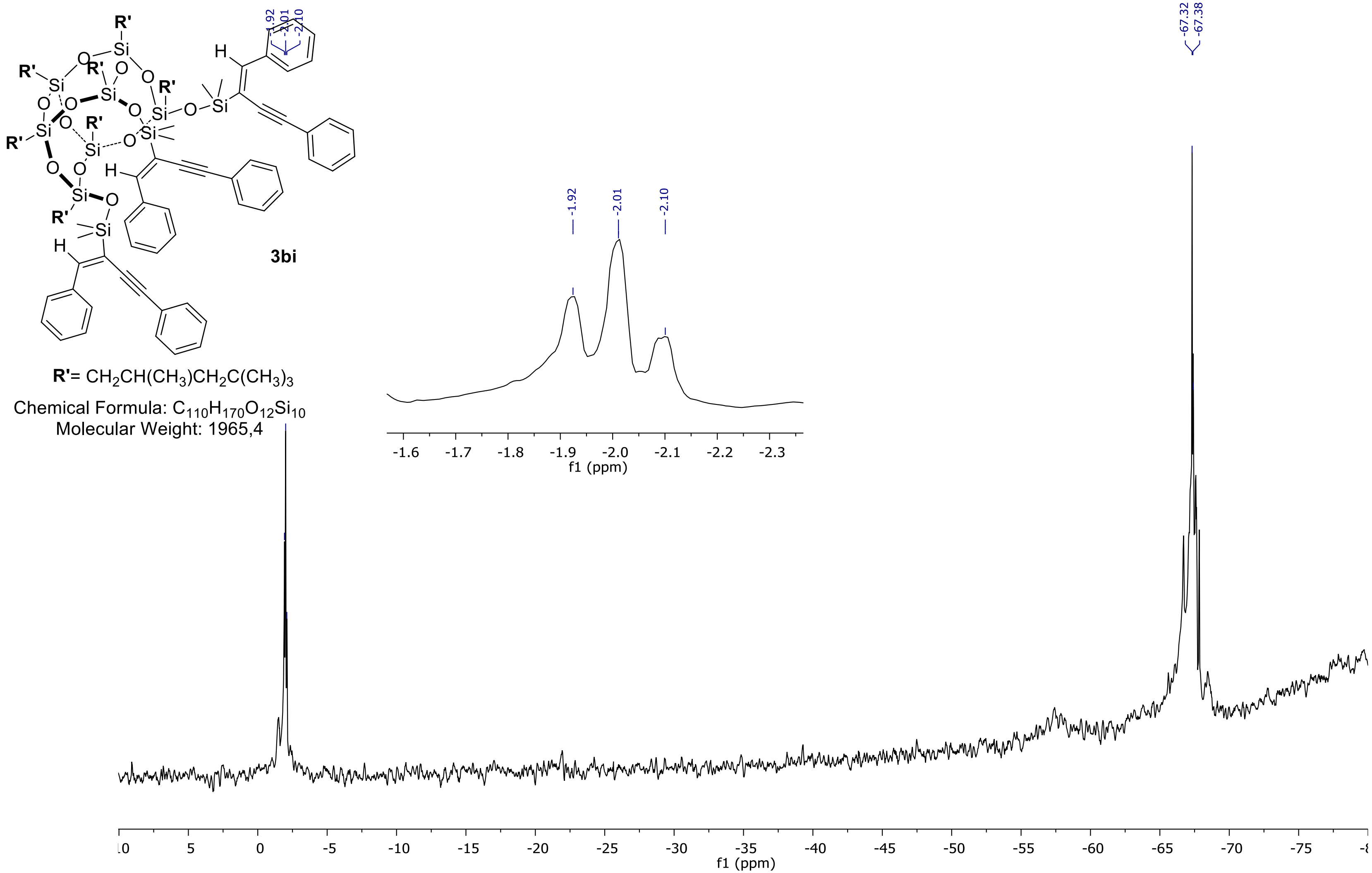

Figure S84. ^{29}Si NMR of compound **3bi**.

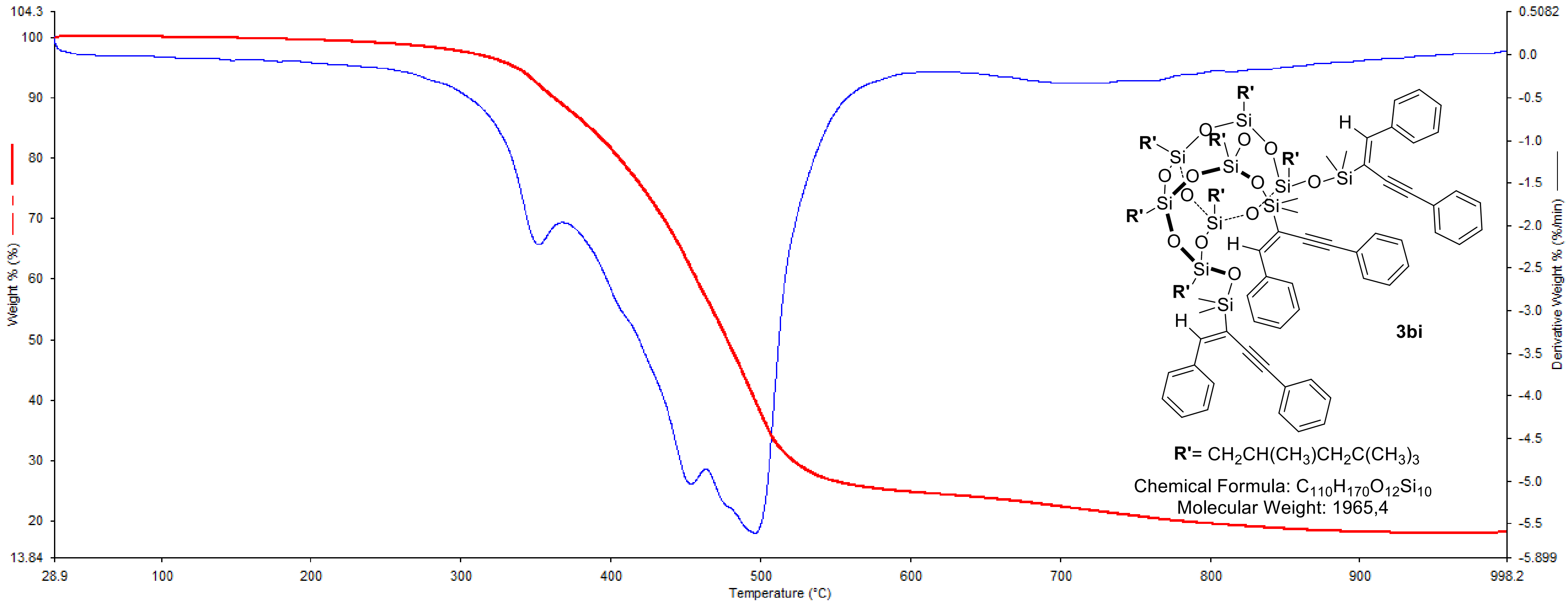

Figure S85. TGA/DTG curves of compound **3bi**.

3aj

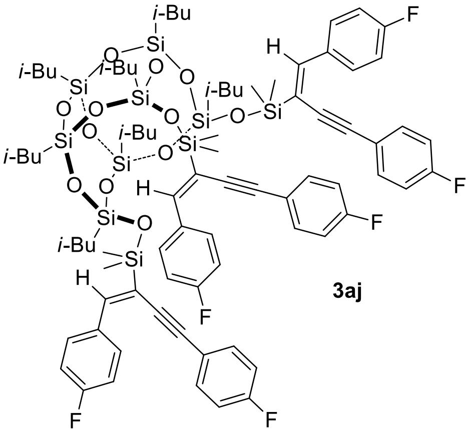

3aj

Chemical Formula: $C_{82}H_{108}F_6O_{12}Si_{10}$
Molecular Weight: 1680,57

Isolated yield = 94%, colorless oil.

^{1}H NMR ($CDCl_3$, 300 MHz, δ, ppm): 0.38 (s, 9H, $SiC\underline{H}_3$), 0.56 – 0.66 (m, 14H, $C\underline{H}_2$), 0.92-0.97 (m, 42H, $C\underline{H}_3$), 1.80-1.91 (m, 21H, $C\underline{H}$), 6.94-7.91 (m, 33H, $=C\underline{H}$, $C_6\underline{H}_5$). **^{13}C NMR** ($CDCl_3$, 75 MHz, δ, ppm): 0.10 ($OSi\underline{C}H_3$), 23.84 ($\underline{C}H_2CH(CH_3)_2$), 24.13, 24.22 ($CH_2\underline{C}H(CH_3)_2$), 25.07, 25.77, 26.00, 26.19 ($CH_2CH(\underline{C}H_3)_2$), 89.62 ($\underline{C}{\equiv}CC_6H_5$, $\equiv\underline{C}CC_6H_5$), 115.11- 133.23 ($\underline{C}_6H_5$), 143.77 ($=\underline{C}(H)CC_6H_5$, $=\underline{C}C{\equiv}CC_6H_5$). **^{29}Si NMR** ($CDCl_3$, 79 MHz, δ, ppm): -67.30, -67.14, -67.02 (SiO_3), -1.99 ($OSi(CH_3)_2$). **FT IR (cm^{-1}):** 2953.6, 2902.7, 2869.5, 1600.1, 1504.1, 1464.8, 1401.9, 1365.9, 1331.5, 1252.2, 1229.0, 1156.0, 1073.7, 1031.9, 863.2, 830.7, 787.4, 735.4, 592.1, 526.6, 445.5. **MALDI TOF MS -** (m/z) ([M+Na], (%)): 1702.53.

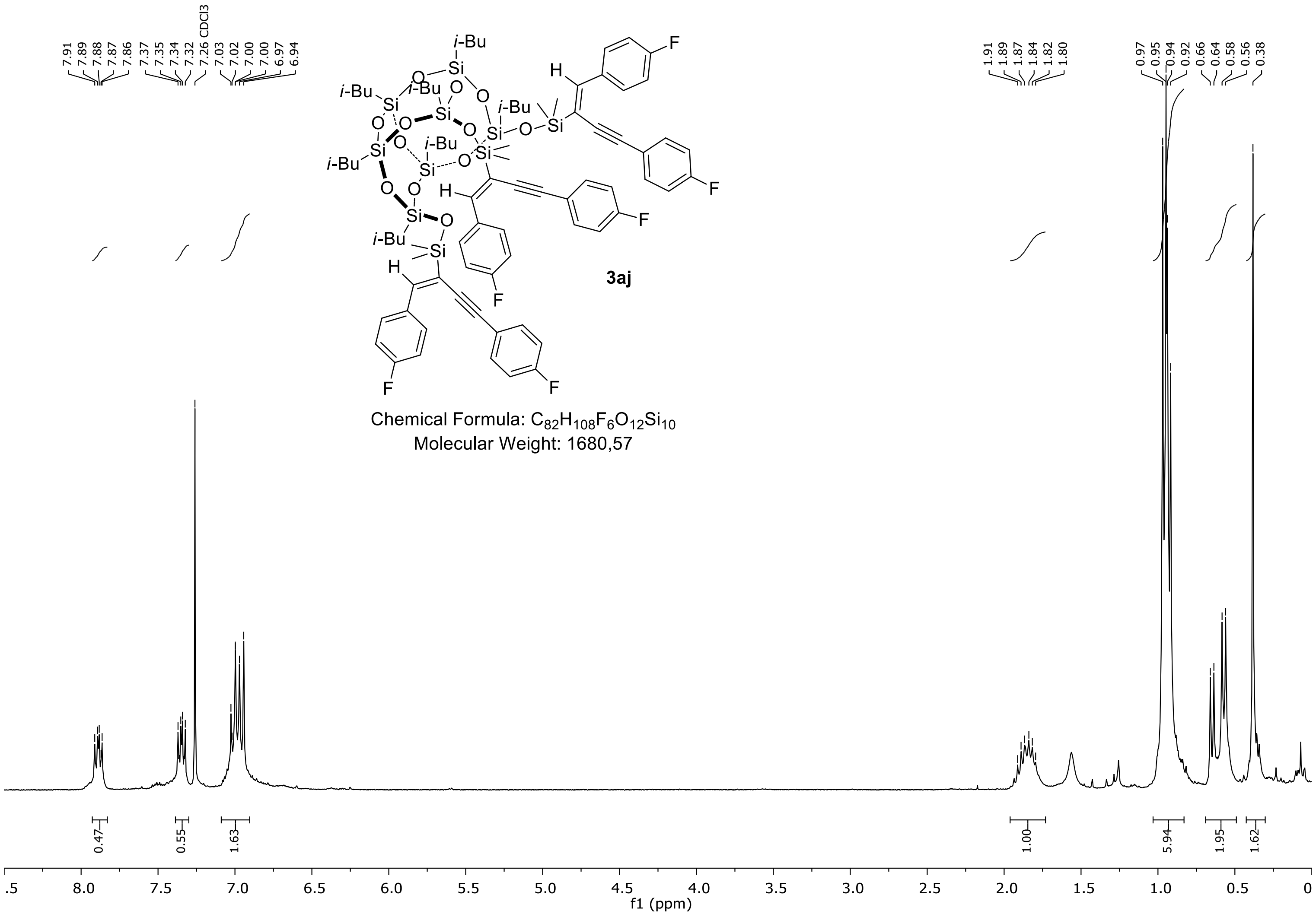

Figure S86. ^{1}H NMR of compound **3aj**.

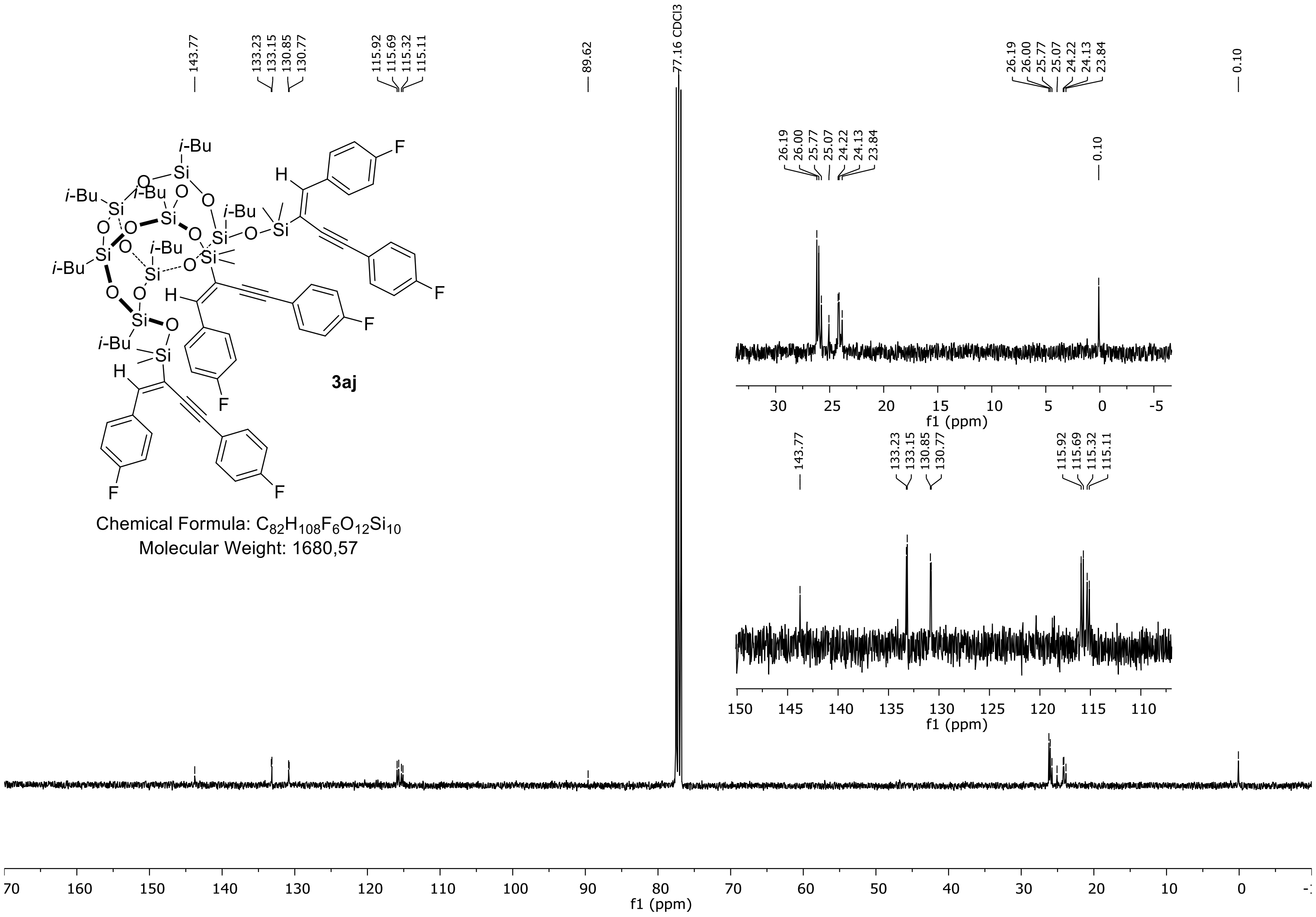

Figure S87. ^{13}C NMR of compound **3aj**.

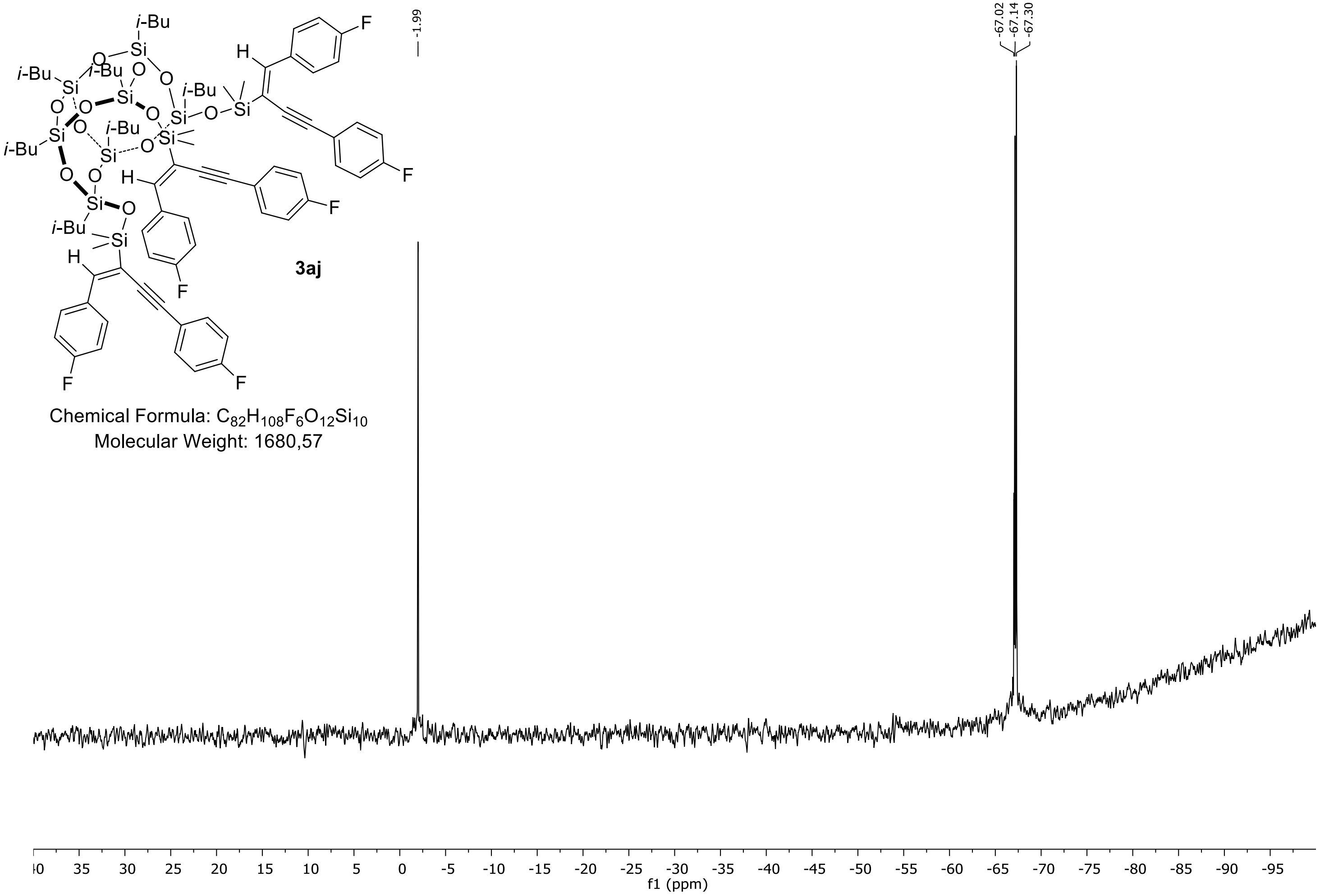

Figure S88. ^{29}Si NMR of compound **3aj**.

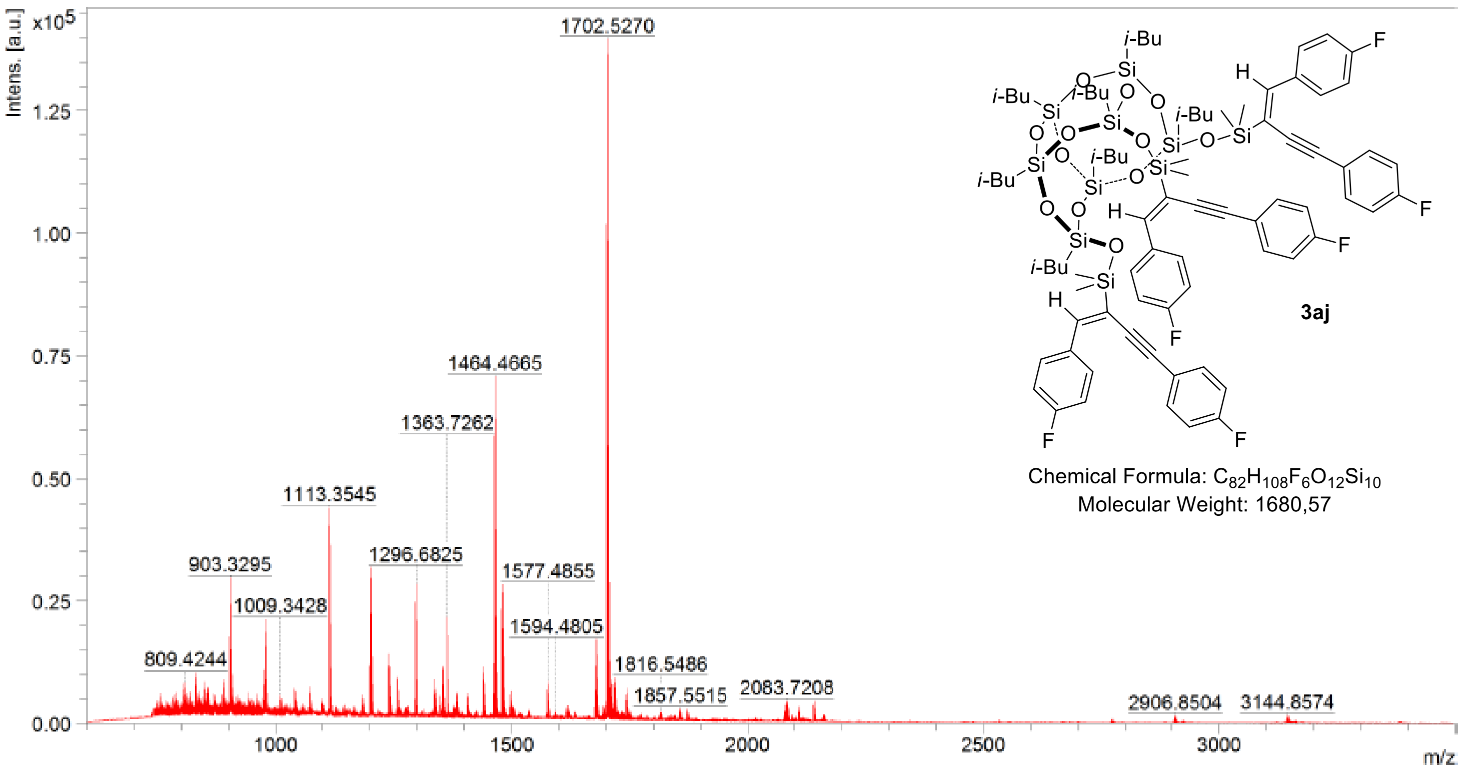

Figure S89. MALDI TOF MS spectra of compound **3aj**.

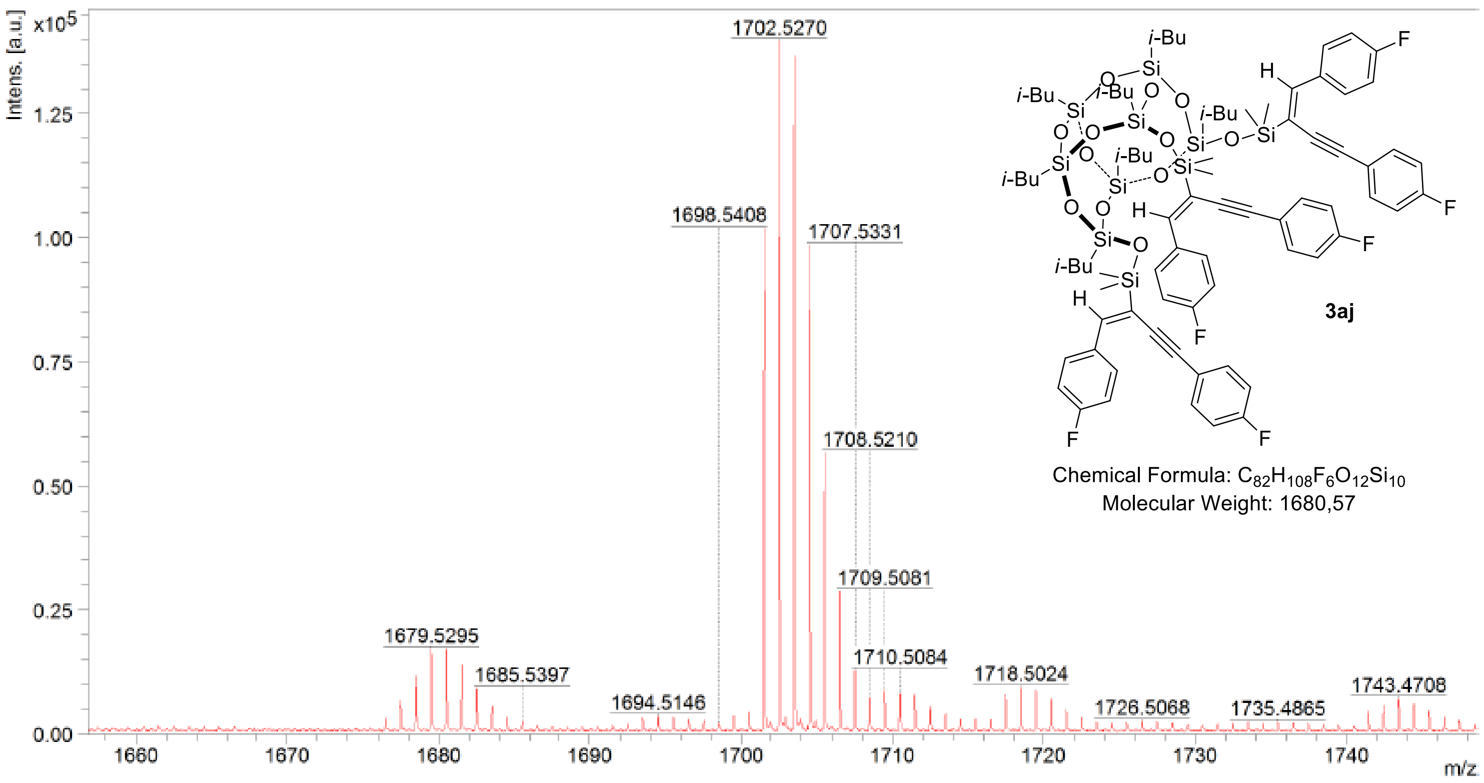

Figure S90. MALDI TOF MS spectra of compound **3aj**.

3ak

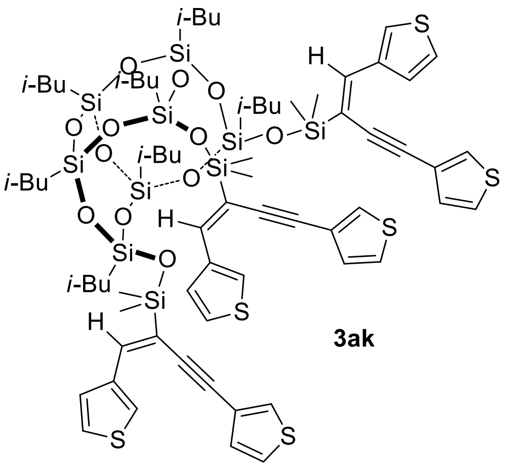

3ak

Chemical Formula: $C_{70}H_{102}O_{12}S_6Si_{10}$
Molecular Weight: 1608,80

Isolated yield = 88%, colorless oil.

^{1}**H NMR** ($CDCl_3$, 300 MHz, δ, ppm): 0.38 (s, 9H, $SiC\underline{H}_3$), 0.55 – 0.65 (m, 14H, $C\underline{H}_2$), 0.92-0.97 (m, 42H, $C\underline{H}_3$), 1.81-1.87 (m, 21H, $C\underline{H}$), 7.03 (s, 3H, $=C\underline{H}$), 6.94-7.91 (m, 18H, $C_4\underline{H}_3S$). ^{13}**C NMR** ($CDCl_3$, 75 MHz, δ, ppm): 0.16 ($OSi\underline{C}H_3$), 23.82 ($\underline{C}H_2CH(CH_3)_2$), 23.98, 24.10, 24.20, 25.06 ($CH_2\underline{C}H(CH_3)_2$), 25.77, 26.01, 26.20 ($CH_2CH(\underline{C}H_3)_2$), 73.65 ($\underline{C}{\equiv}CC_4H_3S$, **2k**), 76.69 (${\equiv}\underline{C}C_4H_3S$, **2k**), 90.29 ($\underline{C}{\equiv}CC_4H_3S$), 95.48 (${\equiv}\underline{C}C_4H_3S$), 123.55- 131.38 ($\underline{C}_4H_3S$), 138.77 ($=\underline{C}C{\equiv}CC_4H_3S$), 140.50 ($=\underline{C}(H)CC_4H_3S$). ^{29}**Si NMR** ($CDCl_3$, 79 MHz, δ, ppm): -67.33, -67.19 (SiO_3), -1.78 ($OSi(CH_3)_2$). **FT IR (cm^{-1}):** 2952.9, 2913.1, 2868.7, 1666.9, 1539.0, 1463.0, 1365.2, 1332.4, 1252.2, 1227.7, 1076.4, 927.7, 867.2, 837.9, 816.9, 776.4, 687.7, 638.4, 620.6, 451.9. **MALDI TOF MS -** (m/z) ([M+Na], (%)): 1631.33.

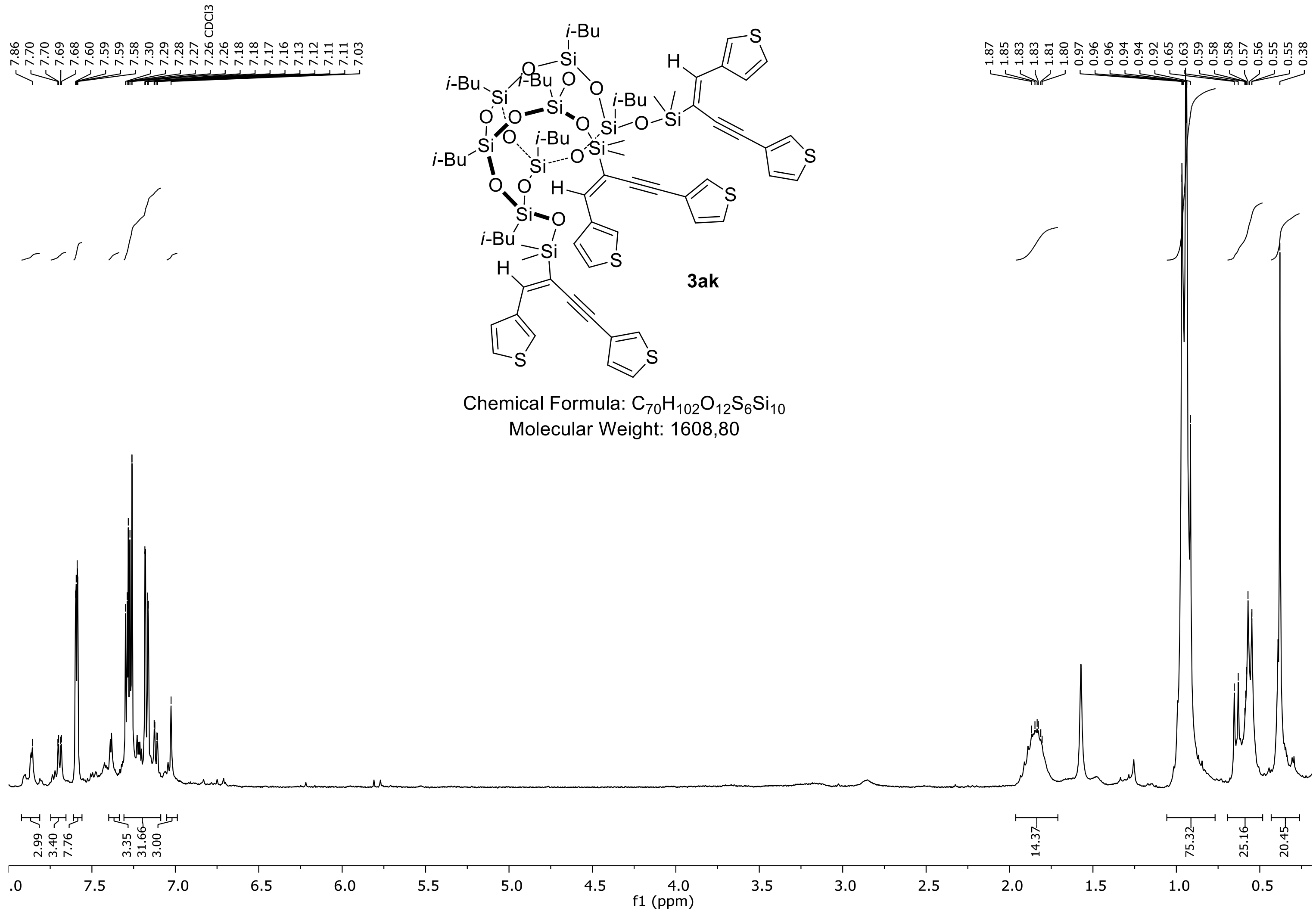

Figure S91. ^{1}H NMR of compound **3ak**.

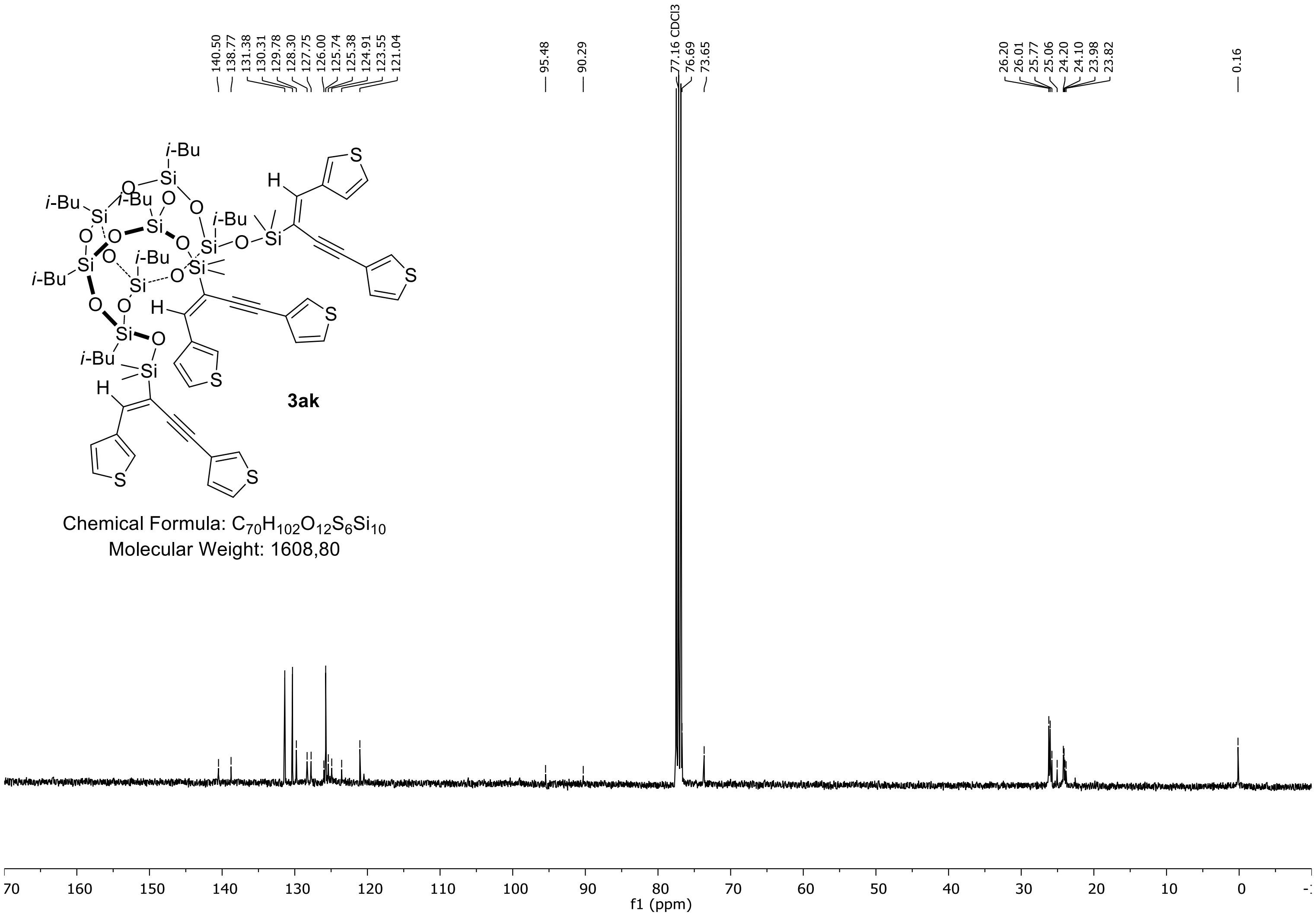

Figure S92. ^{13}C NMR of compound **3ak**.

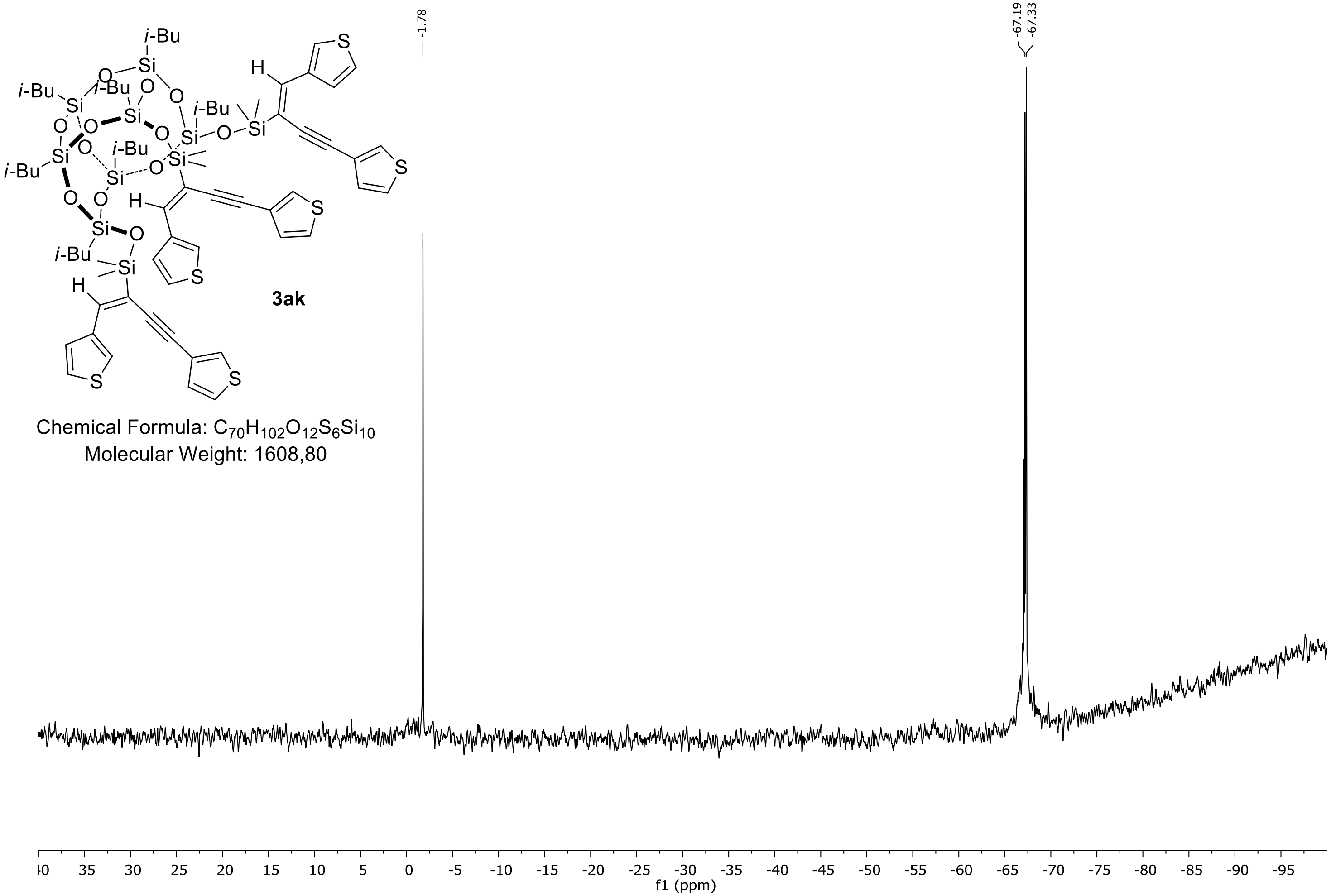

Figure S93. ^{29}Si NMR of compound **3ak**.

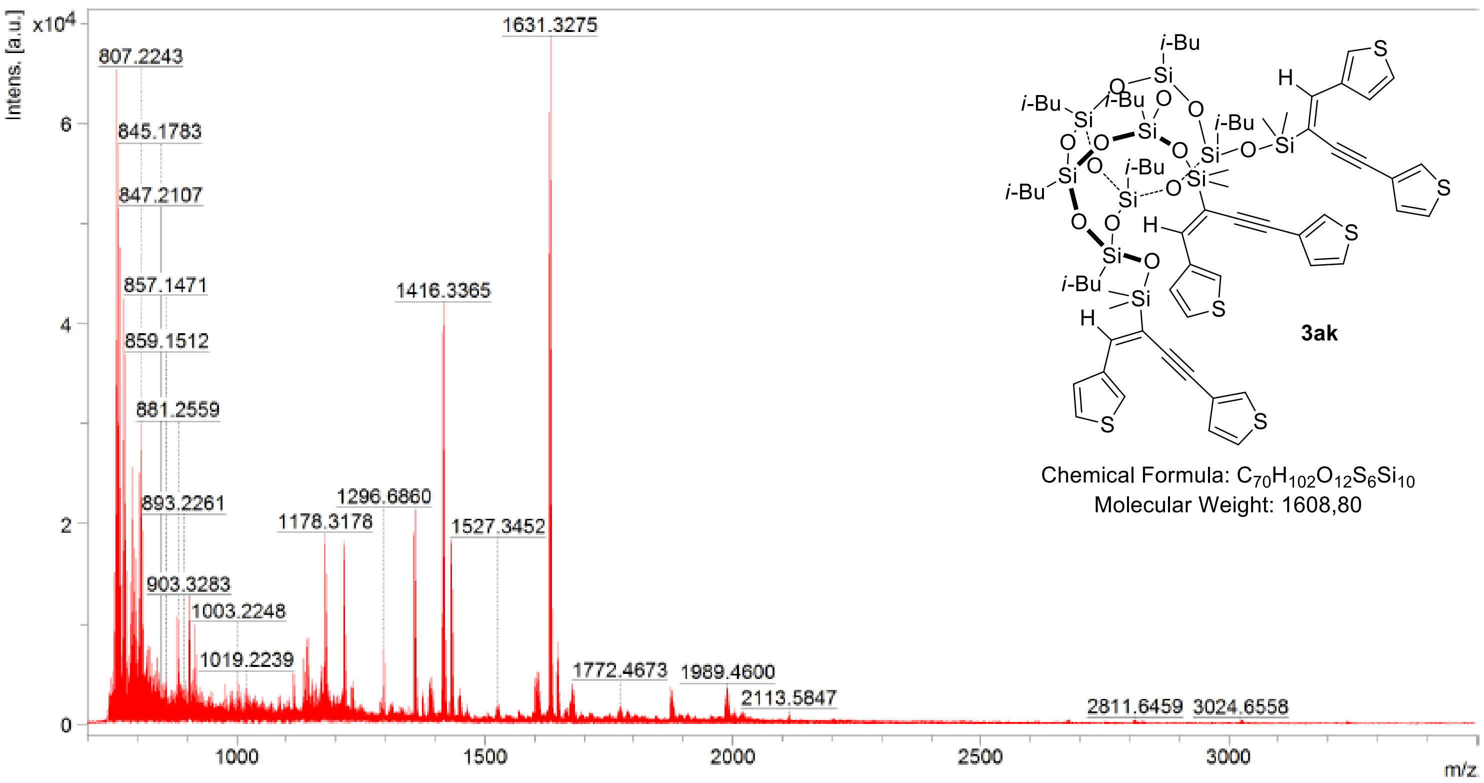

Figure S94. MALDI TOF MS spectra of compound **3ak**.

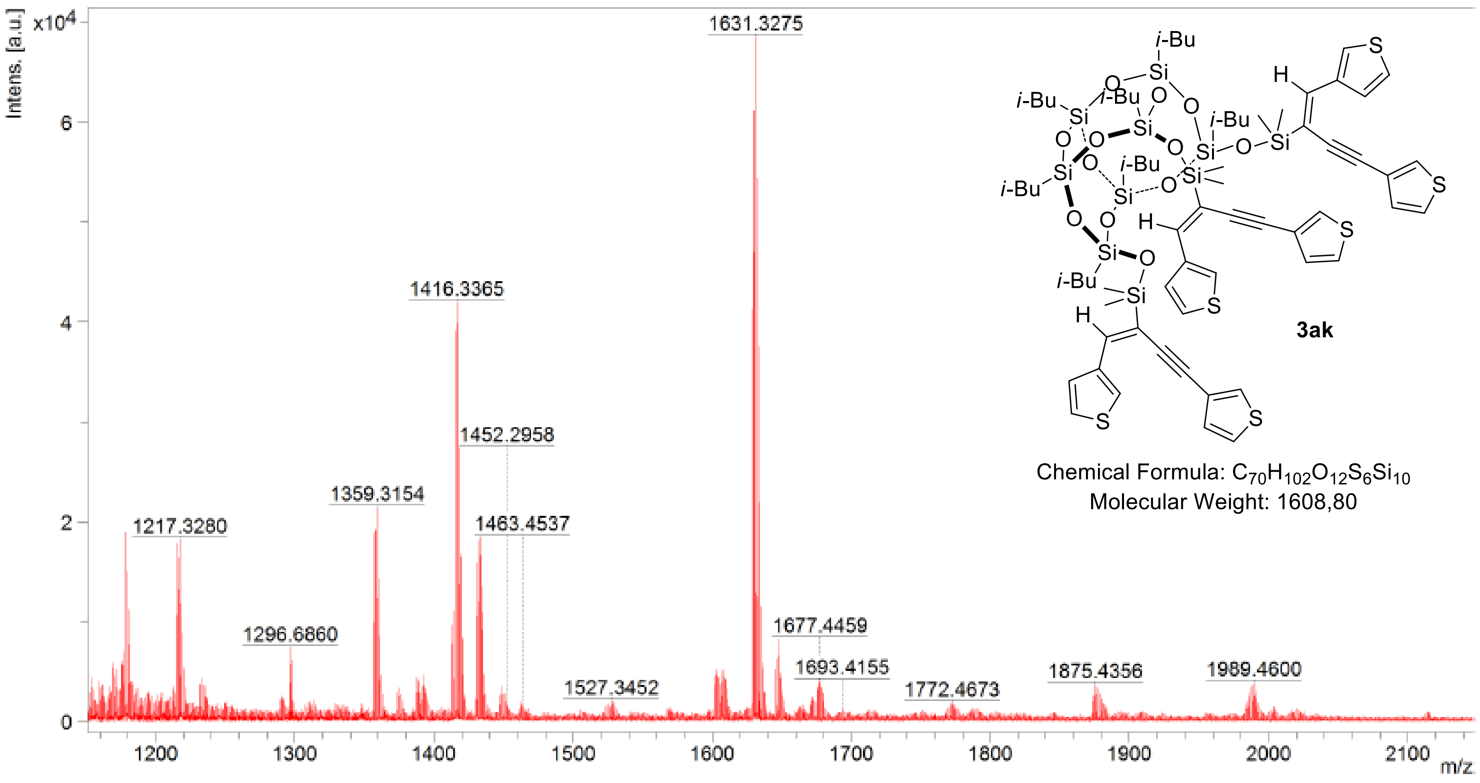

Figure S95. MALDI TOF MS spectra of compound **3ak**.

3al

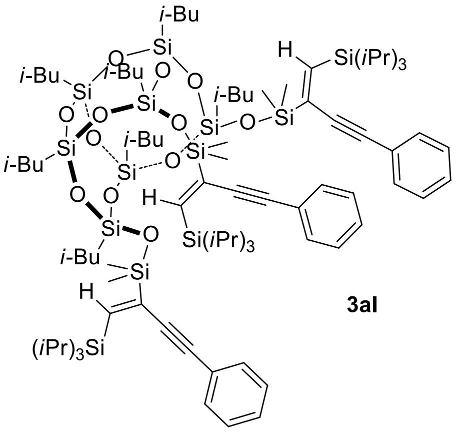

3al

Chemical Formula: $C_{91}H_{162}O_{12}Si_{13}$
Molecular Weight: 1813,36

Isolated yield = 90%, colorless oil.

^{1}H NMR ($CDCl_3$, 300 MHz, δ, ppm): 0.34 (s, 9H, SiCH_3), 0.54–0.64 (m, 14H, CH_2), 0.93-0.96 (m, 42H, CH_3), 1.06-1.08 (m, 63H, $CH(CH_3)_2$, $CH(CH_3)_2$), 1.82-1.89 (m, 21H, CH), 6.97 (s, 3H, =CH), 7.22-7.99 (m, 15H, C_6H_5). **^{13}C NMR** ($CDCl_3$, 75 MHz, δ, ppm): 0.06 (OSiCH_3), 11.65 ($Si(CH(CH_3)_2)_3$), 18.85 ($Si(CH(CH_3)_2)_3$), 22.59 ($CH_2CH(CH_3)_2$), 23.90, 24.02, 24.23 ($CH_2CH(CH_3)_2$), 25.13, 25.79, 26.02, 26.04, 26.08, 26.25 ($CH_2CH(CH_3)_2$), 103.70 ($C≡CC_6H_5$), 107.37 ($C≡CC_6H_5$), 123.02, 128.12, 128.60, 129.18 (C_6H_5), 137.80 ($=CC≡CC_6H_5$), 145.77 (=C(H)Si(iPr)$_3$). **^{29}Si NMR** ($CDCl_3$, 79 MHz, δ, ppm): -67.37, -67.24 (SiO_3), -2.93 ($OSi(CH_3)_2$), -1.94 (Si(iPr)$_3$). **FT IR (cm^{-1}):** 2953.4, 2892.7, 2865.7, 1493.5, 1463.6, 1401.2, 1382.6, 1365.8, 1331.5, 1252.4, 1228.0, 1077.1, 1046.8, 920.7, 882.2, 844.2, 786.0, 751.6, 689.1, 676.1, 658.4, 623.2, 604.2, 451.7. **MALDI TOF MS** - (m/z) ([M+Na], (%)): 1835.91.

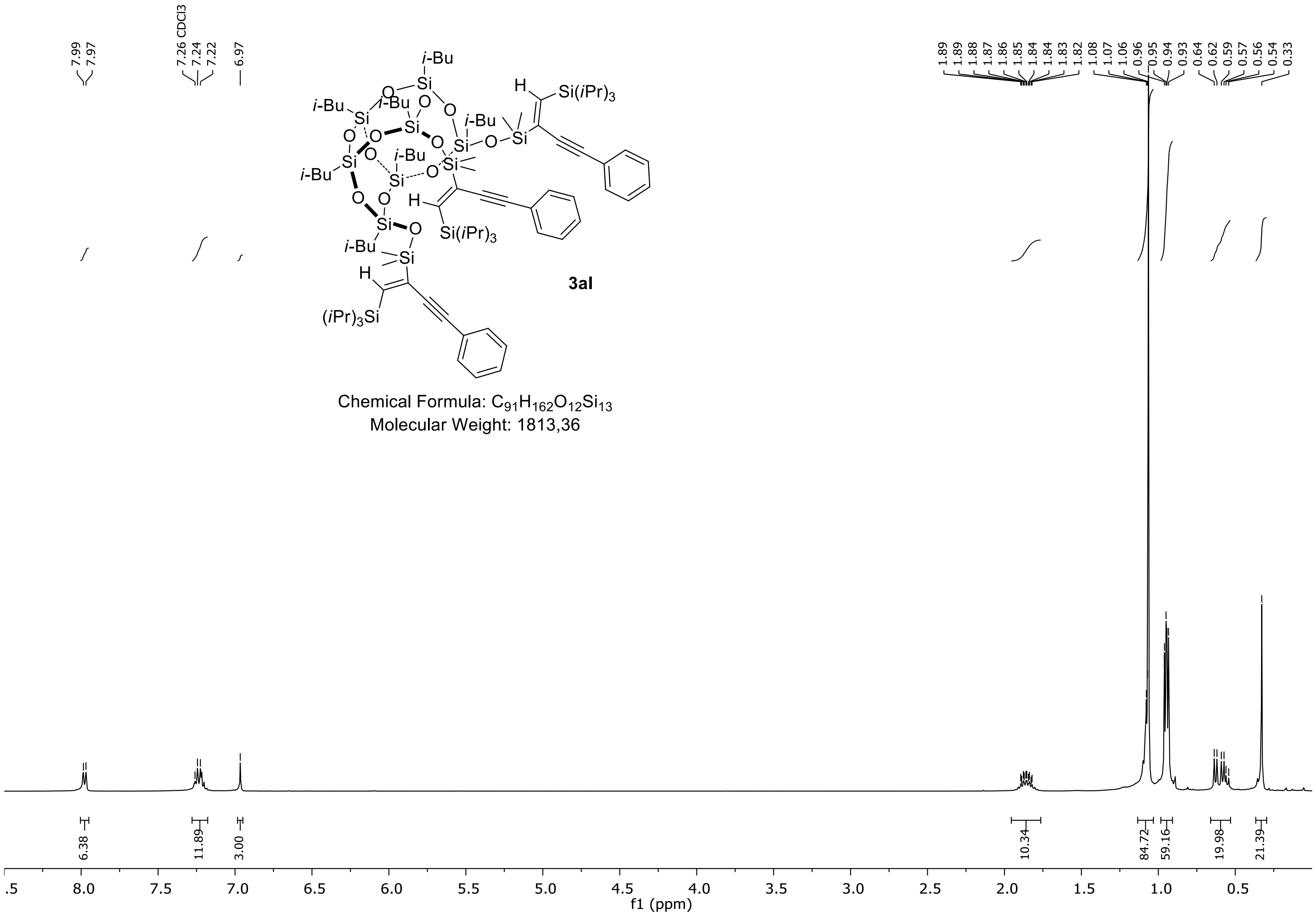

Figure S96. ^{1}H NMR of compound **3al**.

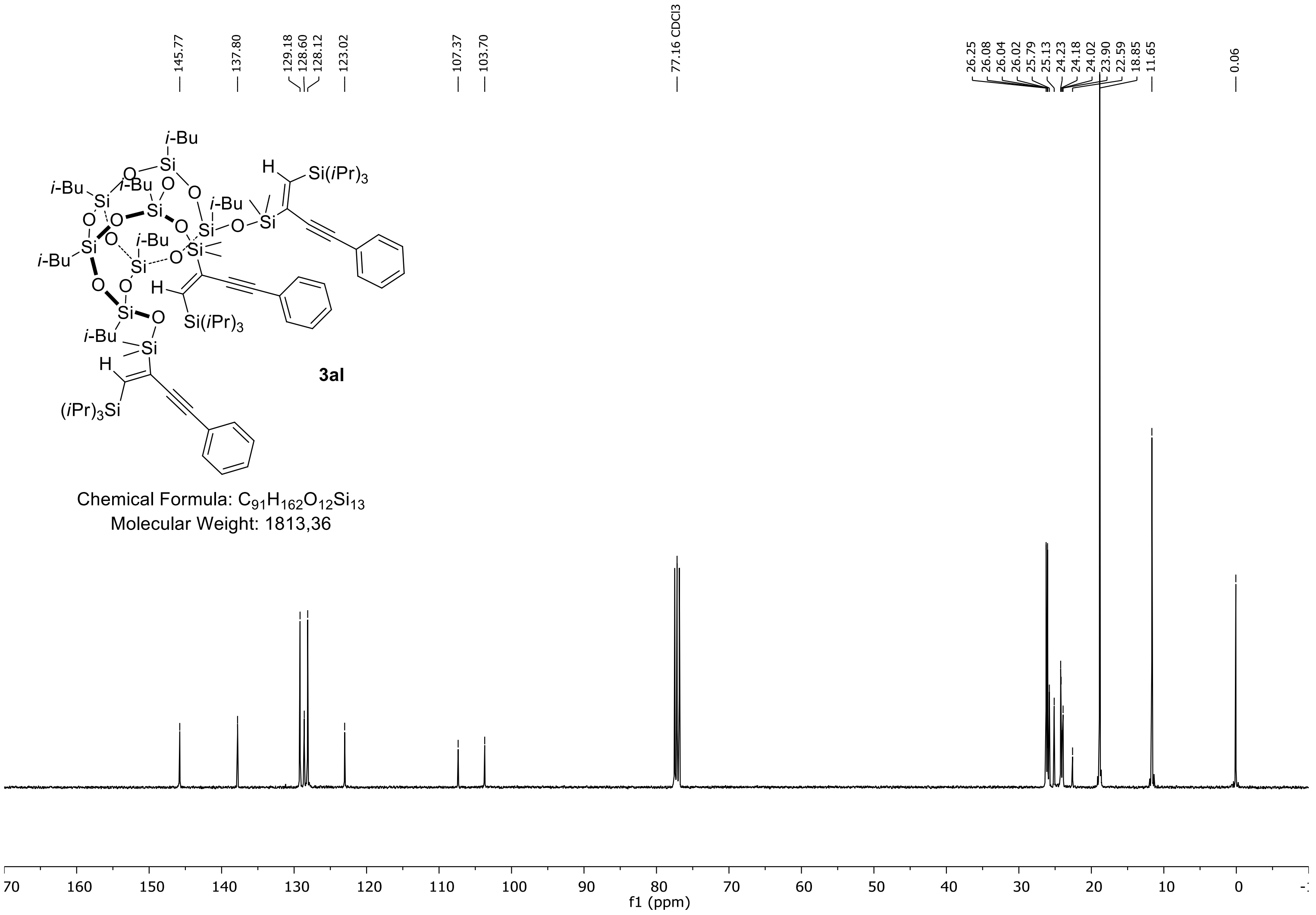

Figure S97. ^{13}C NMR of compound **3al**.

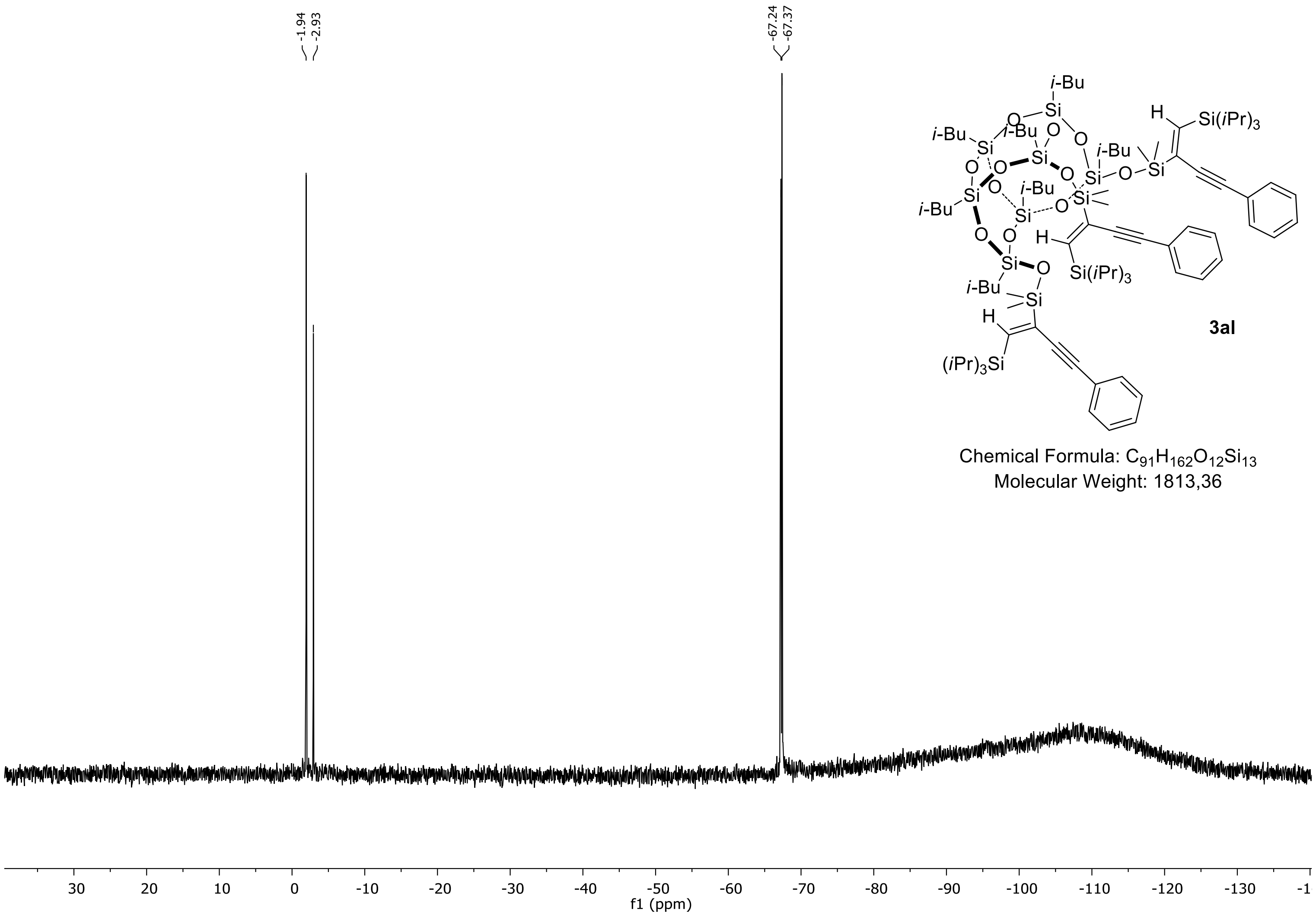

Figure S98. ^{29}Si NMR of compound **3al**.

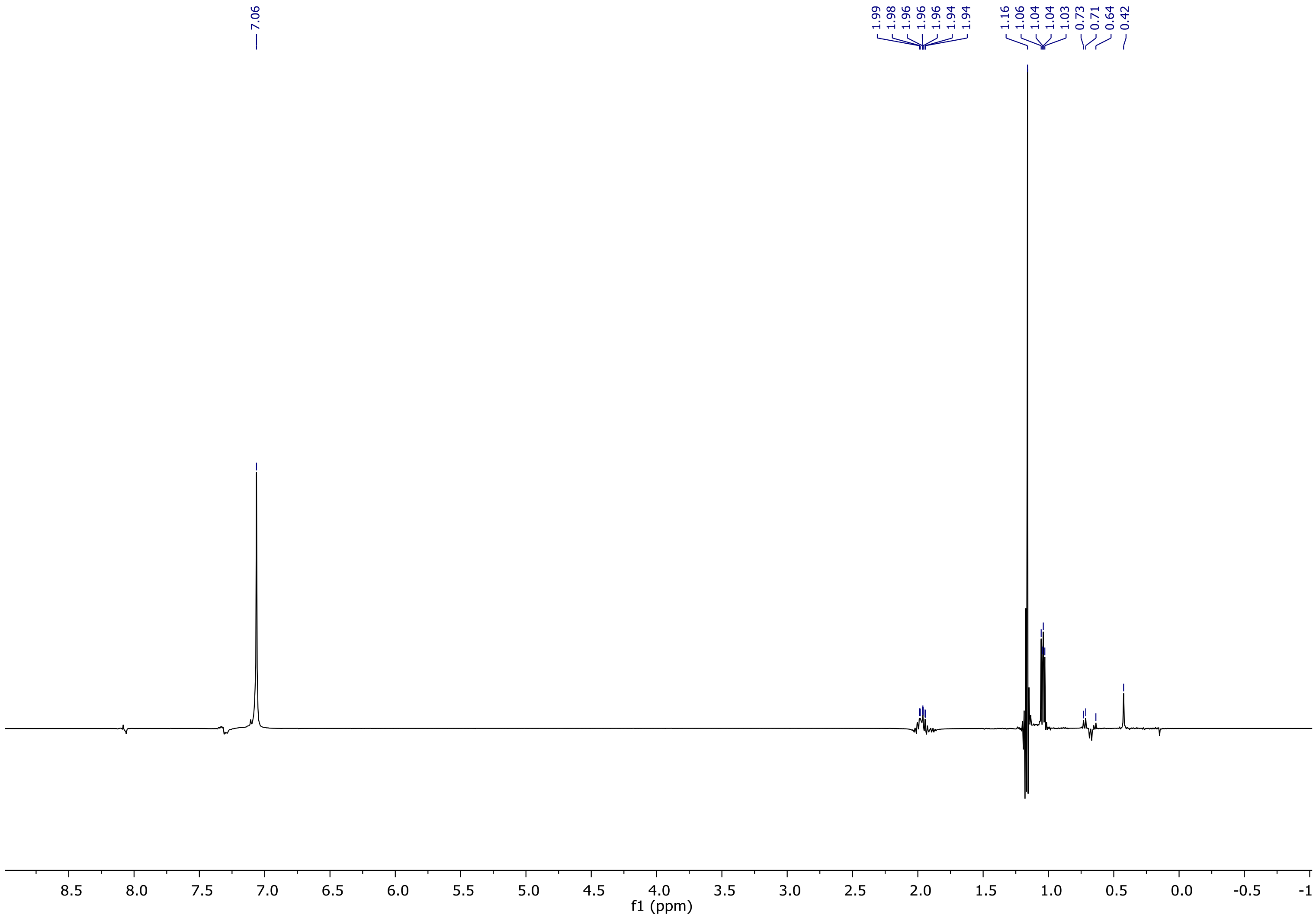

Figure S99. 1D selective gradient NOESY NMR of compound **3al** directed at alkenyl proton at 7.06 ppm.

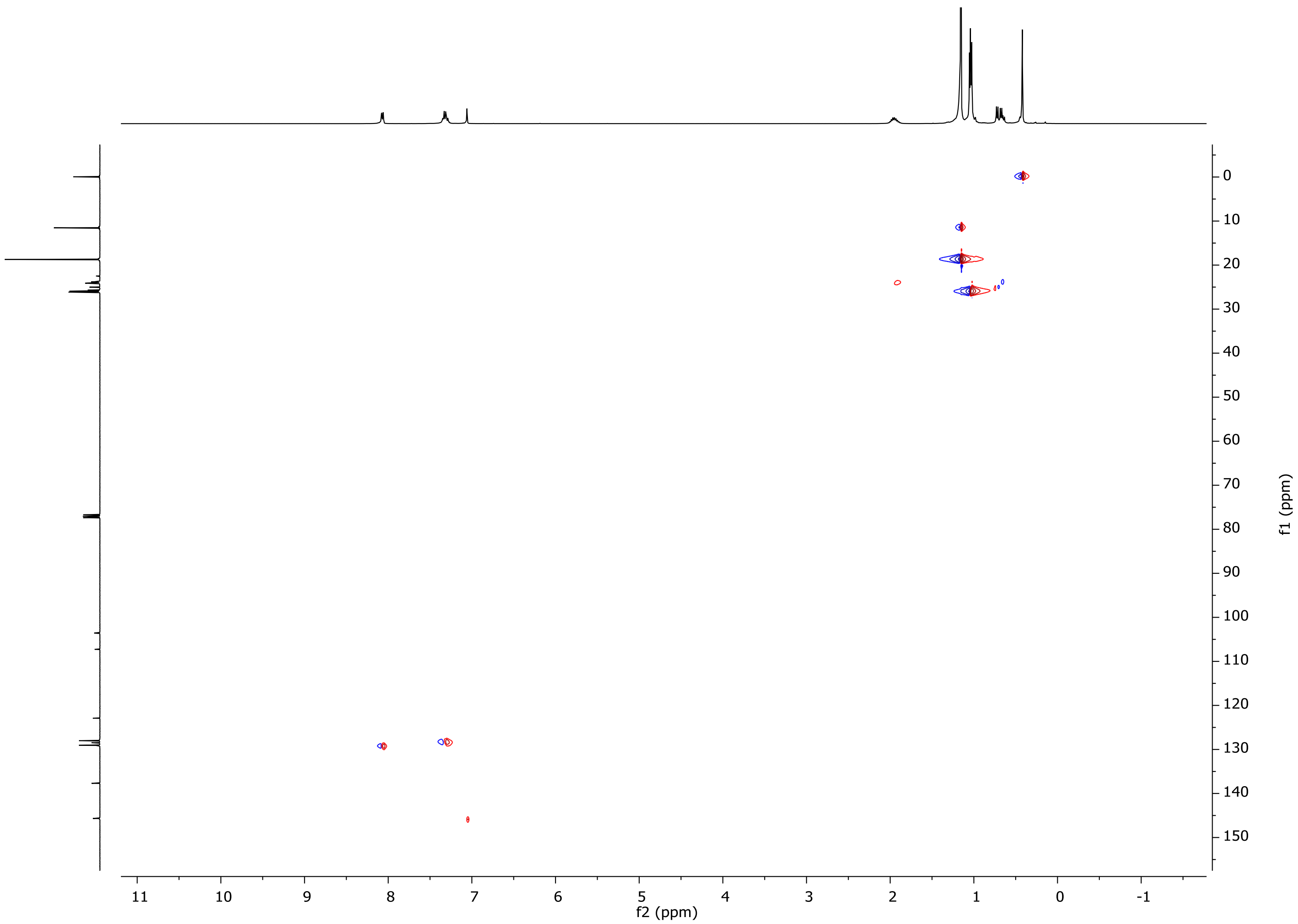

Figure S100. ^{1}H-^{13}C HSQC NMR of compound **3al**.

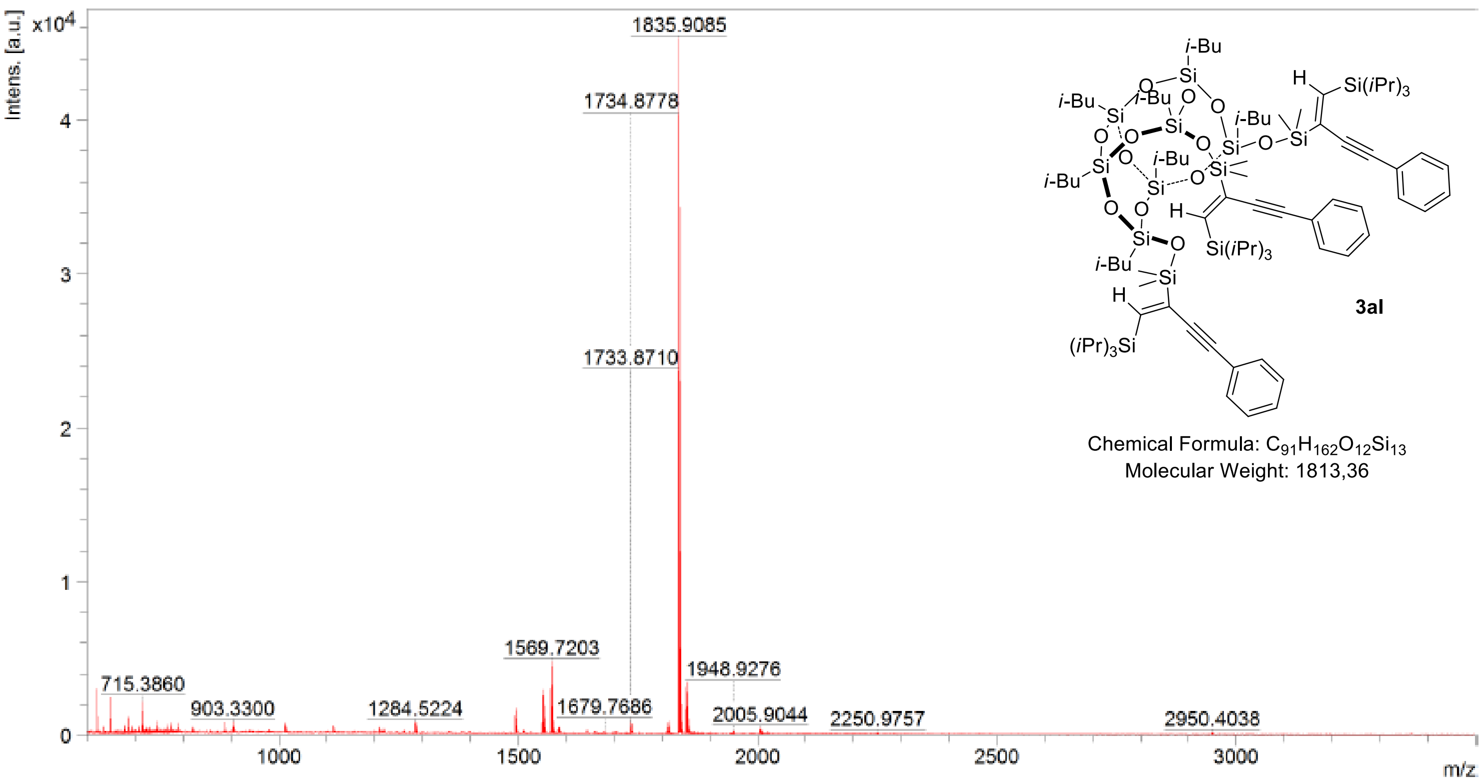

Figure S101. MALDI TOF MS spectra of compound **3al**.

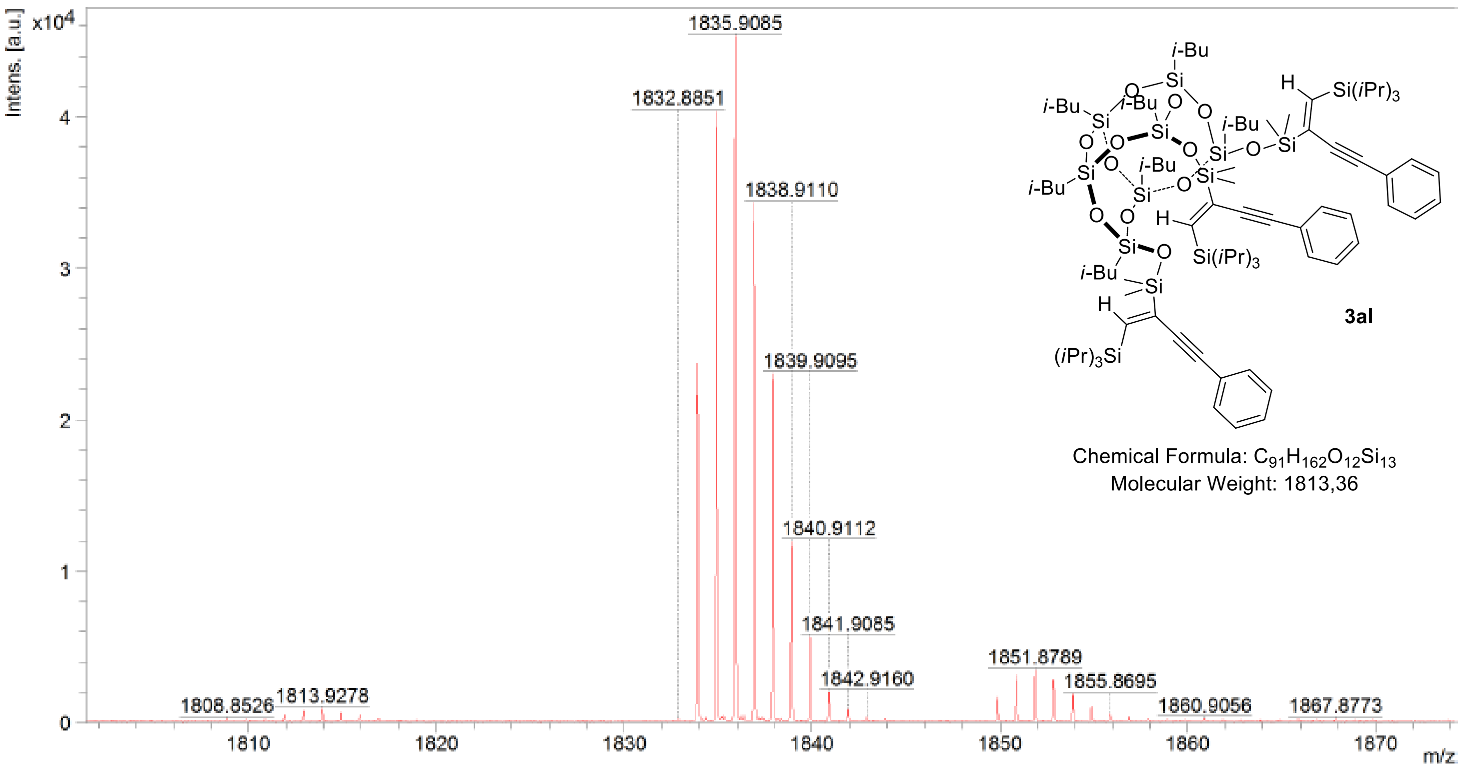

Figure S102. MALDI TOF MS spectra of compound **3al**.

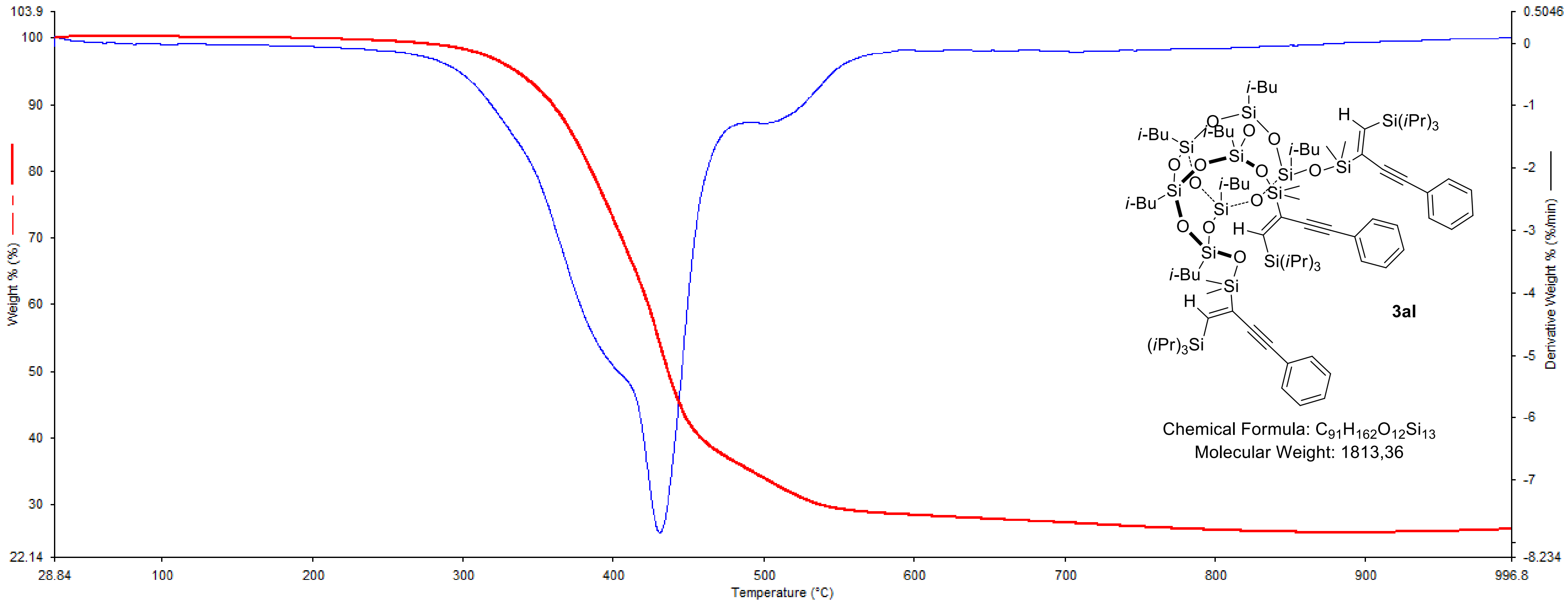

Figure S103. TGA/DTG curves of compound **3al**.

3am

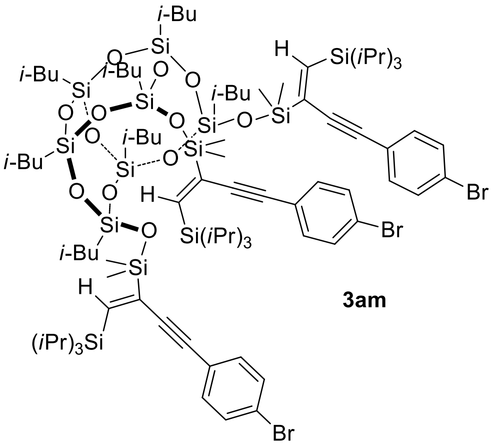

3am

Chemical Formula: $C_{91}H_{159}Br_3O_{12}Si_{13}$
Molecular Weight: 2050,05

Isolated yield = 97%, colorless oil.

^{1}**H NMR** ($CDCl_3$, 300 MHz, δ, ppm): 0.34 (s, 9H, $SiC\underline{H}_3$), 0.60–0.66 (m, 14H, $C\underline{H}_2$), 0.97-0.99 (m, 42H, $C\underline{H}_3$), 1.09-1.12 (m, 63H, $CH(C\underline{H}_3)_2$, $C\underline{H}(CH_3)_2$), 1.83-1.92 (m, 21H, $C\underline{H}$), 6.90 (s, 3H, $=C\underline{H}$), 7.38 (d, 6H, $J_{(H,H)}$= 8.54 Hz, $C_6\underline{H}_4Br$), 7.87 (d, 6H, $J_{(H,H)}$= 8.53 Hz, $C_6\underline{H}_4Br$). ^{13}**C NMR** ($CDCl_3$, 75 MHz, δ, ppm): 0.02 ($OSi\underline{C}H_3$), 11.44, 11.60, 11.62, 11.64 ($Si(CH(\underline{C}H_3)_2)_3$), 18.60, 18.72, 18.76, 18.83, 18.85, 18.86 ($Si(\underline{C}H(CH_3)_2)_3$), 22.54 ($\underline{C}H_2CH(CH_3)_2$), 23.87, 24.00, 24.19, 24.23 ($CH_2\underline{C}H(CH_3)_2$), 25.09, 25.78, 26.04, 26.23 ($CH_2CH(\underline{C}H_3)_2$), 105.07 ($\underline{C}\equiv CC_6H_4Br$), 106.92 ($C\equiv\underline{C}C_6H_4Br$), 122.47, 124.01, 130.57, 131.29, 131.90, 134.16 ($\underline{C}_6H_4Br$), 136.57 ($=\underline{C}C\equiv CC_6H_4Br$), 144.15 ($=\underline{C}(H)Si(iPr)_3$). ^{29}**Si NMR** ($CDCl_3$, 79 MHz, δ, ppm): -67.30, -67.12, -67.04 (SiO_3), -2.68 ($OSi(CH_3)_2$), -2.01 ($Si(iPr)_3$). **FT IR (cm^{-1}):** 2953.1, 2865.6, 1584.7, 1485.8, 1463.4, 1401.2, 1382.5, 1365.7, 1331.4, 1252.3, 1227.9, 1071.4, 1044.9, 1009.7, 882.0, 840.6, 785.8, 737.5, 676.1, 658.3, 633.1, 600.4, 448.7. **MALDI TOF MS -** (m/z) ([M+Na], (%)): 2072.63.

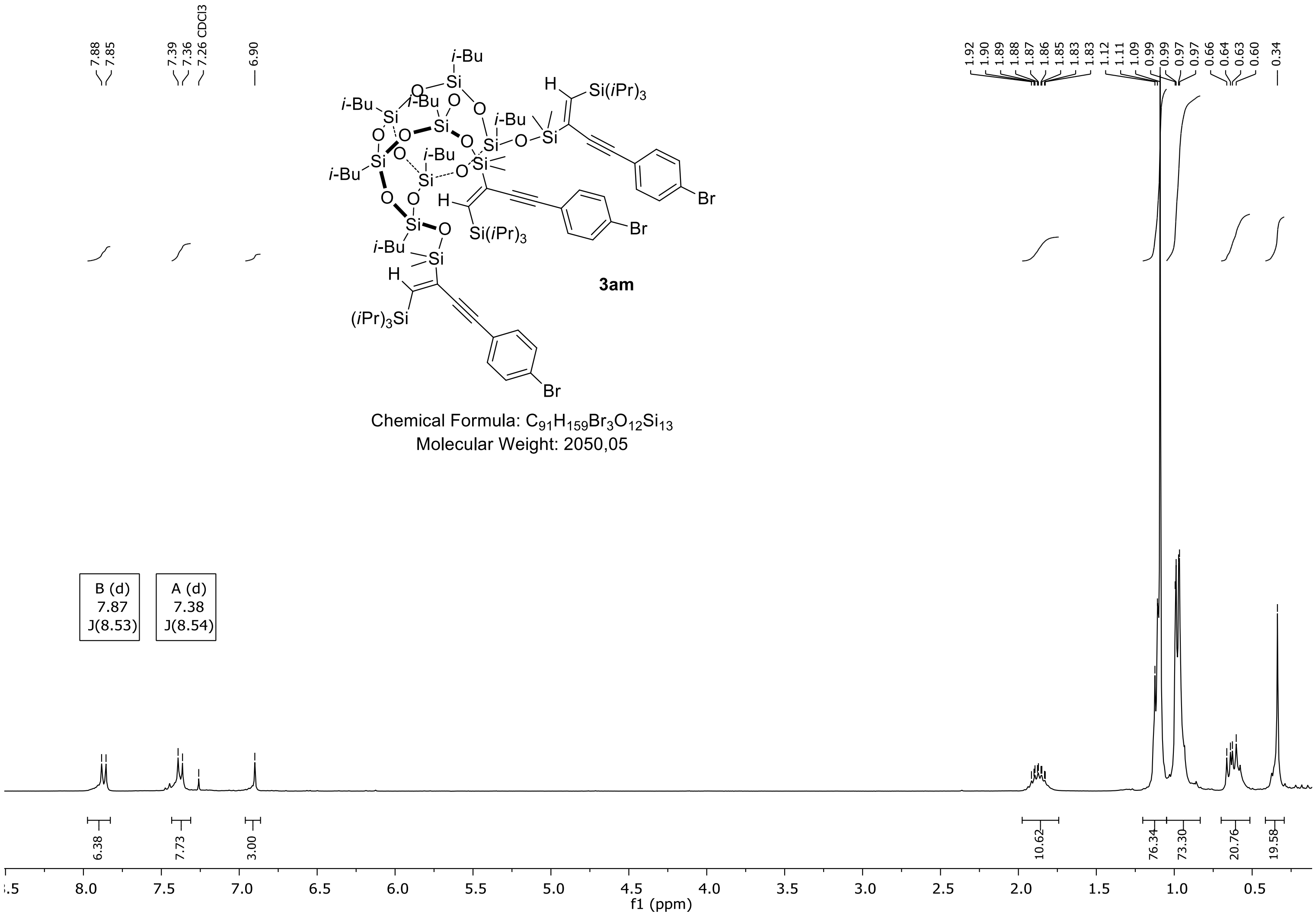

Figure S104. ^{1}H NMR of compound **3am**.

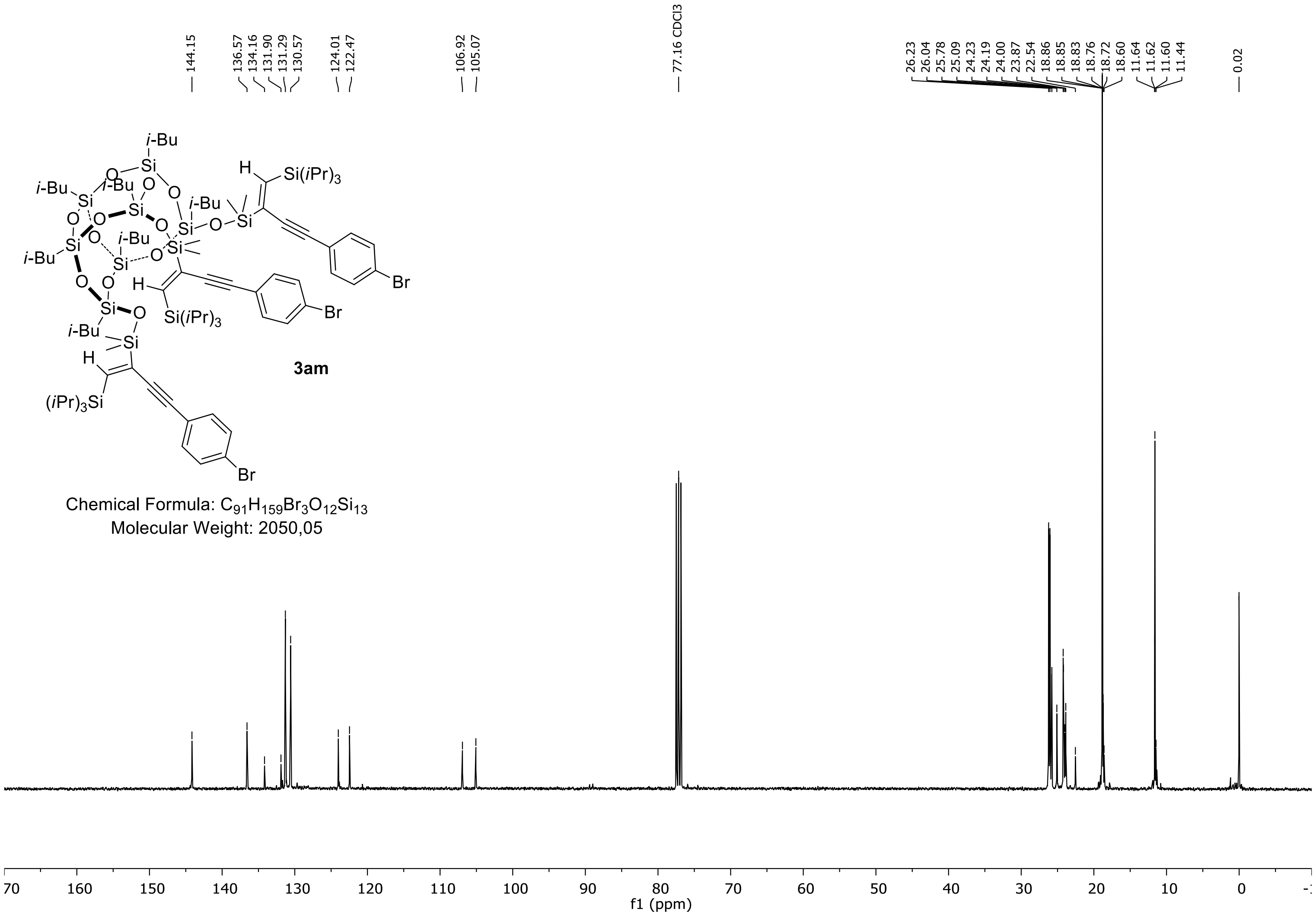

Figure S105. ^{13}C NMR of compound **3am**.

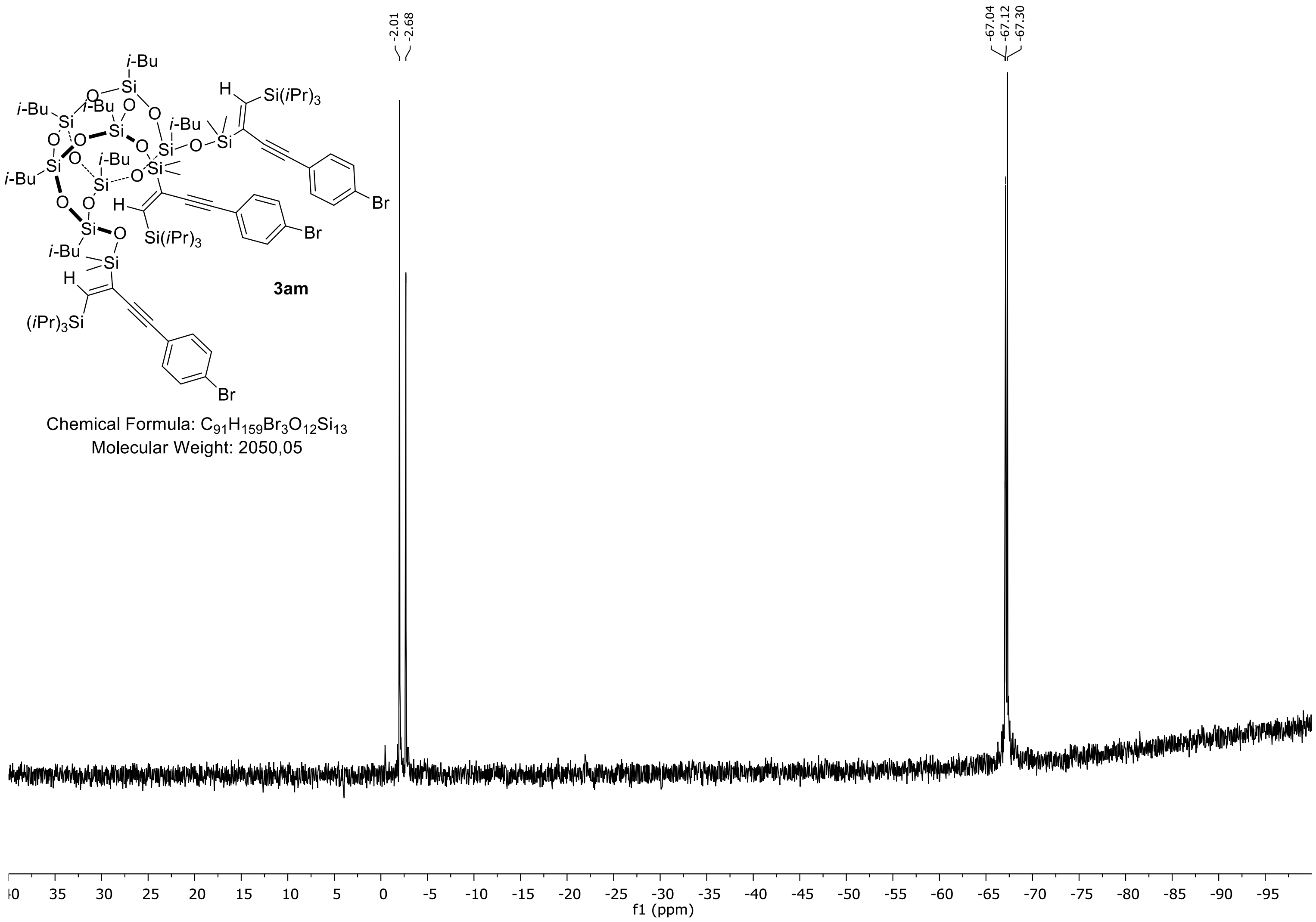

Figure S106. ^{29}Si NMR of compound **3am**.

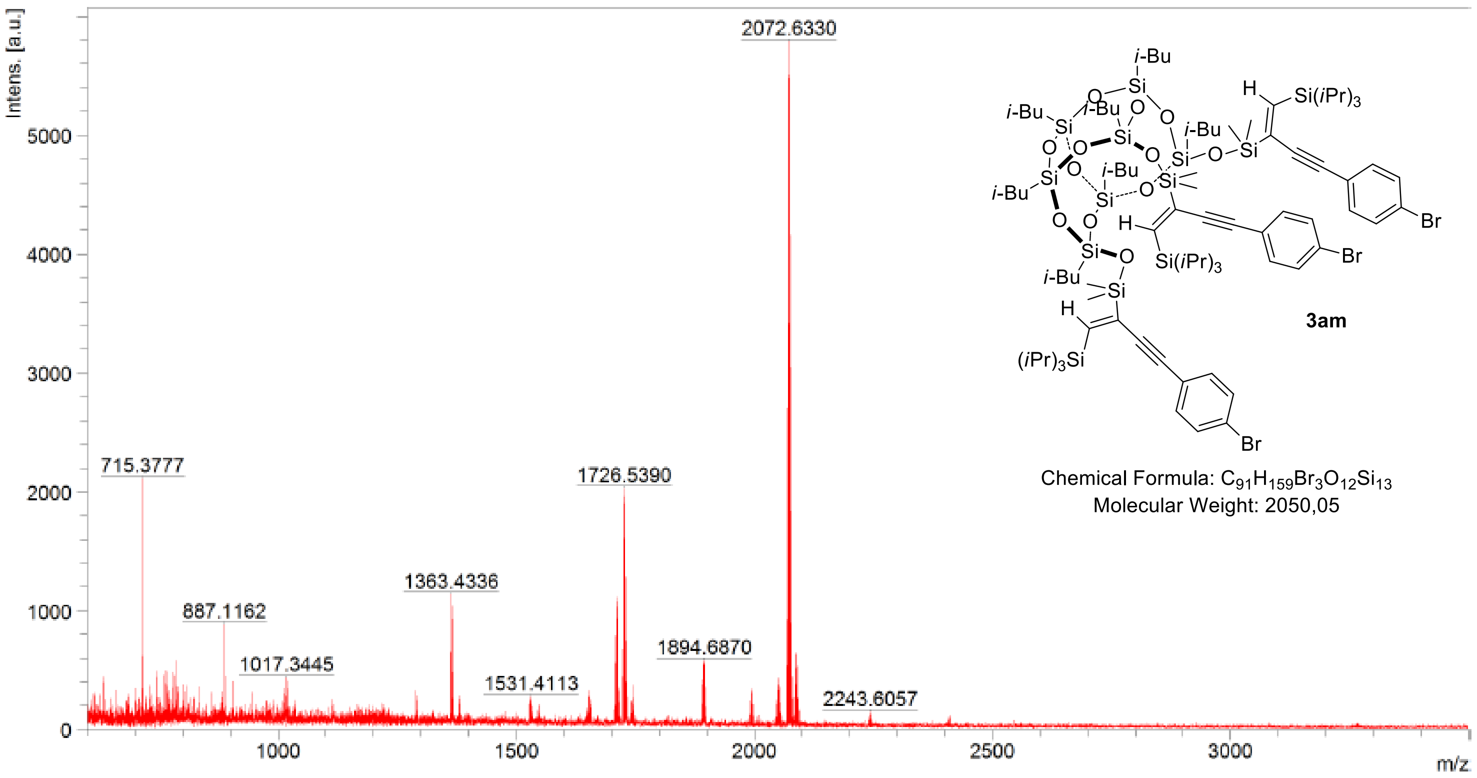

Figure S107. MALDI TOF MS spectra of compound **3am**.

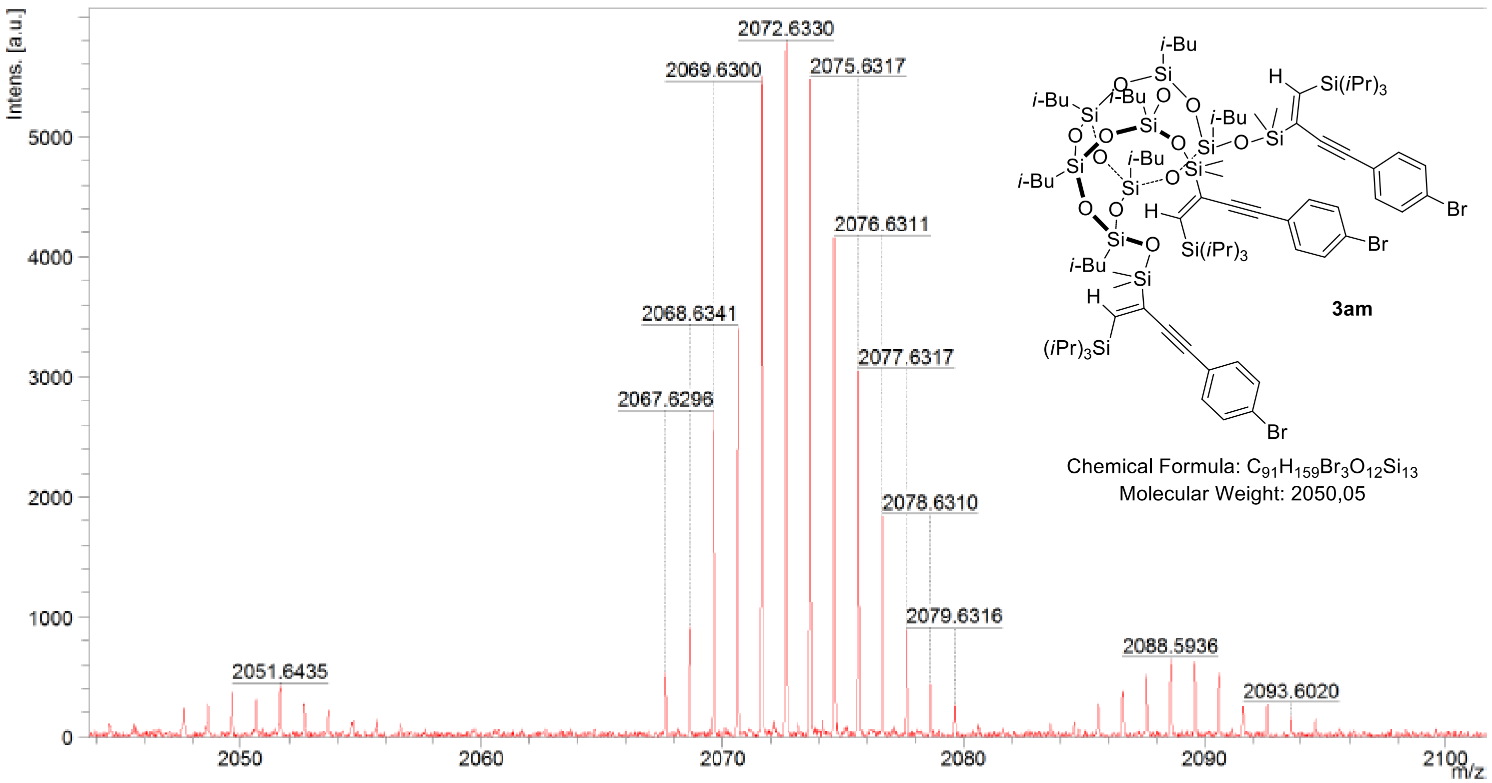

Figure S108. MALDI TOF MS spectra of compound **3am**.

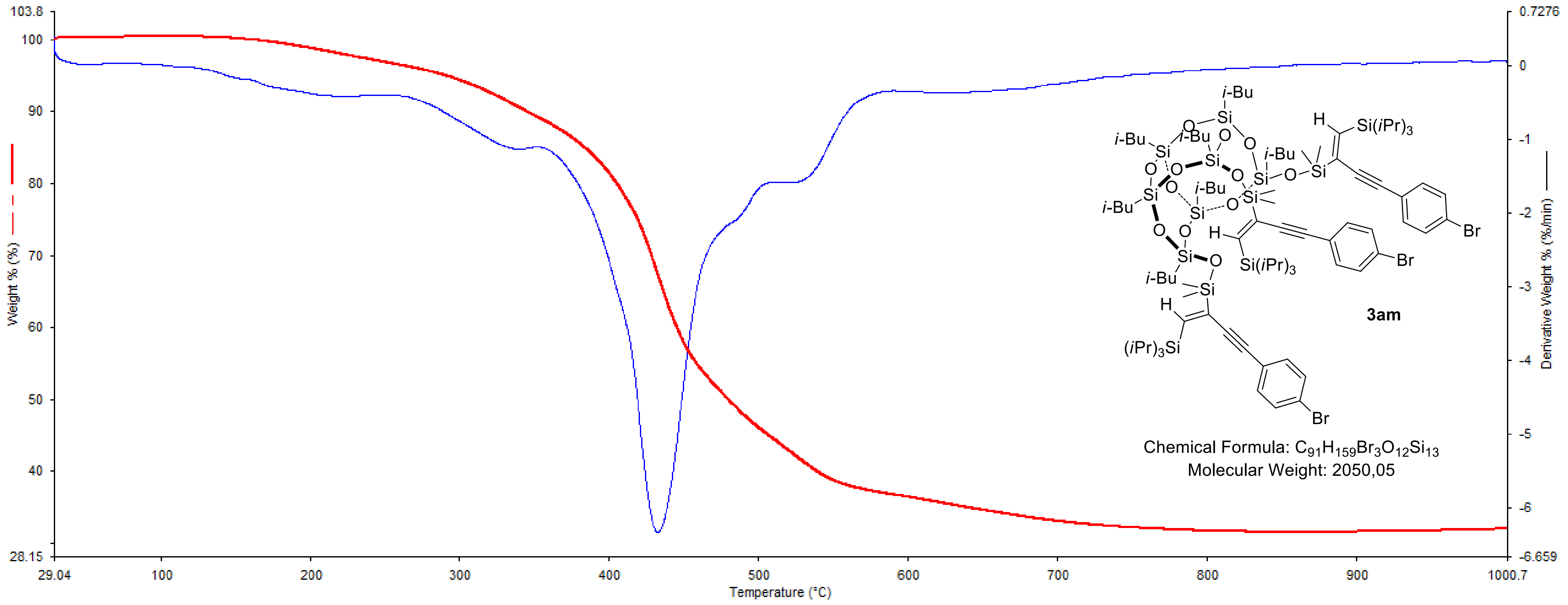

Figure S109. TGA/DTG curves of compound **3am**.

3an

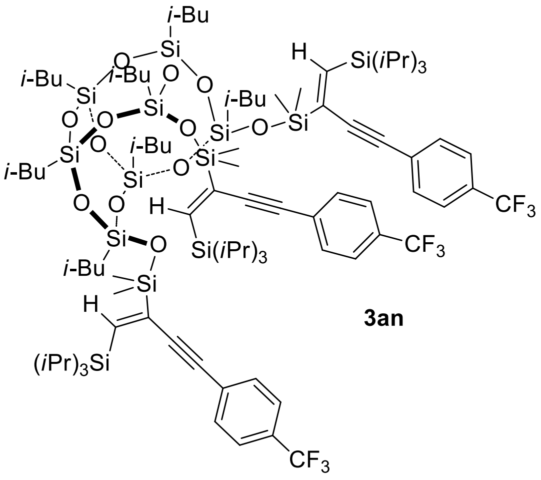

3an

Chemical Formula: $C_{94}H_{159}F_9O_{12}Si_{13}$
Molecular Weight: 2017,36

Isolated yield = 98%, colorless oil.

^{1}H NMR ($CDCl_3$, 300 MHz, δ, ppm): 0.37 (s, 9H, $SiC\underline{H}_3$), 0.62-0.69 (m, 14H, $C\underline{H}_2$), 0.99-1.02 (m, 42H, $C\underline{H}_3$), 1.10-1.14 (m, 63H, $CH(C\underline{H}_3)_2$, $C\underline{H}(CH_3)_2$), 1.86-1.94 (m, 21H, $C\underline{H}$), 7.02 (s, 3H, $=C\underline{H}$), 7.50 (d, 6H, $J_{(H,H)}$= 8.29 Hz, $C_6\underline{H}_4CF_3$), 8.07 (d, 6H, $J_{(H,H)}$= 8.24 Hz, $C_6\underline{H}_4CF_3$). **^{13}C NMR** ($CDCl_3$, 75 MHz, δ, ppm): 0.02 ($OSi\underline{C}H_3$), 11.59 ($Si(CH(\underline{C}H_3)_2)_3$), 18.79 ($Si(\underline{C}H(CH_3)_2)_3$), 22.56 ($\underline{C}H_2CH(CH_3)_2$), 23.89, 24.04, 24.24, 24.28 ($CH_2\underline{C}H(CH_3)_2$), 25.13, 25.79, 26.05, 26.24, 27.10 ($CH_2CH(\underline{C}H_3)_2$), 105.86 ($\underline{C}{\equiv}CC_6H_4CF_3$), 106.61 ($C{\equiv}\underline{C}C_6H_4CF_3$), 122.89, 125.07, 125.11, 125.60, 126.50, 129.10, 129.93, 130.25 ($\underline{C}_6H_4CF_3$, $C_6H_4\underline{C}F_3$), 133.07 ($=\underline{C}C{\equiv}CC_6H_4CF_3$), 140.78 ($=\underline{C}C{\equiv}CC_6H_4CF_3$), 143.77 ($=\underline{C}(H)Si(iPr)_3$). **^{29}Si NMR** ($CDCl_3$, 79 MHz, δ, ppm): -67.19, -66.96 (SiO_3), -2.47 ($OSi(CH_3)_2$), -2.07 ($Si(iPr)_3$). **FT IR (cm^{-1}):** 2954.5, 2887.0, 1594.5, 1499.7, 1463.8, 1403.5, 1323.1, 1253.2, 1227.3, 1159.3, 1067.6, 882.5, 826.4, 788.3, 735.1, 676.3, 609.2, 524.0, 445.2. **MALDI TOF MS -** (m/z) ([M+Na], (%)): 2039.86.

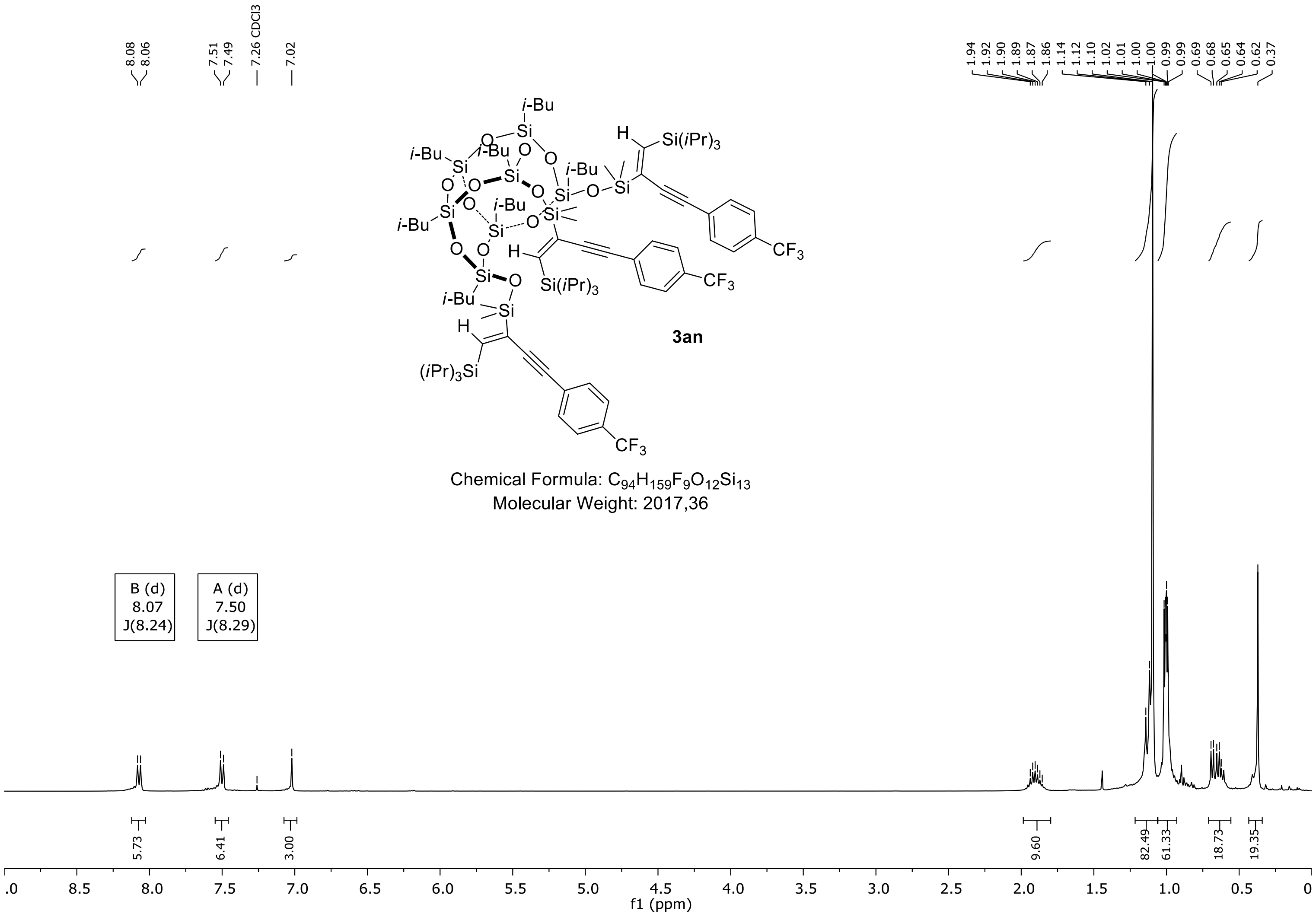

Figure S110. ^{1}H NMR of compound **3an**.

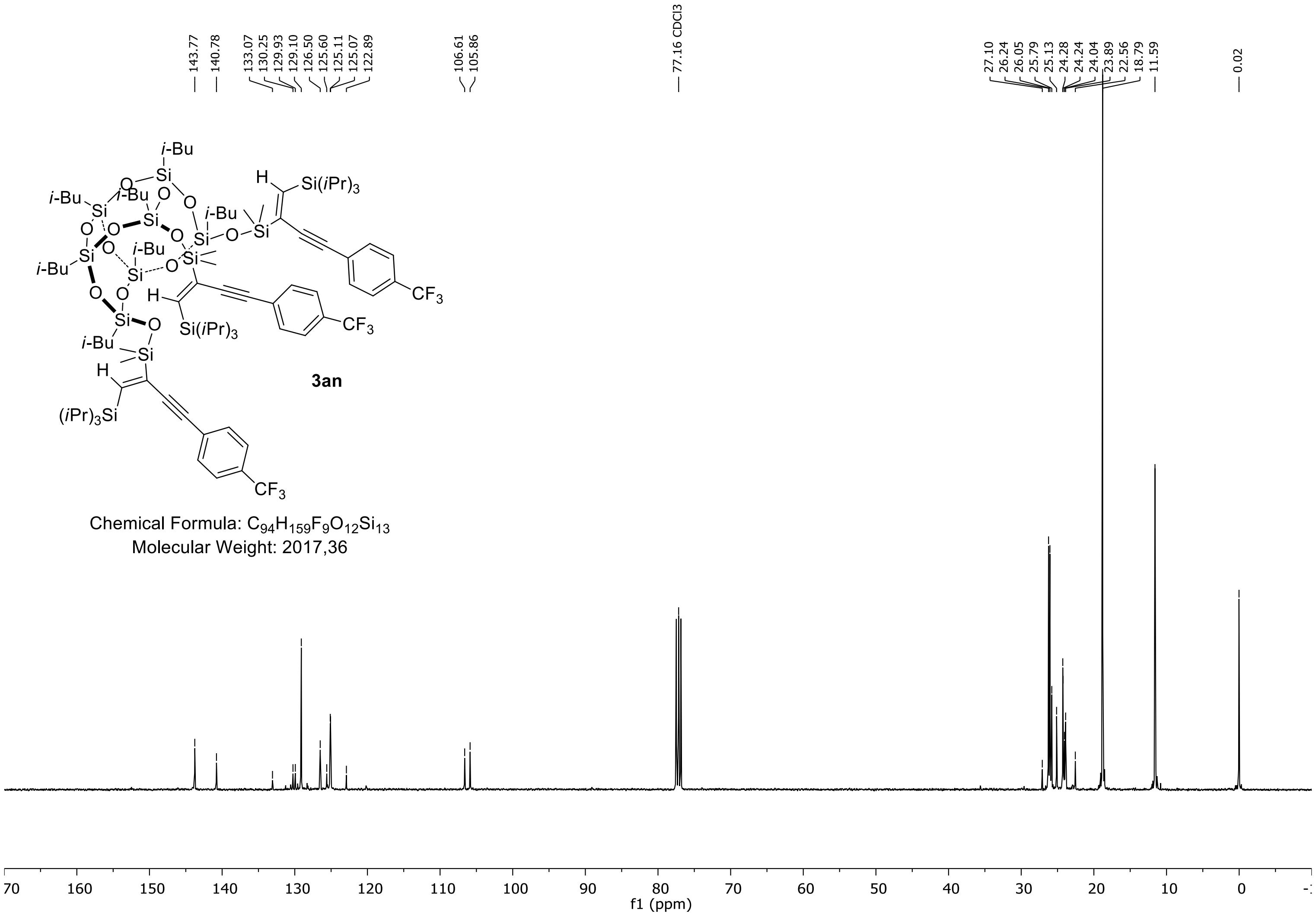

Figure S111. ^{13}C NMR of compound **3an**.

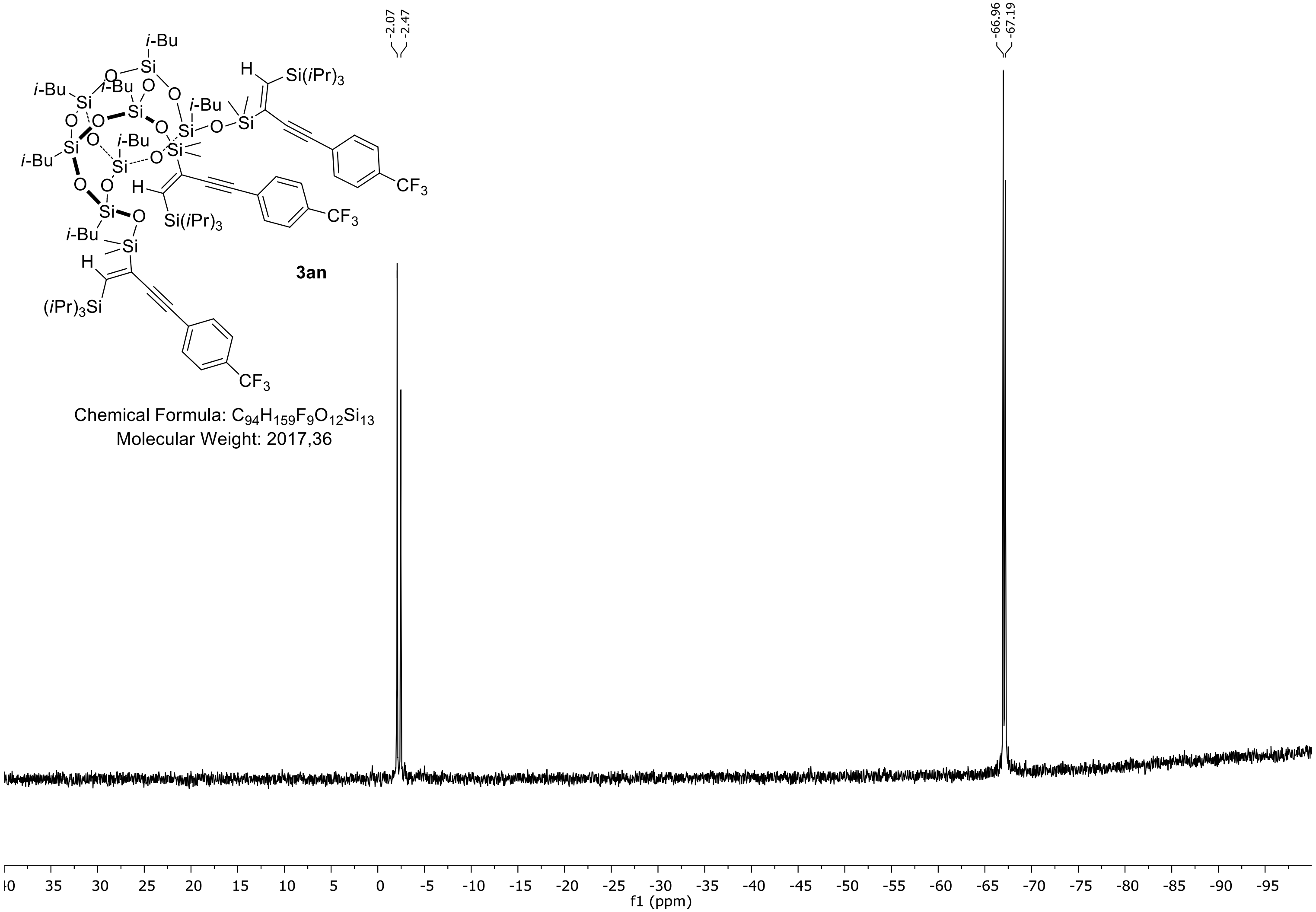

Figure S112. ^{29}Si NMR of compound **3an**.

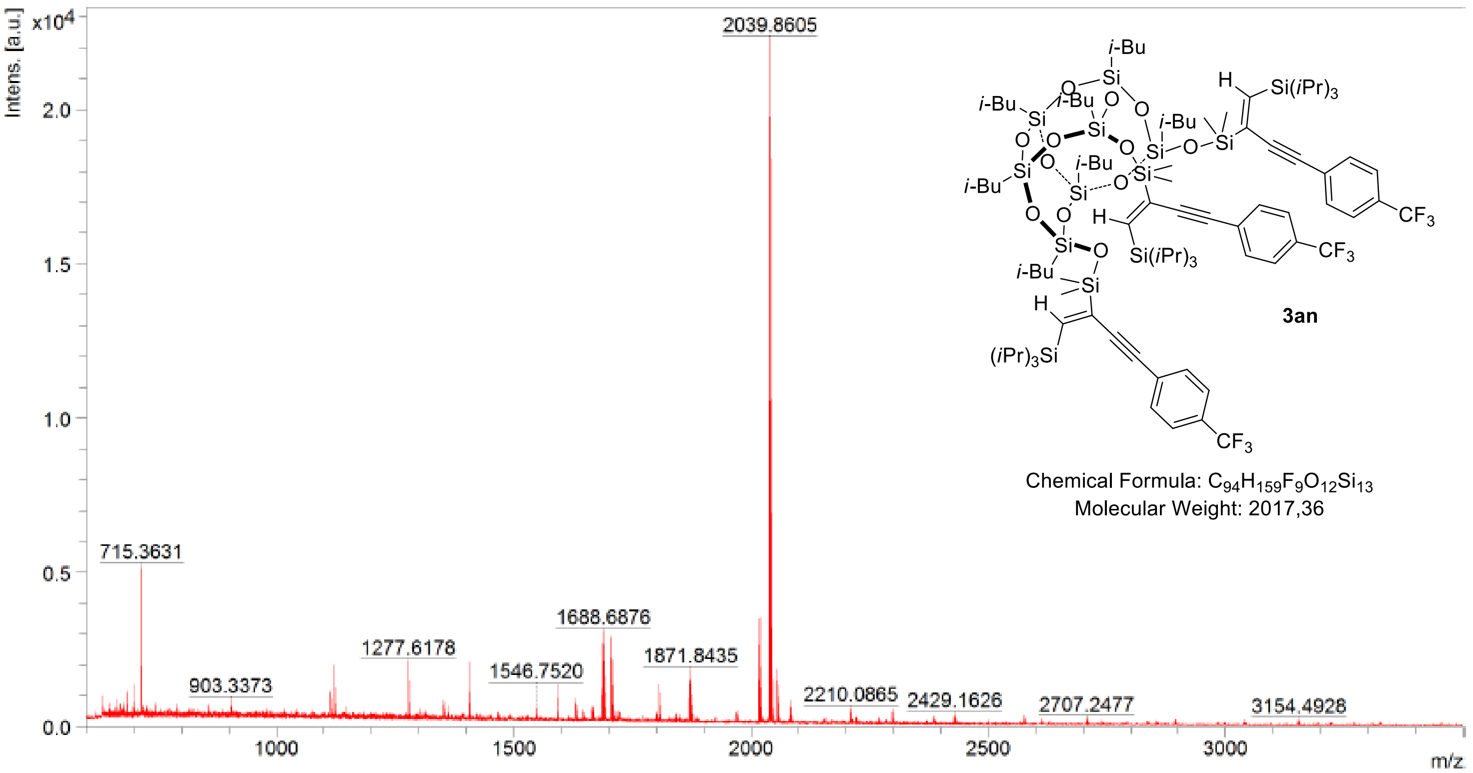

Figure S113. MALDI TOF MS spectra of compound **3an**.

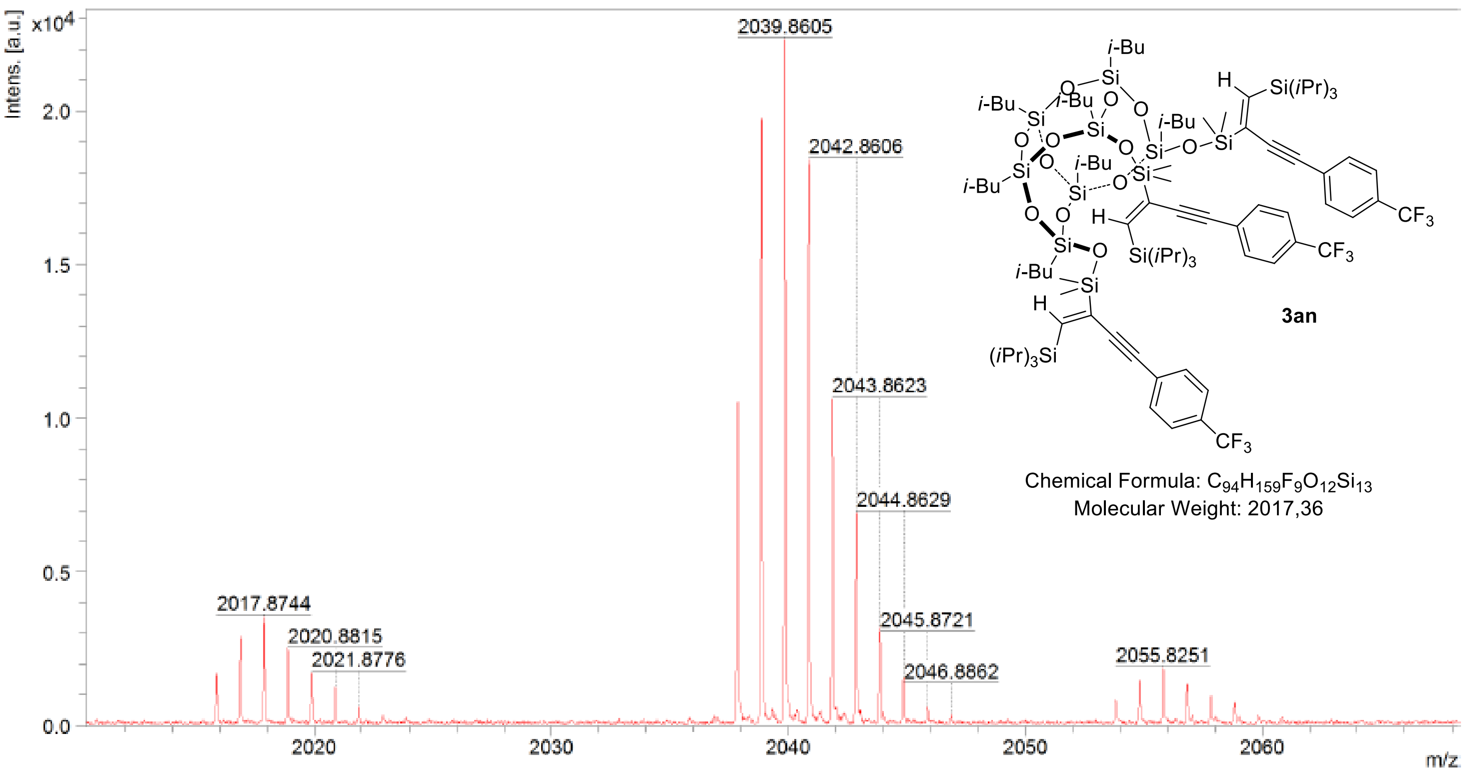

Figure S114. MALDI TOF MS spectra of compound **3an**.
